# Adherence to prehabilitation in adult surgical patients: a systematic review, meta-analysis, meta-regression, and qualitative synthesis

**DOI:** 10.1016/j.bja.2025.06.003

**Published:** 2025-07-04

**Authors:** Marta I. Berrio-Valencia, Mariam Al-Bayati, Adir Baxi, Karina Branje, Ingrid Chitiva-Martinez, Emily Hladkowicz, Gurlavine Kidd, Brian Hutton, Dianna M. Wolfe, Manoj Lalu, Sylvain Boet, Chelsia Gillis, Daniel I. McIsaac, Canadian Prehabilitation Knowledge Network

**Affiliations:** 1 Department of Anaesthesiology and Pain Medicine, University of Ottawa, Ottawa, ON, Canada; 2 Methodological and Implementation Research, The Ottawa Hospital Research Institute, Ottawa, ON, Canada; 3 Patient Partner, The Ottawa Hospital Research Institute, Ottawa, ON, Canada; 4 Department of Anaesthesia, McGill University, Montreal, QC, Canada; 5 School of Epidemiology & Public Health, University of Ottawa, Ottawa, ON, Canada

**Keywords:** adherence, exercise, nutrition, prediction models, prehabilitation, qualitative methods, systematic review

## Abstract

**Background:**

Prehabilitation is hypothesised to play an important role in optimising postoperative outcomes. However, achieving high adherence can be challenging. Our objectives were to synthesise current approaches to adherence measurement and reporting, estimate prehabilitation adherence across trials, identify procedural-, programme-, or patient-level factors associated with adherence, and report barriers and facilitators to adherence.

**Methods:**

Ovid MEDLINE, Embase, the CINAHL, PsycINFO, Web of Science, and the Cochrane CENTRAL Register of Controlled Trials were searched from inception until April 10, 2024. We included randomised trials of adults undergoing major elective surgery allocated to a prehabilitation programme, with at least one binary or continuous measure of adherence to prehabilitation, to an individual component, or both. Random-effects meta-analysis pooled overall adherence rates; meta-regression evaluated predictors of adherence. Qualitative synthesis of reported barriers and facilitators was informed by the Theoretical Domains Framework.

**Results:**

We screened 11 652 titles and abstracts, followed by 1232 full texts, and included 105 trials (*n*=4941). Pooled adherence was 79% (95% confidence interval [CI] 70–88; *I*^2^=95.4%). Substantial qualitative and statistical heterogeneity existed in defining prehabilitation adherence. Only patient age was significantly associated with adherence (per year older: odds ratio 0.95 [95% CI 0.91–0.99]). Based on qualitative synthesis, common barriers were logistical issues and health conditions; facilitators included supervision by specialists and personalisation.

**Conclusions:**

Prehabilitation adherence metrics are variable across trials and standardisation is required to improve reporting and interpretation of prehabilitation evidence. Little credible evidence identifies factors associated with adherence; however, qualitative barriers and facilitators could inform programme design and implementation.

**Systematic review protocol:**

PROSPERO (CRD42024518851).

**Editor’s key points**

• Prehabilitation could meaningfully improve postoperative outcomes; however, effective prehabilitation requires adequate adherence. To what extent adherence is achieved in prehabilitation trials, what factors predict adherence, and what barriers and facilitators to adherence exist is unknown.
• Trials generally achieve >75% adherence; however, definitions are variable, and few patient, procedural, or programme factors strongly predict adherence.
• Consensus is required to inform a definition of prehabilitation adherence. Future prehabilitation programmes should consider identified barriers and facilitators in their designs.

Prehabilitation is a process from diagnosis to surgery, consisting of one or more preoperative exercise, nutrition, psychological, or cognitive interventions (i.e. components), that aims to enhance reserve to improve postoperative outcomes.^1^^,^^2^ Each year, millions of people have major surgery; rates of moderate to severe postoperative complications remain >20%.^3^ Concurrently, a growing number of older adults are presenting for surgery, meaning that the overall risk profile of the perioperative population is increasing.^4^, ^5^, ^6^ Prehabilitation is hypothesised to play an important role in preparing high-risk patients for surgery with the objective of optimising outcomes.^7^

Prehabilitation may improve postoperative functional recovery, reduce complications, and decrease length of stay, although evidence certainty is low.^2^^,^^8^ Although exercise, nutrition, and multicomponent interventions that include exercise are the most likely interventions to improve a variety of outcomes,^8^ inadequate adherence likely decreases efficacy.^9^^,^^10^ In particular, frailty may limit adherence and reduce prehabilitation efficacy.^9^^,^^10^

Although adherence is a top research priority in prehabilitation,^11^ many knowledge gaps remain. Qualitative data describing barriers and facilitators are limited to single trials conducted in a small number of centres amongst unique populations.^12^ Descriptions of how adherence is reported in RCTs, what the overall, range, and component-specific prehabilitation adherence rates are, and what procedural-, programme-, or patient-level factors predict higher adherence are not well explored. To address these gaps, we conducted a systematic review to inform the current state of prehabilitation adherence while informing the future design and implementation of prehabilitation interventions and trials.

## Methods

### Protocol and registration

This was a systematic review, meta-analysis and -regression, and qualitative synthesis of RCTs, designed according to best practice recommendations.^13^ Preferred Reporting Items for Systematic Reviews and Meta-analyses (PRISMA) guidelines guided reporting (Supplementary Appendix 1), along with the Guidance for Reporting Involvement of Patients and the Public-2 (GRIPP-2; Supplementary Appendix 2).^14^^,^^15^ This study is part of an overarching prehabilitation knowledge synthesis programme. This study’s specific protocol was independently developed and prospectively registered on March 11, 2024 (CRD42024518851).

### Patient and public involvement

Design, conduct, and interpretation of this study was informed by patient partners (including on the study executive [GK]) and other knowledge users with lived experience providing, delivering, and studying prehabilitation and perioperative care. Partnership was promoted through integrated knowledge translation^16^^,^^17^ via direct engagement, surveys, emails, and team meetings (Supplementary Appendix 2).

### Search strategy

Our search strategy (Supplementary Appendix 3) was developed with our team’s information specialist, underwent independent peer review,^18^ and was applied to MEDLINE, Embase, CINAHL, PsycINFO, Web of Science, and the Cochrane CENTRAL Register (inception to April 10, 2024). No language exclusions were applied. Grey literature was searched. The references of included studies were also screened.

### Eligibility criteria

We included RCTs addressing a population of adults (≥18 yr) undergoing elective surgery allocated to a prehabilitation programme, consisting of one or more components (e.g. exercise, nutrition, psychosocial, cognitive), and that reported at least one binary or continuous measure of overall adherence to prehabilitation, adherence to each individual component, or both. We excluded studies where prehabilitation was offered for <7 days before surgery,^2^ and quasi-experimental or nonrandomised designs.

### Study selection

Title and abstract, and full-text screening were completed independently in duplicate and piloted on a set of four citations by each reviewer. Citations with conflict between reviewers or marked as unsure were reviewed by the senior author (DIM). The screening process was conducted using DistillerSR® (Evidence Partners Inc., Ottawa, ON, Canada).

### Data extraction and risk of bias assessment

Two investigators (MBV, ICM) independently extracted data in duplicate using a pilot data extraction form designed specifically for this study; 10 studies were piloted for each extractor (Microsoft Excel®; Microsoft Corporation, Redmond, WA, USA). The senior author (DIM) reviewed accuracy and resolved discrepancies. For each study, we extracted pre-specified variables specific to study, procedural, programme, and population characteristics. Any missing data were sought from study authors via e-mail. The Cochrane Risk of Bias 1 (RoB *1.0*) tool^19^ was applied in duplicate (MA, AB, DIM) to assess within-study bias of the included evidence. Disagreements were resolved by discussion with the senior investigator (DIM). We collected all measures of adherence and associated measures of variance, using described methods^20^ to estimate mean and standard deviation from related measures (e.g. medians, interquartile range [IQR], or range).

#### Effect modifiers

From each included study reporting a measure of prehabilitation adherence, we extracted prehabilitation programme, surgical procedural, and population characteristics. A full list of all pre-specified effect modifiers is provided in Supplementary Appendix 4.

### Outcomes

Our primary outcome was any reported measure of adherence to a prehabilitation intervention (continuous or dichotomous). Continuous measures represented the proportion of all prehabilitation tasks completed. Binary measures represented the proportion of individuals meeting a pre-specified threshold (either explicitly described, or based on per-protocol definitions). Where both a continuous and dichotomised measure of adherence were reported, we collected the continuous measure.

Secondary outcomes were any reported barriers or facilitators to prehabilitation adherence. These outcomes were extracted as direct text quotations from the study manuscripts.

### Data syntheses and analyses

Descriptive statistics were used to summarise the overall types of prehabilitation programmes, surgical, and population characteristics. Quantitative syntheses addressed data from studies reporting continuous and binary measures of adherence. A two-sided alpha of 0.05 was used for all analyses. The *I*^2^ statistic was used to quantify the percentage of variability across study effect estimates owing to between-study heterogeneity.^21^

#### Meta-analysis

Random-effects inverse variance meta-analysis was used for all pooled analyses (‘*metafor*’ package; R Foundation for Statistical Computing, Vienna, Austria),^22^ with confidence intervals (CIs) for the summary effect calculated with the Hartung–Knapp–Sidik–Jonkman method, between-study variance estimated using the restricted maximum likelihood method, and estimation of prediction intervals.^23^^,^^24^ As both continuous and binary adherence measures were proportions, we estimated pooled adherence and within-component mean adherence, along with 95% CIs using the ‘*metaprop’* function with a logit transformation.

#### Meta-regression

Where at least 10 studies were available reporting a pre-specified effect modifier, we performed meta-regression (‘*meta’* package*)* to estimate the summary univariable association (mean difference and 95% CI [continuous]; odds ratio [OR] and 95% CI [binary]) of the effect modifier with adherence, and the associated R^2^ statistic as a measure of heterogeneity in adherence explained by the postulated effect modifier. We assessed the credibility of significant effect modifiers using the Instrument to assess the Credibility of Effect Modification Analyses (ICEMAN) in a meta-analysis of RCTs.^25^

### Descriptive and qualitative analyses

From each study reporting adherence, we extracted any text describing how adherence was defined and qualitatively synthesised common approaches.

To synthesise barriers and facilitators, two reviewers (MBV, ICM) assessed an initial set of 10 studies and inductively developed codes to describe common textual references that either explicitly described a barrier or facilitator to adherence, or that described factors that acted as barriers or facilitators, even if such factors were not explicitly described in the study using barrier/facilitator language. With this set of codes, reviewers then coded subsequent study reports to generate an overall frequency count for each code. If new codes were proposed by one reviewer, the two reviewers again met to find agreement. Codes and frequency counts were then reviewed with the senior author (DM) and consensus used to determine if codes represented a single theme, or if some codes could be combined into larger themes. We then deductively mapped themes to at least one domain of the Theoretical Domains Framework (TDF),^26^ a leading framework informing implementation and behaviour change in health care across 14 domains.^26^^,^^27^ Where available, quotes were extracted to support themes.

### Protocol amendments

Protocol deviations are reported in Supplementary Appendix 5.

## Results

### Study selection and characteristics of included studies

After screening 11 652 titles and abstracts, followed by 1232 full texts, we included 105 trials (*n*=4941 participants allocated to prehabilitation) published between 2000 and 2024; 20% (*n*=220) of excluded studies met inclusion criteria but did not report adherence data (PRISMA Diagram, Supplementary Appendix 1; included studies, Table 1; excluded studies, Supplementary Appendix 6). Overall, 76 (72.4%) studies reported adherence as continuous measure, whereas 29 (27.6%) studies reported adherence as a binary outcome. Most studies (*n*=76; 72.4%) reported adherence to exercise (continuous *n*=55; binary *n*=21), followed by 27 (25.7%) nutrition studies (continuous *n*=20; binary *n*=7). Eight studies addressed adherence to a psychosocial intervention (continuous *n*=7; binary *n*=1) and only two to a cognitive component (continuous *n*=1; binary *n*=1).

**Table 1:** Included randomised controlled trials. HI, high-intensity pulmonary exercises; HIT, high-intensity training; IMT, inspiratory muscle training; IMT-E, inspiratory muscle training-endurance; MIT, moderate-intensity training. ∗Multi-arm trials.

Study	Country	N prehab	Female (%)	Type of surgery	Mean age	Adherence reported to the prehab components	Only binary adherence reported	Continuous adherence reported
Wall, 2000	USA	53	46.2	Oncological	62.5	Exercise		✓
Wang, 2002	Australia	15	64	Orthopaedic	65.7	Exercise		✓
Beaupre, 2003	Canada	65	55	Orthopaedic	67	Exercise		✓
Rooks, 2006	USA	54	57.4	Orthopaedic	65	Exercise		✓
Nielsen, 2008	Denmark	28	60	Orthopaedic	52	Exercise		✓
Gill, 2009∗	Australia	42, 42	71.6, 69.2	Orthopaedic	70.3	Exercise		✓
Tibaek, 2009	Denmark	26	0	Major non-oncological	68	Exercise	✓	
Carli, 2010	Canada	58	42	Mixed	60	Exercise	✓	
Dronkers, 2010	Netherlands	22	26.2	Oncological	68.8	Exercise		✓
Hoogeboom, 2010	Netherlands	10	66.6	Orthopaedic	75	Exercise		✓
Benzo, 2011	USA	10	50	Oncological	70.2	Exercise		✓
Bitterli, 2011	Switzerland	41	38.8	Orthopaedic	66.8	Exercise		✓
Burden, 2011	UK	54	37.9	Oncological	65.3	Nutrition	✓	
van Nieuwenhove, 2011	Western Europe (Sweden, Lithuania, Spain, Belgium, and Netherlands)	137	70	Major non-oncological	40	Nutrition	✓	
Brown, 2012	USA	17		Orthopaedic		Exercise		✓
McKay, 2012	Canada	10	59	Orthopaedic	60.5	Exercise		✓
Oosting, 2012	Netherlands	15	80	Orthopaedic	75	Exercise		✓
Parikh, 2012	USA	29	84	Major non-oncological	46.2	Nutrition		✓
Morano, 2013	Brazil	12	62.5	Oncological	68.8	Exercise		✓
Soares, 2013	Brazil	16	46.9	Mixed	55	Exercise		✓
Falewee, 2014	France	73	16	Oncological	59.5	Nutrition	✓	
Gillis, 2014	Canada	38	37.6	Oncological	65.8	Nutrition		✓
Jensen, 2014	Denmark	50	26.2	Oncological	71	Exercise	✓	
Matassi, 2014	Belgium	61	48.3	Orthopaedic	67	Exercise		✓
van Adrichem, 2014∗	Netherlands	20, 19	25.6	Oncological	62	Exercise: • IMT-HI • IMT-E		✓
Zeng, 2014	China	32	47.5	Orthopaedic	64.8	Exercise		✓
Reynolds, 2015	USA	36		Oncological		Exercise		✓
Huber, 2015	Switzerland	22	47	Orthopaedic	70.3	Exercise	✓	
Ruiz-Tovar, 2015∗	Spain	20, 20	75	Major non-oncological	43	Nutrition: • High protein • Immunonutrition		✓
Dunne, 2016	UK	20	29.7	Oncological	62	Exercise		✓
Gade, 2016	Denmark	19	51	Oncological	69	Nutrition	✓	
Gillis, 2016	Canada	22	34.9	Oncological	69.1	Nutrition		✓
Kalarchian, 2016	USA	71	90.2	Major non-oncological	44.9	Psychosocial		✓
Licker, 2016	Switzerland	74	40	Oncological	64	Exercise		✓
Moya, 2016	Spain	122	46.3	Oncological	68	Nutrition		✓
Palma-Milla, 2016	Spain	20	16	Oncological	59.6	Nutrition	✓	
Rolving, 2016	Denmark	59	56.7	Orthopaedic	47.7	Psychosocial		✓
Lluch, 2017	Spain	22	59	Orthopaedic	67.7	Exercise		✓
Baillot, 2017	Canada	13	80	Major non-oncological	41.1	Exercise		✓
Huang, 2017∗	China	30, 30	31.7,30	Oncological	63.6	Exercise: • HI • IMT	✓	
Ida, 2017	Japan	63	28	Oncological	65.6	Nutrition		✓
Lindback, 2017	Sweden	99	53	Orthopaedic	59	Exercise	✓	
Tew, 2017	UK	27	5.6	Cardiovascular	74.9	Exercise		✓
Bousquet-Dion, 2018∗	Canada	37	27	Oncological		Nutrition		✓
						Exercise		
Contreras, 2018	Spain	43	67.5	Major non-oncological	45	Nutrition	✓	
Santa Mina, 2018	Canada	44	0	Oncological	62.2	Exercise	✓	
Minnella, 2018	Canada	26	25.4	Oncological	68	Nutrition + exercise		✓
Moug, 2018	UK	24	35	Oncological	46	Exercise		✓
Mudge, 2018∗	Australia	69	18.5	Oncological	64.6	Nutrition: • Perioperative • Preoperative		✓
Valkenet, 2018	Netherlands, Belgium, Ireland, Finland	120	39	Oncological	62.7	Exercise		✓
Barth, 2019	USA	30	55	Oncological	54	Nutrition	✓	
Hjelmesæth, 2019	Norway	28	70	Major non-oncological	42.4	Psychosocial		✓
Hollis, 2019	Australia	23	63	Major non-oncological	51.6	Nutrition		✓
Karlsson, 2019	Sweden	10	62	Oncological	76	Exercise		✓
Lai, 2019	China	34	49	Oncological	63.4	Exercise	✓	
Milios, 2019	Australia	50	0	Oncological	63.5	Exercise		✓
Ruiz-Tovar, 2019	Spain	20	62	Major non-oncological	45.9	Nutrition		✓
Birch, 2020	Denmark	31	66.7	Orthopaedic	66	Psychosocial		✓
Blackwell, 2020	UK	19		Oncological	71	Exercise	✓	
Blasco, 2020∗	Spain	25,26	50,56	Orthopaedic	70.9	Exercise: • Hospital • Home		✓
Carli, 2020	Canada	55	52.7	Oncological	82	Exercise		✓
Domínguez-Navarro, 2020∗	Spain	24, 20	58.3,65	Orthopaedic	70.8, 70.4	Exercise: • Strengthening • Strengthening + balance		✓
Ferreira, 2020∗	Canada	52	46	Oncological	66.5	Nutrition		✓
						Exercise		
Granicher, 2020	Switzerland	10	40	Orthopaedic	67.3	Exercise		✓
Holsgaard-Larsen, 2020	Denmark	40	65	Orthopaedic	70.8	Exercise		✓
Humeidan, 2020	USA	125	64.9	Mixed	67	Cognitive	✓	
Laurent, 2020	France	14	30.7	Oncological	62.9	Exercise	✓	
Liu, 2020 (1)	China	26	30	Oncological	64.5	Nutrition		✓
Liu, 2020 (2)	China	37	68.4	Oncological	56.2	Exercise	✓	
Minnella, 2020∗	Canada	21, 21	52.4, 23.8	Oncological	67	Nutrition: • On HIT • On MIT		✓
						Exercise: • On HIT • On MIT		
Northgraves, 2016	UK	10	47.6	Oncological	63.5	Exercise		✓
O'Gara, 2020	USA	20	27.5	Cardiovascular	69	Cognitive		✓
Allen, 2021∗	UK	26	45.9	Oncological	64	Exercise		✓
						Psychosocial		
Aragoncillo Sauco, 2021	Spain	53	24.6	Cardiovascular	66.3	Exercise		✓
Gravier, 2021	France	18	36	Oncological	65	Exercise		✓
Heiman, 2021	Sweden	200	100	Oncological	63	Exercise	✓	
Hernon, 2021	UK	68	31.2	Oncological	69.1	Exercise	✓	
Mathew, 2021	Norway	75	100	Oncological	60.6	Exercise	✓	
Marchand, 2021	Canada	35	41	Major non-oncological	71.6	Exercise		✓
Minnella, 2021	Canada	35	70	Oncological	66	Nutrition + exercise		✓
Przkora, 2021	USA	6	70	Orthopaedic	67.2	Exercise		✓
Yau, 2021	China	34		Cardiovascular		Exercise		✓
Schulz, 2021	Canada	9	0	Oncological	66	Exercise	✓	
Steffens, 2021∗	Australia	11	45.5	Oncological	63	Exercise: • Supervised • Home		✓
Tenconi, 2021	Italy	70	38.6	Oncological	66.8	Exercise	✓	
Diaz-Feijoo, 2022∗	Spain	15	100	Oncological	60	Nutrition		✓
						Exercise		
						Psychosocial		
Klek, 2022	Poland	136	43.5	Oncological	60.8	Nutrition		✓
McIsaac, 2022	Canada	94	57	Oncological	74	Exercise		✓
McLean, 2022	Canada	51	100	Major non-oncological	52	Exercise	✓	
Nguyen, 2022	France	131	68	Orthopaedic	68.6	Exercise		✓
Onerup, 2022	Sweden	317	40	Oncological	68	Exercise	✓	
Waller, 2022	UK	11	63.6	Oncological	61	Exercise		✓
Paul, 2022	Netherlands	65	75	Major non-oncological	41.4	Psychosocial	✓	
Serrano, 2022	Canada	36	40.8	Oncological	63	Nutrition		✓
Woodfield, 2022	New Zealand	28	39.7	Major non-oncological	66	Exercise		✓
Akowuah, 2023	UK	91	17.5	Cardiovascular	64.7	Exercise	✓	
Atoui, 2023∗	Canada	43	48.3	Oncological	64.4	Nutrition		✓
						Exercise		
Kasvis, 2023	Canada	17	29.4	Oncological	63.6	Psychosocial		✓
Molenaar, 2023	Netherlands	123	49.6	Oncological	69	Exercise	✓	
Singh, 2023	Australia	17	0	Oncological	64.5	Exercise		✓
Tan, 2023	Singapore	31	69.3	Oncological	61	Nutrition		✓
Bojesen, 2023	Denmark	16	31	Oncological	79	Exercise		✓
						Nutrition		
Ninomiya, 2023	Japan	29		Orthopaedic	70.5	Exercise		✓
Li, 2024	China	68	31.6	Oncological		Exercise		✓
						Nutrition		
Granicher, 2024	Switzerland	10	80	Orthopaedic	72.7	Exercise		✓

For studies reporting adherence as a continuous measure, 32.5% (*n*=1606) of participants underwent oncological surgery and 17.5% (*n*=865) orthopaedic surgery. Median age was 65 yr and 53.6% of participants were female. For studies reporting adherence as a binary measure, 26.6% (*n*=1316) underwent oncological surgery and 2.4% (*n*=121) orthopaedic surgery. Median age was 64 yr and 49.6% of participants were female. Overall, most studies (*n*=77; 73.3%) were high risk of bias, 19 (18.1%) had unclear risk of bias, and nine (8.6%) were rated as low risk of bias. Factors contributing to high risk of bias were lack of blinding of participants, personnel, and standard care of participants (Supplementary Appendix 7).

### Adherence definitions

Of included studies, 65 (61.9%) provided explicit definitions of adherence, which were heterogeneous. Adherence was most often reported by component (*n*=100; 95.2%). Five studies (4.8%) reported multicomponent adherence. Methods used to ascertain adherence were reported in 61 studies, with 20 (32.8%) using logbooks only, five (8.2%) using phone calls only, and 36 (59%) using combined methods, typically logbooks plus phone calls.

Of the 72 studies reporting adherence to exercise interventions, 33 (45.8%) defined adherence based on completion of prescribed exercise, whereas 24 (33.3%) defined adherence based on attendance at exercise sessions. For studies that dichotomised exercise adherence definitions, the median threshold defining adherence was 75% (IQR 75–100).

Of the 27 studies reporting adherence to nutritional interventions, 17 (63%) definitions were based on patient self-report of supplement consumption, and three (11%) were based on verification of supplement consumption by the research team. The median threshold to define adherence was 75% (IQR 70–100).

### Overall adherence to prehabilitation interventions

**Fig 1:**
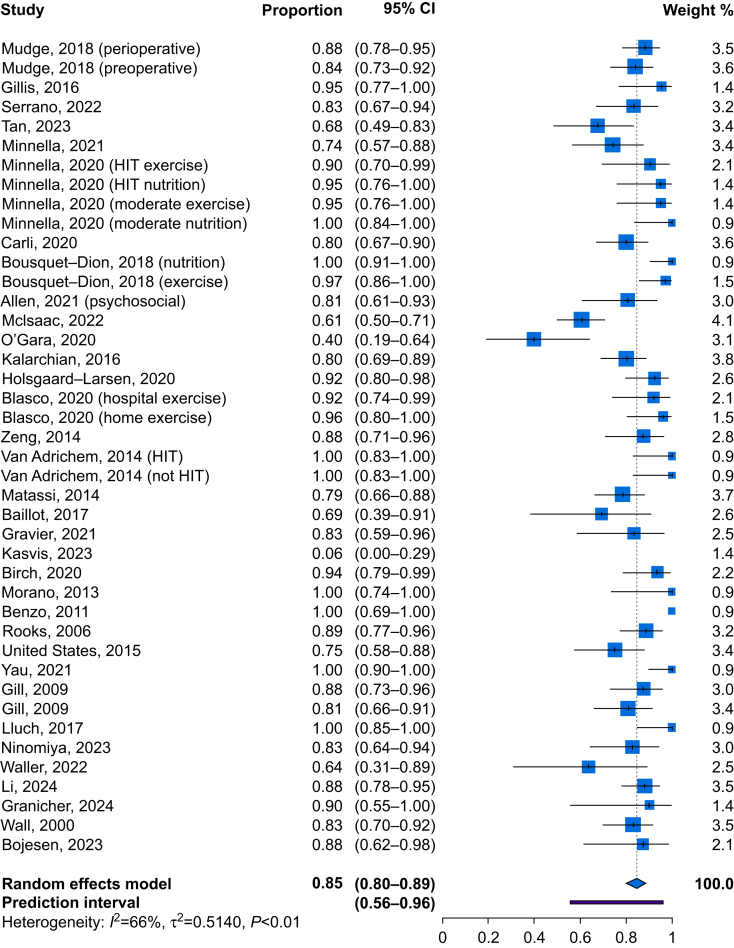
Forest plot for continuous adherence. CI, confidence interval; HIT, high-intensity training.

From studies reporting any type of adherence as a continuous measure, the pooled mean adherence was 79% (95% CI 70–88; *I*^2^=95.4%; Fig. 1). For exercise interventions, the pooled mean adherence was 85% (95% CI 80–89; *I*^2^=64%). For nutrition interventions, pooled mean adherence was 83% (95% CI 74–89; *I*^2^=73%).

**Fig 2:**
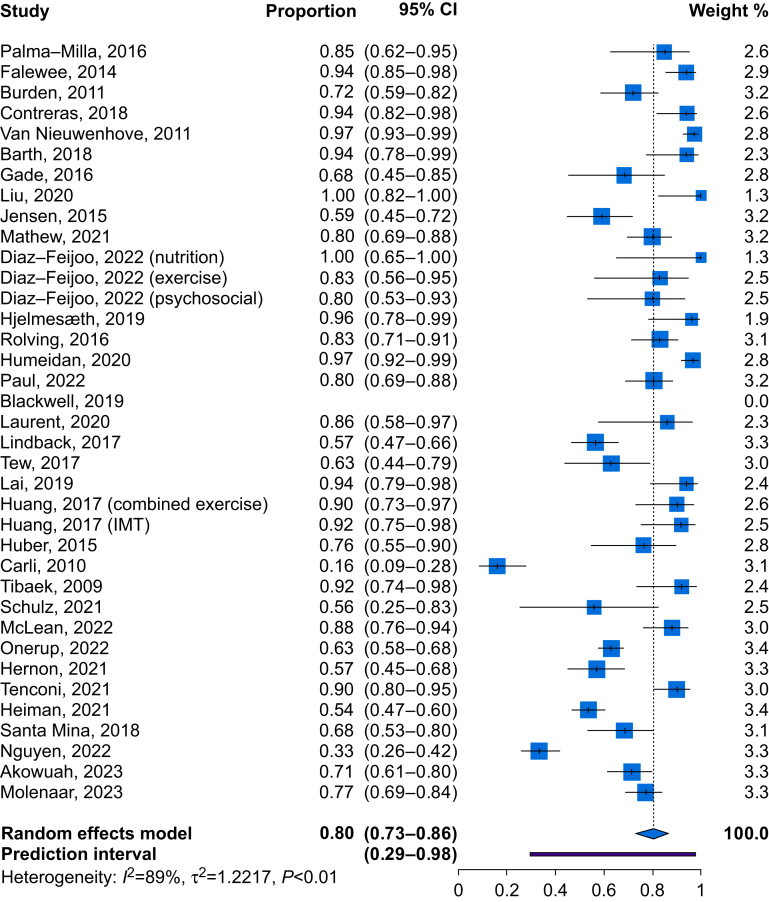
Forest plot for binary adherence. CI, confidence interval; IMT, inspiratory muscle training.

Where studies reported adherence using a threshold, the overall proportion achieving adherence was 80% (95% CI 73–86; *I*^2^=89%; Fig. 2). For exercise interventions, 74% (95% CI 63–82; *I*^2^=88%) achieved adherence, whereas for nutrition, 85% (95% CI 76–91; *I*^2^=76%) achieved adherence.

### Predictors of adherence

#### Mean adherence to prehabilitation (continuous)

Across all types of prehabilitation interventions, no procedural-, programme-, or patient-level factors were significantly associated with mean adherence to prehabilitation. Only programme supervision (17%) explained more than 10% of observed heterogeneity (Table 2).

**Table 2:** Predictors of continuous adherence for prehabilitation. Effect modifier *P*-value represents the type III *P*-value for the effect modifier. CI, confidence interval; ERAS, Enhanced Recovery After Surgery; MD, mean difference; OSS, Operative Stress Score.

Effect modifier		Effect modifier *P*-value	MD	95% CI			R^2^ (%)
**Procedural factors**							
Procedure type		0.239					1.5
(*vs* orthopaedic)	Cardiac/Vascular		–46.6	–92.31	to	–0.97	
	Oncological		–9.6	–32.74	to	13.60	
	Other		–13.2	–51.01	to	24.57	
Operative Stress Score		0.797					0.0
(*vs* OSS 2)	OSS 3		–6.1	–41.52	to	29.26	
	OSS 4		–8.8	–40.29	to	22.51	
ERAS (*vs* not)		0.077					7.7
			18.5	–2.17	to	39.27	
**Patient factors**							
Age (per year)		0.777	0.1	–1.03	to	1.36	0.0
Female sex (per 1% increase)		0.920	0.0	–0.51	to	0.45	0.0
Presence of cancer		0.919					0.0
(*vs* none)	Cancer patients		1.0	–18.86	to	20.84	
**Programme factors**							
Location		0.589					0.0
(*vs* home)	Facility		12.9	–13.47	to	39.40	
	Combined		2.2	–18.92	to	23.30	
Supervision		0.048					17.0
(*vs* self-directed)	Coached		16.5	–4.64	to	37.74	
	Combined		–9.0	–31.89	to	13.84	
Personalised programme (*vs* not)		0.550	–5.4	–23.56	to	12.85	0.0
Motivational technique (*vs* none)		0.891	–1.7	–26.95	to	23.57	0.0
Format		0.793					0.0
	Combined		7.0	–47.32	to	61.32	

#### Proportion adherent to prehabilitation (binary)

Among postulated programme-, procedural-, and patient-level effect modifiers, only patient age was significantly associated with the odds of adherence to prehabilitation (per year older: OR 0.95 [95% CI 0.91–0.99]; R^2^=13.30%, *P*=0.025; low credibility; Supplementary Appendix 8). Only age (13%), surgery type (14%), and programme personalisation (*vs* none: 12%) explained more than 10% of observed heterogeneity (Table 3).

**Table 3:** Predictors of binary adherence for prehabilitation. The ORs and 95% CIs represent the association compared with a pre-specified reference category or per unit change. CI, confidence interval; ERAS; Enhanced Recovery After Surgery; OR, odds ratio; OSS; Operative Stress Score.

Effect modifier		Effect modifier *P*-value	OR	95% CI			R^2^ (%)
**Procedural factors**							
Procedure type		0.127					14.1
(*vs* orthopaedic)	Major non-oncological		1.63	0.36	to	7.45	
	Cardiac/vascular		1.23	0.20	to	7.60	
	Oncological		2.38	0.74	to	7.68	
	Mixed		17.82	1.48	to	215.08	
	Other		5.36	1.12	to	25.69	
Operative Stress Score		0.620					0.0
(*vs* OSS 2)	OSS 3		1.81	0.43	to	7.68	
	OSS 4		1.29	0.34	to	4.90	
ERAS (*vs* not)		0.871					0.0
			0.90	0.26	to	3.14	
**Patient factors**							
Age (per year)		**0.025**	0.95	0.91	to	0.99	13.3
Female sex (per 1% increase)		0.451	1.01	0.99	to	1.02	0.0
Presence of cancer		0.245					2.9
(*vs* none)	Cancer patients		0.66	0.32	to	1.35	
							
**Programme factors**							
Location		0.954					0.0
(*vs* home)	Facility		0.85	0.28	to	2.57	
	Combined		0.91	0.32	to	2.57	
Supervision		0.191					3.7
(*vs* self-directed)	Coached		0.89	0.34	to	2.34	
	Combined		0.39	0.13	to	1.18	
Personalised programme (*vs* not)		0.080	0.50	0.23	to	1.09	12.3
Motivational technique (*vs* none)		0.540	1.30	0.54	to	3.13	0.0
Format		0.818					0.0
	Group		0.69	0.17	to	5.54	
	Combined		0.71	0.23	to	2.14	

### Barriers and facilitators to adherence to prehabilitation

Based on qualitative synthesis, we identified 10 barriers and facilitators themes, which were mapped to nine of 14 TDF domains. Frequency counts and supporting quotes are presented in Supplementary Appendix 9.

#### Theme 1: health condition

For exercise adherence, acute and chronic medical conditions (e.g. colds, flu, exacerbation of chronic pulmonary disease) were reported as barriers. Symptom burden, such as fatigue and pain, also arose as barriers. For nutritional adherence, gastrointestinal side-effects decreased adherence to supplement consumption.

#### Theme 2: personal factors

Across types of prehabilitation, personal feelings of being overwhelmed, anxiety, and time constraints were identified as barriers. For cognitive prehabilitation, some participants expressed challenges with focus, and completing numerous assigned tasks in a timely manner.

#### Theme 3: logistical issues

Logistical issues were common barriers, including a lack of access to transport for participants in hospital-based exercise programmes, and lack of technology access for cognitive prehabilitation participants. For participants receiving nutritional supplements, having to wait to receive supplements at a pharmacy was a barrier.

#### Theme 4: social influences

Lack of family or social support was the most common social barrier. Social isolation and lack of family support, competing family or social commitments, and work responsibilities impeded adherence to prehabilitation. Conversely, exercise engagement was facilitated by the presence of a support person (e.g. spouse, family member), especially if they participated in training with the participant.

#### Theme 5: supervision by specialists

Full or partial supervision was a facilitator of adherence to exercise and nutrition (e.g. dietitians or physiotherapists following individual progression). Participants expressed feeling cared for when their exercise was supervised, and interaction with a coach represented an important social contact for individuals living alone. Supervision also helped to make exercise programmes more manageable.

#### Theme 6: training locations including home-based programmes

Exercise programmes delivered at home facilitated adherence and helped to overcome barriers to transportation expressed by some participants.

#### Theme 7: personalisation

Intervention personalisation was a facilitator. Approaches to personalisation included informing programming using baseline functional status (either self-reported, or quantified using exercise testing), and health history (e.g. comorbidities and pain conditions). Other approaches included individualised strategies to manage exercise-related pain symptoms, and providing recipe tips and additives to improve the palatability of nutritional supplements.

#### Theme 8: adequate access to materials, supplements, and equipment

Having adequate and patient-friendly programme materials (e.g. instructional booklet or video) was a facilitator to exercise adherence, as was the use of activity trackers such as pedometers and heart rate monitors. For nutritional supplements, providing supplements along with shaker jugs (to mix protein powders with liquids) improved adherence. Provision of a tablet-based brain-training application facilitated cognitive prehabilitation adherence.

#### Theme 9: resources to support engagement and motivation

Providing education, skill building, and supporting intrinsic motivation facilitated adherence. For example, implementing structured education about stress and anxiety management, decreasing fear avoidance behaviours, resilience profiling, goal setting, and re-enforcing the positive effects of exercise were mentioned as enablers.

## Discussion

In this systematic review, meta-analysis, meta-regression, and qualitative synthesis, we identified substantial quantitative, statistical, and operational heterogeneity in adherence definitions in prehabilitation RCTs and the extent to which intervention adherence was achieved. Although we estimate that participants in prehabilitation trials typically complete 85% of prescribed intervention activities, and that 80% meet defined thresholds, heterogeneity in adherence was high and 20% of identified trials did not report adherence. Despite pre-specifying a set of procedural-, programme-, and patient-level factors, only increased patient age was associated with adherence (decreased), and this estimate had low credibility. In contrast, several qualitative barriers and facilitators to adherence were identified and aligned with many postulated predictors. Together, these findings highlight the need to clearly and consistently report adherence using standardised methods along with standardised reporting of key patient and programme characteristics. Identified barriers and facilitators could be used to enhance design and implementation of prehabilitation programmes.

Despite being a top priority in prehabilitation science,^11^ our results indicate that measurement and reporting of prehabilitation adherence is variable. Variation existed in whether adherence was reported, with 20% of eligible studies excluded for not reporting adherence. Where adherence was reported, studies often did not provide a definition, used definitions that were based on attendance (as opposed to completion of prescribed interventions), and typically relied on self-report. Many studies reported adherence dichotomously as opposed to reporting a more information-rich continuous measure. As prehabilitation research continues to be prioritised by patients, the public, and clinicians, efforts are warranted to develop consensus guidelines and definitions for adherence measurement and reporting. Ideally, this process would meaningfully engage patients, clinicians, researchers, and other interest holders to ensure a meaningful and balanced set of recommendations. Fortunately, where reported, available data suggested that high levels of adherence can be achieved, which aligns with recent knowledge synthesis reporting consistent benefit from prehabilitation in improving clinical outcomes.^8^ However, these promising adherence levels should be considered in context of the underlying trials, which were mostly single centre with high or unclear risk of bias.

Adherence demonstrated statistical heterogeneity, with *I*^2^ values for pooled estimates exceeding 70% in all meta-analyses, including those limited to a single component (e.g. exercise only). This likely reflects inconsistency across adherence definitions, and variations in prehabilitation programmes, surgical procedures, and patient characteristics. A lack of strong associations could have several explanations, including heterogeneity in how factors were measured and reported within trials, or our use of aggregate (i.e. study-level) variables, in contrast to individual patient data.^28^ A lack of consistent reporting of factors including frailty, malnutrition, and comorbidity precluded their analysis, which previous trials suggest are likely important modifiers of prehabilitation adherence.^9^^,^^29^

Our qualitative synthesis identified consistent themes. Several barriers were related to patient-level factors, including acute and chronic medical conditions, the presence of anxiety, a lack of social support, and lack of time to complete prehabilitation tasks. At a programme level, logistical considerations, such as accessing facility-based programmes, were barriers, whereas provision of home-based programming facilitated adherence. Although supervision further facilitated adherence, advancing virtual technology, including incorporation of wearable sensors, will likely be required to provide home-based programming with adequate supervision and could help to quantify adherence with greater precision. As the field continues to develop, use of innovative designs such as Sequential, Multiple Assignment, Randomised Trial (SMART) designs could play a role in advancing intervention design to optimise adherence.^30^ Briefly, SMART designs are multistage, factorial randomised trials where participants can be randomised at multiple points. In the setting of prehabilitation, this could allow participants to undergo sequential, adaptive testing of promising adherence optimisation strategies over the course of their enrolment dependent on their adherence levels.

### Strengths and limitations

Our review involved patient and public engagement from inception to reporting. Our peer-reviewed search included six databases, all the stages of the review were performed in duplicate, and no language restrictions were applied. Limitations include heterogeneity in pooled adherence definitions and pooled estimates. Adherence to psychosocial and cognitive components was poorly reported. Although inclusion was limited to RCTs, stratified randomisation of predictors in RCTs was not conducted; therefore, confounding (e.g. the association of other or unmeasured factors with adherence) in our estimated associations was possible. Reported barriers and facilitators were extracted based on data that typically lacked direct participant perspective.

### Conclusions

Adherence to prehabilitation is generally high; however, reporting quality and adherence definitions are heterogeneous. Although only age was identified as a significant predictor of adherence, a lack of reporting of key patient characteristics such as frailty limits certainty. Future interventions and implementation programmes should aim to overcome logistical and social support-related barriers while optimising delivery of home-based interventions with adequate supervision and material support.

## Authors’ contributions

Conception of the work: DIM, GK

Conception, interpretation, and review: GK

Design of the work and data interpretation: DIM, MBV, EH, GK, BH, DMW, ML, SB, CG

Data acquisition: DIM, MBV, ICM, MA, AB, KB

Data analysis: DIM

Writing and review of the manuscript: DIM, MBV, GK

## Funding

Canadian Institutes of Health Research (CIHR) grant number 202109PJK by DIM; Department of Anesthesiology & Pain Medicine at The Ottawa Hospital provided access to DistillerSR software.

## Declaration of interest

DIM receives salary support from a Clinical Research Chair at the University of Ottawa Faculty of Medicine, from the Physician Services Inc. Mid-Career Knowledge Translation Fellowship, and from The Ottawa Hospital Anesthesia Alternate Funds Association. The other authors declare no conflict of interest.

## Appendix 1 Canadian Prehabilitation Knowledge Network Group

Laura Boland, Sylvie Aucoin, Rebecca Auer, Paul Beaule, Gregory Bryson, Franco Carli, Antoine Eskander, Dean Fergusson, Julio Fiore Jr, Alan Forster, Jayna Holroyd-Leduc, Rachel Khadaroo, Guillaume Martel, Colin McCartney, Julie Nantel, Stephane Poitras, Celena Scheede-Bergdahl, Monica Taljaard, Areti Angeliki Veroniki, Duminda Wijeysundera, Shamsuddin Akhtar, Marlis Atkins, Carlota Basualdo-Hammond, Mary Brindle, Melani Gillam, Leah Gramlich, Timothy Jackson, Cameron Love, Dolores McKeen, Amanda Meliambo, Husein Moloo, Ronald Moore, John Muscedere, Risa Shorr, Tom Wallace, Alexa Grudzinski, Al Crawford, Mandy Li, Cian Devine, Taylor Gilmore, Rahul Mor, Julien LeBlanc, David Schramm, Lana Ramic, Emily Liang, Nathaniel Neilipovitz, Samantha Dunlop.
