# Supplementary material for: Adherence to prehabilitation in adult surgical patients: a systematic review, meta-analysis, meta-regression, and qualitative synthesis
Source: Br J Anaesth. 2025 Jul 4;135(3):582–93. doi: 10.1016/j.bja.2025.06.003 (PMC12489362; doi:10.1016/j.bja.2025.06.003)
Supplement: Multimedia component 1 [file mmc1.docx]

**Study appendices and supplemental data**

**Table of contents**

**Appendix 1 –** PRISMA Flow Diagram ……………………...……………………………………………...

**Appendix 2 –** GRIPP2 Reporting Checklist…………………………………………………………………

**Appendix 3 –** Search strategies for all included databases………………………………………………….

**Appendix 4 –** Data points extracted from each randomised clinical trial………………………………..…

**Appendix 5 –** Protocol deviations…………………………………………………………………………...

**Appendix 6 –** Excluded randomised clinical trials ……………………...…………………………………

**Appendix 7 –** Trial Risk of Bias…………………………………………………………………………….

**Appendix 8 -** Credibility of Effect Modification Analyses (ICEMAN) in a meta-analysis of randomized controlled trials……………………………………………………………………………………………….

**Appendix 9** - Frequency of themes and overarching quotes for barriers and facilitators…...……………………………………………………………………………………………….

**Appendix 1 – Preferred Reporting Items for Systematic Reviews and Meta-Analyses Flow Diagram**

**Identification of studies via databases and registers**

Records removed *before screening*:

Duplicate records removed (n = 4,966)

Records marked as ineligible by automation tools (n = 0)

Records removed for other reasons (n = 0)

Records identified from original search*:

Databases (n = 11,652)

**Identification**

Records excluded**

(n = 5,454)

Records screened

(n =6,686)

Reports not retrieved

(n = 0)

Reports sought for retrieval (n = 1,232)

**Screening**

Reports excluded:

Duplicate (n = 117)

Intervention not preoperative (n = 36)

No results (n = 67)

Not randomized trial (n = 85)

Not prehabilitation (n = 88)

Protocol (n = 61)

Single risk factor management (n = 16)

Unregistered abstract (n = 15)

Wrong comparator group (n = 5)

Wrong or unclear duration (n = 209)

Wrong or No outcome (n = 160)

Wrong population (n = 18)

Not reporting adherence (n= 220)

Total (n = 1,098)

Reports assessed for eligibility (n = 1,232)

Forward search citations assessed for eligibility (n = 1)

Studies included in review

(n = 105)

Reports of included studies

(n = 135)

**Included**

**Appendix 2 – GRIPP2 Reporting Checklist**

| **Section and topic** | **Reported on page number** |
| --- | --- |
| **1: Abstract of paper** |  |
| 1a: Aim | 2 |
| 1b: Methods | 2 |
| 1c: Results | 2 |
| 1d: Conclusions | 2 |
| 1e: Keywords | 2 |
| **2: Background to paper** |  |
| 2a: Definition | 3 |
| 2b: Theoretical underpinnings | 3 |
| 2c: Concepts and theory development | 3 |
| **3: Aims of paper** |  |
| 3: Aim | 3 |
| **4: Methods of paper** |  |
| 4a: Design | 3 |
| 4b: People involved | 4 |
| 4c: Stages of involvement | 4 |
| 4d: Level or nature of involvement | 4 |
| **5: Capture or measurement of PPI impact** |  |
| 5a: Qualitative evidence of impact | 4 |
| 5b: Quantitative evidence of impact | NA |
| 5c: Robustness of measure | NA |
| **6: Economic assessment** |  |
| 6: Economic assessment | NA |
| **7: Study results** |  |
| 7a: Outcomes of PPI | 4 |
| 7b: Impacts of PPI | 4 |
| 7c: Context of PPI | 4 |
| 7d: Process of PPI | 4 |
| 7ei: Theory development | NA |
| 7eii: Theory development | NA |
| 7f: Measurement | NA |
| 7g: Economic assessment | NA |
| **8: Discussion and conclusions** |  |
| 8a: Outcomes | 11-13 |
| 8b: Impacts | 11-13 |
| 8c: Definition | NA |
| 8d: Theoretical underpinnings | 11-13 |
| 8e: Context | NA |
| 8f: Process | NA |
| 8g: Measurement and capture of PPI impact | NA |
| 8h: Economic assessment | NA |
| 8i: Reflections/critical perspective | 11-13 |

**Appendix 3 – Search Strategy**

Ovid MEDLINE(R)

1 (prehab* or pre-hab*).tw,kf.

2 (preoperative* rehabilitation or preoperative* training).tw,kw.

3 ((perioperat* or peri operat*) adj3 (exercis* or physiotherap* or physical therapy or diet or nutrition* or physical activit* or counsel?ing)).tw.

4 Preoperative Exercise/

5 ((exercise* or diet or nutrition* counsel?ing) and (surgery or surgical)).ti.

6 ((preoperat* or pre-operat* or presurg* or pre-surg*) adj3 (exercis* or physiotherap* or physical therapy or diet or nutrition* or physical activit* or counsel?ing or conditioning)).tw,kf.

7 ((preoperat* or pre-operat* or presurg* or pre-surg*) adj5 ((psychological or psychosocial or cognitive) adj3 (intervention* or therap*))).tw,kf.

8 ((preoperat* or pre-operat* or presurg* or pre-surg*) adj5 (diet or dietary or nutrit*)).tw,kf.

9 ((preoperat* or pre-operat* or presurg* or pre-surg*) adj5 (resistance or strength or weight or muscle) adj2 training).tw,kf.

10 ((preoperat* or pre-operat* or presurg* or pre-surg*) adj5 (food adj3 (counsel* or support* or modif*))).tw,kf.

11 ((preoperat* or pre-operat* or presurg* or pre-surg*) adj5 ((energy or protein) adj3 supplement*)).tw,kf.

12 ((preoperat* or pre-operat* or presurg* or pre-surg*) adj5 (exercise or exercises or stretch* or aerobic* or physical activit*)).tw,kf.

13 ((preoperat* or pre-operat* or presurg* or pre-surg*) adj5 (cbt or cognitive behavio?r*)).tw,kf.

14 ((preoperat* or pre-operat* or presurg* or pre-surg*) adj2 counsel*).tw,kf.

15 ((preoperat* or pre-operat* or presurg* or pre-surg*) adj2 supplement*).tw,kf.

16 or/1-15

17 *Preoperative Care/ or *Preoperative Period/ or (preoperat* or pre-operat* or presurg* or pre-surg*).ti.

18 exp Exercise Therapy/ or Exercise/

19 Physical Fitness/

20 (exercise or exercises or stretch* or aerobic* or physical activit*).tw,kw.

21 ((resistance or strength or weight or muscle) adj2 training).tw.

22 diet therapy/ or Nutrition Therapy/ or Nutritional Support/

23 (diet or dietary or nutrit*).tw,kw.

24 (food adj3 (counsel* or support* or modif*)).tw.

25 ((energy or protein) adj3 supplement*).tw.

26 Dietary Supplements/

27 Physical Therapy Modalities/ or (physical therap* or physiotherap*).tw,kw.

28 Counseling/ or counsel?ing.tw,kw.

29 behavior therapy/ or cognitive behavioral therapy/ or Psychotherapy/ or Relaxation Therapy/

30 Stress, Psychological/rh, th

31 ((psychological or psychosocial or cognitive) adj3 (intervention* or therap*)).tw.

32 or/18-31

33 17 and 32

34 16 or 33

35 randomized controlled trial.pt.

36 controlled clinical trial.pt.

37 random*.tw.

38 placebo.ab.

39 clinical trials as topic.sh.

40 trial.ti.

41 or/35-40

42 exp animals/ not humans/

43 (infant/ or child/ or adolescent/) not adult/

44 41 not (42 or 43)

45 34 and 44

46 45 use medall

Embase Classic+Embase

1 (prehab* or pre-hab*).mp.

2 (preoperative* rehabilitation or preoperative* training).tw.

3 ((perioperat* or peri operat*) adj3 (exercis* or physiotherap* or physical therapy or diet or nutrition* or physical activit* or counsel?ing)).tw.

4 ((preoperat* or pre-operat* or presurg* or pre-surg*) adj5 (exercis* or physiotherap* or physical therapy or diet or nutrition* or physical activit* or counsel?ing or conditioning)).tw.

5 ((preoperat* or pre-operat* or presurg* or pre-surg*) adj5 (diet or dietary or nutrit*)).tw.

6 ((preoperat* or pre-operat* or presurg* or pre-surg*) adj5 ((psychological or psychosocial or cognitive) adj3 (intervention* or therap*))).tw.

7 ((preoperat* or pre-operat* or presurg* or pre-surg*) adj5 (resistance or strength or weight or muscle) adj2 training).tw.

8 ((preoperat* or pre-operat* or presurg* or pre-surg*) adj5 (food adj3 (counsel* or support* or modif*))).tw.

9 ((preoperat* or pre-operat* or presurg* or pre-surg*) adj5 ((energy or protein) adj3 supplement*)).tw.

10 ((preoperat* or pre-operat* or presurg* or pre-surg*) adj5 (stretch* or aerobic*)).tw.

11 ((exercise* or diet or nutrition* counsel?ing) and (surgery or surgical)).ti.

12 ((preoperat* or pre-operat* or presurg* or pre-surg*) adj5 (cbt or cognitive behavio?r*)).tw.

13 ((preoperat* or pre-operat* or presurg* or pre-surg*) adj2 counsel*).tw.

14 ((preoperat* or pre-operat* or presurg* or pre-surg*) adj2 supplement*).tw.

15 or/1-14

16 *preoperative care/ or *preoperative period/ or (preoperat* or pre-operat* or presurg* or pre-surg*).ti.

17 exp *kinesiotherapy/

18 exp *exercise/

19 (exercise or exercises or stretch* or aerobic* or physical activit*).ti.

20 ((resistance or strength or weight or muscle) adj2 training).ti.

21 diet therapy/ or diet supplementation/

22 (diet or dietary or nutrit*).ti.

23 (food adj3 (counsel* or support* or modif*)).ti.

24 ((energy or protein) adj3 supplement*).tw.

25 physiotherapy/

26 (physical therap* or physiotherap*).ti.

27 counseling/ or nutritional counseling/

28 behavior therapy/

29 cognitive behavioral therapy/ or cognitive therapy/

30 psychotherapy/

31 ((psychological or psychosocial or cognitive) adj3 (intervention* or therap*)).ti.

32 or/17-31

33 16 and 32

34 15 or 33

35 double-blind*.mp. or placebo*.tw. or blind*.tw. or trial.ti.

36 random*.ti.

37 (random* adj2 (trial* or stud*)).tw.

38 35 or 36 or 37

39 34 and 38

40 (child/ or childhood/ or infant/) not adult/

41 (exp animal/ or nonhuman/) not exp human/

42 39 not (40 or 41)

EBM Reviews - Cochrane Central Register of Controlled Trials

1 (prehab* or pre-hab*).tw,kw.

2 (preoperative* rehabilitation or preoperative* training).tw,kw.

3 ((perioperat* or peri operat*) adj3 (exercis* or physiotherap* or physical therapy or diet or nutrition* or physical activit* or counsel?ing)).tw.

4 ((preoperat* or pre-operat* or presurg* or pre-surg*) adj2 (exercis* or physiotherap* or physical therapy or diet or nutrition* or physical activit* or counsel?ing or conditioning)).tw.

5 ((preoperat* or pre-operat* or presurg* or pre-surg*) adj4 (exercis* or physiotherap* or physical therapy or diet or nutrition* or physical activit* or counsel?ing or conditioning)).ti.

6 Preoperative Exercise/

7 ((exercise* or diet or nutrition* counsel?ing) and (surgery or surgical)).ti.

8 ((preoperat* or pre-operat* or presurg* or pre-surg*) adj5 ((psychological or psychosocial or cognitive) adj3 (intervention* or therap*))).tw.

9 ((preoperat* or pre-operat* or presurg* or pre-surg*) adj2 (diet or dietary or nutrit*)).tw.

10 ((preoperat* or pre-operat* or presurg* or pre-surg*) adj5 (resistance or strength or weight or muscle) adj2 training).tw.

11 ((preoperat* or pre-operat* or presurg* or pre-surg*) adj5 (food adj3 (counsel* or support* or modif*))).tw.

12 ((preoperat* or pre-operat* or presurg* or pre-surg*) adj5 ((energy or protein) adj3 supplement*)).tw.

13 ((preoperat* or pre-operat* or presurg* or pre-surg*) adj3 (exercise or exercises or stretch* or aerobic* or physical activit*)).tw.

14 ((preoperat* or pre-operat* or presurg* or pre-surg*) adj3 (cbt or cognitive behavio?r*)).tw.

15 ((preoperat* or pre-operat* or presurg* or pre-surg*) adj2 counsel*).tw.

16 ((preoperat* or pre-operat* or presurg* or pre-surg*) adj2 supplement*).tw.

17 or/1-16

18 (infant/ or child/ or adolescent/) not adult/

19 17 not 18

20 remove duplicates from 19

APA PsycInfo

1 (prehab* or pre-hab*).tw.

2 (preoperative* rehabilitation or preoperative* training).tw.

3 ((perioperat* or peri operat*) adj3 (exercis* or physiotherap* or physical therapy or diet or nutrition* or physical activit* or counsel?ing)).tw.

4 ((exercise* or diet or nutrition* counsel?ing) and (surgery or surgical)).ti.

5 1 or 2 or 3 or 4

6 surgical patients/ or (preoperat* or pre-operat* or presurg* or pre-surg*).ti.

7 exp exercise/ or physical activity/ or physical fitness/

8 (exercise or exercises or stretch* or aerobic* or physical activit*).tw.

9 ((resistance or strength or weight or muscle) adj2 training).tw.

10 Diets/

11 Nutrition/ or Dietary Supplements/

12 (diet or dietary or nutrit*).tw.

13 (food adj3 (counsel* or support* or modif*)).tw.

14 ((energy or protein) adj3 supplement*).tw.

15 physical therapy/ or physical treatment methods/

16 (physical therap* or physiotherap*).tw.

17 Counseling/

18 counsel?ing.tw.

19 Behavior Therapy/ or Cognitive Behavior Therapy/ or Cognitive Therapy/

20 Psychotherapy/

21 ((psychological or psychosocial or cognitive) adj3 (intervention* or therap*)).tw.

22 or/7-21

23 6 and 22

24 5 or 23

25 exp Clinical Trials/ or Placebo/ or (random* or sham or placebo* or ((singl* or doubl*) adj (blind* or dumm* or mask*)) or ((tripl* or trebl*) adj (blind* or dumm* or mask*)) or (control* adj3 (study or studies or trial* or group*)) or Nonrandom* or non random* or non-random* or quasi-random* or quasirandom* or allocated or ((open label or open-label) adj5 (study or studies or trial*)) or ((equivalence or superiority or non-inferiority or noninferiority) adj3 (study or studies or trial*)) or ((pragmatic or practical) adj3 trial*) or ((quasiexperimental or quasi-experimental) adj3 (study or studies or trial*)) or (phase adj3 (III or "3") adj3 (study or studies or trial*))).ti,ab,hw.

26 24 and 25

**CinahlComplete**

|  |
| --- |

| **#** | **Query** |  |
| --- | --- | --- |
| S1 | (MH "Prehabilitation") |  |
| S2 | TI ( (prehab* or pre-hab*) ) OR AB ( (prehab* or pre-hab*) ) |  |
| S3 | TI ( (preoperative* rehabilitation or preoperative* training) ) OR AB ( (preoperative* rehabilitation or preoperative* training) ) |  |
| S4 | TI ( ((perioperat* or peri operat*) N3 (exercis* or physiotherap* or physical therapy or diet or nutrition* or physical activit* or counsel?ing)) ) OR AB ( ((perioperat* or peri operat*) N3 (exercis* or physiotherap* or physical therapy or diet or nutrition* or physical activit* or counsel?ing)) ) |  |
| S5 | S1 OR S2 OR S3 OR S4 |  |
| S6 | (MH "Preoperative Care") or (MH "Preoperative Period") or TI (preoperat* or pre-operat* or presurg* or pre-surg) |  |
| S7 | (MH "Therapeutic Exercise+") |  |
| S8 | (MH "Exercise+") |  |
| S9 | (MH "Physical Fitness+") |  |
| S10 | TI ( (exercise or exercises or stretch* or aerobic* or physical activit*) ) OR AB ( (exercise or exercises or stretch* or aerobic* or physical activit*) ) |  |
| S11 | TI ( (resistance or strength or weight or muscle) N2 training ) OR AB ( (resistance or strength or weight or muscle) N2 training ) |  |
| S12 | (MH "Diet Therapy+") |  |
| S13 | (MH "Nutritional Support+") |  |
| S14 | TI ( (diet or dietary or nutrit*) ) OR AB ( (diet or dietary or nutrit*) ) |  |
| S15 | TI ( food N3 (counsel* or support* or modif*) ) OR AB ( food N3 (counsel* or support* or modif*) ) |  |
| S16 | TI ( (energy or protein) N3 supplement* ) OR AB ( (energy or protein) N3 supplement* ) |  |
| S17 | (MH "Dietary Supplementation") |  |
| S18 | (MH "Physical Therapy+") |  |
| S19 | (MH "Physical Therapy Service") |  |
| S20 | TI ( (physical therap* or physiotherap*) ) OR AB ( (physical therap* or physiotherap*) ) |  |
| S21 | (MH "Counseling+") |  |
| S22 | TI counsel?ing OR AB counsel?ing |  |
| S23 | (MH "Behavior Therapy") OR (MH "Cognitive Therapy") |  |
| S24 | (MH "Psychotherapy+") |  |
| S25 | TI ( (psychological or psychosocial or cognitive) N3 (intervention* or therap*) ) OR AB ( (psychological or psychosocial or cognitive) N3 (intervention* or therap*) ) |  |
| S26 | S7 OR S8 OR S9 OR S10 OR S11 OR S12 OR S13 OR S14 OR S15 OR S16 OR S17 OR S18 OR S19 OR S20 OR S21 OR S22 OR S23 OR S24 OR S25 |  |
| S27 | S6 AND S26 |  |
| S28 | TI ( (preoperat* or pre-operat* or presurg* or pre-surg*) N3 (exercis* or physiotherap* or physical therapy or diet or nutrition* or physical activit* or counsel?ing or conditioning) ) OR AB ( (preoperat* or pre-operat* or presurg* or pre-surg*) N3 (exercis* or physiotherap* or physical therapy or diet or nutrition* or physical activit* or counsel?ing or conditioning) ) |  |
| S29 | TI ( ((preoperat* or pre-operat* or presurg* or pre-surg*)N5 ((psychological or psychosocial or cognitive) N3 (intervention* or therap*)) ) OR AB ( ((preoperat* or pre-operat* or presurg* or pre-surg*)N5 ((psychological or psychosocial or cognitive) N3 (intervention* or therap*)) ) |  |
| S30 | TI ( ((preoperat* or pre-operat* or presurg* or pre-surg*) N5 (diet or dietary or nutrit*)) ) OR AB ( ((preoperat* or pre-operat* or presurg* or pre-surg*) N5 (diet or dietary or nutrit*)) ) |  |
| S31 | TI ( ((preoperat* or pre-operat* or presurg* or pre-surg*) N5 (resistance or strength or weight or muscle) N2 training) ) OR AB ( ((preoperat* or pre-operat* or presurg* or pre-surg*) N5 (resistance or strength or weight or muscle) N2 training) ) |  |
| S32 | TI ( ((preoperat* or pre-operat* or presurg* or pre-surg*) N5 ((energy or protein) N3 supplement*)) ) OR AB ( ((preoperat* or pre-operat* or presurg* or pre-surg*) N5 ((energy or protein) N3 supplement*)) ) |  |
| S33 | TI ( ((preoperat* or pre-operat* or presurg* or pre-surg*) N5 (exercise or exercises or stretch* or aerobic* or physical activit*)) ) OR AB ( ((preoperat* or pre-operat* or presurg* or pre-surg*) N5 (exercise or exercises or stretch* or aerobic* or physical activit*)) ) |  |
| S34 | TI ( (preoperat* or pre-operat* or presurg* or pre-surg*) N5 (cbt or cognitive behavior* or cognitive behaviour)) ) OR AB ( (preoperat* or pre-operat* or presurg* or pre-surg*) N5 (cbt or cognitive behavior* or cognitive behaviour)) ) |  |
| S35 | TI ( ((preoperat* or pre-operat* or presurg* or pre-surg*) N2 counsel*) ) OR AB ( ((preoperat* or pre-operat* or presurg* or pre-surg*) N2 counsel*) ) |  |
| S36 | TI ( ((preoperat* or pre-operat* or presurg* or pre-surg*) N2 supplement ) OR AB ( ((preoperat* or pre-operat* or presurg* or pre-surg*) N2 supplement ) |  |
| S37 | S5 OR S27 OR S28 OR S29 OR S30 OR S31 OR S32 OR S33 OR S34 OR S35 OR S36 |  |
| S38 | ( MH ( randomized controlled trials OR double‐blind studies OR single‐blind studies OR random assignment OR pretest‐posttest design OR cluster sample ) OR TI ( randomised OR randomized ) OR AB random* OR TI trial OR ( (MH (sample size) AND AB (assigned OR allocated OR control)) ) OR MH ( placebos OR crossover design OR comparative studies ) OR AB ( (control W5 group) OR (cluster W3 RCT) OR PT (randomized controlled trial)) ) NOT ( ( MH animals+ OR MH (animal studies) OR TI (animal model*) ) NOT MH (human) ) |  |
| S39 | S37 AND S38 |  |
| S40 | (MH "Child+") NOT (MH "Adult+") |  |
| S41 | S39 NOT S40 |  |

Web of Science

(infant/ or child/ or adolescent/) not adult/

((preoperat* or pre-operat* or presurg* or pre-surg*) N5 (cbt or cognitive behavior* or cognitive behaviour)).

1. (ALL=(prehab*)) OR ALL=("pre habilitation")

2. (TI=((preoperat* OR pre-operat* OR presurg* OR pre-surg*) )) AND TI=(exercis* or physiotherap* or physical therapy or diet or nutrition* or physical activit* or counsel* OR condition*)

3. (TI=((preoperat* OR pre-operat* OR presurg* OR pre-surg*))) AND TI=(counsel* OR psych* OR behavior therap* OR behaviour therap*)

4. (TI=(preoperat* OR pre-operat* OR presurg* OR pre-surg*)) AND TI=(nutrition OR diet OR supplement OR dietary)

5. (TI=(exercise* or diet or nutrition* counsel*)) AND TI=(surgery or surgical)

6. ((((#1) OR #2) OR #3) OR #4) OR #5

7. Search

(TS=(randomised OR randomized OR randomisation OR randomisation OR placebo* OR (random* AND (allocat* OR assign*) ) OR (blind* AND (single OR double OR treble OR triple) )) NOT TS=(animal or animals or pisces or fish or fishes or catfish or catfishes or sheatfish or silurus or arius or heteropneustes or clarias or gariepinus or fathead minnow or fathead minnows or pimephales or promelas or cichlidae or trout or trouts or char or chars or salvelinus or salmo or oncorhynchus or guppy or guppies or millionfish or poecilia or goldfish or goldfishes or carassius or auratus or mullet or mullets or mugil or curema or shark or sharks or cod or cods or gadus or morhua or carp or carps or cyprinus or carpio or killifish or eel or eels or anguilla or zander or sander or lucioperca or stizostedion or turbot or turbots or psetta or flatfish or flatfishes or plaice or pleuronectes or platessa or tilapia or tilapias or oreochromis or sarotherodon or common sole or dover sole or solea or zebrafish or zebrafishes or danio or rerio or seabass or dicentrarchus or labrax or morone or lamprey or lampreys or petromyzon or pumpkinseed or pumpkinseeds or lepomis or gibbosus or herring or clupea or harengus or amphibia or amphibian or amphibians or anura or salientia or frog or frogs or rana or toad or toads or bufo or xenopus or laevis or bombina or epidalea or calamita or salamander or salamanders or newt or newts or triturus or reptilia or reptile or reptiles or bearded dragon or pogona or vitticeps or iguana or iguanas or lizard or lizards or anguis fragilis or turtle or turtles or snakes or snake or aves or bird or birds or quail or quails or coturnix or bobwhite or colinus or virginianus or poultry or poultries or fowl or fowls or chicken or chickens or gallus or zebra finch or taeniopygia or guttata or canary or canaries or serinus or canaria or parakeet or parakeets or grasskeet or parrot or parrots or psittacine or psittacines or shelduck or tadorna or goose or geese or branta or leucopsis or woodlark or lullula or flycatcher or ficedula or hypoleuca or dove or doves or geopelia or cuneata or duck or ducks or greylag or graylag or anser or harrier or circus pygargus or red knot or great knot or calidris or canutus or godwit or limosa or lapponica or meleagris or gallopavo or jackdaw or corvus or monedula or ruff or philomachus or pugnax or lapwing or peewit or plover or vanellus or swan or cygnus or columbianus or bewickii or gull or chroicocephalus or ridibundus or albifrons or great tit or parus or aythya or fuligula or streptopelia or risoria or spoonbill or platalea or leucorodia or blackbird or turdus or merula or blue tit or cyanistes or pigeon or pigeons or columba or pintail or anas or starling or sturnus or owl or athene noctua or pochard or ferina or cockatiel or nymphicus or hollandicus or skylark or alauda or tern or sterna or teal or crecca or oystercatcher or haematopus or ostralegus or shrew or shrews or sorex or araneus or crocidura or russula or european mole or talpa or chiroptera or bat or bats or eptesicus or serotinus or myotis or dasycneme or daubentonii or pipistrelle or pipistrellus or cat or cats or felis or catus or feline or dog or dogs or canis or canine or canines or otter or otters or lutra or badger or badgers or meles or fitchew or fitch or foumart or foulmart or ferrets or ferret or polecat or polecats or mustela or putorius or weasel or weasels or fox or foxes or vulpes or common seal or phoca or vitulina or grey seal or halichoerus or horse or horses or equus or equine or equidae or donkey or donkeys or mule or mules or pig or pigs or swine or swines or hog or hogs or boar or boars or porcine or piglet or piglets or sus or scrofa or llama or llamas or lama or glama or deer or deers or cervus or elaphus or cow or cows or bos taurus or bos indicus or bovine or bull or bulls or cattle or bison or bisons or sheep or sheeps or ovis aries or ovine or lamb or lambs or mouflon or mouflons or goat or goats or capra or caprine or chamois or rupicapra or leporidae or lagomorpha or lagomorph or rabbit or rabbits or oryctolagus or cuniculus or laprine or hares or lepus or rodentia or rodent or rodents or murinae or mouse or mice or mus or musculus or murine or woodmouse or apodemus or rat or rats or rattus or norvegicus or guinea pig or guinea pigs or cavia or porcellus or hamster or hamsters or mesocricetus or cricetulus or cricetus or gerbil or gerbils or jird or jirds or meriones or unguiculatus or jerboa or jerboas or jaculus or chinchilla or chinchillas or beaver or beavers or castor fiber or castor canadensis or sciuridae or squirrel or squirrels or sciurus or chipmunk or chipmunks or marmot or marmots or marmota or suslik or susliks or spermophilus or cynomys or cottonrat or cottonrats or sigmodon or vole or voles or microtus or myodes or glareolus or primate or primates or prosimian or prosimians or lemur or lemurs or lemuridae or loris or bush baby or bush babies or bushbaby or bushbabies or galago or galagos or anthropoidea or anthropoids or simian or simians or monkey or monkeys or marmoset or marmosets or callithrix or cebuella or tamarin or tamarins or saguinus or leontopithecus or squirrel monkey or squirrel monkeys or saimiri or night monkey or night monkeys or owl monkey or owl monkeys or douroucoulis or aotus or spider monkey or spider monkeys or ateles or baboon or baboons or papio or rhesus monkey or macaque or macaca or mulatta or cynomolgus or fascicularis or green monkey or green monkeys or chlorocebus or vervet or vervets or pygerythrus or hominoidea or ape or apes or hylobatidae or gibbon or gibbons or siamang or siamangs or nomascus or symphalangus or hominidae or orangutan or orangutans or pongo or chimpanzee or chimpanzees or pan troglodytes or bonobo or bonobos or pan paniscus or gorilla or gorillas or troglodytes))

8. (#6) AND #7

859 results from Science Citation Index Expanded (SCI-EXPANDED), Conference Proceedings Citation Index – Science (CPCI-S), Conference Proceedings Citation Index – Social Science & Humanities (CPCI-SSH), Emerging Sources Citation Index (ESCI)

**Appendix 4 – Data extraction**

1. Author

2. Year of publication

3. Countries of origin

4. Study start and end dates

5. Prehabilitation characteristics

a. Multimodal intervention

6. Prehabilitation components (exercise, nutrition, psychosocial, cognitive)

a. Type of exercise

i. Unimodal

ii. Multimodal

iii. Interval training

iv. Other

b. Exercise components

i. Cardio

ii. Strength

iii. Stretching

iv. Respiratory exercise

c. Type of nutrition

i. Counselling

ii. Supplementation

iii. Combined

iv. Other

d. Type of psychosocial intervention*

i. Motivational techniques

ii. Anxiety management

iii. Stress management

iv. Other

e. Type of cognitive

6. Concurrent use of Enhanced Recovery After Surgery (ERAS)

7. Duration of prehabilitation

a. Session (in weeks)

b. Program

i. Minimum participation

ii. Maximum participation

c. Per session (min)

8. Frequency of prehabilitation per week

9. Intervention time-point

a. Preoperative only

b. Preoperative plus postoperative

10. Location of prehabilitation

a. Home

b. Facility

c. Combined

11. Supervision

a. Self-directed

b. Coach-led

c. Combined

12. Session format

a. Individual

b. Group

c. Combined

13. Personalisation †

14. Surgical specialty

a. Orthopaedic

b. Major non-oncology

c. Cardiac/vascular

d. Oncology

e. Mixed

f. Other

15.Specific surgical procedure

16. Operative Stress Score (OSS)

17. Population characteristics

a. Total sample size

b. Sample size per arm

c. Age

d. Sex

e. Presence of specific risk factors

i. Multimorbidity (>2 comorbidities)

ii. ASA score

iii. Frailty status

iv. Malnutrition

v. Presence of cancer

*Motivational techniques, anxiety and/or stress management, cognitive behavioural therapy, education in pain and the interaction between cognition and pain perception; training in cognitive and behavioural pain coping skills; and training in how to apply the learned coping skills in real-life situations.

† Exercise was personalised if training weights or intensity (speed and power) were based on the patient's ability and maximum heart rate or inspiratory pressure. Nutrition was personalised if energy consumption (Kcal) and/or daily protein intake were based on the patient's weight or nutritional status. Cognitive and psychosocial personalization could not consist solely of a single point-in-time intervention and ongoing action support tailored to the participant's needs was required.

**Appendix 5 – Protocol deviations**

1. Based on the reporting from some studies, we interpreted “attendance” and “completed” as adherence.
2. Some studies were two-, three- and up to four-arm trials. We only included arms that reported adherence for the respective arm. We approached each arm, whether binary or continuous and analysed it within the reported adherence prehabilitation component.
3. ASA score, multimorbidity, frailty status and malnutrition or risk of malnutrition could not be included in the meta-regression due to lack of data.

**Appendix 6. Excluded randomised controlled trials**

| Citation | Reason for exclusion: |
| --- | --- |
| Nirali, M., Srivastava, S.. Added effect of deep breathing and diaphragmatic breathing exercise in upper abdominal surgery patients: A randomised clinical trial. Indian Journal of Public Health Research and Development. 2020. 11:544-549 | Wrong or no Outcome |
| Docherty, L., Quinn, J. A., Edwards, J., Moug, S. J., Park, J. H.. Does prehabilitation affect immune checkpoint expression in patients with locally advanced rectal cancer?. British Journal of Surgery. 2020. 107:131 | Wrong or no Outcome |
| Zhao, K., Guo, M., Hinkley, M., Luu, K., Javer, A. R., Thamboo, A.. The effect of online education videos on patient experience in endoscopic sinus surgery for chronic rhinosinusitis. Otolaryngology - Head and Neck Surgery. 2020. 163:P136-P137 | Wrong or Unclear Duration |
| Hanley, A., Gililland, J., Erickson, J., Rojas, J., Garland, E.. A preoperative mind-body intervention improves total joint arthroplasty patients' postoperative physical function: Primary outcomes and mechanistic explorations from a randomized controlled trial. Global Advances in Health and Medicine. 2020. 9:21-22 | Wrong or Unclear Duration |
| Shukla, A., Granger, C., Edbrook, L., Wright, G., Denehy, L.. Prehabilitation for Individuals Having Lung Cancer Surgery: Feasibility and Acceptability of A Pre-Operative Exercise Intervention. Respirology. 2020. 25:59 | Wrong or no Outcome |
| Wang, Y. Q., Cao, H. P., Liu, X., Yang, Z., Yin, Y. Y., Ma, R. C., Xie, J.. Effect of breathing exercises in patients with non-small cell lung cancer receiving surgical treatment: A randomized controlled trial. European Journal of Integrative Medicine. 2020. 38:101175 | Wrong or Unclear Duration |
| Waller, E., Rahman, S., Sutton, P., Allen, J., Saxton, J., Aziz, O.. Randomised controlled trial of patients undergoing prehabilitation with wearables versus standard of care before major abdominal cancer surgery (Trial Registration: NCT04047524). Colorectal Disease. 2020. 22:7 | Wrong or no Outcome |
| Kate, V., Kundra, P., Swaminathan, N.. ENHANCED RECOVERY AFTER SURGERY WITH RESPIRATORY PREHABILITATION VERSUS CONVENTIONAL PERIOPERATIVE PROTOCOL IN PATIENTS UNDERGOING ELECTIVE GASTRECTOMY- A RANDOMIZED CONTROLLED TRIAL. Gastroenterology. 2020. 158:S-1558 | Unregistered Abstract |
| Berger-Richardson, D., Alavi, N., Trudeau, M., Lemon-Wong, S., Look-Hong, N., Mascarenhas, J., Gibson, L., Wisdom-Gilliam, P., Isenberg-Grzeda, E., Santa-Mina, D., Wright, F.. A randomized controlled feasibility study comparing a multimodal prehabilitation protocol to normal care for women undergoing neo-adjuvant chemotherapy for breast cancer. Annals of Surgical Oncology. 2020. 27:S162 | Wrong or no Outcome |
| Lee, I. K., Kye, B. H., Lee, J. H., Park, I. J., Oh, H. K., Cho, Y. B., Kim, Y. T., Kim, J. Y., Lee, J. L., Sim, J. H.. THE EFFECTS OF PROBIOTICS ON SYMPTOM AND SURGICAL OUTCOME AFTER ANTERIOR RESECTION OF COLON CANCER; MULTICENTER, DOUBLE-BLIND, RANDOMIZED, PLACEBO-CONTROLLED TRIAL (POSTCARE STUDY). Gastroenterology. 2019. 156:S-1423 | Single Risk Factor Management |
| Duchalais, E.. Impact of pelvic floor prehabilitation using biofeedback therapy on the severity of low anterior resection syndrome following total mesorectal excision: CONTICARE randomized controlled trial. Colorectal Disease. 2019. 21:131 | No Results |
| Allen, S., Brown, V., White, D., King, D., Hunt, J., Prabhu, P., Rockall, T., Preston, S., Sultan, J.. Multi-modal prehabilitation during neoadjuvant therapy prior to resection for oesophagogastric cancer: A pilot randomised controlled trial. British Journal of Surgery. 2019. 106:90-91 | Duplicate |
| Mak, T., Futaba, K., Leung, W. W., Ma, B., Lau, V., Ng, S.. Multidisciplinary prehabilitation programme on patients undergoing colorectal cancer surgery: A single centre randomised controlled trial. Colorectal Disease. 2019. 21:16 | Wrong or Unclear Duration |
| Danzi, O. P., Marinelli, V., Tuveri, M., Salvia, R., Secchettin, E., Bonamini, D., Mazzi, M. A., Rimondini, M., Bassi, C., Del Piccolo, L.. PREPARE: PREoPerative Anxiety REduction. Pre-operative psychological intervention to increase patients' self-efficacy in managing anxiety before major pancreatic surgery: Preliminary results of a randomized clinical trial. Psychotherapy and Psychosomatics. 2019. 88:33 | Wrong or Unclear Duration |
| Wierdak, M., Surmiak, M., Rubinkiewicz, M., Wysocki, M., Milian-Ciesielska, K., Major, P., Rzepa, A., Pedziwiatr, M.. PREOPERATIVE IMMUNONUTRITION COMPARED TO STANDARD NUTRITIONAL SUPPORT AFFECTS THE INFLAMMATORY RESPONSE WITHIN THE COLORECTAL CANCER TISSUE - RANDOMIZED CONTROLLED TRIAL. Clinical Nutrition. 2019. 38:S56-S57 | Wrong or no Outcome |
| Gonzalez Valverde, F. M., Tamayo Rodriguez, M. E., Del Valle Ruiz, S. R., Medina Manuel, E., Fernandez Lopez, A. J., Gimenez Frances, C., Lopez Morales, P.. Outcome of preoperative surveillance before bariatric surgery. Obesity Surgery. 2019. 29:985 | Wrong or Unclear Duration |
| Sayner, A.. Functional pelvic floor muscle training before radical prostatectomy: A prospective randomised controlled pilot study. Neurourology and Urodynamics. 2019. 38:S348-S350 | Unregistered Abstract |
| Sifuentes, A. M. M., Flores, D. S., Villegas, L. H.. Effect of pre-habilitation on quality of life and postoperative fatigue syndrome in Medico Nacional- Leon, IMSS de Leon, Guanajuato. Revista Hispanoamericana de Hernia. 2018. 6:11-16 | Duplicate |
| Kanekiyo, S., Takeda, S., Iida, M., Nishiyama, M., Kitahara, M., Tokumitsu, Y., Tomochika, S., Suzuki, N., Yoshino, S., Hazama, S., Nagano, H.. Efficacy of perioperative immunonutrition of esophagectomy for esophageal cancer. Diseases of the Esophagus. 2018. 31:170 | Unregistered Abstract |
| Allen, S., White, D., Rockall, T., Preston, S., Sultan, J.. The effect of prehabilitation on sarcopenia development during neoadjuvant chemotherapy for oesophagogastric cancer: A randomised controlled trial. Diseases of the Esophagus. 2018. 31:191 | Unregistered Abstract |
| Allen, S., Brown, V., Prabhu, P., Rockall, T., Preston, S., Sultan, J.. Effect of prehabilitation on fitness in patients undergoing neoadjuvant treatment and oesophagogastric cancer surgery: A randomised controlled trial. Diseases of the Esophagus. 2018. 31:172 | Unregistered Abstract |
| Greene, K. A., Tanner, J. P., Wyman, A. M., Bassaly, R. M.. Factors associated with postoperative patient preparedness. Female Pelvic Medicine and Reconstructive Surgery. 2018. 24:S116 | Not a Randomized Trial |
| Minnella, E. M., Awasthi, R., Loiselle, S. E., Ramanakuma, A., Ferri, L., Carli, F.. Prehabilitation improves functional capacity in esophago-gastric cancer surgery: A randomized control trial. Canadian Journal of Anesthesia. 2018. 65:S91-S92 | Duplicate |
| Ausania, F., Melendez, R., Senra, P., Palmeiro, R., Oubina, R., Otero, I.. Prehabilitation in patients undergoing pancreaticoduodenectomy: a randomized controlled trial. HPB. 2018. 20:S627-S628 | Duplicate |
| Hile, E., Neuhold, R., Davidson, V.. Training for the fight: Adherence to a novel prehab approach in pancreaticoduodenectomy. Rehabilitation Oncology. 2018. 36:E8 | Wrong or no Outcome |
| Erdem, N. Z., Ozelgun, D., Taskin, H. E., Avsar, F. M., Taskin, M.. The effects of protein-riched diet, performed before bariatric surgery, on losing weight, clinical results and liver volume reduction. Obesity Surgery. 2018. 28:S61 | Wrong or no Outcome |
| Kassouf, W., Minnella, E., Awasthi, R., Ferreira, V., Aprikian, A., Tanguay, S., Carli, F.. Prehabilitation for patients undergoing cystectomy: Preliminary analysis of a single-center, randomized controlled trial. Journal of Urology. 2018. 199:e622 | Wrong or no Outcome |
| Heiman Ullmark, J., Bock, D., Fagevik Olsen, M., Olofsson Bagge, R., Haglind, E.. PhysSurg-B-PHYSical activity in relation to SURGical operations. European Journal of Cancer. 2018. 92:S77 | No Results |
| Barberan-Garcia, A., Ubre, M., Roca, J., Lacy, A. M., Burgos, F., Risco, R., Momblan, D., Balust, J., Blanco, I., Martinez-Palli, G.. Personalised prehabilitation in high-risk patients undergoing elective major abdominal surgery: A randomised controlled trial. European Respiratory Journal. 2017. 50: | Duplicate |
| Shah, R., Shah, S., Sawant, A., Gangwani, J., Khamkar, A., Thakker, F., Shah, P.. Can pharmacotherapy be superior to diet for preoperative bariatric surgery preparation? Pre-operative management. Obesity Surgery. 2017. 27:137 | Not Prehabilitation |
| Elrefai, M.. Value of low calorie diet before sleeve gastrectomy: Prospectieve randomised study. pre-operative management. Obesity Surgery. 2017. 27:813 | Wrong or no Outcome |
| Thomas, G., Van Rooijen, S. J., Roumen, R., Schep, G., Slooter, G.. Feasibility of a multimodal prehabilitation program for patients undergoing surgery for colorectal cancer. South African Gastroenterology Review. 2017. 15:5-6 | Wrong or Unclear Duration |
| Tenconi, S., Galeone, C., Fugazzaro, S., Rapicetta, C., Piro, R., Formisano, D.. Perioperative and long-term effects of comprehensive pulmonary rehabilitation on exercise capacity, postoperative outcome and quality of life in patients undergoing lung resection: A randomized controlled trial granted by the ministry of health. Interactive Cardiovascular and Thoracic Surgery. 2017. 25: | No Results |
| Ilyas, A., Alter, T.. A prospective randomized study analyzing the effect of pre-operative opioid counseling on post-operative opioid consumption after hand surgery. Journal of Hand Surgery. 2017. 42:S20 | Not Prehabilitation |
| Onerup, A., Thorn, S. E., Angenete, E., Bock, D., Gryback-Gillheimer, E., Haglind, E., Nilsson, H.. The effect of pre-and postoperative physical activity on recovery after colorectal cancer surgery (PHYSSURG-C): A randomized controlled trial. Colorectal Disease. 2017. 19:141 | Protocol |
| Thomas, G., Van Rooijen, S., Roumen, R., Schep, G., Slooter, G.. Feasibility of a prehabilitation program for patients undergoing surgery for colorectal cancer. Colorectal Disease. 2017. 19:74 | Duplicate |
| Jensen, B. T., Sondergaard, I., Kiesbye, B., Jensen, J. B., Kristensen, S. A.. Efficacy of preoperative uro-stoma-education on self-efficacy after radical cystectomy; Secondary outcome from a prospective randomized controlled trial. Scandinavian Journal of Urology. 2017. 51:64 | Not Prehabilitation |
| Hile, E., Hoffman, L., Postier, R., Ding, K., Yang, J., Li, M.. A pilot RCT of sarcopenia-focused prehabilitation in pancreas cancer. Journal of Clinical Oncology. 2017. 35: | Not a Randomized Trial |
| Bond, D. S., Graham Thomas, J., Vithiananthan, S., Webster, J., Unick, J., Ryder, B. A., Pohl, D.. Changes in enjoyment, self-efficacy, and motivation during a randomized trial to promote habitual physical activity adoption in bariatric surgery patients. Surgery for Obesity and Related Diseases. 2016. 12:1072-1079 | Wrong or no Outcome |
| Hernon, J.. SupPoRtive Exercise Programmes for Accelerating REcovery after major ABdominal Cancer surgery (PREPARE-ABC). Colorectal Disease. 2016. 18:126 | Duplicate |
| Burden, S., Gibson, D. J., Lal, S., Hill, J., Pilling, M., Soop, M., Ramesh, A., Todd, C.. A single blinded randomised controlled trial of preoperative oral supplements in weight losing patients with colorectal cancer. Clinical Nutrition. 2016. 35:S17 | Wrong or Unclear Duration |
| Karenovics, W., Licker, M., Christodoulou, M., Diaper, J., Bhatia, C., Bridevaux, P., Triponez, F.. Does short-term preoperative exercise therapy influence longterm lung functional outcome following lung cancer surgery?. Interactive Cardiovascular and Thoracic Surgery. 2016. 23: | Wrong or Unclear Duration |
| Salzmann, S., Euteneuer, F., Auer, C. J., Laferton, J. A., Schedlowski, M., Moosdorf, R., Rief, W.. Pre-surgical psychological interventions lead to reduced levels of adrenaline and cortisol after surgery in coronary artery bypass graft patients: The PSY-heart study. Psychosomatic Medicine. 2016. 78:A29 | Wrong or Unclear Duration |
| Sockalingam, S., Cassin, S., Du, C., Wnuk, S., Hawa, R., Jackson, T., Parikh, S.. A pilot randomized controlled trial of telephone-based cognitive behavioral therapy for preoperative bariatric surgery patients. Surgery for Obesity and Related Diseases. 2015. 11:S53-S54 | Wrong or no Outcome |
| Adib, H., Kaviani, S., Dadgostar, H.. Beneficial effects of home-based and supervised exercise programs on anthropometric indices, body composition, and aerobic capacity in obese patients who undergoing laparoscopic gastric bypass surgery. Journal of Science and Medicine in Sport. 2015. 19:e41 | Wrong or no Outcome |
| Jensen, B. T., Jensen, J. B., Borre, M., Laustsen, S., Petersen, A. K.. Physical prehabilitation is feasible and effective in patients with advanced bladder cancer. Cancer Nursing. 2015. 38:S5 | Wrong or no Outcome |
| Jones, A., Alvadj-Korenic, T., Mayan, M., Beaupre, L., Hawker, G.. Delivering preoperative rehabilitation exercise program to patients with severe functional limitations awaiting total knee arthoplasty: Experiences of patients and physical therapists. Journal of Rheumatology. 2015. 42:1270 | Not a Randomized Trial |
| Yasueda, A., Miyazaki, S., Matsuda, C., Mizushima, T., Nishimura, J., Danno, K., Fujitani, K., Iwase, K., Ito, T.. A randomized controlled trial regarding perioperative management using formulation (PN-2) contained collagen peptides for patients with colorectal cancer: A preliminary report. Clinical Nutrition. 2015. 34:S224 | Wrong or no Outcome |
| Valtonen, A., Poyhonen, T., Manninen, M., Heinonen, A., Sipila, S.. Effects of preoperative aquatic resistance training on knee pain, mobility limitation and muscle impairments in people with late-stage knee osteoarthritis. Physiotherapy (United Kingdom). 2015. 101:eS1568-eS1569 | Wrong or no Outcome |
| Efficacy of action observation pre-operative training in functional recovery after hip and knee prosthesis. 2015. | Not Prehabilitation |
| Oosting, E., Hoogeboom, T., Appelman, S., Dronkers, J., Van Meeteren, N.. Feasibility of an intensive therapeutic exercise program for frail elderly prior to total hip arthroplasty: Two randomized pilot studies. Physiotherapy (United Kingdom). 2015. 101:eS1150-eS1151 | Not a Randomized Trial |
| Karihtala, T., Heinonen, A., Manninen, M., Poyhonen, T., Sipila, S., Valtonen, A.. Effects of preoperative group-based aquatic training on health related quality of life in persons with late stage knee osteoarthritis. Physiotherapy (United Kingdom). 2015. 101:eS723 | Wrong or no Outcome |
| Louw, A., Diener, I.. Preoperative neuroscience education for lumbar radiculopathy patients-a randomised control trial. Physiotherapy (United Kingdom). 2015. 101:eS317 | Wrong or Unclear Duration |
| Rolving, N., Soegaard, R., Nielsen, C. V., Christensen, F. B., Bunger, C. E., Oestergaard, L. G.. Preoperative cognitive-behavioural patient education versus standard care after lumbar spinal fusion: Economic evaluation alongside a randomized controlled trial. European Spine Journal. 2015. 24:S692 | Wrong or Unclear Duration |
| Banerjee, S., Manley, K., Shaw, B., Kumar, V., Ho, E. T. S., Rochester, M., Mills, R., Saxton, J.. 'Prehabilitation' of patients undergoing radical cystectomy to assist recovery: Results of a feasibility study. European Urology, Supplements. 2015. 14:e444 | Wrong or no Outcome |
| Dunne, D., Jones, R., Lythgoe, D., Malik, H., Poston, G. J., Jack, S., Palmer, D. H., Fenwick, S. W.. Prehabilitation before liver surgery. European Journal of Surgical Oncology. 2014. 40:S52 | Wrong or no Outcome |
| Moriya, T., Fukatsu, K., Okamoto, K., Shinto, E., Ueno, H., Hase, K., Yamamoto, J.. Effects of preoperative use of an immune-enhancing diet on postoperative complications and long-term outcome: A randomized clinical trial in colorectal cancer surgery in Japanese patients. Clinical Nutrition. 2014. 33:S247 | Wrong or Unclear Duration |
| Jarosz, A., Szlubowski, A., Grochowski, Z., Janczura, M., Ladynska, M., Pominkiewicz, L., Gnass, M., Cmiel, A., Soja, J., Kuzdzal, J.. The evaluation of utility of preoperative systematized pulmonological physiotherapy among non-small-cell lung cancer patients undergoing anatomical lung resection. Interactive Cardiovascular and Thoracic Surgery. 2014. 18:S32 | Wrong or no Outcome |
| Richardson, K., Sanders, G., Hayden, P., Marcora, S., Hopker, J.. The effect of preoperative exercise on postoperative outcome in abdominal aortic aneurysm (AAA) patients: Pilot study. Intensive Care Medicine. 2014. 40:S136 | Wrong or Unclear Duration |
| Pournaras, D. J., Arner, P., Hagstrom-Toft, E., Le Roux, C. W., Thorell, A.. Improvement in insulin resistance after gastric bypass; is it the bypass of the gut or the calorie restriction? Lessons from a randomised control trial. Obesity Surgery. 2014. 24:1003-1004 | Wrong or no Outcome |
| Baillot, A., Mampuya, W. M., Dionne, I. J., Comeau, E. M., Meziat-Burdin, A., Langlois, M.. Adding supervised group exercise training to interdisciplinary lifestyle management in subjects awaiting bariatric surgery: A randomized controlled study. Obesity Surgery. 2014. 24:1357 | Wrong or no Outcome |
| Liljensoe, A., Laursen, J., Bliddal, H., Soballe, K., Mechlenburg, I.. Weight loss intervention before total knee replacement. A safety study. Obesity Reviews. 2014. 15:154 | Wrong or no Outcome |
| Bottin, J., Balogun, B., Thomas, E., Fitzpatrick, J., Moorthy, K., Leeds, A., Bell, J., Frost, G.. Changes in body composition induced by pre-operative liquid low-calorie diet in morbid obese patients undergoing Roux-en-Y gastric bypass. Obesity Reviews. 2014. 15:129-130 | Wrong or no Outcome |
| Jensen, B., Jensen, J. B., Petsersen, A. K., Laustsen, S., Sondergaard, I., Borre, M.. Early rehabilitation can impact on health-related quality of life outcome in radical cystectomy: A randomised controlled trial. Supportive Care in Cancer. 2014. 22:S185 | Wrong or Unclear Duration |
| Schutz, T., Peter, V., Garnov, N., Schaudinn, A., Linder, N., Busse, H., Edward, S., Petroff, D., Dietrich, A.. Effect of two preoperative low energy diets on liver volume in bariatric patients: A randomized trial. Obesity Facts. 2014. 7:83 | Wrong or no Outcome |
| Hermann, A., Holsgaard-Larsen, A., Zerahn, B., Mejdahl, S., Overgaard, S.. Preoperative effects of progressive explosive-type resistance training in patients with osteoarthritis scheduled for total hip arthroplasty-a prospective randomized clinical trial. Osteoarthritis and Cartilage. 2014. 22:S454-S455 | Wrong or no Outcome |
| Burr, N., Burnand, K., Lahiri, R., Bennett, J. M., Lewis, M. P.. A randomised, single-blinded trial assessing the effect of a two week preoperative very low calorie diet on laparoscopic cholecystectomy procedure in obese patients. Gastroenterology. 2014. 146:S-1024 | Wrong or no Outcome |
| Hermann, A., Holsgaard-Larsen, A., Mejdahl, S., Zerahn, B., Overgaard, S.. Preoperative resistance training increases muscle function in patients diagnosed with hip osteoarthritis scheduled for total hip arthroplasty A- a randomized explorative trial. Osteoarthritis and Cartilage. 2013. 21:S149-S150 | Wrong or no Outcome |
| Al-Najjar, M. M. H., Rajab, M. F., Abukhudair, W., Sirajuddin, S. A., Eman, K.. Proper method for pre-operative chest preparation of patients listed for cardiac surgery. Intensive Care Medicine. 2013. 39:S415 | Not Prehabilitation |
| Collado Serra, A., Pellicer Cabo, M., Ramirez Backhaus, M., Dominguez-Escrig, J., Rubio-Briones, J., Gomez-Ferrer, A., Iborra Juan, I., Casanova Ramon-Borja, J., Ricos Torrent, J. V., Monros Lliso, J. L., Dumont Martinez, R., Solsona Narbon, E.. Intensive preoperatory Pelvic Floor Muscle Training reduce duration and severity of stress urinary incontinence after radical prostatectomy: A randomized controlled trial. European Urology, Supplements. 2013. 12:e1007-e1008 | Wrong or no Outcome |
| Gade, H. G., Rosenvinge, J. H. R., Friborg, O. F., Hjelmesaeth, J. H.. Cognitive behavioural therapy significantly reduces emotional eating in bariatric surgery patients. A randomised controlled trial. Obesity Facts. 2013. 6:30-31 | Wrong or no Outcome |
| Banerjee, S., Manley, K., Thomas, L., Shaw, B., Saxton, J., Mills, R., Rochester, M.. Preoperative exercise protocol to aid recovery of radical cystectomy: Results of a feasibility study. European Urology, Supplements. 2013. 12:125-126 | Wrong or no Outcome |
| Slotwinski, R., Dabrowska, A., Lech, G., Slodkowski, M.. Innate immunity in pancreatic cancer patients and changes after immuno-enhancing nutrition (P1308). Journal of Immunology. 2013. 190: | Wrong or no Outcome |
| Amaravati, R. S., Sekaran, P.. Does preoperative exercise influence the outcome of ACL reconstruction?. Arthroscopy - Journal of Arthroscopic and Related Surgery. 2013. 29:e182-e183 | Unregistered Abstract |
| Fu, D., Yang, J., Zhu, R., Pan, Q., Shen, X., Peng, Y., Guo, X. R., Wang, F. Z.. Preoperative psychoprophylactic visiting alleviates maternal anxiety and stress and improves outcomes of cesarean patients: A randomized, double-blind and controlled trial. HealthMED. 2012. 6:263-277 | Wrong Population |
| Barbalho-Moulim, M., Costa, D., Miguel, G., Campos, F., Forti, E. M.. Effects of preoperative inspiratory muscle training (IMT) in obese women undergoing open bariatric surgery: Respiratory muscle strength. European Respiratory Journal. 2011. 38: | Duplicate |
| Carli, F., Feldman, L., Charlebois, P., Stein, B.. Effect of prehabilitation on surgical recovery. Canadian Journal of Anesthesia. 2011. 58:S40 | Not a Randomized Trial |
| Tan, C. S. H.. A structured program to improve patient satisfaction and reduce fear during cataract surgery-a randomided controlled study on the efficacy of preoperative counselling. Annals of the Academy of Medicine Singapore. 2011. 40:S181 | Intervention Not Preoperative |
| Martelli, M., Renghi, A., Gramaglia, L., De Simeis, M. L., Mottini, F., Brusita, P.. Preoperative oral nutritional supplementation of carbohydrates in aortic surgery: A randomized trial of 40 patients. Interactive Cardiovascular and Thoracic Surgery. 2011. 12:S177 | Wrong or Unclear Duration |
| Voorham-Van Der Zalm, P. J., Stoetman, A. M., Putter, H., Bevers, R. F. M., Pelger, R. C. M.. Effect of preoperative pelvic floor physiotherapy versus standard care on incontinence in men undergoing radical laparoscopic prostatectomy: An ongoing study. International Urogynecology Journal and Pelvic Floor Dysfunction. 2011. 22:S722-S723 | Wrong or no Outcome |
| Svege, I. C., Fernandes, L., Nordsletten, L., Risberg, M.. Time to total hip replacement surgery after supervised exercise and patient education in patients with hip osteoarthritis. A randomized intervention study with between 3.5 and 6 years follow up. Osteoarthritis and Cartilage. 2011. 19:S44-S45 | Wrong or no Outcome |
| Carver, T. E., Mayo, N., Andersen, R. E., Zavorsky, G. S.. Pilot investigation to evaluate changes in exercise capacity following a prehabilitation intervention among seriously obese patients awaiting bariatric surgery. Canadian Journal of Diabetes. 2011. 35:149 | Wrong or no Outcome |
| Tan, C. S., Yang, F. P., Chew, M. C., Venkatesh, R., Au Eong, K. G.. Improving patient satisfaction and reducing fear during cataract surgery-a randomized controlled study on the efficacy of preoperative counselling. Annals of the Academy of Medicine Singapore. 2010. 39:S253 | Intervention Not Preoperative |
| Cattano, D., Altamirano, A., Melnikov, V., Pivalizza, E., Feldman, A., Hagberg, C.. Postoperative pulmonary inspiratory reserve volume and incentive spirometry in morbidly obese patients undergoing bariatric surgery. Journal of Investigative Medicine. 2010. 58:678-679 | Wrong or Unclear Duration |
| Urinary incontinence after radical prostatectomy: A randomized controlled trial comparing preoperative intensive pelvic muscle exercises with or without proprioceptive training. 2010. | Wrong or Unclear Duration |
| Sivaraman, A., Vanithamani, Manoharan, T. S.. Yoga breathing exercise to reduce postoperative pulmonary complications in patients undergoing elective valve replacement for valvular heart disease: A randomized clinical trial. Heart Surgery Forum. 2010. 13:S83 | Wrong or Unclear Duration |
| Soni, A., Mudge, N., Joshi, A., Wyatt, M., Williamson, L.. Severe knee osteoarthritis: A study of combined acupuncture and physiotherapy vs home exercise advice in patients awaiting total knee arthroplasty. Rheumatology. 2010. 49: | Wrong or no Outcome |
| Ma, B., Bao, H.. Reduction in pulmonary complications in high risk patients undergoing surgery for total hip replacement under general anesthesia by preoperative intensive inspiratory muscle training : A randomized controlled clinical trial. Journal of Nanjing Medical University. 2009. 23:328-334 | Wrong or Unclear Duration |
| Erdem, N. Z., Yasti, A. C., Atli, M., Gozalan, A. U., Dolapci, M., Kama, N. A., Bozkurt, N.. The effects of perioperative oral enteral support with glutamine-added elemental formulas in patients with gastrointestinal cancers. A prospective, randomized, clinical study. Nutrition Research. 2002. 22:977-988 | Wrong or no Outcome |
| Omlor, G., Kiewitz, S., Pietschmann, S., Roesler, S.. The benefit of preoperative psychotherapy on surgical outcome after inguinal hernia repair and thyroid gland surgery. Zentralblatt fur Chirurgie. 2000. 125:380-386 | Wrong or Unclear Duration |
| Burton, M. V., Parker, R. W., Farrell, A., Bailey, D., Conneely, J., Booth, S., Elcombe, S.. A randomized controlled trial of preoperative psychological preparation for mastectomy. Psycho-Oncology. 1995. 4:1-19 | Wrong or Unclear Duration |
| Sameen, Zaka, Talib, Khan, Wani, Shaqul Q., Ashraf, Muntasir, Nengroo, Showkat H.. Preoperative education improves the preparedness for extubation at emergence from general anaesthesia!. Journal of perioperative practice. 2020. :1750458920936213 | Wrong or Unclear Duration |
| Sahar, Wajeeha, Ajaz, Noor, Haider, Zulfiqar, Jalal, Anjum. Effectiveness of Pre-operative Respiratory Muscle Training versus Conventional Treatment for Improving Post operative Pulmonary Health after Coronary Artery Bypass Grafting. Pakistan journal of medical sciences. 2020. 36:1216-1219 | Wrong or Unclear Duration |
| Santa Mina, Daniel, Dolan, Lianne B., Lipton, Jeffrey H., Au, Darren, Camacho Perez, Encarna, Franzese, Alyssa, Alibhai, Shabbir M. H., Jones, Jennifer M., Chang, Eugene. Exercise before, during, and after Hospitalization for Allogeneic Hematological Stem Cell Transplant: A Feasibility Randomized Controlled Trial. Journal of clinical medicine. 2020. 9: | Wrong Population |
| Nejkov, Sonja, Bokan-Mirkovic, Vesna, Dukic-Macut, Natasa, Vukovic, Marina. EFFECT OF PREOPERATIVE RESPIRATORY REHABILITATION IN PATIENTS UNDERGOING CARDIAC SURGERY. Acta clinica Croatica. 2020. 59:597-604 | Wrong or no Outcome |
| Peng, Fei, Peng, Tao, Yang, Qiange, Liu, Meihan, Chen, Guangxiang, Wang, Maohua. Preoperative communication with anesthetists via anesthesia service platform (ASP) helps alleviate patients' preoperative anxiety. Scientific reports. 2020. 10:18708 | Not Prehabilitation |
| Shirdel, Zandi, Behzad, Imani, Manafi, Babak, Saheb, Mehdi. The interactive effect of preoperative consultation and operating room admission by a counselor on anxiety level and vital signs in patients undergoing Coronary Artery Bypass Grafting surgery. A clinical trial study. Investigacion y educacion en enfermeria. 2020. 38: | Wrong or Unclear Duration |
| Resnick, Matthew J.. Re: Effect of Multimodal Prehabilitation vs Postoperative Rehabilitation on 30-Day Postoperative Complications for Frail Patients Undergoing Resection of Colorectal Cancer: A Randomized Clinical Trial. The Journal of urology. 2020. 204:869-870 | Not a Randomized Trial |
| Boden, Ianthe, Robertson, Iain K., Neil, Amanda, Reeve, Julie, Palmer, Andrew J., Skinner, Elizabeth H., Browning, Laura, Anderson, Lesley, Hill, Cat, Story, David, Denehy, Linda. Preoperative physiotherapy is cost-effective for preventing pulmonary complications after major abdominal surgery: a health economic analysis of a multicentre randomised trial. Journal of physiotherapy. 2020. 66:180-187 | Not Prehabilitation |
| Moug, S. J., Barry, S. J. E., Maguire, S., Johns, N., Dolan, D., Steele, R. J. C., Buchan, C., Mackay, G., Anderson, A. S., Mutrie, N.. Does prehabilitation modify muscle mass in patients with rectal cancer undergoing neoadjuvant therapy? A subanalysis from the REx randomised controlled trial. Techniques in coloproctology. 2020. 24:959-964 | Wrong or no Outcome |
| Vereeck, S., Neels, H., Govaerts, J., Jacquemyn, Y.. Re: Effect of preoperative pelvic floor muscle training on pelvic floor muscle contraction and symptomatic and anatomical pelvic organ prolapse after surgery: randomized controlled trial. Ultrasound in obstetrics & gynecology : the official journal of the International Society of Ultrasound in Obstetrics and Gynecology. 2020. 56:120-121 | Not a Randomized Trial |
| Soffin, Ellen M., Beckman, James D., Tseng, Audrey, Zhong, Haoyan, Huang, Russel C., Urban, Michael, Guheen, Carrie R., Kim, Han-Jo, Cammisa, Frank P., Nejim, Jemiel A., Schwab, Frank J., Armendi, Isabel F., Memtsoudis, Stavros G.. Enhanced Recovery after Lumbar Spine Fusion: A Randomized Controlled Trial to Assess the Quality of Patient Recovery. Anesthesiology. 2020. 133:350-363 | Not Prehabilitation |
| Blackwell, J. E. M., Doleman, B., Boereboom, C. L., Morton, A., Williams, S., Atherton, P., Smith, K., Williams, J. P., Phillips, B. E., Lund, J. N.. High-intensity interval training produces a significant improvement in fitness in less than 31 days before surgery for urological cancer: a randomised control trial. Prostate cancer and prostatic diseases. 2020. 23:696-704 | Wrong or no Outcome |
| Zhang, Xiaoyuan, Huang, Hongshi, Yu, Yuanyuan, Yang, Jie, Liang, Zixuan, Chang, Cuiqing. Impact of whey protein isolate and eccentric training on quadriceps mass and strength in patients with anterior cruciate ligament rupture: A randomized controlled trial. Journal of rehabilitation medicine. 2020. 52:jrm00035 | Not Prehabilitation |
| He, Dan, Wang, Fu Zhe, Zhang, Zhan, Huang, Feng, Chen, Jiao Jiao, Li, Bai. [Effect of low-frequency electrical acupoint stimulation on gastrointestinal motility function following radical gastrectomy in patients with gastric cancer]. Zhen ci yan jiu = Acupuncture research. 2020. 45:51-6 | Intervention Not Preoperative |
| Wang, Bei, Shelat, Vishalkumar G., Chow, Jaclyn Jie Ling, Huey, Terence Cheong Wei, Low, Jee Keem, Woon, Winston Wei Liang, Junnarkar, Sameer P.. Prehabilitation Program Improves Outcomes of Patients Undergoing Elective Liver Resection. The Journal of surgical research. 2020. 251:119-125 | Not a Randomized Trial |
| Wood, William A., Weaver, M., Smith-Ryan, A. E., Hanson, E. D., Shea, T. C., Battaglini, C. L.. Lessons learned from a pilot randomized clinical trial of home-based exercise prescription before allogeneic hematopoietic cell transplantation. Supportive care in cancer : official journal of the Multinational Association of Supportive Care in Cancer. 2020. 28:5291-5298 | Wrong Population |
| Briguglio, Matteo, Hrelia, Silvana, Malaguti, Marco, De Vecchi, Elena, Lombardi, Giovanni, Banfi, Giuseppe, Riso, Patrizia, Porrini, Marisa, Romagnoli, Sergio, Pino, Fabio, Crespi, Tiziano, Perazzo, Paolo. Oral Supplementation with Sucrosomial Ferric Pyrophosphate Plus L-Ascorbic Acid to Ameliorate the Martial Status: A Randomized Controlled Trial. Nutrients. 2020. 12: | Single Risk Factor Management |
| Onerup, Aron, Thorn, Sven-Egron, Angenete, Eva, Bock, David, Gryback Gillheimer, Elin, Haglind, Eva, Nilsson, Hanna. Effects of a home-based exercise program on the insulin-like growth factor axis in patients operated for colorectal cancer in Sweden: Results from the randomised controlled trial PHYSSURG-C. Growth hormone & IGF research : official journal of the Growth Hormone Research Society and the International IGF Research Society. 2020. 51:27-33 | Wrong or no Outcome |
| Anan, Go, Kaiho, Yasuhiro, Iwamura, Hiromichi, Ito, Jun, Kohada, Yuki, Mikami, Jotaro, Sato, Makoto. Preoperative pelvic floor muscle exercise for early continence after holmium laser enucleation of the prostate: a randomized controlled study. BMC urology. 2020. 20:3 | Wrong or Unclear Duration |
| Morkane, Clare M., Kearney, Orla, Bruce, David A., Melikian, Clare N., Martin, Daniel S.. An Outpatient Hospital-based Exercise Training Program for Patients With Cirrhotic Liver Disease Awaiting Transplantation: A Feasibility Trial. Transplantation. 2020. 104:97-103 | Not a Randomized Trial |
| Martinez, Jose L., Bosco-Garate, Ilka, Souza-Gallardo, Luis Manuel, Mendez, Jose D., Juarez-Oropeza, Marco A., Roman-Ramos, Ruben, Ferat-Osorio, Eduardo. Effect of Preoperative Administration of Oral Arginine and Glutamine in Patients with Enterocutaneous Fistula Submitted to Definitive Surgery: a Prospective Randomized Trial. Journal of gastrointestinal surgery : official journal of the Society for Surgery of the Alimentary Tract. 2020. 24:426-434 | Not Prehabilitation |
| Franko, Jan, Raman, Shankar, Krishnan, Nivedita, Frankova, Daniela, Tee, May C., Brahmbhatt, Rushin, Goldman, Charles D., Weigel, Ronald J.. Randomized Trial of Perioperative Probiotics Among Patients Undergoing Major Abdominal Operation. Journal of the American College of Surgeons. 2019. 229:533-540.e1 | Wrong or Unclear Duration |
| Khan Bhettani, Mehreen, Rehman, Mubarik, Ahmed, Moiz, Altaf, Humera Naz, Choudry, Usama Khalid, Khan, Kamran Hakeem. Role of pre-operative vitamin D supplementation to reduce post-thyroidectomy hypocalcemia; Cohort study. International journal of surgery (London, England). 2019. 71:85-90 | Single Risk Factor Management |
| Bhatia, Chetna, Kayser, Bengt. Preoperative high-intensity interval training is effective and safe in deconditioned patients with lung cancer: A randomized clinical trial. Journal of rehabilitation medicine. 2019. 51:712-718 | Wrong or no Outcome |
| Zong, Lei, Li, Haiyan, Li, Shuhua. Effects of neoadjuvant chemotherapy combined with enteral nutrition on perioperative immunity, inflammation and intestinal flora in gastric cancer patients. Journal of B.U.ON. : official journal of the Balkan Union of Oncology. 2019. 24:1113-1119 | Wrong or no Outcome |
| Wallen, Matthew P., Keating, Shelley E., Hall, Adrian, Hickman, Ingrid J., Pavey, Toby G., Woodward, Aidan J., Skinner, Tina L., Macdonald, Graeme A., Coombes, Jeff S.. Exercise Training Is Safe and Feasible in Patients Awaiting Liver Transplantation: A Pilot Randomized Controlled Trial. Liver transplantation : official publication of the American Association for the Study of Liver Diseases and the International Liver Transplantation Society. 2019. 25:1576-1580 | Wrong or no Outcome |
| Moradian, Seyed Tayeb, Heydari, Amir Abas, Mahmoudi, Hosein. What is the Role of Preoperative Breathing Exercises in Reducing Postoperative Atelectasis after CABG?. Reviews on recent clinical trials. 2019. 14:275-279 | Wrong or Unclear Duration |
| Guo, Miao, Lu, Ling, Sun, Yu, Li, Lei, Wu, Ming, Lang, Jinghe. Comprehensive functional exercises with patient education for the prevention of venous thrombosis after major gynecologic surgery: A randomized controlled study. Thrombosis research. 2019. 178:69-74 | Wrong or Unclear Duration |
| Christensen, J. F., Simonsen, C., Banck-Petersen, A., Thorsen-Streit, S., Herrstedt, A., Djurhuus, S. S., Egeland, C., Mortensen, C. E., Kofoed, S. C., Kristensen, T. S., Garbyal, R. S., Pedersen, B. K., Svendsen, L. B., Hojman, P., de Heer, P.. Safety and feasibility of preoperative exercise training during neoadjuvant treatment before surgery for adenocarcinoma of the gastro-oesophageal junction. BJS open. 2019. 3:74-84 | Not a Randomized Trial |
| Miyauchi, Youhei, Furukawa, Katsunori, Suzuki, Daisuke, Yoshitomi, Hideyuki, Takayashiki, Tsukasa, Kuboki, Satoshi, Miyazaki, Masaru, Ohtsuka, Masayuki. Additional effect of perioperative, compared with preoperative, immunonutrition after pancreaticoduodenectomy: A randomized, controlled trial. International journal of surgery (London, England). 2019. 61:69-75 | Wrong or Unclear Duration |
| das Nair, Roshan, Mhizha-Murira, Jacqueline R., Anderson, Pippa, Carpenter, Hannah, Clarke, Simon, Groves, Sam, Leighton, Paul, Scammell, Brigitte E., Topcu, Gogem, Walsh, David A., Lincoln, Nadina B.. Home-based pre-surgical psychological intervention for knee osteoarthritis (HAPPiKNEES): a feasibility randomized controlled trial. Clinical rehabilitation. 2018. 32:777-789 | Wrong or no Outcome |
| Minnella, Enrico M., Awasthi, Rashami, Loiselle, Sarah-Eve, Agnihotram, Ramanakumar V., Ferri, Lorenzo E., Carli, Francesco. Effect of Exercise and Nutrition Prehabilitation on Functional Capacity in Esophagogastric Cancer Surgery: A Randomized Clinical Trial. JAMA surgery. 2018. 153:1081-1089 | Duplicate |
| Steurer, Johann. . Operationen im oberen Abdominalbereich: Praoperative Physiotherapie reduziert Risiko pulmonaler Komplikationen.. 2018. 107:605-606 | Not a Randomized Trial |
| Forsmo, Havard Mjorud, Erichsen, Christian, Rasdal, Anne, Tvinnereim, Jon Meyer, Korner, Hartwig, Pfeffer, Frank. Randomized Controlled Trial of Extended Perioperative Counseling in Enhanced Recovery After Colorectal Surgery. Diseases of the colon and rectum. 2018. 61:724-732 | Not Prehabilitation |
| Valkenet, K., Trappenburg, J. C. A., Ruurda, J. P., Guinan, E. M., Reynolds, J. V., Nafteux, P., Fontaine, M., Rodrigo, H. E., van der Peet, D. L., Hania, S. W., Sosef, M. N., Willms, J., Rosman, C., Pieters, H., Scheepers, J. J. G., Faber, T., Kouwenhoven, E. A., Tinselboer, M., Rasanen, J., Ryynanen, H., Gosselink, R., van Hillegersberg, R., Backx, F. J. G.. Multicentre randomized clinical trial of inspiratory muscle training versus usual care before surgery for oesophageal cancer. The British journal of surgery. 2018. 105:502-511 | Duplicate |
| Wilschut, Esther D., Rotmans, Joris I., Bos, Ernst Jan, van Zoest, Danielle, Eefting, Daniel, Hamming, Jaap F., van der Bogt, Koen E. A.. Supervised preoperative forearm exercise to increase blood vessel diameter in patients requiring an arteriovenous access for hemodialysis: rationale and design of the PINCH trial. The journal of vascular access. 2018. 19:84-88 | Protocol |
| Boden, Ianthe, El-Ansary, Doa, Zalucki, Nadia, Robertson, Iain K., Browning, Laura, Skinner, Elizabeth H., Denehy, Linda. Physiotherapy education and training prior to upper abdominal surgery is memorable and has high treatment fidelity: a nested mixed-methods randomised-controlled study. Physiotherapy. 2018. 104:194-202 | Not Prehabilitation |
| Gorecki, Patricia, Burke, Danielle L., Chapple, Iain L. C., Hemming, Karla, Saund, Daniel, Pearson, David, Stahl, Wilhelm, Lello, Ryan, Dietrich, Thomas. Perioperative supplementation with a fruit and vegetable juice powder concentrate and postsurgical morbidity: A double-blind, randomised, placebo-controlled clinical trial. Clinical nutrition (Edinburgh, Scotland). 2018. 37:1448-1455 | Not Prehabilitation |
| Lluch, Enrique, Duenas, Lirios, Falla, Deborah, Baert, Isabel, Meeus, Mira, Sanchez-Frutos, Jose, Nijs, Jo. Preoperative Pain Neuroscience Education Combined With Knee Joint Mobilization for Knee Osteoarthritis: A Randomized Controlled Trial. The Clinical journal of pain. 2018. 34:44-52 | Not Prehabilitation |
| Demark-Wahnefried, Wendy, Rais-Bahrami, Soroush, Desmond, Renee A., Gordetsky, Jennifer B., Hunter, Gary R., Yang, Eddy S., Azrad, Maria, Fruge, Andrew D., Tsuruta, Yuko, Norian, Lyse A., Segal, Roanne, Grizzle, William E.. Presurgical weight loss affects tumour traits and circulating biomarkers in men with prostate cancer. British journal of cancer. 2017. 117:1303-1313 | Wrong or no Outcome |
| Salzmann, Stefan, Euteneuer, Frank, Laferton, Johannes A. C., Auer, Charlotte J., Shedden-Mora, Meike C., Schedlowski, Manfred, Moosdorf, Rainer, Rief, Winfried. Effects of Preoperative Psychological Interventions on Catecholamine and Cortisol Levels After Surgery in Coronary Artery Bypass Graft Patients: The Randomized Controlled PSY-HEART Trial. Psychosomatic medicine. 2017. 79:806-814 | Wrong or Unclear Duration |
| Auer, Charlotte J., Laferton, Johannes A. C., Shedden-Mora, Meike C., Salzmann, Stefan, Moosdorf, Rainer, Rief, Winfried. Optimizing preoperative expectations leads to a shorter length of hospital stay in CABG patients: Further results of the randomized controlled PSY-HEART trial. Journal of psychosomatic research. 2017. 97:82-89 | Wrong or Unclear Duration |
| Ertug, Nurcan, Ulusoylu, Ozge, Bal, Ayca, Ozgur, Hazal. Comparison of the effectiveness of two different interventions to reduce preoperative anxiety: A randomized controlled study. Nursing & health sciences. 2017. 19:250-256 | Wrong or Unclear Duration |
| Kitagawa, Hiroyuki, Namikawa, Tsutomu, Yatabe, Tomoaki, Munekage, Masaya, Yamasaki, Fumiyasu, Kobayashi, Michiya, Hanazaki, Kazuhiro. Effects of a preoperative immune-modulating diet in patients with esophageal cancer: a prospective parallel group randomized study. Langenbeck's archives of surgery. 2017. 402:531-538 | Wrong or Unclear Duration |
| Bond, Dale S., Raynor, Hollie A., Thomas, J. Graham, Unick, Jessica, Webster, Jennifer, Ryder, Beth, Vithiananthan, Sivamainthan. Greater Adherence to Recommended Morning Physical Activity is Associated With Greater Total Intervention-Related Physical Activity Changes in Bariatric Surgery Patients. Journal of physical activity & health. 2017. 14:492-498 | Not a Randomized Trial |
| Turky, Khalid, Afify, Amera M. Abdelaziz. Effect of Preoperative Inspiratory Muscle Training on Alveolar-Arterial Oxygen Gradients After Coronary Artery Bypass Surgery. Journal of cardiopulmonary rehabilitation and prevention. 2017. 37:290-294 | Wrong or no Outcome |
| Rief, Winfried, Shedden-Mora, Meike C., Laferton, Johannes A. C., Auer, Charlotte, Petrie, Keith J., Salzmann, Stefan, Schedlowski, Manfred, Moosdorf, Rainer. Preoperative optimization of patient expectations improves long-term outcome in heart surgery patients: results of the randomized controlled PSY-HEART trial. BMC medicine. 2017. 15:4 | Wrong or Unclear Duration |
| Burden, Sorrel T., Gibson, Debra J., Lal, Simon, Hill, James, Pilling, Mark, Soop, Mattias, Ramesh, Aswatha, Todd, Chris. Pre-operative oral nutritional supplementation with dietary advice versus dietary advice alone in weight-losing patients with colorectal cancer: single-blind randomized controlled trial. Journal of cachexia, sarcopenia and muscle. 2017. 8:437-446 | Wrong or Unclear Duration |
| Marcon, Emilian Rejane, Baglioni, S., Bittencourt, L., Lopes, C. L. N., Neumann, C. R., Trindade, M. R. M.. What Is the Best Treatment before Bariatric Surgery? Exercise, Exercise and Group Therapy, or Conventional Waiting: a Randomized Controlled Trial. Obesity surgery. 2017. 27:763-773 | Wrong or no Outcome |
| Camolas, Jose, Santos, Osvaldo, Moreira, Pedro, do Carmo, Isabel. INDIVIDUO: Results from a patient-centered lifestyle intervention for obesity surgery candidates. Obesity research & clinical practice. 2017. 11:475-488 | Wrong or no Outcome |
| Vaegter, Henrik B., Handberg, Gitte, Emmeluth, Claus, Graven-Nielsen, Thomas. Preoperative Hypoalgesia After Cold Pressor Test and Aerobic Exercise is Associated With Pain Relief 6 Months After Total Knee Replacement. The Clinical journal of pain. 2017. 33:475-484 | Not a Randomized Trial |
| Nardi, Paolo, Pellegrino, Antonio, Pisano, Calogera, Vacirca, Sara Rita, Anselmi, Deborah, Saulle, Silvia, Dandi, Romana, Romano, Alessia, Servadio, Annamaria, Gianlorenzi, Alessandra, Ruvolo, Giovanni. The effect of preoperative respiratory physiotherapy and motor exercise in patients undergoing elective cardiac surgery: short-term results. Kardiochirurgia i torakochirurgia polska = Polish journal of cardio-thoracic surgery. 2019. 16:81-87 | Wrong or Unclear Duration |
| Krasowska, Katarzyna, Skrobot, Wojciech, Liedtke, Ewelina, Sawicki, Piotr, Flis, Damian Jozef, Dzik, Katarzyna Patrycja, Libionka, Witold, Kloc, Wojciech, Kaczor, Jan Jacek. The Preoperative Supplementation With Vitamin D Attenuated Pain Intensity and Reduced the Level of Pro-inflammatory Markers in Patients After Posterior Lumbar Interbody Fusion. Frontiers in pharmacology. 2019. 10:527 | Not Prehabilitation |
| Aragoncillo, Ines, Ligero, Jose Manuel, Hevia, Covadonga, Morales, Angel Luis, Amezquita, Yesika, Cervera, Teresa, Vega, Almudena, Abad, Soraya, Macias, Nicolas, Luno, Jose. Rationale and design of the PHYSICALFAV trial: a randomized controlled trial to evaluate the effect of preoperative isometric exercise on vascular calibre and maturation of autologous arteriovenous fistulas. Clinical kidney journal. 2018. 11:841-845 | Protocol |
| van Eck, Carola F., Toor, Aneet, Banffy, Michael B., Gambardella, Ralph A.. Web-Based Education Prior to Outpatient Orthopaedic Surgery Enhances Early Patient Satisfaction Scores: A Prospective Randomized Controlled Study. Orthopaedic journal of sports medicine. 2018. 6:2325967117751418 | Single Risk Factor Management |
| Wu, Brian, Lorezanza, Dan, Badash, Ido, Berger, Max, Lane, Christianne, Sum, Jonathan C., Hatch, George F., 3rd, Schroeder, E. Todd. Perioperative Testosterone Supplementation Increases Lean Mass in Healthy Men Undergoing Anterior Cruciate Ligament Reconstruction: A Randomized Controlled Trial. Orthopaedic journal of sports medicine. 2017. 5:2325967117722794 | Single Risk Factor Management |
| Creel, David B., Schuh, Leslie M., Reed, Christina A., Gomez, Adrienne R., Hurst, Lori A., Stote, Joseph, Cacucci, Brenda M.. A randomized trial comparing two interventions to increase physical activity among patients undergoing bariatric surgery. Obesity (Silver Spring, Md.). 2016. 24:1660-8 | Wrong or no Outcome |
| Forsmo, H. M., Pfeffer, F., Rasdal, A., Ostgaard, G., Mohn, A. C., Korner, H., Erichsen, C.. Compliance with enhanced recovery after surgery criteria and preoperative and postoperative counselling reduces length of hospital stay in colorectal surgery: results of a randomized controlled trial. Colorectal disease : the official journal of the Association of Coloproctology of Great Britain and Ireland. 2016. 18:603-11 | Wrong or Unclear Duration |
| Sommer, Maja S., Trier, Karen, Vibe-Petersen, Jette, Missel, Malene, Christensen, Merete, Larsen, Klaus R., Langer, Seppo W., Hendriksen, Carsten, Clementsen, Paul Frost, Pedersen, Jesper H., Langberg, Henning. Perioperative Rehabilitation in Operable Lung Cancer Patients (PROLUCA): A Feasibility Study. Integrative cancer therapies. 2016. 15:455-466 | Wrong or Unclear Duration |
| Tan, Chun Khui, Said, Suraya, Rajandram, Retnagowri, Wang, Zhiqiang, Roslani, April Camilla, Chin, Kin Fah. Pre-surgical Administration of Microbial Cell Preparation in Colorectal Cancer Patients: A Randomized Controlled Trial. World journal of surgery. 2016. 40:1985-92 | Not Prehabilitation |
| Alito, Miguel Aprelino, de Aguilar-Nascimento, Jose Eduardo. Multimodal perioperative care plus immunonutrition versus traditional care in total hip arthroplasty: a randomized pilot study. Nutrition journal. 2016. 15:34 | Wrong or Unclear Duration |
| Baillot, Aurelie, Mampuya, Warner M., Dionne, Isabelle J., Comeau, Emilie, Meziat-Burdin, Anne, Langlois, Marie-France. Impacts of Supervised Exercise Training in Addition to Interdisciplinary Lifestyle Management in Subjects Awaiting Bariatric Surgery: a Randomized Controlled Study. Obesity surgery. 2016. 26:2602-2610 | Wrong or no Outcome |
| Seguin, Philippe, Locher, Clara, Boudjema, Karim, Hamon, Catherine, Mouchel, Catherine, Malledant, Yannick, Bellissant, Eric. Effect of a Perioperative Nutritional Supplementation with Oral Impact R in Patients undergoing Hepatic Surgery for Liver Cancer: A Prospective, Placebo-Controlled, Randomized, Double-Blind Study. Nutrition and cancer. 2016. 68:464-72 | Wrong or no Outcome |
| Cassin, Stephanie E., Sockalingam, Sanjeev, Du, Chau, Wnuk, Susan, Hawa, Raed, Parikh, Sagar V.. A pilot randomized controlled trial of telephone-based cognitive behavioural therapy for preoperative bariatric surgery patients. Behaviour research and therapy. 2016. 80:17-22 | Wrong or no Outcome |
| Gade, Josephine, Levring, Trine, Hillingso, Jens, Hansen, Carsten Palnaes, Andersen, Jens Rikardt. The Effect of Preoperative Oral Immunonutrition on Complications and Length of Hospital Stay After Elective Surgery for Pancreatic Cancer--A Randomized Controlled Trial. Nutrition and cancer. 2016. 68:225-33 | Duplicate |
| Demark-Wahnefried, Wendy, Nix, Jeffery W., Hunter, Gary R., Rais-Bahrami, Soroush, Desmond, Renee A., Chacko, Balu, Morrow, Casey D., Azrad, Maria, Fruge, Andrew D., Tsuruta, Yuko, Ptacek, Travis, Tully, Scott A., Segal, Roanne, Grizzle, William E.. Feasibility outcomes of a presurgical randomized controlled trial exploring the impact of caloric restriction and increased physical activity versus a wait-list control on tumor characteristics and circulating biomarkers in men electing prostatectomy for prostate cancer. BMC cancer. 2016. 16:61 | Wrong or no Outcome |
| Ener, K., Aldemir, M., Isik, E., Okulu, E., Ozcan, M. F., Ugurlu, M., Tangal, S., Ozayar, A.. The impact of vitamin E supplementation on semen parameters and pregnancy rates after varicocelectomy: a randomised controlled study. Andrologia. 2016. 48:829-34 | Intervention Not Preoperative |
| Rolving, Nanna, Sogaard, Rikke, Nielsen, Claus Vinther, Christensen, Finn Bjarke, Bunger, Cody, Oestergaard, Lisa Gregersen. Preoperative Cognitive-Behavioral Patient Education Versus Standard Care for Lumbar Spinal Fusion Patients: Economic Evaluation Alongside a Randomized Controlled Trial. Spine. 2016. 41:18-25 | Duplicate |
| Hermann, A., Holsgaard-Larsen, A., Zerahn, B., Mejdahl, S., Overgaard, S.. Preoperative progressive explosive-type resistance training is feasible and effective in patients with hip osteoarthritis scheduled for total hip arthroplasty--a randomized controlled trial. Osteoarthritis and cartilage. 2016. 24:91-8 | Wrong or no Outcome |
| Laferton, Johannes A. C., Auer, Charlotte J., Shedden-Mora, Meike C., Moosdorf, Rainer, Rief, Winfried. Optimizing preoperative expectations in cardiac surgery patients is moderated by level of disability: the successful development of a brief psychological intervention. Psychology, health & medicine. 2016. 21:272-85 | Wrong or no Outcome |
| Jepson, Paul, Sands, Gina, Beswick, Andrew D., Davis, Edward T., Blom, Ashley W., Sackley, Catherine M.. A feasibility randomised controlled trial of pre-operative occupational therapy to optimise recovery for patients undergoing primary total hip replacement for osteoarthritis (PROOF-THR). Clinical rehabilitation. 2016. 30:156-66 | Wrong or Unclear Duration |
| Schmidt, Maren, Eckardt, Rahel, Scholtz, Kathrin, Neuner, Bruno, von Dossow-Hanfstingl, Vera, Sehouli, Jalid, Stief, Christian G., Wernecke, Klaus-Dieter, Spies, Claudia D., Group, Peratecs. Patient Empowerment Improved Perioperative Quality of Care in Cancer Patients Aged >= 65 Years - A Randomized Controlled Trial. PloS one. 2015. 10:e0137824 | Wrong or Unclear Duration |
| Yin, Bob, Goldsmith, Laura, Gambardella, Ralph. Web-Based Education Prior to Knee Arthroscopy Enhances Informed Consent and Patient Knowledge Recall: A Prospective, Randomized Controlled Study. The Journal of bone and joint surgery. American volume. 2015. 97:964-71 | Not Prehabilitation |
| Wang, Jin-Yi, Hong, Xuan, Chen, Guo-Han, Li, Qin-Chuan, Liu, Zhong-Min. Clinical application of the fast track surgery model based on preoperative nutritional risk screening in patients with esophageal cancer. Asia Pacific journal of clinical nutrition. 2015. 24:206-11 | Wrong or Unclear Duration |
| Paul, Linda, van Rongen, Sofie, van Hoeken, Daphne, Deen, Mathijs, Klaassen, Rene, Biter, L. Ulas, Hoek, Hans W., van der Heiden, Colin. Does cognitive behavioral therapy strengthen the effect of bariatric surgery for obesity? Design and methods of a randomized and controlled study. Contemporary clinical trials. 2015. 42:252-6 | Protocol |
| Saleh, Amin J., Tang, Guan-Xiu, Hadi, Sally M., Yan, Liao, Chen, Ming-Hua, Duan, Kai-Ming, Tong, Jianbin, Ouyang, Wen. Preoperative cognitive intervention reduces cognitive dysfunction in elderly patients after gastrointestinal surgery: a randomized controlled trial. Medical science monitor : international medical journal of experimental and clinical research. 2015. 21:798-805 | Wrong or Unclear Duration |
| Bond, Dale S., Thomas, J. Graham, King, Wendy C., Vithiananthan, Sivamainthan, Trautvetter, Jennifer, Unick, Jessica L., Ryder, Beth A., Pohl, Dieter, Roye, G. Dean, Sax, Harry C., Wing, Rena R.. Exercise improves quality of life in bariatric surgery candidates: results from the Bari-Active trial. Obesity (Silver Spring, Md.). 2015. 23:536-42 | Wrong or no Outcome |
| Grass, F., Bertrand, P. C., Schafer, M., Ballabeni, P., Cerantola, Y., Demartines, N., Hubner, M.. Compliance with preoperative oral nutritional supplements in patients at nutritional risk--only a question of will?. European journal of clinical nutrition. 2015. 69:525-9 | Not a Randomized Trial |
| Bond, Dale S., Vithiananthan, Sivamainthan, Thomas, J. Graham, Trautvetter, Jennifer, Unick, Jessica L., Jakicic, John M., Pohl, Dieter, Ryder, Beth A., Roye, G. Dean, Sax, Harry C., Wing, Rena R.. Bari-Active: a randomized controlled trial of a preoperative intervention to increase physical activity in bariatric surgery patients. Surgery for obesity and related diseases : official journal of the American Society for Bariatric Surgery. 2015. 11:169-77 | Wrong or no Outcome |
| Plank, Lindsay D., Mathur, Sachin, Gane, Edward J., Peng, Sze-Lin, Gillanders, Lyn K., McIlroy, Kerry, Chavez, Carolina Paras, Calder, Philip C., McCall, John L.. Perioperative immunonutrition in patients undergoing liver transplantation: a randomized double-blind trial. Hepatology (Baltimore, Md.). 2015. 61:639-47 | Wrong or Unclear Duration |
| Azman, Mawaddah, Mohd Yunus, Mohd Razif, Sulaiman, Suhaina, Syed Omar, Syed Nabil. Enteral glutamine supplementation in surgical patients with head and neck malignancy: A randomized controlled trial. Head & neck. 2015. 37:1799-807 | Intervention Not Preoperative |
| Svege, Ida, Nordsletten, Lars, Fernandes, Linda, Risberg, May Arna. Exercise therapy may postpone total hip replacement surgery in patients with hip osteoarthritis: a long-term follow-up of a randomised trial. Annals of the rheumatic diseases. 2015. 74:164-9 | Wrong or no Outcome |
| Santa Mina, Daniel, Matthew, Andrew G., Hilton, William J., Au, Darren, Awasthi, Rashami, Alibhai, Shabbir M. H., Clarke, Hance, Ritvo, Paul, Trachtenberg, John, Fleshner, Neil E., Finelli, Antonio, Wijeysundera, Duminda, Aprikian, Armen, Tanguay, Simon, Carli, Franco. Prehabilitation for men undergoing radical prostatectomy: a multi-centre, pilot randomized controlled trial. BMC surgery. 2014. 14:89 | Wrong or no Outcome |
| Limongi, V., dos Santos, D. C., da Silva, A. M. O., Ataide, E. C., Mei, M. F. T., Udo, E. Y., Boin, I. F. S. F., Stucchi, R. S. B.. Effects of a respiratory physiotherapeutic program in liver transplantation candidates. Transplantation proceedings. 2014. 46:1775-7 | Wrong Population |
| Heinberg, Leslie J., Schauer, Philip R.. Pilot testing of a portion-controlled, commercially available diet on presurgical weight loss and metabolic outcomes in patients undergoing bariatric surgery. Obesity surgery. 2014. 24:1817-20 | Wrong or no Outcome |
| Barakat, Hashem M., Shahin, Yousef, Barnes, Rachel, Gohil, Risha, Souroullas, Panos, Khan, Junaid, McCollum, Peter T., Chetter, Ian C.. Supervised exercise program improves aerobic fitness in patients awaiting abdominal aortic aneurysm repair. Annals of vascular surgery. 2014. 28:74-9 | Wrong or no Outcome |
| [Clinical observation of preoperative administration of enteral nutrition support in gastric cancer patients at risk of malnutrition]. 2013. | Wrong or no Outcome |
| Laurienzo, Carla Elaine, Sacomani, Carlos Alberto Ricetto, Rodrigues, Telma Ribeiro, Zequi, Stenio de Cassio, Guimaraes, Gustavo Cardoso, Lopes, Ademar. Results of preoperative electrical stimulation of pelvic floor muscles in the continence status following radical retropubic prostatectomy. International braz j urol : official journal of the Brazilian Society of Urology. 2013. 39:182-8 | Wrong or Unclear Duration |
| Zareba, K., Czygier, M., Kamocki, Z., Cepowicz, D., Szmitkowski, M., Kedra, B.. Parenteral nutrition and preOp preparation in prevention of post-operative insulin resistance in gastrointestinal carcinoma. Advances in medical sciences. 2013. 58:150-5 | Wrong or Unclear Duration |
| Liu, Yan, Tao, Kai-xiong, Wang, Guo-bin, Lu, Xin, Li, Xiao-hui, Huang, Yang, Ye, Fang. [Effect of enteral nutrition as replacement of traditional bowel preparation on the intraperitoneal and intraluminal disseminated tumor cells, recurrence and metastasis in patients with colorectal cancer]. Zhonghua wei chang wai ke za zhi = Chinese journal of gastrointestinal surgery. 2013. 16:350-3 | Wrong or Unclear Duration |
| Kalarchian, M. A., Marcus, M. D., Courcoulas, A. P., Cheng, Y., Levine, M. D.. Preoperative lifestyle intervention in bariatric surgery: initial results from a randomized, controlled trial. Obesity (Silver Spring, Md.). 2013. 21:254-60 | Wrong or no Outcome |
| Oliphant, Sallie S., Lowder, Jerry L., Ghetti, Chiara, Zyczynski, Halina M.. Effect of a preoperative self-catheterization video on anxiety: a randomized controlled trial. International urogynecology journal. 2013. 24:419-24 | Not Prehabilitation |
| Castello-Simoes, Viviane, Polaquini Simoes, Rodrigo, Beltrame, Thomas, Bassi, Daniela, Maria Catai, Aparecida, Arena, Ross, Azambuja, Noe Carvalho, Jr., do Nascimento Ortega, Joao, Borghi-Silva, Audrey. Effects of aerobic exercise training on variability and heart rate kinetic during submaximal exercise after gastric bypass surgery--a randomized controlled trial. Disability and rehabilitation. 2013. 35:334-42 | Intervention Not Preoperative |
| Alghadir, Ahmad, Iqbal, Zaheen Ahmed, Anwer, Shahnawaz. Comparison of the effect of pre- and post-operative physical therapy versus post-operative physical therapy alone on pain and recovery of function after total knee arthroplasty. Journal of physical therapy science. 2016. 28:2754-2758 | Wrong or Unclear Duration |
| Samnani, Sunil Sadruddin, Umer, Muhammad Farooq, Mehdi, Syed Hussain, Farid, Farah Naz. Impact of Preoperative Counselling on Early Postoperative Mobilization and Its Role in Smooth Recovery. International scholarly research notices. 2014. 2014:250536 | Intervention Not Preoperative |
| Wang, Fen, Li, Chun-Bo, Li, Shenghua, Li, Quan. Integrated interventions for improving negative emotions and stress reactions of young women receiving total hysterectomy. International journal of clinical and experimental medicine. 2014. 7:331-6 | Wrong or Unclear Duration |
| Gopinath, Ramachandran, Yelliboina, Sreekanth, Singh, Madhavi, Prasad, V. B. N.. Impact of supplementing preoperative intravenous omega 3 Fatty acids in fish oil on immunomodulation in elderly patients undergoing hip surgery. The Indian journal of surgery. 2013. 75:478-84 | Wrong or Unclear Duration |
| Caglayan, Kasim, Oner, Ibrahim, Gunerhan, Yusuf, Ata, Pinar, Koksal, Neset, Ozkara, Selvinaz. The impact of preoperative immunonutrition and other nutrition models on tumor infiltrative lymphocytes in colorectal cancer patients. American journal of surgery. 2012. 204:416-21 | Wrong or no Outcome |
| Hubner, M., Cerantola, Y., Grass, F., Bertrand, P. C., Schafer, M., Demartines, N.. Preoperative immunonutrition in patients at nutritional risk: results of a double-blinded randomized clinical trial. European journal of clinical nutrition. 2012. 66:850-5 | Wrong or Unclear Duration |
| Anderson, Roger T., Kimmick, Gretchen G., McCoy, Thomas P., Hopkins, Judith, Levine, Edward, Miller, Gary, Ribisl, Paul, Mihalko, Shannon L.. A randomized trial of exercise on well-being and function following breast cancer surgery: the RESTORE trial. Journal of cancer survivorship : research and practice. 2012. 6:172-81 | Not Prehabilitation |
| Nickkholgh, Arash, Schneider, Heinz, Encke, Jens, Buchler, Markus W., Schmidt, Jan, Schemmer, Peter. PROUD: effects of preoperative long-term immunonutrition in patients listed for liver transplantation. Trials. 2007. 8:20 | Protocol |
| Gomez Sanchez, Ma B., Garcia Talavera Espin, N. V., Monedero Saiz, T., Sanchez Alvarez, C., Zomeno Ros, A. I., Nicolas Hernandez, M., Gomez Ramos, Ma J., Parra Banos, P., Gonzalez Valverde, F. M.. [Evaluation of perioperative nutritional therapy in patients with gastrointestinal tract neoplasms]. Evaluacion de la terapia nutricional perioperatoria en pacientes con neoplasia del tracto gastrointestinal superior.. 2011. 26:1073-80 | Not a Randomized Trial |
| Slotwinski, Robert, Olszewski, Waldemar, Slodkowski, Maciej, Lech, Gustaw, Zaleska, Marzanna, Kedziora, Sylwia, Wluka, Anna, Domaszewska, Anna, Slotwinska, Sylwia, Krasnodebski, Wojciech, Wojcik, Zdzislaw. Apoptosis in lymphocytes of pancreatic cancer patients: influence of preoperative enteral immunonutrition and extensive surgery. Archivum immunologiae et therapiae experimentalis. 2011. 59:385-97 | Wrong or no Outcome |
| Rotovnik Kozjek, Nada, Kompan, Lidija, Soeters, Peter, Oblak, Irena, Mlakar Mastnak, Denis, Mozina, Barbara, Zadnik, Vesna, Anderluh, Franc, Velenik, Vaneja. Oral glutamine supplementation during preoperative radiochemotherapy in patients with rectal cancer: a randomised double blinded, placebo controlled pilot study. Clinical nutrition (Edinburgh, Scotland). 2011. 30:567-70 | Single Risk Factor Management |
| Dao, Tam K., Youssef, Nagy A., Armsworth, Mary, Wear, Emily, Papathopoulos, Katina N., Gopaldas, Raja. Randomized controlled trial of brief cognitive behavioral intervention for depression and anxiety symptoms preoperatively in patients undergoing coronary artery bypass graft surgery. The Journal of thoracic and cardiovascular surgery. 2011. 142:e109-15 | Wrong or Unclear Duration |
| Swank, Ann M., Kachelman, Joseph B., Bibeau, Wendy, Quesada, Peter M., Nyland, John, Malkani, Arthur, Topp, Robert V.. Prehabilitation before total knee arthroplasty increases strength and function in older adults with severe osteoarthritis. Journal of strength and conditioning research. 2011. 25:318-25 | Wrong or no Outcome |
| Miettinen, T. A., Nissinen, M., Lepantalo, M., Alback, A., Railo, M., Vikatmaa, P., Kaste, M., Mustanoja, S., Gylling, H.. Non-cholesterol sterols in serum and endarterectomized carotid arteries after a short-term plant stanol and sterol ester challenge. Nutrition, metabolism, and cardiovascular diseases : NMCD. 2011. 21:182-8 | Wrong or no Outcome |
| Gomez Sanchez, Ma B., Garcia-Talavera Espin, N. V., Sanchez Alvarez, C., Zomeno Ros, A. I., Hernandez, M. Nicolas, Gomez Ramos, Ma J., Parra Banos, P., Gonzalez Valverde, F. M.. [Perioperative nutritional support in patients with colorectal neoplasms]. Apoyo nutricional perioperatorio en pacientes con neoplasia colorrectal.. 2010. 25:797-805 | Not a Randomized Trial |
| Felekis, Dimitrios, Eleftheriadou, Anna, Papadakos, Georgios, Bosinakou, Irini, Ferekidou, Eliza, Kandiloros, Dimitrios, Katsaragakis, Stylianos, Charalabopoulos, Konstantinos, Manolopoulos, Leonidas. Effect of perioperative immuno-enhanced enteral nutrition on inflammatory response, nutritional status, and outcomes in head and neck cancer patients undergoing major surgery. Nutrition and cancer. 2010. 62:1105-12 | Wrong or Unclear Duration |
| Liu, Yan, Tao, Kai-xiong, Wang, Guo-bin. [Effects of perioperative total parenteral nutrition support on cyclin D1 expression, recurrence and metastasis of colorectal cancer cells]. Zhonghua wei chang wai ke za zhi = Chinese journal of gastrointestinal surgery. 2010. 13:433-5 | Wrong or no Outcome |
| Horvat, Matjaz, Krebs, Bojan, Potrc, Stojan, Ivanecz, Arpad, Kompan, Lidija. Preoperative synbiotic bowel conditioning for elective colorectal surgery. Wiener klinische Wochenschrift. 2010. 122 Suppl 2:26-30 | Wrong or Unclear Duration |
| Botella-Carretero, Jose I., Iglesias, Borja, Balsa, Jose A., Arrieta, Francisco, Zamarron, Isabel, Vazquez, Clotilde. Perioperative oral nutritional supplements in normally or mildly undernourished geriatric patients submitted to surgery for hip fracture: a randomized clinical trial. Clinical nutrition (Edinburgh, Scotland). 2010. 29:574-9 | Wrong or Unclear Duration |
| Hartigan, Erin H., Axe, Michael J., Snyder-Mackler, Lynn. Time line for noncopers to pass return-to-sports criteria after anterior cruciate ligament reconstruction. The Journal of orthopaedic and sports physical therapy. 2010. 40:141-54 | Wrong Population |
| Garbossa, Aline, Maldaner, Emilia, Mortari, Daiana Moreira, Biasi, Janaina, Leguisamo, Camila Pereira. Effects of physiotherapeutic instructions on anxiety of CABG patients. Revista brasileira de cirurgia cardiovascular : orgao oficial da Sociedade Brasileira de Cirurgia Cardiovascular. 2009. 24:359-66 | Wrong or Unclear Duration |
| Asprer, Jonathan M., Llido, Luisito O., Sinamban, Reynaldo, Schlotzer, Ewald, Kulkarni, Hrishikesh. Effect on immune indices of preoperative intravenous glutamine dipeptide supplementation in malnourished abdominal surgery patients in the preoperative and postoperative periods. Nutrition (Burbank, Los Angeles County, Calif.). 2009. 25:920-5 | Wrong or Unclear Duration |
| Gill, Stephen D., McBurney, Helen, Schulz, Debra L.. Land-based versus pool-based exercise for people awaiting joint replacement surgery of the hip or knee: results of a randomized controlled trial. Archives of physical medicine and rehabilitation. 2009. 90:388-94 | Duplicate |
| Schenk, Jeannette M., Neuhouser, Marian L., Lin, Daniel W., Kristal, Alan R.. A dietary intervention to elicit rapid and complex dietary changes for studies investigating the effects of diet on tissues collected during invasive surgical procedures. Journal of the American Dietetic Association. 2009. 109:459-63 | No Results |
| Kim, Do Jun, Mayo, Nancy E., Carli, Franco, Montgomery, David L., Zavorsky, Gerald S.. Responsive measures to prehabilitation in patients undergoing bowel resection surgery. The Tohoku journal of experimental medicine. 2009. 217:109-15 | Wrong or no Outcome |
| Liu, Jin-Yang, Li, Kang-Hua, Hu, Jian-Zhong, Zhang, Hong-Qi. [A controlled clinical trail of perioperative nutritional support of thoracolumbar spinal tuberculosis]. Zhongguo gu shang = China journal of orthopaedics and traumatology. 2008. 21:28-9 | Not Prehabilitation |
| Demark-Wahnefried, Wendy, Polascik, Thomas J., George, Stephen L., Switzer, Boyd R., Madden, John F., Ruffin, Mack T. th, Snyder, Denise C., Owzar, Kouros, Hars, Vera, Albala, David M., Walther, Philip J., Robertson, Cary N., Moul, Judd W., Dunn, Barbara K., Brenner, Dean, Minasian, Lori, Stella, Philip, Vollmer, Robin T.. Flaxseed supplementation (not dietary fat restriction) reduces prostate cancer proliferation rates in men presurgery. Cancer epidemiology, biomarkers & prevention : a publication of the American Association for Cancer Research, cosponsored by the American Society of Preventive Oncology. 2008. 17:3577-87 | Wrong or no Outcome |
| Tobia, Ignacio, Gonzalez, Mariano S., Martinez, Pablo, Tejerizo, Juan C., Gueglio, Guillermo, Damia, Oscar, Marti, Maria I., Giudice, Carlos A.. [Randomized study on urinary continence after radical prostatectomy with previous kinesic perineal physiotherapy]. Estudio randomizado sobre continencia urinaria postprostatectomia radical con rehabilitacion perineal kiesica previa.. 2008. 61:793-8 | Wrong or Unclear Duration |
| Vukomanovic, Aleksandra, Popovic, Zoran, Durovic, Aleksandar, Krstic, Ljiljana. The effects of short-term preoperative physical therapy and education on early functional recovery of patients younger than 70 undergoing total hip arthroplasty. Vojnosanitetski pregled. 2008. 65:291-7 | Wrong or Unclear Duration |
| Back, Maria, Wennerblom, Bertil, Wittboldt, Susanna, Cider, Asa. Effects of high frequency exercise in patients before and after elective percutaneous coronary intervention. European journal of cardiovascular nursing : journal of the Working Group on Cardiovascular Nursing of the European Society of Cardiology. 2008. 7:307-13 | Wrong Population |
| Hulzebos, E. H. J., Helders, P. J. M., Favie, N. J., de Bie, R. A., Brutel de la Riviere, A., van Meeteren, N. L. U.. [Fewer lung complications following inspiratory muscle training in patients undergoing coronary bypass surgery: a randomized trial]. Minder longcomplicaties door ademspiertraining bij patienten die een coronaire bypassoperatie moeten ondergaan: een gerandomiseerde trial.. 2007. 151:2505-11 | Duplicate |
| Pacelli, Fabio, Bossola, Maurizio, Teodori, Laura, Trinca, Maria Luisa, Tortorelli, Antonio, Rosa, Fausto, Doglietto, Giovan Battista. Parenteral nutrition does not stimulate tumor proliferation in malnourished gastric cancer patients. JPEN. Journal of parenteral and enteral nutrition. 2007. 31:451-5 | Intervention Not Preoperative |
| Pour, Aidin Eslam, Parvizi, Javad, Sharkey, Peter F., Hozack, William J., Rothman, Richard H.. Minimally invasive hip arthroplasty: what role does patient preconditioning play?. The Journal of bone and joint surgery. American volume. 2007. 89:1920-7 | Intervention Not Preoperative |
| Pasternak, K., Dabrowski, W., Dobija, J., Wronskal, J., Rzecki, Z., Biernacka, J.. The effect of preoperative magnesium supplementation on blood catecholamine concentrations in patients undergoing CABG. Magnesium research. 2006. 19:113-22 | Wrong or Unclear Duration |
| Wu, Guo-Hao, Liu, Zhong-Hua, Wu, Zhao-Han, Wu, Zhao-Guang. Perioperative artificial nutrition in malnourished gastrointestinal cancer patients. World journal of gastroenterology. 2006. 12:2441-4 | Not Prehabilitation |
| Liao, Quan, Zhao, Yu-pei, Wang, Wei-bin, Dai, Meng-hua, Hu, Ya, Liu, Zi-wen, Zhu, Yu. [Perioperative nutrition support of the patients with pancreatic head cancer]. Zhongguo yi xue ke xue yuan xue bao. Acta Academiae Medicinae Sinicae. 2005. 27:579-82 | Not a Randomized Trial |
| Yao, Guo-Xiang, Wang, Xiu-Rong, Jiang, Zhu-Ming, Zhang, Si-Yuang, Ni, An-Ping. Role of perioperative parenteral nutrition in severely malnourished patients with Crohn's disease. World journal of gastroenterology. 2005. 11:5732-4 | Not Prehabilitation |
| Kim, Jeri, Sun, Peiyu, Lam, Ying-Wai, Troncoso, Patricia, Sabichi, Anita L., Babaian, Richard J., Pisters, Louis L., Pettaway, Curtis A., Wood, Christopher G., Lippman, Scott M., McDonnell, Timothy J., Lieberman, Ronald, Logothetis, Christopher, Ho, Shuk-Mei. Changes in serum proteomic patterns by presurgical alpha-tocopherol and L-selenomethionine supplementation in prostate cancer. Cancer epidemiology, biomarkers & prevention : a publication of the American Association for Cancer Research, cosponsored by the American Society of Preventive Oncology. 2005. 14:1697-702 | Wrong or no Outcome |
| Plank, Lindsay D., McCall, John L., Gane, Edward J., Rafique, Mohammad, Gillanders, Lynn K., McIlroy, Kerry, Munn, Stephen R.. Pre- and postoperative immunonutrition in patients undergoing liver transplantation: a pilot study of safety and efficacy. Clinical nutrition (Edinburgh, Scotland). 2005. 24:288-96 | Not a Randomized Trial |
| Charman, A., Muriithi, E. W., Milne, E., Wheatley, D. J., Armstrong, R. A., Belcher, P. R.. Fish oil before cardiac surgery: neutrophil activation is unaffected but myocardial damage is moderated. Prostaglandins, leukotrienes, and essential fatty acids. 2005. 72:257-65 | Wrong or no Outcome |
| Does preoperative hip rehabilitation advice improve recovery and patient satisfaction?. 2004. | Not Prehabilitation |
| Osinowo, H. O., Olley, B. O., Adejumo, A. O.. Evaluation of the effect of cognitive therapy on perioperative anxiety and depression among Nigerian surgical patients. West African journal of medicine. 2003. 22:338-42 | Wrong or Unclear Duration |
| Crowe, Jean, Henderson, Jennifer. Pre-arthroplasty rehabilitation is effective in reducing hospital stay. Canadian journal of occupational therapy. Revue canadienne d'ergotherapie. 2003. 70:88-96 | Intervention Not Preoperative |
| Heye, Mary L., Foster, Loretta, Bartlett, Mary Kay, Adkins, Sherry. A preoperative intervention for pain reduction, improved mobility, and self-efficacy. Applied nursing research : ANR. 2002. 15:174-83 | Wrong or Unclear Duration |
| Rohling, R. G., Zimmermann, A. P., Breymann, C.. Intravenous versus oral iron supplementation for preoperative stimulation of hemoglobin synthesis using recombinant human erythropoietin. Journal of hematotherapy & stem cell research. 2000. 9:497-500 | Not Prehabilitation |
| Omlor, G., Kiewitz, S., Pietschmann, S., Roesler, S.. [Effect of preoperative preoperative visualization therapy on postoperative outcome after inguinal hernia surgery and thyroid resection]. Einfluss einer praoperativen Visualisierungstherapie auf die postoperativen Ergebnisse nach Leistenherniotomie und Strumaresektion.. 2000. 125:380-6 | Wrong or Unclear Duration |
| Le Cornu, K. A., McKiernan, F. J., Kapadia, S. A., Neuberger, J. M.. A prospective randomized study of preoperative nutritional supplementation in patients awaiting elective orthotopic liver transplantation. Transplantation. 2000. 69:1364-9 | Wrong or Unclear Duration |
| Larson, M. R., Duberstein, P. R., Talbot, N. L., Caldwell, C., Moynihan, J. A.. A presurgical psychosocial intervention for breast cancer patients. psychological distress and the immune response. Journal of psychosomatic research. 2000. 48:187-94 | Wrong or Unclear Duration |
| Snyderman, C. H., Kachman, K., Molseed, L., Wagner, R., D'Amico, F., Bumpous, J., Rueger, R.. Reduced postoperative infections with an immune-enhancing nutritional supplement. The Laryngoscope. 1999. 109:915-21 | Wrong or Unclear Duration |
| McCarter, M. D., Gentilini, O. D., Gomez, M. E., Daly, J. M.. Preoperative oral supplement with immunonutrients in cancer patients. JPEN. Journal of parenteral and enteral nutrition. 1998. 22:206-11 | Wrong or no Outcome |
| Fagevik Olsen, M., Hahn, I., Nordgren, S., Lonroth, H., Lundholm, K.. Randomized controlled trial of prophylactic chest physiotherapy in major abdominal surgery. The British journal of surgery. 1997. 84:1535-8 | Intervention Not Preoperative |
| Westhuyzen, J., Cochrane, A. D., Tesar, P. J., Mau, T., Cross, D. B., Frenneaux, M. P., Khafagi, F. A., Fleming, S. J.. Effect of preoperative supplementation with alpha-tocopherol and ascorbic acid on myocardial injury in patients undergoing cardiac operations. The Journal of thoracic and cardiovascular surgery. 1997. 113:942-8 | Not Prehabilitation |
| Ziegler, T. R.. Perioperative nutritional support in patients undergoing hepatectomy for hepatocellular carcinoma. JPEN. Journal of parenteral and enteral nutrition. 1996. 20:91-2 | Not Prehabilitation |
| Von Meyenfeldt, M. F., Meijerink, W. J., Rouflart, M. M., Builmaassen, M. T., Soeters, P. B.. Perioperative nutritional support: a randomised clinical trial. Clinical nutrition (Edinburgh, Scotland). 1992. 11:180-6 | Not Prehabilitation |
| Dionigi, P., Jemos, V., Cebrelli, T., Ferrari, C., Ferrari, A., Berizzi, F., Mazzini, G.. Pre-operative nutritional support and tumour cell kinetics in malnourished patients with gastric cancer. Clinical nutrition (Edinburgh, Scotland). 1991. 10 Suppl:77-84 | Wrong or no Outcome |
| Zeiderman, M. R., Gowland, G., Peel, B., McMahon, M. J.. The influence of short-term pre-operative intravenous nutrition upon anthropometric variables, protein synthesis and immunological indices in patients with gastrointestinal cancer. Clinical nutrition (Edinburgh, Scotland). 1991. 10:213-21 | Not Prehabilitation |
| Sedman, P. C., MacFie, J., Palmer, M. D., Mitchell, C. J., Sagar, P. M.. Preoperative total parenteral nutrition is not associated with mucosal atrophy or bacterial translocation in humans. The British journal of surgery. 1995. 82:1663-7 | Not a Randomized Trial |
| Manyande, A., Berg, S., Gettins, D., Stanford, S. C., Mazhero, S., Marks, D. F., Salmon, P.. Preoperative rehearsal of active coping imagery influences subjective and hormonal responses to abdominal surgery. Psychosomatic medicine. 1995. 57:177-82 | Wrong or Unclear Duration |
| Wijgman, A. J., Dekkers, G. H., Waltje, E., Krekels, T., Arens, H. J.. [No positive effect of preoperative exercise therapy and teaching in patients to be subjected to hip arthroplasty]. Geen positief effect van preoperatieve oefentherapie en instructie bij patienten die heupartroplastiek zullen ondergaan.. 1994. 138:949-52 | Not a Randomized Trial |
| Fan, S. T., Lo, C. M., Lai, E. C., Chu, K. M., Liu, C. L., Wong, J.. Perioperative nutritional support in patients undergoing hepatectomy for hepatocellular carcinoma. The New England journal of medicine. 1994. 331:1547-52 | Not Prehabilitation |
| Croog, S. H., Baume, R. M., Nalbandian, J.. Pain response after psychological preparation for repeated periodontal surgery. Journal of the American Dental Association (1939). 1994. 125:1353-60 | Not a Randomized Trial |
| Nilsen, D. W., Almdahl, S. M., Svensson, B., Vaage, J., Rasmussen, K., Osterud, B.. Lipopolysaccharide induced monocyte thromboplastin synthesis and coagulation responses in patients undergoing coronary bypass surgery after preoperative supplementation with n-3 fatty acids. Thrombosis and haemostasis. 1993. 70:900-2 | Not Prehabilitation |
| Rice, V. H., Mullin, M. H., Jarosz, P.. Preadmission self-instruction effects on postadmission and postoperative indicators in CABG patients: partial replication and extension. Research in nursing & health. 1992. 15:253-9 | Wrong or Unclear Duration |
| Crawford, B. L., Blunnie, W. P., Elliott, A. G.. The value of self-administered peri-operative physiotherapy. Irish journal of medical science. 1990. 159:51-2 | Wrong or Unclear Duration |
| Zeiderman, M. R., King, R. F., Young, G. A., McMahon, M. J.. Metabolic changes in human liver associated with preoperative intravenous nutrition. Clinical science (London, England : 1979). 1989. 77:343-9 | Wrong or Unclear Duration |
| He, G. Z.. [The effect of enriched branched chain amino acid solution on amino acid metabolism during perioperative total parenteral nutrition support]. Zhonghua wai ke za zhi [Chinese journal of surgery]. 1989. 27:756-782 | Wrong or Unclear Duration |
| Bellantone, R., Doglietto, G. B., Bossola, M., Pacelli, F., Negro, F., Sofo, L., Crucitti, F.. Preoperative parenteral nutrition in the high risk surgical patient. JPEN. Journal of parenteral and enteral nutrition. 1988. 12:195-7 | Not Prehabilitation |
| Sculati, O., Giampiccoli, G., Gozzi, B., Minissale, V., Zambetti, N., Iapichino, G., Ipezzoli, C., Giacomelli, M., Lazzari, P., Franzosi, M. G.. Bran diet for an earlier resolution of post-operative ileus. The Journal of international medical research. 1982. 10:194-7 | Not a Randomized Trial |
| Muller, J. M., Brenner, U., Dienst, C., Pichlmaier, H.. Preoperative parenteral feeding in patients with gastrointestinal carcinoma. Lancet (London, England). 1982. 1:68-71 | Not Prehabilitation |
| Gurry, J. F., Ellis-pegler, R. B.. An elemental diet as preoperative preparation of the colon. The British journal of surgery. 1976. 63:969-72 | Wrong or Unclear Duration |
| Tomlinson, R. J., Newman, B. M., Schofield, P. F.. Is colostomy closure a hazardous procedure? A comparison of elemental diet and routine bowel preparation. The British journal of surgery. 1976. 63:799-800 | Wrong or Unclear Duration |
| Nichols, P. J., Howell, B.. Routine pre-and post-operative physiotherapy. A preliminary trial. Physiotherapy. 1970. 56:356-9 | Wrong or Unclear Duration |
| Boright, Lori Elizabeth. Development and pilot results of a cancer prehabilitation study protocol for patients diagnosed with head and neck cancer. Dissertation Abstracts International: Section B: The Sciences and Engineering. 2020. 81:No-Specified | Not a Randomized Trial |
| Barrett-Bernstein, Meagan, Carli, Francesco, Gamsa, Ann, Scheede-Bergdahl, Celena, Minnella, Enrico, Ramanakumar, Agnihotram V., Tourian, Leon. Depression and functional status in colorectal cancer patients awaiting surgery: Impact of a multimodal prehabilitation program. Health Psychology. 2019. 38:900-909 | Not a Randomized Trial |
| Heikkinen, Katja, Helena, Leino-Kilpi, Taina, Nummela, Anne, Kaljonen, Sanna, Salantera. A comparison of two educational interventions for the cognitive empowerment of ambulatory orthopaedic surgery patients. Patient Education and Counseling. 2008. 73:272-279 | Not Prehabilitation |
| Mingyan, Du, Fuyuan, Liu. Effect of psychological intervention on mental state of surgical patients with primary angle-closure glaucoma. Chinese Mental Health Journal. 2001. 15:412-414 | Intervention Not Preoperative |
| Wall, Lisa Marie. An exploration of hope and power among lung cancer patients who have and have not participated in a preoperative exercise program. Dissertation Abstracts International: Section B: The Sciences and Engineering. 1999. 60:0133 | Not a Randomized Trial |
| Grundner, R., Gotz-Frei, M. L., Huber, H. P., Kurz, R., Sauer, H.. Psychological preparation for surgery among 48 year olds. Psychologische Operationsvorbereitung bei 4-8jahrigen.. 1988. 37:34-38 | Wrong Population |
| Lobb, Michael L., Shannon, Mary C., Rrecer, Sara L., Allen, Janice B.. A behavioral technique for recovery from the psychological trauma of hysterectomy. Perceptual and Motor Skills. 1984. 59:677-678 | Not a Randomized Trial |
| Ridgeway, Valerie, Mathews, Andrew. Psychological preparation for surgery: A comparison of methods. British Journal of Clinical Psychology. 1982. 21:271-280 | Not a Randomized Trial |
| Shukla, G. D., Srivastava, R. P.. Pre-operative anxiety and its management in dentistry. Indian Journal of Psychiatry. 1981. 23:254-255 | Wrong or Unclear Duration |
| Maddison R, Prapavessis H. Clatworthy M.. Modeling and rehabilitation following anterior cruciate ligament reconstruction. Annals of behavioral medicine. 2006. 31:89 | Wrong or Unclear Duration |
| Guo Jc, Li J. Hu Y. Zhang T. P. Liao Q. Dai M. H. Zhao Y. P.. The role of perioperative enteral and parenteral nutrition treatment in pancreatic cancer: a multicenter, prospective randomized controlled trial. Zhonghua wai ke za zhi [Chinese journal of surgery]. 2013. 51:987 | Not Prehabilitation |
| Chakravartty S, Vivian G. Mullholland N. Shaikh H. McGrath J. Sidhu P. S. Jaffer O. Patel A. G.. Preoperative liver shrinking diet for bariatric surgery may impact wound healing: a randomized controlled trial. Surgery for obesity and related diseases. 2018. (no pagination): | Duplicate |
| Xu B, Xu W. X. Lao Y. J. Ding W. G. Lu D. Sheng H. F.. Multimodal Nutritional Management in Primary Lumbar Spine Surgery: a Randomized Controlled Trial. Spine. 2019. 44:967 | Wrong or Unclear Duration |
| Thornes E, Stendal Robinson H. Moosmayer S. Ekeland A. Vollestad N. K.. Low-impact exercise program for patients with symptomatic lumbar spinal stenosis awaiting surgery: a controlled pilot study. Low-impact exercise program for patients with symptomatic lumbar spinal stenosis awaiting surgery: a controlled pilot study. 2019. | Wrong or no Outcome |
| Hruba S, Chovanec M. Cada Z. Balatkova Z. Fik Z. Slaby K. Zverina E. Betka J. Plzak J. Cakrt O.. The evaluation of vestibular compensation by vestibular rehabilitation and prehabilitation in short-term postsurgical period in patients following surgical treatment of vestibular schwannoma. The evaluation of vestibular compensation by vestibular rehabilitation and prehabilitation in short-term postsurgical period in patients following surgical treatment of vestibular schwannoma. 2019. | Single Risk Factor Management |
| Argunova Y, Korotkevich A. Belik E. Pomeshkina S. Barbarash O.. Efficacy and safety of prehabilitation before coronary artery bypass grafting. Efficacy and safety of prehabilitation before coronary artery bypass grafting. 2019. 40:721 | Not a Randomized Trial |
| . Erratum: effect of multimodal prehabilitation vs postoperative rehabilitation on 30-day postoperative complications for frail patients undergoing resection of colorectal cancer: a randomized clinical trial (JAMA Surgery (2020) DOI: 10.1001/jamasurg.2019.5474). Erratum: effect of multimodal prehabilitation vs postoperative rehabilitation on 30-day postoperative complications for frail patients undergoing resection of colorectal cancer: a randomized clinical trial (JAMA Surgery (2020) DOI: 10.1001/jamasurg.2019.5474). 2020. 155:269 | Not a Randomized Trial |
| Chang X-K, Xu R. Zheng Y. J. Liu X. Wang Y. Jiao Y.. Effect of preoperative meibomian gland physiotherapy on ocular surface of patients with meibomian gland dysfunction after cataract surgery. Effect of preoperative meibomian gland physiotherapy on ocular surface of patients with meibomian gland dysfunction after cataract surgery. 2020. 20:1422 | Intervention Not Preoperative |
| Nct. "Impact of Pelvic Floor Prehabilitation Using Biofeedback on the Severity of the Low Anterior Resection Syndrome in Patients Undergoing a Total Mesorectal Excision for Rectal Cancer". "Impact of Pelvic Floor Prehabilitation Using Biofeedback on the Severity of the Low Anterior Resection Syndrome in Patients Undergoing a Total Mesorectal Excision for Rectal Cancer". 2019. | Not a Randomized Trial |
| Isrctn. The feasibility of performing a walking programme in patients with rectal cancer undergoing chemo-radiotherapy (the REx trial). A pilot study of the feasibility and patient-related outcomes of performing a walking intervention in patients undergoing treatment for rectal cancer (the REx trial). 2014. | Protocol |
| Humeidan Ml, Reyes J. P. C. Mavarez-Martinez A. Roeth C. Nguyen C. M. Sheridan E. Zuleta-Alarcon A. Otey A. Abdel-Rasoul M. Bergese S. D.. Effect of Cognitive Prehabilitation on the Incidence of Postoperative Delirium among Older Adults Undergoing Major Noncardiac Surgery: the Neurobics Randomized Clinical Trial. JAMA surgery. 2020. | Duplicate |
| Bernardi K, Olavarria O. A. Dhanani N. H. Lyons N. Holihan J. L. Cherla D. V. Berger D. H. Ko T. C. Kao L. S. Liang M. K.. Two Year Outcomes of Prehabilitation Among Obese Patients with Ventral Hernias: a Randomized Controlled Trial (NCT02365194). Annals of surgery. 2020. | Duplicate |
| Carvalho T, Bonorino K. C. Panigas T. F.. Preoperative respiratory muscle training reduces complications in coronary artery bypass surgery. European heart journal. 2011. 32 Suppl:328 | Unregistered Abstract |
| George J, Whyte M. Scott M. Rockall T.. Effect of 4-week multimodal prehabilitation on fitness before pancreatic resection: an interventional pilot study. HPB. 2020. 22:S267 | Not a Randomized Trial |
| Gruber Es, Hasenoehrl T. Sahora K. Tamandl D. Jomrich G. Prager G. Schmid R. Wagner E. Karin M. Crevenna R. Schindl M.. Impact of an individual pre-habilitation program on body composition, physical fitness and perioperative outcome in patients with pancreatic ductal adenocarcinoma undergoing neoadjuvant treatment. HPB. 2020. 22:S385 | Protocol |
| Nct. Preoperative Psychosocial Support for Postoperative Recovery and Health. Preoperative Psychosocial Support for Postoperative Recovery and Health. 2020. | No Results |
| Edwards J, Moug S. Barry S.. Does pre-habilitation, in the form of a walking programme, impact upon levels of sarcopenia (low muscle mass) in patients with rectal cancer undergoing neo-adjuvant chemoradiotherapy?. Anaesthesia. 2020. 75:79 | Wrong or no Outcome |
| Salik Sengul Y, Kaya N. Yalcinkaya G. Kirmizi M. Kalemci O.. The effects of the addition of motor imagery to home exercises on pain, disability and psychosocial parameters in patients undergoing lumbar spinal surgery: a randomized controlled trial. Explore (New York, N.Y.). 2020. | Intervention Not Preoperative |
| Shi Z-X, Kang K. Shu X. L.. Effects of individualized nutritional therapy on nutritional status of patients in perioperative period of digestive system operation. Academic journal of second military medical university. 2020. 41:24 | Wrong or Unclear Duration |
| Ruiz-Tovar J, Blanca M. Garcia A. Gonzalez J. Gutierrez S. Paniagua A. Prieto M. J. Ramallo L. Llanos L. Duran M.. Preoperative administration of Omega-3 fatty acids on postoperative pain and acute-phase reactants in patients undergoing Roux-en-Y gastric bypass: a randomized clinical trial. Clinical nutrition (Edinburgh, Scotland). 2018. | Duplicate |
| Nct. A Preoperative Cognitive Behavioural Therapy Program Based on Self-determination Theory for Bariatric Surgery Candidates. Evaluation of a Preoperative Cognitive Behavioural Therapy (CBT) Program Based on Self-determination Theory for Bariatric Surgery Candidates : an Open-label Controlled, Randomized, Superiority Study. 2019. | No Results |
| Nct. The Effects of Preoperative and Postoperative Oral Nutritional Supplements in Malnourished Post-gastrectomy Patients. A Prospective Randomized Controlled Trial Evaluating the Effects of Preoperative and Postoperative Oral Nutritional Supplements (ONS) in Malnourished Post-gastrectomy Patients. 2011. | Intervention Not Preoperative |
| Nct. Pre-habilitation Program for Elective Coronary Artery Bypass Graft Surgery Patients. Pre-habilitation Program for Elective Coronary Artery Bypass Graft Surgery Patients. 2013. | Duplicate |
| Nct. Effect of Preoperative Exercise in Patients Undergoing Total Knee Arthroplasty. Effect of Preoperative Exercise in Patients Undergoing Total Knee Arthroplasty: a Randomized Controlled Pilot Trial. 2017. | Protocol |
| Nct. Effect of a Perioperative Oral Nutritional Supplementation on Patients Undergoing Hepatic Surgery for Liver Cancer. Prospective, Randomized, Double Blind, Placebo-Controlled Study Evaluating the Effect of Oral Immune-Enhancing Nutritional Supplement on Hepatic Function After Liver Resection for Primary or Secondary Cancer on Cirrhosis or Liver Fibrosis. 2005. | Protocol |
| Nct. Immunonutrition Versus Standard Enteral Nutrition Before Major Surgery. Immunonutrition Versus Standard Enteral Nutrition Before Major Surgery: a Single-center Double-blinded Controlled Randomized Superiority Trial. 2007. | Wrong or Unclear Duration |
| Nct. Perioperative Immunonutrition, Phagocytic and Bactericidal Activity of Blood Platelets in Gastric Cancer Patients. Prospective Study of the Effect of Perioperative Immunonutrition on the Immune Host Defense and the Phagocytic and Bactericidal Activity of Blood Platelets in Gastric Cancer Patients. 2012. | Protocol |
| Nct. Impact of Prehabilitation in Total Knee Replacement. Impact of Prehabilitation in Total Knee Arthroplasty: outcomes and Healthcare Service Utilization. 2013. | No Results |
| Nct. Impact of Presurgical Exercise on Hemodialysis Fistula Outcomes. The Impact of Exercise on Vascular Remodeling, Arteriovenous Fistula Creation and Use in Patients With Chronic Kidney Disease: a Randomized Controlled Trial. 2014. | No Results |
| Nct. Using Multimodal Prehabilitation to Improve Outcomes for Frail Patients Undergoing Resection of Colorectal Cancer. https://clinicaltrials.gov/show/NCT02502760. 2015. | No Results |
| Nct. Neuromuscular Electrical Stimulation (NMES) for Improving Outcomes Following Total Knee Arthroplasty (TKA). Prospective Evaluation of Neuromuscular Electrical Stimulation (NMES) for Improving Outcomes Following Total Knee Arthroplasty (TKA). 2016. | Not Prehabilitation |
| Ip Wtk, Chandramouli C. Smith J. A. McLennan P. L. Pepe S. Delbridge L. M. D.. A Small Cohort Omega-3 PUFA Supplement Study: implications of Stratifying According to Lipid Membrane Incorporation in Cardiac Surgical Patients. Heart lung and circulation. (no pagination). 2017. Date of Publication: September 16: | Single Risk Factor Management |
| Ligibel Ja, Giobbie-Hurder A. Dillion D. Shockro L. Campbell N. Rhei E. Troyan S. Dominici L. Golshan M. Chagpar A. Yung R. Freedman R. Tolaney S. Winer E. Frank E. McTiernan A. Irwin M.. Impact of pre-operative exercise and mind-body interventions on patient-reported outcomes in women with newly diagnosed breast cancer. Cancer research. 2017. 77: | Duplicate |
| Laurent H, Aubreton S. Galvaing G. Pereira B. Merle P. Richard R. Costes F. Filaire M.. Preoperative respiratory muscle endurance training improves ventilatory capacity and prevents pulmonary postoperative complications after lung surgery: a randomized controlled trial. European journal of physical and rehabilitation medicine. 2019. | Duplicate |
| Yulia Argunova Y, Pomeshkina S. A. Kokov A. N. Barbarash O. L.. Cardioprotective effects of exercise trainings prior to coronary artery bypass grafting. European journal of preventive cardiology. 2019. 26:S37 | Duplicate |
| Minnella Em, Awasthi R. Bousquet-Dion G. Ferreira V. Austin B. Audi C. Tanguay S. Aprikian A. Carli F. Kassouf W.. Multimodal Prehabilitation to Enhance Functional Capacity Following Radical Cystectomy: a Randomized Controlled Trial. European urology focus. 2019. | Wrong or Unclear Duration |
| Hulzebos Ehj, Helders P. J. M. Favie N. J. Bie de R. A. Brutel de la Riviere A., Meeteren van, N. L. U.. Preoperative intensive inspiratory muscle training to prevent postoperative pulmonary complications in high-risk patients undergoing CABG surgery: a randomized clinical trial. Preventie van postoperatieve pulmonale complicaties door intensieve preoperatieve ademspiertraining bij hoogrisicopatienten die een bypassoperatie moeten ondergaan: een gerandomiseerde klinische trial. 2007. 117:2 | Duplicate |
| Nolan F, Lyon K. Lambie N.. Initial results: the effect of a physiotherapy prehabilitation programme on postoperative outcomes in patients undergoing cardiac or thoracic surgery. Clinical nutrition ESPEN. 2019. 31:111 | Wrong or Unclear Duration |
| Maguire S, Kinsella J. Steele R. Mutrie N. Anderson A. Moug S.. Does prehabilitation modify muscle wasting in patients with rectal cancer undergoing neoadjuvant therapy?. Colorectal disease. 2018. 20:9 | Wrong or no Outcome |
| Sheng B, Chen W. Zhao L.. Efficacy of perioperative enteral nutrition in management of hepatocellular carcinoma with cirrhosis. World chinese journal of digestology. 2013. 21:2999 | Wrong or Unclear Duration |
| Wang Z-Y, Wu X. Q.. Preoperative psychological nursing intervention promotes recovery of patients with esophageal cancer. World chinese journal of digestology. 2014. :2905 | Wrong or Unclear Duration |
| Lidder Pg, Thomas S. Fleming S. C. Hosie K. B. Lewis S. J.. Nutritional intervention in patients undergoing colorectal surgery: support for the routine prescription of oral nutritional supplements pre-and post-operatively. Gut. 2010. 59:A35 | Unregistered Abstract |
| Garcia Rs, Paz A. L. Brage M. I. Y. Moolhuyzen E. G. Rioboo M. S. Mate J. M. B.. Preliminary efficacy of preoperative exercise training in patients with lung malignancies undergoing VATS. European respiratory journal. 2016. 48: | Wrong or no Outcome |
| Kwok K, Hardy K. Bouchard D. Vergis A.. The impact of a pre-operative exercise program on patients awaiting bariatric surgery. Surgery for obesity and related diseases. 2016. 12:S201 | Wrong or no Outcome |
| Dayucos A, French L. A. Kelemen A. Liang Y. Sik Lanyi C.. Creation and Evaluation of a Preoperative Education Website for Hip and Knee Replacement Patients-A Pilot Study. Medicina (kaunas, lithuania). 2019. 55: | Not Prehabilitation |
| Wang H-X, Xia Y. Shao S. Y.. Influence of enteral nutrition during the preoperative and postoperative periods on postoperative nutritional status and immunologic function in patients with gastric cancer. Journal of xi'an jiaotong university (medical sciences). 2011. 32:375 | Wrong or Unclear Duration |
| Hjelmesaeth J, Rosenvinge J. H. Gade H. Friborg O.. Effects of Cognitive Behavioral Therapy on Eating Behaviors, Affective Symptoms, and Weight Loss After Bariatric Surgery: a Randomized Clinical Trial. Obesity surgery. 2018. | Duplicate |
| Hiroshima Y, Yabushita Y. Kikuchi Y. Kawagushi D. Murakami T. Ichikawa Y. Endo I. Tanaka K.. Preoperative supplementation of branched-chain amino acids suppresses Osteopontin expression in patients with hepatocellular carcinoma. Cancer research. 2018. 78: | Wrong or Unclear Duration |
| Salzmann S, Laferton J. Auer C. Shedden-Mora M. Wambach K. Rief W.. Optimizing Patients&#039; Expectations: description of a Brief Preoperative Intervention for Patients Undergoing Coronary Artery Bypass Graft Surgery. Patientenerwartungen optimieren: beschreibung einer praoperativen Kurzintervention am Beispiel von Patienten vor einer Bypass-Operation. 2018. 28:157 | Wrong or Unclear Duration |
| Moug S, Rooney L. Mackay G. Barry S. Buchan C. Steele R. Anderson A. Mutrie N.. Initial findings of the REx Trial: a study of the feasibility of performing pre-habilitation in patients undergoing treatment for rectal cancer. Colorectal disease. 2016. 18:75 | Wrong or no Outcome |
| Jing L-S, Feng J. Tang F. M. Ma Y. Wang H. Tan H. M.. Comparison of effect of preoperative informational support system and normal psychological nursing intervention on the terrified anxiety of surgery patients. Chinese journal of clinical rehabilitation. 2004. 8:3478 | Wrong or Unclear Duration |
| Zi Y, Li Z. Shang Y.. Effect of preoperative psychological intervention on the myocardial oxygen consumption in perioperative patients under decompression of spinal cord. Chinese journal of clinical rehabilitation. 2005. 9:40 | Wrong or Unclear Duration |
| Wu W, Lin S. Wu G. J. Li L.. Influence of perioperative supportive psychotherapy on the postoperative mental state and sexual life in patients with uterine cervix cancer. Chinese journal of clinical rehabilitation. 2005. 9:42 | Not Prehabilitation |
| Ligibel Ja, Giobbie-Hurder A. Shockro L. Rhei E. Troyan S. Dominici L. S. Chagpar A. B. Frank E. S. McTiernan A. Yung R. L. Freedman R. A. Tolaney S. M. O'Connor K. Stecker K. Dillon D. Irwin M. L.. Impact of a pre-operative exercise intervention on Ki-67 and metabolic markers in women with early breast cancer. Journal of clinical oncology. 2016. 34: | Duplicate |
| Gorecki P, Burke D. L. Chapple I. L. C. Hemming K. Saund D. Pearson D. Stahl W. Lello R. Dietrich T.. Perioperative supplementation with a fruit and vegetable juice powder concentrate and postsurgical morbidity: a double-blind, randomised, placebo-controlled clinical trial. Clinical nutrition (edinburgh, scotland). 2017. (no pagination): | Duplicate |
| Palma-Milla S, Lopez-Plaza B. Santamaria B. de Arriba-Sanchez A. Bermejo L. M. Gomez-Candela C.. New, immunomodulatory, oral nutrition formula for use prior to surgery in patients with head and neck cancer: an exploratory study. Jpen: journal of parenteral & enteral nutrition. 2016. | Duplicate |
| Ng, S. I.. A Randomized Controlled Trial Study of the Efficacy of Intensive Preoperative Pelvic Floor Muscle Training to Decrease Post-prostatectomy Urinary Incontinence (Trials registry number: nCT01338584). Clinicaltrials.gov (http://clinicaltrials.gov/show/nct01338584). 2011. | Protocol |
| Nct, Mina D. S. Matthew A. G. Carli F.. A Multicentre, Pilot Randomized Controlled Trial to Examine the Effects of Prehabilitation on Functional Outcomes After Radical Prostatectomy. Http://clinicaltrials.gov/show/nct02036684. 2013. | No Results |
| Voorham-Van Der Zalm Pj, Stoetman A. M. Putter H. Bevers R. F. M. Pelger R. C. M.. Effect of preoperative pelvic floor physiotherapy versus standard care on incontinence in men undergoing radical laparascopic prostatectomy: an ongoing study (Abstract number 590). Proceedings of the joint meeting of the international continence society (ICS) and the international urogynecological association. 2010. #volume#:#pages# | Wrong or Unclear Duration |
| Tang Y, Wu X. S. Wei B. Chen L. Li R.. Clinical application of perioperative fast-track and nutrition support program in elderly patients with gastric cancer. Chinese journal of clinical nutrition. 2010. 18:137 | Wrong or Unclear Duration |
| Schmidt M, Eckardt R. Scholtz K. Neuner B. Von Dossow-Hanfstingl V. Sehouli J. Stief C. G. Wernecke K. D. Spies C. D.. Patient empowerment improved Perioperative quality of care in cancer patients aged > 65 Years - a randomized controlled trial. Plos one. 2015. 10: | Wrong or Unclear Duration |
| van Stijn Mfm, Soeters M. R. van Leeuwen P. A. M. Schreurs W. H. Schoorl M. G. Twisk J. W. R. De Bandt J. P. Bonnefont-Rousselot D. Cynober L. Ackermans M. T. Serlie M. J. Houdijk A. P. J.. Effects of a Carbohydrate-, Glutamine-, and Antioxidant-Enriched Oral Nutrition Supplement on Major Surgery-Induced Insulin Resistance: a Randomized Pilot Study. JPEN. Journal of parenteral and enteral nutrition. 2017. (no pagination): | Wrong or Unclear Duration |
| Wang Z-D, Peng J. S. Chen S. Huang Z. M. Huang L.. Effects of perioperative enteral immunonutrition on nutritional status, immunity and inflammatory response of elderly patiens. National medical journal of china. 2006. 86:1410 | Wrong or Unclear Duration |
| Jiang Y-J, Kong X. J. Cheng G. Tian Z. B.. Effects of reasonable preoperative nutrition on recoveries of gastrointestinal cancer patients. World chinese journal of digestology. 2006. 14:1928 | Not Prehabilitation |
| Cheesman E, Ray C. Jones E. Baum M.. A controlled trial of preoperative counselling for women undergoing breast surgery. Clinical oncology. 1979. 5:194 | Not Prehabilitation |
| Frawley Hc, Galea M. Phillips B. Bo K.. The effect of a physiotherapy exercise program on bladder, prolapse and bowel outcomes in women undergoing gynaecological surgery: an assessor-blinded randomised controlled trial (Abstract number 46). Neurourology and urodynamics. 2008. 27:623 | Not Prehabilitation |
| Futter C, Weiler-Mithoff E. Hagen S. Blondeel P. N.. A randomised conrolled trial investigating the effect of pre-operative abdominal exercises on post-operative abdominal muscle strength of women undergoing breast reconstruction with a TRAM or DIEP flap. Personal communication. 2001. | Duplicate |
| Lim Stk, Choa R. G. Lam K. H., et al.. Total parenteral nutrition versus gastrotomy in the preoperative preparation of patients with carcinoma of the oesophagus. BR. J. SURG. 1981. 68:69 | Not Prehabilitation |
| Veen H, Meyerink W. J. Buil-Maassen M. T. Soeters P. B. von Meyenfeldt M. F.. Perioperative nutritional support: a prospective randomized trial. Clin-nutr. 1989. 8 Spec Suppl:58 | Not Prehabilitation |
| Pfirrmann D, Tug S. Brosteanu O. Mehdorn M. Busse M. Grimminger P. P. Lordick F. Glatz T. Hoeppner J. Lang H. Simon P. Gockel I.. Internet-based perioperative exercise program in patients with Barrett's carcinoma scheduled for esophagectomy a prospective randomizedcontrolled trial. BMC cancer. 2017. 17: | Protocol |
| Camolas J, Santos O. Moreira P. do Carmo I.. INDIVIDUO: results from a patient-centered lifestyle intervention for obesity surgery candidates. Obesity research and clinical practice. (no pagination). 2016. Date of Publication: January 29: | Wrong or no Outcome |
| Marcon Er, Baglioni S. Bittencourt L. Lopes C. L. N. Neumann C. R. Trindade M. R. M.. What Is the Best Treatment before Bariatric Surgery? Exercise, Exercise and Group Therapy, or Conventional Waiting: a Randomized Controlled Trial. Obesity surgery. 2016. :1 | Wrong or no Outcome |
| Perioperative rehabilitation in operations for lung cancer - A feasibility study (PROLUCA). 2015. | Wrong Comparator Group |
| Belda Nacher Fj, Rovira Soriano L. Moreno Pachon J. Hernandez Laforet J. Ballester Lujan M. Ortega Serrano J. Cassinello Fernandez N. Llorens Herrerias J.. Effect of preoperative respiratory physiotherapy program on the oxygenation in obese morbid patients undergoing laparoscopic bariatric surgery. American journal of respiratory and critical care medicine. 2012. 185: | Wrong or no Outcome |
| Soares Smtp, Nucci L. B. Campacci T. C.. Impairment of respiratory muscle and lung function in patients submitted to abdominal surgery with and without preoperative physiotherapy care. American journal of respiratory and critical care medicine. 2012. 185: | Wrong or no Outcome |
| Bottin Jh, Thomas E. L. Fitzpatrick J. A. Durighel G. Balogun B. Moorthy K. Leeds A. R. Bell J. D. Frost G. S.. Pre-operative liquid low-calorie diet reduces liver fat and liver volume in morbidly obese patients undergoing roux-en-y gastric bypass. Obesity facts. 2015. 8:219 | Wrong or no Outcome |
| Loi, N.. A Randomised Controlled Trial Study of the Efficacy of Intensive Pre-Operative Pelvic Floor Muscle Training to Decrease Post-Prostatectomy Urinary Incontinence (Abstract number OP.4.7Dec.38). International Journal of Urology. 2014. 21:A169 | Wrong or no Outcome |
| Eschalier B, Descamps S. Pereira B. Girard M. G. Boisgard S. Coudeyre E.. Evaluation of a pre operative education approach for patient undergoing total knee replacement, Evaluation d'une demarche d'education therapeutique preoperatoire avant arthroplastie totale de genou. [French, English]. Annals of physical and rehabilitation medicine. 2012. 55:e117 | Not Prehabilitation |
| Tan Y, Xu Y. Wang S. Wang X. Li L.. A prospective study on quality of lfe in colorectal cancer patients with prophylactic preoperative enteral nutrition. Colorectal disease. 2012. 14:30 | Unregistered Abstract |
| Creel D, Schuh L. M. Gomez A. Reed C. Baumer L. Cacucci B. Diaz D. Evanson C. M. Huse J. M. Inman M. Kaderabek D.. The impact of exercise counseling on physical activity among bariatric surgery patients. Obesity (Silver Spring, Md.). 2011. 19:S191 | Wrong or Unclear Duration |
| Yavangi M, Mahmoodvand T.. Evaluation of the effect of pre-operative physiotherapy on quality of life and complications of colporraphy surgery in women suffering from pelvic organ prolapse. Http://www.irct.ir/searchresult.php?id=8772&number=1. 2013. | Protocol |
| Rovira Soriano L, Moreno Pachon J. Hernandez Laforet J. Ballester Lujan M. Belda Nacher F. J. Llorens Herrerias J.. Effects of respiratory preoperative physiotherapy on the intraoperative respiratory mechanics and oxygenation in patients undergoing laparoscopic bariatric surgery. European journal of anaesthesiology. 2012. 29:88 | Wrong or no Outcome |
| Haji S, Ohyanagi H.. Apoptosis induction of hepatocellular carcinoma via mtor signaling pathway by oral BCAA administration in surgical patients. Clinical Nutrition, Supplement. 2011. 6:146 | Not Prehabilitation |
| Lu Q-L, Zheng K. Zhang P.. Effect of preoperative enteral nutrition on postoperative infections and nutritional indices in esophageal cancer patients with esophageal stenosis. World Chinese Journal of Digestology. 2013. 21:2434 | Not Prehabilitation |
| Hu Ql, Xu Y. Feng C. C. Dong Wang X. Li K.. A prospective study on postoperative nutrition and complications in colorectal cancer patients with preoperative immune-enhancing enteral nutrition. Colorectal Disease. Conference: 7th Scientific and Annual Meeting of the European Society of Coloproctology Vienna Austria. Conference Start:. 2012. 14:3 | Wrong or Unclear Duration |
| Chapman P, McDonald S. Stalker L. Badcoe A. Lethborg C. Santamaria J., et al.. A pilot study of the effectiveness of a pre-operative supportive care education and counselling intervention for patients undergoing major head and neck cancer surgery. Asia-Pacific journal of clinical oncology. 2012. 8:339 | Not Prehabilitation |
| Walther C, Fiess A.. Preoperative exercise training is associated with less peri- and postoperative adverse events but similar long term outcome in patients with stable coronary artery disease. European journal of cardiovascular prevention and rehabilitation. 2010. 17:S59 | Unregistered Abstract |
| De-Fang Z, Ke Z. Ren L. Li-Jun Z.. Clinical observation of enteral immunonutrition in patients undergoing liver transplantation. Journal of Clinical Rehabilitative Tissue Engineering Research. 2011. 15:5873 | Wrong or no Outcome |
| St-Pierre, J., Drummond, K., Minella, E., Scheede-Bergdahl, C., Ferri, L., Carli, F.. Feasibility of multimdodal prehabilitation to enhance preoperative functional capacity of esophageal cancer patients during concurrent neoadjuvant chemotherapies - a pilot interventional study. European Journal of Surgical Oncology. 2022. 48:e38 | Wrong or no Outcome |
| Perioperative optimization with nutritional supplements in patients undergoing gastrointestinal surgery for cancer: A randomized, placebo controlled feasibility clinical trial. 2022. | Duplicate |
| Loughney, L., Tully, R., Bolger, J. C., Sorensen, J., McAnena, O., Collins, C. G., Carroll, P. A., Arumugasamy, M., Murphy, T. J., McCaffrey, N., Robb, W. B.. The effect of a pre-and post-operative exercise programme versus standard care on physical fitness of patients with oesophageal and gastric cancer undergoing neoadjuvant treatment prior to surgery (The PERIOP-OG Trial): A Randomised controlled trial. British Journal of Surgery. 2021. 108: | Wrong or Unclear Duration |
| Heiman, J., Haglind, E., Olofsson Bagge, R.. Author response to: Recovery after breast cancer surgery following a recommendation of physical activity pre- And postoperatively (PhysSURG-B) - A randomized clinical trial. British Journal of Surgery. 2021. 108:E273 | Not a Randomized Trial |
| Braxton, E., Myers, E., Zhao, J., Evans, S., Tarr, M.. In-office versus telemedicine preoperative visit: A randomized controlled trial. Female Pelvic Medicine and Reconstructive Surgery. 2021. 27:S3 | Not Prehabilitation |
| Braxton, E. G., Myers, E. M., Zhao, J., Evans, S., Tarr, M. E.. In-Person Versus Video Preoperative Visit: A Randomized Clinical Trial. Journal of Minimally Invasive Gynecology. 2021. 28:S17 | Not Prehabilitation |
| Lahteenmaki, S. I., Sioris, T., Mahrberg, H. S. S., Rinta-Kiikka, I. C., Laurikka, J. O.. A randomized trial comparing inspiratory training and positive pressure training in immediate lung recovery after minor pleuro-pulmonary surgery. Journal of Thoracic Disease. 2021. 13:4690-4702 | Wrong or Unclear Duration |
| Samir, M., Amrawy, W., Hassab, T.. A low cost eras bundle reduces the length and cost of stay following colorectal surgery in a community hospital: A randomized controlled study. Diseases of the Colon and Rectum. 2021. 64:193 | Not Prehabilitation |
| Koc, Mehmet A., Akyol, Cihangir, Gokmen, Derya, Aydin, Durucan, Erkek, Bulent Ayhan, Kuzu, Mehmet A.. Effect of Prehabilitation on Stoma Self-Care, Anxiety, Depression and Quality of Life in Stoma Patients: A Randomized Controlled Trial. Diseases of the colon and rectum. 2022. | Wrong or Unclear Duration |
| Meyer, Vanessa M., Beydoun, Hind A., Gyenai, Leonora, Goble, Nicole M., Hunter, Michelle M., McGill, Robert J.. The Effect of Preoperative Behavioral Intervention on Pain, Anxiety, Opioid Use, and Function in Patients Undergoing Total Knee Arthroplasty: A Randomized Controlled Study. Military medicine. 2021. | Wrong or Unclear Duration |
| Kushner, B. S., Holden, T., Han, B., Sehnert, M., Majumder, A., Blatnik, J. A., Holden, S. E.. Randomized control trial evaluating the use of a shared decision-making aid for older ventral hernia patients in the Geriatric Assessment and Medical Preoperative Screening (GrAMPS) Program. Hernia : the journal of hernias and abdominal wall surgery. 2021. | Wrong or no Outcome |
| Allen, Sophie, Sultan, Javed. ASO Author Reflections: A Randomized Controlled Trial to Address the Effect of Prehabilitation During Neoadjuvant Therapy on Cardiopulmonary Fitness, Muscle Mass and Quality of Life in the Oesophagogastric Cancer Patient. Annals of surgical oncology. 2022. 29:1851-1852 | Not a Randomized Trial |
| Vincent, Sage, Paskey, Taylor, Critchlow, Elizabeth, Mann, Erica, Chapman, Talia, Abboudi, Jack, Jones, Christopher, Kirkpatrick, William, Namdari, Surena, Hammoud, Sommer, Ilyas, Asif M.. Prospective Randomized Study Examining Preoperative Opioid Counseling on Postoperative Opioid Consumption after Upper Extremity Surgery. Hand (New York, N.Y.). 2022. 17:200-205 | Wrong or Unclear Duration |
| Schiavo, Luigi, Pierro, Roberto, Asteria, Carmela, Calabrese, Pietro, Di Biasio, Alberto, Coluzzi, Ilenia, Severino, Lucia, Giovanelli, Alessandro, Pilone, Vincenzo, Silecchia, Gianfranco. Low-Calorie Ketogenic Diet with Continuous Positive Airway Pressure to Alleviate Severe Obstructive Sleep Apnea Syndrome in Patients with Obesity Scheduled for Bariatric/Metabolic Surgery: a Pilot, Prospective, Randomized Multicenter Comparative Study. Obesity surgery. 2022. 32:634-642 | Wrong or no Outcome |
| Waller, Ellen, Sutton, Paul, Rahman, Seema, Allen, Jonathan, Saxton, John, Aziz, Omer. Prehabilitation with wearables versus standard of care before major abdominal cancer surgery: a randomised controlled pilot study (trial registration: NCT04047524). Surgical endoscopy. 2022. 36:1008-1017 | Wrong or no Outcome |
| Zheng, Yuliu, Huang, Zida, Dai, Liqun, Liu, Yu, Chen, Yanqin, Zhang, Wenming, Lin, Rongjin. The Effect of Preoperative Rehabilitation Training on the Early Recovery of Joint Function after Artificial Total Knee Arthroplasty and Its Effect Evaluation. Journal of healthcare engineering. 2022. 2022:3860991 | Wrong or Unclear Duration |
| Lopes, Andre, Yamada, Alayne Magalhaes Trindade Domingues, Cardenas, Thais de Campos, Carvalho, Jaqueline Nunes de, Oliveira, Emilia de Azevedo, Silva, Marina Elisa Ribeiro da, Andrade, Juliana Fenerich Mauri, de Souza Neto, Eduardo, Barros, Lilian Arruda do Rego, Costa, Ronaldo Lucio Rangel. PROPER-PRehabilitatiOn Plus Enhanced Recovery after surgery versus enhanced recovery after surgery in gynecologic oncology: a randomized clinical trial. International journal of gynecological cancer : official journal of the International Gynecological Cancer Society. 2022. 32:195-197 | Protocol |
| Weerasinghe, Kalani, Rishard, Mohamed, Brabaharan, Subhani, Mohamed, Aysha. Effectiveness of face-to-face physiotherapy training and education for women who are undergoing elective caesarean section: a randomized controlled trial. Archives of physiotherapy. 2022. 12:4 | Intervention Not Preoperative |
| Tabriz, Navid, Fried, Dennis, Uslar, Verena, Weyhe, Dirk. The Influence of Prophylactic Calcium and Magnesium Supplementation on Postoperative Quality of Life and Hypocalcemia After Total Thyroidectomy: Study Protocol for a Randomized Controlled Trial. Frontiers in surgery. 2021. 8:758205 | Not Prehabilitation |
| Rydja, Johanna, Kollen, Lena, Hellstrom, Per, Owen, Katarina, Lundgren Nilsson, Asa, Wikkelso, Carsten, Tullberg, Mats, Lundin, Fredrik. Physical exercise and goal attainment after shunt surgery in idiopathic normal pressure hydrocephalus: a randomised clinical trial. Fluids and barriers of the CNS. 2021. 18:51 | Wrong or Unclear Duration |
| Savkin, Raziye, Buker, Nihal, Gungor, Harun R.. The effects of preoperative neuromuscular electrical stimulation on the postoperative quadriceps muscle strength and functional status in patients with fast-track total knee arthroplasty. Acta orthopaedica Belgica. 2021. 87:735-744 | Not Prehabilitation |
| Murdoch, Jamie, Varley, Anna, McCulloch, Jane, Jones, Megan, Thomas, Laura B., Clark, Allan, Stirling, Susan, Turner, David, Swart, Ann Marie, Dresser, Kerry, Howard, Gregory, Saxton, John, Hernon, James. Implementing supportive exercise interventions in the colorectal cancer care pathway: a process evaluation of the PREPARE-ABC randomised controlled trial. BMC cancer. 2021. 21:1137 | Not a Randomized Trial |
| Allen, Sophie K., Brown, Vanessa, White, Daniel, King, David, Hunt, Julie, Wainwright, Joe, Emery, Annabelle, Hodge, Emily, Kehinde, Aga, Prabhu, Pradeep, Rockall, Timothy A., Preston, Shaun R., Sultan, Javed. Multimodal Prehabilitation During Neoadjuvant Therapy Prior to Esophagogastric Cancer Resection: Effect on Cardiopulmonary Exercise Test Performance, Muscle Mass and Quality of Life-A Pilot Randomized Clinical Trial. Annals of surgical oncology. 2022. 29:1839-1850 | Duplicate |
| Lambaudie, Eric, Bannier/Braticevic, Cecile, Villaron/Goetgheluck, Charlene, Zemmour, Christophe, Boher, Jean-Marie, Ben Soussan, Patrick, Pakradouni, Jihane, Brun, Clement, Lopez Almeida, Leonor, Marino, Patricia. TRAINING-Ovary 01 (connecTed pRehabiliAtIoN pelvIc caNcer surGery): multicenter randomized study comparing neoadjuvant chemotherapy for patients managed for ovarian cancer with or without a connected pre-habilitation program. International journal of gynecological cancer : official journal of the International Gynecological Cancer Society. 2021. 31:920-924 | Not a Randomized Trial |
| Ben-Porat, Tair, Weiss, Ram, Khalaileh, Abed, Abu Gazala, Mahmud, Kaluti, Dunia, Mintz, Yoav, Sherf-Dagan, Shiri, Yackobovitch-Gavan, Michal, Rottenstreich, Amihai, Brodie, Ronit, Pikarsky, Alon J., Elazary, Ram. The impact of preoperative vitamin administration on skeletal status following sleeve gastrectomy in young and middle-aged women: a randomized controlled trial. International journal of obesity (2005). 2021. 45:1925-1936 | Not Prehabilitation |
| Feng, Jinhua, Xu, Ruihua, Li, Ka, Li, Fuyu, Gao, Min, Han, Qiang, Feng, Huan, Ye, Hui. Effects of preoperative oral carbohydrate administration combined with postoperative early oral intake in elderly patients undergoing hepatectomy with acute-phase inflammation and subjective symptom burden: A prospective randomized controlled study. Asian journal of surgery. 2022. 45:386-395 | Wrong or Unclear Duration |
| Atkins, Kelly J., Scott, David A., Silbert, Brendan, Pike, Kerryn E., Evered, Lis. Preventing Delirium and Promoting Long-Term Brain Health: A Clinical Trial Design for the Perioperative Cognitive Enhancement (PROTECT) Trial. Journal of Alzheimer's disease : JAD. 2021. 83:1637-1649 | Not a Randomized Trial |
| Sassani, Jessica C., Grosse, Philip J., Kunkle, Lauren, Baranski, Lindsey, Ackenbom, Mary F.. Patient Preparedness for Pelvic Organ Prolapse Surgery: A Randomized Equivalence Trial of Preoperative Counseling. Female pelvic medicine & reconstructive surgery. 2021. 27:719-725 | Not Prehabilitation |
| Diaz, Sofia I., Yan, Luying, Dai, Feng, Zhou, Bin, Burg, Matthew M., Schonberger, Robert B.. Feasibility of a randomized hypertension screening initiative in the perioperative setting. Perioperative medicine (London, England). 2021. 10:39 | Single Risk Factor Management |
| Effects of preoperative physiotherapy on signs and symptoms of pulmonary collapse and infection after major abdominal surgery: secondary analysis of the LIPPSMAck-POP multicentre randomised controlled trial. 2021. | Not Prehabilitation |
| Su, Xiaojing, Wang, Zheng, Wu, Bing, Song, Kai, Chen, Yixiu, Zhang, Chunzheng, Xue, Chuanjuan, Ge, Lili, Liu, Yang. Care of abdominal skin in ankylosing spondylitis patients undergoing corrective spinal surgery. Experimental and therapeutic medicine. 2021. 22:1350 | Not Prehabilitation |
| Cannata, Francesca, Laudisio, Alice, Russo, Fabrizio, Ambrosio, Luca, Vadala, Gianluca, Cardinale, Marco Edoardo, Bartolomei, Chiara, Iannone, Gabriella, Napoli, Nicola, Papalia, Rocco. Weight Loss in Patients Waiting for Total Hip Arthroplasty: Fiber-Enriched High Carbohydrate Diet Improves Hip Function and Decreases Pain before Surgery. Journal of clinical medicine. 2021. 10: | Not Prehabilitation |
| Alaparthi, Gopala Krishna, Amin, Revati, Gatty, Aishwarya, Raghavan, Harish, Bairapareddy, Kalyana Chakravarthy, Vaishali, K., Borghi-Silva, Audrey, Hegazy, Fatma A.. Contrasting effects of three breathing techniques on pulmonary function, functional capacity and daily life functional tasks in patients following valve replacement surgery- A pilot randomized clinical trial. Heliyon. 2021. 7:e07643 | Not Prehabilitation |
| Miller, William C., Mohammadi, Somayyeh, Watson, Wendy, Crocker, Morag, Westby, Marie. The Hip Instructional Prehabilitation Program for Enhanced Recovery (HIPPER) as an eHealth Approach to Presurgical Hip Replacement Education: Protocol for a Randomized Controlled Trial. JMIR research protocols. 2021. 10:e29322 | Protocol |
| Perez-Saez, Maria Jose, Morgado-Perez, Andrea, Faura, Anna, Munoz-Redondo, Elena, Garriz, Miguel, Muns, Maria Dolors, Nogues, Xavier, Marco, Ester, Pascual, Julio. The FRAILMar Study Protocol: Frailty in Patients With Advanced Chronic Kidney Disease Awaiting Kidney Transplantation. A Randomized Clinical Trial of Multimodal Prehabilitation. Frontiers in medicine. 2021. 8:675049 | Protocol |
| Wierdak, Mateusz, Surmiak, Marcin, Milian-Ciesielska, Katarzyna, Rubinkiewicz, Mateusz, Rzepa, Anna, Wysocki, Michal, Major, Piotr, Klek, Stanislaw, Pedziwiatr, Michal. Immunonutrition Changes Inflammatory Response in Colorectal Cancer: Results from a Pilot Randomized Clinical Trial. Cancers. 2021. 13: | Wrong or no Outcome |
| Wong, T. X., Chen, S. T., Ong, S. H., Shyam, S., Kandasami, P., Chee, W. S. S.. Study protocol for an open labelled randomised controlled trial of perioperative oral nutrition supplement in breast and colorectal cancer patients undergoing elective surgery. Trials. 2021. 22:767 | Wrong or Unclear Duration |
| Ferreira, Vanessa, Lawson, Claire, Carli, Francesco, Scheede-Bergdahl, Celena, Chevalier, Stephanie. Feasibility of a novel mixed-nutrient supplement in a multimodal prehabilitation intervention for lung cancer patients awaiting surgery: A randomized controlled pilot trial. International journal of surgery (London, England). 2021. 93:106079 | Wrong or no Outcome |
| Sweity, Essa M., Alkaissi, Aidah A., Othman, Wafiq, Salahat, Ahmad. Preoperative incentive spirometry for preventing postoperative pulmonary complications in patients undergoing coronary artery bypass graft surgery: a prospective, randomized controlled trial. Journal of cardiothoracic surgery. 2021. 16:241 | Wrong or Unclear Duration |
| Shonka, David C., Jr., Maxwell, Anne K., Petroni, Gina R., Jameson, Mark J.. Phase II randomized study of preoperative calcitriol to prevent hypocalcemia following thyroidectomy. Head & neck. 2021. 43:2935-2945 | Not Prehabilitation |
| Gonzalez-Santos, Angela, Postigo-Martin, Paula, Gallart-Aragon, Tania, Esteban-Cornejo, Irene, Lopez-Garzon, Maria, Galiano-Castillo, Noelia, Arroyo-Morales, Manuel, Illescas-Montes, Rebeca, Artacho-Cordon, Francisco, Martin-Martin, Lydia, Forneiro-Perez, Rocio, Lozano-Lozano, Mario, Fernandez-Lao, Carolina, Ruiz-Vozmediano, Julia, Sanchez-Salgado, Carmen, Cantarero-Villanueva, Irene. Neurotoxicity prevention with a multimodal program (ATENTO) prior to cancer treatment versus throughout cancer treatment in women newly diagnosed for breast cancer: Protocol for a randomized clinical trial. Research in nursing & health. 2021. 44:598-607 | Intervention Not Preoperative |
| Rao, Brian M., Cieslewicz, Thomas J., Sochacki, Kyle R., Kohlrieser, David A., Moylan, Daniel D., Ellis, Thomas J.. Worse Preoperative Pain and Higher Activity Levels Predict Patient Choice of Formal Physical Therapy After Primary Anterior Total Hip Arthroplasty. The Journal of arthroplasty. 2021. 36:2823-2828.e2 | Not Prehabilitation |
| Chen, Xinrong, Li, Ka, Yang, Kun, Hu, Jiankun, Yang, Jie, Feng, Jinhua, Hu, Yanjie, Zhang, Xingxia. Effects of preoperative oral single-dose and double-dose carbohydrates on insulin resistance in patients undergoing gastrectomy:a prospective randomized controlled trial. Clinical nutrition (Edinburgh, Scotland). 2021. 40:1596-1603 | Wrong or Unclear Duration |
| Arman, Nilay, Tokgoz, Gulfidan, Seyit, Hakan, Karabulut, Mehmet. The effects of core stabilization exercise program in obese people awaiting bariatric surgery: A randomized controlled study. Complementary therapies in clinical practice. 2021. 43:101342 | Wrong or no Outcome |
| Koet, Lesley Larissa, Kraima, Annelot, Derksen, Ilona, Lamme, Bas, Belt, Eric Jacobus Theodorus, van Rosmalen, Joost, Smeenk, Robert Matthijs, van der Hoeven, Joost Alexander Boreas. Effectiveness of preoperative group education for patients with colorectal cancer: managing expectations. Supportive care in cancer : official journal of the Multinational Association of Supportive Care in Cancer. 2021. 29:5263-5271 | Not Prehabilitation |
| Rojewski, Alana M., Fucito, Lisa M., Baker, Nathaniel L., Krishnan-Sarin, Suchitra, Carpenter, Matthew J., Bernstein, Steven L., Toll, Benjamin A.. A Preoperative Contingency Management Intervention for Smoking Abstinence in Cancer Patients: A Preliminary Randomized Controlled Trial. Nicotine & tobacco research : official journal of the Society for Research on Nicotine and Tobacco. 2021. 23:1064-1067 | Single Risk Factor Management |
| Herrera-Santelices, Andrea, Tabach-Apraiz, Andrea, Andaur-Caceres, Karen, Zamuner, Antonio Roberto. Effect of physical exercise in bariatric surgery patients: protocol of a randomized controlled clinical trial. Trials. 2021. 22:107 | Protocol |
| Rose, Alexandra V., Duhamel, Todd, Hyde, Chris, Kent, Dave E., Afilalo, Jonathan, Schultz, Annette S. H., Chudyk, Anna, Kehler, Dustin S., Dave, Mudra, Arora, Rakesh C.. Randomised controlled trial protocol for the PROTECT-CS Study: PROTein to Enhance outComes of (pre)frail paTients undergoing Cardiac Surgery. BMJ open. 2021. 11:e037240 | Protocol |
| Buvanendran, Asokumar, Sremac, Amanda C., Merriman, Patricia A., Della Valle, Craig J., Burns, John W., McCarthy, Robert J.. Preoperative cognitive-behavioral therapy for reducing pain catastrophizing and improving pain outcomes after total knee replacement: a randomized clinical trial. Regional anesthesia and pain medicine. 2021. 46:313-321 | Wrong or no Outcome |
| van der Velde, Miriam, Valkenet, Karin, Geleijn, Edwin, Kruisselbrink, Marjoke, Marsman, Marije, Janssen, Liedewij Mj, Ruurda, Jelle P., van der Peet, Donald L., Aarden, Jesse J., Veenhof, Cindy, van der Leeden, Marike. Usability and Preliminary Effectiveness of a Preoperative mHealth App for People Undergoing Major Surgery: Pilot Randomized Controlled Trial. JMIR mHealth and uHealth. 2021. 9:e23402 | Not Prehabilitation |
| de Carvalho, Celina Soares, Silva, Thiago Huaytalla, Andre, Julio Cezar Sillos, de Barros, Larissa Alves Soares, Ferreira, Aline Alves, Murad, Leonardo Borges, Peres, Wilza Arantes Ferreira. Preoperative Fasting Abbreviation With Whey Protein Reduces the Occurrence of Postoperative Complications in Patients With Head and Neck Cancer: A Randomized Clinical Trial. Nutrition in clinical practice : official publication of the American Society for Parenteral and Enteral Nutrition. 2021. 36:665-672 | Wrong or Unclear Duration |
| Fors, Maria, Oberg, Birgitta, Lindback, Yvonne, Enthoven, Paul, Abbott, Allan. What Mediates Treatment Effects in a Presurgery Physiotherapy Treatment in Surgical Candidates With Degenerative Lumbar Spine Disorders? A Mediation and Conditional Process Analysis of the PREPARE Randomized Controlled Trial. The Clinical journal of pain. 2021. 37:168-176 | Not a Randomized Trial |
| Mousavie, Seyed Hamzeh, Negahi, Alireza, Hosseinpour, Parisa, Mohseni, Masood, Movassaghi, Shima. The Effect of Preoperative Oral Versus Parenteral Dextrose Supplementation on Pain, Nausea, and Quality of Recovery After Laparoscopic Cholecystectomy. Journal of perianesthesia nursing : official journal of the American Society of PeriAnesthesia Nurses. 2021. 36:153-156 | Wrong or Unclear Duration |
| Salzmann, Stefan, Euteneuer, Frank, Laferton, Johannes A. C., Shedden-Mora, Meike C., Schedlowski, Manfred, Moosdorf, Rainer, Rief, Winfried. IL-8 and CRP moderate the effects of preoperative psychological interventions on postoperative long-term outcomes 6 months after CABG surgery - The randomized controlled PSY-HEART trial. Brain, behavior, and immunity. 2021. 91:202-211 | Wrong or no Outcome |
| Yom, Kelly H., Shriver, Erin M., Carter, Keith D., Korn, Bobby S., Kikkawa, Don O., Ko, Audrey C.. The Effect of Photographic Visual Aids in Preoperative Patient Counseling in Oculoplastic Surgery. Ophthalmic plastic and reconstructive surgery. 2021. 37:S70-S75 | Wrong or Unclear Duration |
| Fulop, A., Lakatos, L., Susztak, N., Szijarto, A., Banky, B.. The effect of trimodal prehabilitation on the physical and psychological health of patients undergoing colorectal surgery: a randomised clinical trial. Anaesthesia. 2021. 76:82-90 | Duplicate |
| Longobardi, Ylenia, Savoia, Vezio, Parrilla, Claudio, Marchese, Maria Raffaella, Morra, Luciana, Mari, Giorgia, Degni, Emilia, D'Alatri, Lucia. Pre-operative speech-language pathology counselling in patients undergoing total laryngectomy: A pilot randomized clinical trial. Current Psychology: A Journal for Diverse Perspectives on Diverse Psychological Issues. 2021. :No-Specified | Wrong or Unclear Duration |
| Lopes A, Yamada Amtd Cardenas T. C. Carvalho J. N. Oliveira E. A. Silva Merd Andrade J. F. M. de Souza Neto E. Barros Ladr Costa R. L. R.. PROPER-PRehabilitatiOn Plus Enhanced Recovery after surgery versus enhanced recovery after surgery in gynecologic oncology: a randomized clinical trial. International journal of gynecological cancer. 2021. | Not a Randomized Trial |
| Allen Sk, Brown V. White D. King D. Hunt J. Wainwright J. Emery A. Hodge E. Kehinde A. Prabhu P. Rockall T. A. Preston S. R. Sultan J.. Multimodal Prehabilitation During Neoadjuvant Therapy Prior to Esophagogastric Cancer Resection: effect on Cardiopulmonary Exercise Test Performance, Muscle Mass and Quality of Life-A Pilot Randomized Clinical Trial. Annals of surgical oncology. 2021. | Duplicate |
| Nct. Effect of Perioperative OPEP Therapy on Post-operative Pulmonary Complications: a Pilot Study. Effectiveness of Perioperative Oscillating Positive Expiratory Pressure (OPEP) Therapy in Reduction of Post-operative Respiratory Morbidity in Patients Undergoing Colorectal Surgery: a Pilot Randomized Control Trial. 2021. | Not a Randomized Trial |
| Schiavo L, Pierro R. Asteria C. Calabrese P. Di Biasio A. Coluzzi I. Severino L. Giovanelli A. Pilone V. Silecchia G.. Low-Calorie Ketogenic Diet with Continuous Positive Airway Pressure to Alleviate Severe Obstructive Sleep Apnea Syndrome in Patients with Obesity Scheduled for Bariatric/Metabolic Surgery: a Pilot, Prospective, Randomized Multicenter Comparative Study. Obesity surgery. 2021. | Single Risk Factor Management |
| Nct. Influence of Preoperative Nutritional Status on Perioperative Period in Cardiac Surgery in Patients With Frailty. Influence of Optimization of Preoperative Nutritional Status on Perioperative Period in Cardiac Surgery in Patients With Frailty. 2021. | Protocol |
| Nct. Virtual and Video Counseling Versus In-Office Counseling for Laparoscopic Hysterectomy. Virtual and Video Counseling Versus In-Office Counseling for Laparoscopic Hysterectomy: a Randomized Controlled Trial. 2021. | No Results |
| Reynolds Mj, Townsend C. Ilyas A. M.. Prospective randomized trial studying the effects of preoperative opioid education on postoperative opioid consumption after outpatient upper extremity surgery. AAOS american academy of orthopaedic surgeons. 2021. | Not Prehabilitation |
| Townsend C, Ilyas A. M.. The effect of preoperative opioid education on postoperative opioid consumption after outpatient orthopaedic surgery: prospective randomized trial. AAOS american academy of orthopaedic surgeons. 2021. | Not Prehabilitation |
| Nct. Metabolic Stress-induced Exercise to Prevent Loss of Muscle Mass in Patients With Pancreatic and Biliary Tract Cancer. Metabolic Stress-induced Exercise in the Perioperative Setting to Prevent the Loss of Muscle Mass in Patients With Pancreatic and Biliary Tract Cancer - the PREV-Ex Randomized Controlled Trial. 2021. | No Results |
| Nct. COgnitive and Physical Exercise to Improve Outcomes After Surgery (COPE-iOS) Study. COgnitive and Physical Exercise to Improve Outcomes After Surgery (COPE-iOS) Study. 2021. | No Results |
| Shu X-l, Kang K. Zhong J. x S. H. I. Z. x L. I. U. X. l Wang M. h L. I. U. Z. d Zhang Y. s Yao Y.. Effect of individualized nutritional therapy on clinical outcome of perioperative patients with digestive system diseases. Academic journal of second military medical university. 2021. 42:55 | Wrong or Unclear Duration |
| Nct. Preoperative Rehabilitation in Greek Patients Undergoing Total Knee Arthroplasty. Efficacy of High Intensity Preoperative Training on Postoperative Outcomes in Greek Patients Undergoing Total Knee Arthroplasty: a Randomized Controlled Study. 2021. | No Results |
| Onerup A, Andersson J. Angenete E. Bock D. Borjesson M. Ehrencrona C. Olsen M. F. Larsson P. A. de la Croix H. Wedin A. Haglind E.. Effect of Short-Term Homebased Pre- and Postoperative Exercise on Recovery after Colorectal Cancer Surgery (PHYSSURG-C): a Randomized Clinical Trial. Annals of surgery. 2021. | Duplicate |
| Waller E, Sutton P. Rahman S. Allen J. Saxton J. Aziz O.. Prehabilitation with wearables versus standard of care before major abdominal cancer surgery: a randomised controlled pilot study (trial registration: NCT04047524). Surgical endoscopy. 2021. | Wrong or no Outcome |
| Berkel Aem, Bongers B. C. Kotte H. Weltevreden P. de Jongh F. H. C. Eijsvogel M. M. M. Wymenga A. N. M. Bigirwamungu-Bargeman M. van der Palen J. van Det M. J. van Meeteren N. L. U. Klaase J. M.. Effects of Community-based Exercise Prehabilitation for Patients Scheduled for Colorectal Surgery With High Risk for Postoperative Complications: results of a Randomized Clinical Trial. Annals of surgery. 2021. | Duplicate |
| Chakravartty, S., Sidhu, P., Vivian, G., Shaikh, H., McGrath, J., Patel, A.. PRE-OPERATIVE LIVER SHRINKING DIETS CAN ALTER COLLAGEN GENE EXPRESSION IN WOUND HEALING: A RANDOMISED CONTROLLED TRIAL. Obesity Surgery. 2017. 27:114-114 | Not Prehabilitation |
| Chakravartty, S., Murgatroyd, B., Jaffer, O., Sidhu, P., Vivian, G., Patel, A.. Randomised controlled trial comparing the effect of pre-operative liver shrinking diet on peri-operative outcomes in morbidly obese patients undergoing gastric bypass: Is it time to change our practice?. British Journal of Surgery. 2014. 101:8-8 | Not a Randomized Trial |
| Zgaia, A., Pop, F., Achimas-Cadariu, P., Vlad, C., Rogobete, A., Lisencu, C., Ignat, F., Lazar, G., Muresan, M., Muresan, M. S., Ciorogar, G., Irimie, A.. THE IMPACT OF RELAXATION TECHNIQUE AND PRE-OPERATIVE PSYCHOLOGICAL COUNSELLING ON PAIN, ANALGESIC CONSUMPTION AND PSYCHOLOGICAL SYMPTOMS ON PATIENTS SCHEDULED FOR BREAST CANCER SURGERY - A RANDOMIZED CLINICAL STUDY. Journal of Evidence-Based Psychotherapies. 2016. 16:205-220 | Wrong or Unclear Duration |
| Ligibel, J. A., Dillon, D., Giobbie-Hurder, A., McTiernan, A., Frank, E., Cornwell, M., Pun, M., Campbell, N., Dowling, R. J. O., Chang, M. C., Tolaney, S., Chagpar, A. B., Yung, R. L., Freedman, R. A., Dominici, L. S., Golshan, M., Rhei, E., Taneja, K., Huang, Y., Brown, M., Winer, E. P., Jeselsohn, R., Irwin, M. L.. Impact of a Pre-Operative Exercise Intervention on Breast Cancer Proliferation and Gene Expression: Results from the Pre-Operative Health and Body (PreHAB) Study. Clinical Cancer Research. 2019. 25:5398-5406 | Wrong or no Outcome |
| Silva, L., Hanratty, D., Horwood, J., da Silva, A.. A randomised control trial evaluating the effect of pre- operative forearm exercise on cephalic vein diameter and vascular access outcome. British Journal of Surgery. 2018. 105:65-65 | Unregistered Abstract |
| Baillot, A., Vallee, C. A., Mampuya, W. M., Dionne, I. J., Comeau, E., Meziat-Burdin, A., Langlois, M. F.. Effects of a Pre-surgery Supervised Exercise Training 1 Year After Bariatric Surgery: a Randomized Controlled Study. Obesity Surgery. 2018. 28:955-962 | Duplicate |
| Reynu, R., Zainal, Z. A., Kosai, N. R.. PRE-OPERATIVE METHODS TO REDUCE LIVER VOLUME: A RANDOMISED CONTROLLED TRIAL COMPARING THE USE OF OMEGA-3 POLYUNSATURATED FATTY ACID SUPPLEMENTS VERSUS VERY LOW CALORIE DIETARY RESTRICTION IN PATIENTS AWAITING BARIATRIC SURGERY. Obesity Surgery. 2016. 26:S122-S123 | Not Prehabilitation |
| Schouten, R., van der Kaaden, I., van 't Hof, G., Feskens, Pgbm. Comparison of Preoperative Diets Before Bariatric Surgery: a Randomized, Single-Blinded, Non-inferiority Trial. Obesity Surgery. 2016. 26:1743-1749 | Duplicate |
| A randomised, single-blinded trial assessing the effect of a two week preoperative very low calorie diet on laparoscopic cholecystectomy in obese patients. 2014. | Duplicate |
| Doiron-Cadrin, P., Kairy, D., Vendittoli, P. A., Lowry, V., Poitras, S., Desmeules, F.. Feasibility and preliminary effects of a tele-prehabilitation program and an in-person prehablitation program compared to usual care for total hip or knee arthroplasty candidates: a pilot randomized controlled trial. Disability and Rehabilitation. 2020. 42:989-998 | Wrong or no Outcome |
| Doiron-Cadrin, P., Kairy, D., Vendittoli, P. A., Lowry, V., Poitras, S., Desmeules, F.. Effects of a tele-prehabilitation program or an in-person prehabilitation program in surgical candidates awaiting total hip or knee arthroplasty: Protocol of a pilot single blind randomized controlled trial. Contemporary Clinical Trials Communications. 2016. 4:192-198 | Protocol |
| Gloor, S., Misirlic, M., Frei-Lanter, C., Herzog, P., Muller, P., Schafli-Thurnherr, J., Schregel, D., Lamdark, T., Wyss, R., Unger, I., Gisi, D., Greco, N., Mungo, G., Wirz, M., Raptis, D. A., Tschuor, C., Breitenstein, S.. Prehabilitation in patients undergoing colorectal surgery fails to confer reduction in overall morbidity: Results of a single-center, single-blinded, randomized controlled trial. British Journal of Surgery. 2021. 108:2 | Duplicate |
| Lotzke, H., Jakobsson, M., Brisby, H., Gutke, A., Hagg, O., Smeets, R., den Hollander, M., Olsson, L. E., Lundberg, M.. Use of the PREPARE (PREhabilitation, Physical Activity and exeRcisE) program to improve outcomes after lumbar fusion surgery for severe low back pain: a study protocol of a person-centred randomised controlled trial. Bmc Musculoskeletal Disorders. 2016. 17:13 | Protocol |
| Coca-Martinez, M., Lopez-Hernandez, A., Montane-Muntane, M., Arguis, M. J., Gimeno-Santos, E., Navarro-Ripoll, R., Perdomo, J., Lopez-Baamonde, M., Rios, J., Moises, J., de la Garza, M. S., Sandoval, E., Romano, B., Sebio, R., Dana, F., Martinez-Palli, G.. Multimodal prehabilitation as strategy for reduction of postoperative complications after cardiac surgery: a randomised controlled trial protocol. Bmj Open. 2020. 10:9 | Protocol |
| McIsaac, D. I., Saunders, C., Hladkowicz, E., Bryson, G. L., Forster, A. J., Gagne, S., Huang, A., Lalu, M., Lavallee, L. T., Moloo, H., Nante, J., Power, B., Scheede-Bergdah, C., Taljaard, M., van Walraven, C., McCartney, C. J. L.. PREHAB study: a protocol for a prospective randomised clinical trial of exercise therapy for people living with frailty having cancer surgery. Bmj Open. 2018. 8:8 | Protocol |
| Williams, R. H. P., Heatley, R. V., Lewis, M. H., Hughes, L. E.. RANDOMIZED CONTROLLED TRIAL OF PREOPERATIVE INTRAVENOUS NUTRITION IN PATIENTS WITH STOMACH CANCER. British Journal of Surgery. 1976. 63:667-667 | Not Prehabilitation |
| Fan, S. T., Lau, W. Y., Wong, K. K., Chan, Y. P. M.. PREOPERATIVE PARENTERAL-NUTRITION IN PATIENTS WITH ESOPHAGEAL CANCER - A PROSPECTIVE, RANDOMIZED CLINICAL-TRIAL. Clinical Nutrition. 1989. 8:23-27 | Not Prehabilitation |
| Svinoy, O. E., Bergland, A., Risberg, M. A., Pripp, A. H., Hilde, G.. Better before-better after: efficacy of prehabilitation for older patients with osteoarthritis awaiting total hip replacement-a study protocol for a randomised controlled trial in South-Eastern Norway. Bmj Open. 2019. 9:8 | Protocol |
| Pantoni, C. B. F., Di Thommazo-Luporini, L., Mendes, R. G., Caruso, F. C. R., Castello-Simoes, V., Mezzalira, D., Borghi-Silva, A.. Effect of continuous positive airway pressure associated to exercise on the breathing pattern and heart rate variability of patients undergoing coronary artery bypass grafting surgery: a randomized controlled trial. Brazilian Journal of Medical and Biological Research. 2021. 54:8 | Intervention Not Preoperative |
| Davenport, L., Johari, Y., Klejn, A., Laurie, C., Smith, A., Ooi, G. J., Burton, P. R., Brown, W. A.. Improving Compliance with Very Low Energy Diets (VLEDs) Prior to Bariatric Surgery-A Randomized Controlled Trial of Two Formulations (vol 29, pg 2750, 2019). Obesity Surgery. 2019. 29:2758-2758 | Not a Randomized Trial |
| Davenport, L., Johan, Y., Klejn, A., Laurie, C., Smith, A., Ooi, G. J., Burton, P. R., Brown, W. A.. Improving Compliance with Very Low Energy Diets (VLEDs) Prior to Bariatric Surgery-a Randomised Controlled Trial of Two Formulations. Obesity Surgery. 2019. 29:2750-2757 | Not Prehabilitation |
| van Rooijen, S. J., Molenaar, C. J. L., Schep, G., van Lieshout, Rhma, Beijer, S., Dubbers, R., Rademakers, N., Papen-Botterhuis, N. E., van Kempen, S., Carli, F., Roumen, R. M. H., Slooter, G. D.. Making Patients Fit for Surgery Introducing a Four Pillar Multimodal Prehabilitation Program in Colorectal Cancer. American Journal of Physical Medicine & Rehabilitation. 2019. 98:888-896 | Not a Randomized Trial |
| Blackwell, J. E. M., Brook, M. S., Doleman, B., Morton, A., Williams, J. P., Lund, J. N., Phillips, B. E.. A Randomised Clinical Trial Of 'Prehabilitation' High Intensity Interval Training (HIIT) Before Urological Cancer Surgery. Medicine and Science in Sports and Exercise. 2019. 51:6-6 | Wrong or no Outcome |
| Allen, S., Brown, V., White, D., King, D., Prabhu, P., Rockall, T., Preston, S., Sultan, J.. Multi-modal prehabilitation during neoadjuvant therapy prior to resection for oesophagogastric cancer: A Randomised Controlled Trial. British Journal of Surgery. 2018. 105:8-9 | Wrong or no Outcome |
| Jensen, B. T., Laustsen, S., Jensen, J. B., Borre, M., Petersen, A. K.. EXERCISE-BASED PREHABILITATION IS FEASIBLE AND EFFECTIVE IN RADICAL CYSTECTOMY PATHWAYS - SECONDARY RESULTS FROM A RANDOMIZED CONTROLLED TRIAL. Journal of Urology. 2016. 195:E652-E652 | Wrong or no Outcome |
| Przkora, R., Sibille, K., Victor, S., Meroney, M., Leeuwenburgh, C., Gardner, A., Vasilopoulos, T., Parvataneni, H. K.. Feasibility of Blood Flow Restriction Exercise Prehabilitation to Attenuate Postoperative Loss of Function after Total Knee Replacement: A Randomized Pilot Study. Anesthesia and Analgesia. 2021. 132:868-869 | Duplicate |
| Northgraves, M. J., Arunachalam, L., Marshall, P., Madden, L. A., Hartley, J. E., Macfie, J., Vince, R. V.. The effects of exercise preconditioning on patients scheduled for elective colorectal surgery: a randomised controlled pilot study. British Journal of Surgery. 2016. 103:16-17 | Duplicate |
| Zhu, W. M., Guo, Z., Zuo, L. G., Gong, J. F., Gu, L. L., Cao, L., Li, N., Li, J. S.. CONSORT: Different End-Points of Preoperative Nutrition and Outcome of Bowel Resection of Crohn Disease A Randomized Clinical Trial. Medicine. 2015. 94:6 | Wrong or Unclear Duration |
| Liberman, Stein, Charlebois, Fiore. Effect of Multimodal Prehabilitation vs Postoperative Rehabilitation on 30-Day Postoperative Complications for Frail Patients Undergoing Resection of Colorectal Cancer: A Randomized Clinical Trial (vol 155, pg 233, 2020). Jama Surgery. 2020. 155:269-269 | Duplicate |
| Cho, H., Tsuburaya, A., Sakamoto, J., Morita, S., Oba, K., Yoshikawa, T., Miyajima, N.. A randomized phase II trial of preoperative exercise to reduce operative risk in gastric cancer patients with metabolic syndrome: Adjuvant exercise for general elective surgery (AEGES) study group. Japanese Journal of Clinical Oncology. 2008. 38:71-73 | Protocol |
| Martin, D., Besson, C., Pache, B., Michel, A., Geinoz, S., Gremeaux-Bader, V., Larcinese, A., Benaim, C., Kayser, B., Demartines, N., Hubner, M.. Feasibility of a prehabilitation program before major abdominal surgery: a pilot prospective study. Journal of International Medical Research. 2021. 49:11 | Not a Randomized Trial |
| Zgaia, A., Pop, F., Irimie, A., Rogobete, A., Achimas-Cadariu, P.. THE IMPACT OF PREOPERATIVE PSYCHOLOGICAL COUNSELING ON PAIN, AND ANALGESIC CONSUMPTION ON PATIENTS SCHEDULED FOR BREAST CANCER SURGERY- A RANDOMIZED CLINICAL STUDY. Anesthesia and Analgesia. 2016. 123:446-447 | Unregistered Abstract |
| Loughney, L., West, M. A., Moyses, H., Bates, A., Kemp, G. J., Hawkins, L., Varkonyi-Sepp, J., Burke, S., Barben, C. P., Calverley, P. M., Cox, T., Palmer, D. H., Mythen, M. G., Grocott, M. P. W., Jack, S., Fit4Surg, Grp. The effects of neoadjuvant chemoradiotherapy and an in-hospital exercise training programme on physical fitness and quality of life in locally advanced rectal cancer patients: a randomised controlled trial (The EMPOWER Trial). Perioperative Medicine. 2021. 10:12 | Protocol |
| Frawley, H. C., Galea, M., Phillips, B., Bo, K.. The effect of a physiotherapy exercise program on bladder, prolapse and bowel outcomes in women undergoing gynaecological surgery: An assessor-blinded randomised controlled trial. Neurourology and Urodynamics. 2008. 27:623-624 | Intervention Not Preoperative |
| Demark-Wahnefried, W., Nix, J. W., Hunter, G. R., Rais-Bahrami, S., Desmond, R. A., Chacko, B., Morrow, C. D., Azrad, M., Fruge, A. D., Tsuruta, Y., Ptacek, T., Tully, S. A., Segal, R., Grizzle, W. E.. Feasibility outcomes of a presurgical randomized controlled trial exploring the impact of caloric restriction and increased physical activity versus a wait-list control on tumor characteristics and circulating biomarkers in men electing prostatectomy for prostate cancer (vol 16, 61, 2016). Bmc Cancer. 2017. 17:2 | Duplicate |
| Argunova, Y. A., Zvereva, T. N., Pomeshkina, S. A., Ivanova, A. V., Polikutina, O. M., Gruzdeva, O. V., Kashtalap, V. V., Barbarash, O. L.. Optimization of a Comprehensive Prehabilitation Program for Patients with Stable Coronary Artery Disease Undergoing Elective Coronary Artery Bypass Grafting. Rational Pharmacotherapy in Cardiology. 2020. 16:508-515 | Wrong Comparator Group |
| Ogawa, M., Yoshida, N., Satomi-Kobayashi, S., Tsuboi, Y., Komaki, K., Wakida, K., Gotake, Y., Inoue, T., Tanaka, H., Yamashita, T., Sakai, Y., Izawa, K. P., Takahashi, M., Ogawa, W., Hirata, K.. Efficacy of preoperative amino acid supplements on postoperative physical function and complications in open heart surgery patients: A study protocol for a randomized controlled trial. Journal of Cardiology. 2019. 74:360-365 | Protocol |
| Barakat, H., Khan, J., Shahin, Y., McCollum, P., Chetter, I.. Randomised controlled trial of preoperative supervised exercise in patients undergoing elective abdominal aortic aneurysm repair. British Journal of Surgery. 2015. 102:1-2 | Duplicate |
| Bendz, I., Olsen, M. F.. Evaluation of immediate versus delayed shoulder exercises after breast cancer surgery including lymph node dissection - A randomised controlled trial. Breast. 2002. 11:241-248 | Wrong or Unclear Duration |
| Kuchler, T., Graul, J., Holst, K., Rappat, S., Neu, B., Linhart, D., Broelsch, C.. EVALUATION OF THE IMPACT OF A MEDICAL PSYCHOLOGICAL SUPPORT PROGRAM ON QUALITY-OF-LIFE OF CANCER-PATIENTS PRESURGICAL AND POSTSURGICAL TREATMENT - A RANDOMIZED CLINICAL-TRIAL. Quality of Life Research. 1993. 2:64-64 | Protocol |
| Doulatabad, S. N., Pirami, F., Zaboli, S., Far, A. A.. EFFECT OF KEGEL EXERCISE ON WOUND HEALING INDEX AFTER COLORECTAL SURGERY. International Journal of Life Science and Pharma Research. 2017. 7:P22-P27 | Intervention Not Preoperative |
| Carneiro, E. M., Ramos, M. D., Terra, G. A., Rodrigues, V., Matos, D., Crema, E.. Evaluation of breathing exercise in hormonal and immunological responses in patients undergoing abdominal surgery. Acta Cirurgica Brasileira. 2013. 28:385-390 | Wrong or Unclear Duration |
| Cortes, O. L., Herrera-Galindo, M., Becerra, C., Rincon-Roncancio, M., Povea-Combariza, C., Esparza-Bohorquez, M.. Preoperative walking recommendation for non-cardiac surgery patients to reduce the length of hospital stay: a randomized control trial. Bmc Sports Science Medicine and Rehabilitation. 2021. 13:11 | Wrong or Unclear Duration |
| Rodrigues, M. A., Ferreira, L. M., Calvi, E. N. D., Nahas, F. X.. Preoperative Respiratory Physiotherapy in Abdominoplasty Patients. Aesthetic Surgery Journal. 2018. 38:291-299 | Not a Randomized Trial |
| Morielli, A. R., Usmani, N., Boule, N. G., Severin, D., Tankel, K., Joseph, K., Nijjar, T., Fairchild, A., Courneya, K. S.. Feasibility, Safety, and Preliminary Efficacy of Exercise During and After Neoadjuvant Rectal Cancer Treatment: A Phase II Randomized Controlled Trial. Clinical Colorectal Cancer. 2021. 20:216-226 | Wrong Population |
| Peng, L. H., Wang, W. J., Chen, J., Jin, J. Y., Min, S., Qin, P. P.. Implementation of the pre-operative rehabilitation recovery protocol and its effect on the quality of recovery after colorectal surgeries. Chinese Medical Journal. 2021. 134:2865-2873 | Wrong Population |
| Pereira, L., Figueiredo-Braga, M., Carvalho, I. P.. Preoperative anxiety in ambulatory surgery: The impact of an empathic patient-centered approach on psychological and clinical outcomes. Patient Education and Counseling. 2016. 99:733-738 | Not Prehabilitation |
| Wong, Ting Xuan, Wong, Wei Xiang, Chen, Seong Ting, Ong, Shu Hwa, Shyam, Sangeetha, Ahmed, Nurzarina, Hamdan, Khairul Hazim, Awang, Raflis Ruzairee, Ibrahim, Mohd Razali, Palayan, Kandasami, Chee, Winnie Siew Swee. Effects of Perioperative Oral Nutrition Supplementation in Malaysian Patients Undergoing Elective Surgery for Breast and Colorectal Cancers—A Randomised Controlled Trial. Nutrients. 2022. 14:615 | Wrong or Unclear Duration |
| Moreira Barros, L&iacute;via, do Vale Gomes, Francisca Ant&ocirc;nia, Neves Carneiro, Fl&aacute;vio, Galindo Neto, Nelson Miguel, Marques Frota, Natasha, Caetano, Joselany &Aacute;fio. Knowledge and attitude of candidates to gastroplasty about perioperative: randomized clinical trial. Revista Brasileira de Enfermagem. 2020. 73:1-8 | Not Prehabilitation |
| Iskender, Mahinur Durmus, Bektas, Ozgur, Eren, Handan. Effect of preoperative in‐bed exercises and mobilization training on postoperative anxiety and mobilization level. Japan Journal of Nursing Science. 2020. 17:1-8 | Wrong or Unclear Duration |
| Erdoğan, Esra, Demir, Satı, Çalışkan, Behice Belkıs, Bayrak, Nurten Gülsüm. Effect of psychological care given to the women who underwent hysterectomy before and after the surgery on depressive symptoms, anxiety and the body image levels. Journal of Obstetrics & Gynaecology. 2020. 40:981-987 | Single Risk Factor Management |
| Jintana, Rittharomya, Suparb, Aree-ue, Pomtip, Malathum, Pisamai, Orathai, Belza, Basia, Viroj, Kawinwonggowit. The Effectiveness of Preoperative Quadriceps Exercise and Diet Control Program for Older Adults Waiting for Total Knee Arthroplasty: A Randomized Controlled Trial. Pacific Rim International Journal of Nursing Research. 2020. 24:485-501 | Wrong or no Outcome |
| Qingtong, S. H. I., Yali, Diao, Jun, Qian. Application of Single-hole Thoracoscopic Surgery Combined with ERAS Concept for Respiratory Function Exercise in Perioperative Period of Lung Cancer. Chinese Journal of Lung Cancer. 2020. 23:667-672 | Wrong or Unclear Duration |
| Ayvat, Pınar, Arslan Yurtlu, Derya, ÖZgÜRbÜZ, Uğur, GÜNtÜRkÜN, Fatma, KatircioĞLu, Kaan, Kizilkaya, Mehmet. Does Preoperative Anxiety Decrease with BATHE Method? A Prospective Randomized Study. Archives of Neuropsychiatry / Noropsikiatri Arsivi. 2020. 57:141-147 | Wrong or Unclear Duration |
| Fors, Maria, Enthoven, Paul, Abbott, Allan, Öberg, Birgitta. Effects of pre-surgery physiotherapy on walking ability and lower extremity strength in patients with degenerative lumbar spine disorder: Secondary outcomes of the PREPARE randomised controlled trial. BMC Musculoskeletal Disorders. 2019. 20:1-11 | Wrong or no Outcome |
| Liu, K., Luo, J., Shao, C., Ji, S., Xu, Y., Hu, L., Qiang, Y., Shen, Y.. PT03.04: An Enhanced Nutritional Support Pathway Including Extended Preoperative and Home Enteral Nutrition is Safe, Feasible and May Benefit Patients Undergoing Enhanced Recovery After Esophagectomy: A Pilot Randomized Clinical Trial...41st ESPEN Congress, Krakow, Poland, 31 August–3 September, 2019. Clinical Nutrition. 2019. 38:S39-S39 | Wrong Population |
| Yun, Dong, Yanjun, L. I., Zhu, Zeng, Liping, Chen. Application of social support and psychological intervention in nursing of heart transplant patients during waiting period. Chinese Nursing Research. 2019. 33:2217-2221 | Wrong or no Outcome |
| Temur, Kubra, Kapucu, Sevgisun. The effectiveness of lymphedema self-management in the prevention of breast cancer-related lymphedema and quality of life: A randomized controlled trial. European Journal of Oncology Nursing. 2019. 40:22-35 | Wrong or no Outcome |
| Medina-Garzón, Mauricio. Effectiveness of a Nursing Intervention to Diminish Preoperative Anxiety in Patients Programmed for Knee Replacement Surgery: Preventive Controlled and Randomized Clinical Trial. Investigacion & Educacion en Enfermeria. 2019. 37:1-12 | Wrong or no Outcome |
| Angus Lee, Chun Hin, Murnane, Andrew, Heriot, Alexander G., Ismail, Hilmy, Riedel, Bernhard. Randomized Pilot Study of Enhanced Structured Preoperative Exercise Program for Patients with Rectal Cancer Requiring Neoadjuvant Therapy Before Major Resection. Journal of the American College of Surgeons. 2018. 227:S73-S74 | Unregistered Abstract |
| . Boden I, Skinner EH, Browning L, et al. Preoperative physiotherapy for the prevention of respiratory complications after upper abdominal surgery: pragmatic, double blinded, multicentre randomised controlled trial. BMJ. 2018;360:j5916. AORN Journal. 2018. 108:461-467 | Not Prehabilitation |
| Loghmani, Laleh, Monfared, Mahmood Beheshti. The effect of preoperative training on postoperative depression in patients undergoing open heart surgery (2017). Electronic Journal of General Medicine. 2018. 15:1-7 | Not a Randomized Trial |
| Lin, Yu‐Hua, Lee, Su‐Ying, Su, Wei‐Ren, Kao, Chia‐Chan, Tai, Ta‐Wei, Chen, Tai‐Been. Effects of nurse‐led lower extremity strength training on knee function recovery in patients who underwent total knee replacement. Journal of Clinical Nursing (John Wiley & Sons, Inc.). 2018. 27:1836-1845 | Wrong or Unclear Duration |
| Yin, Ying, Zheng, Xuan, Liu, Shuangyu. Effect of different grip mode on vascular condition of pre operative arteriovenous fistula in forearm. Chinese Nursing Research. 2018. 32:99-102 | Not Prehabilitation |
| Zduński, Sebastian, Rongies, Witold, Ziółkowski, Marcin, Kozieł, Tomasz, Kazimierski, Piotr, Hałaj, Rafał, Sierdziński, Janusz. Ocena zakresu ruchu stawu kolanowego (ROM) oraz poziomu dolegliwości bólowych (VAA) u pacjentów po artroskopowej rekonstrukcji więzadła krzyżowego przedniego w wybranym modelu usprawniania. Advances in Rehabilitation. 2017. 31:41-54 | Duplicate |
| Dickson, Elizabeth L., Stockwell, Erica, Geller, Melissa A., Vogel, Rachel Isaksson, Mullany, Sally A., Ghebre, Rahel, Witherhoff, Boris J. N., Downs Jr, Levi S., Carson, Linda F., Teoh, Deanna, Glasgow, Michelle, Gerber, Matt, Rivard, Colleen, Erickson, Britt K., Hutchins, Jacob, Argenta, Peter A., Downs, Levi S., Jr.. Enhanced Recovery Program and Length of Stay After Laparotomy on a Gynecologic Oncology Service: A Randomized Controlled Trial. Obstetrics & Gynecology. 2017. 129:355-362 | Wrong or Unclear Duration |
| Chen, Brian, Awasthi, Rashami, Sweet, Shane, Minnella, Enrico, Bergdahl, Andreas, Santa Mina, Daniel, Carli, Francesco, Scheede-Bergdahl, Celena, Chen, Brian P., Sweet, Shane N., Minnella, Enrico M.. Four-week prehabilitation program is sufficient to modify exercise behaviors and improve preoperative functional walking capacity in patients with colorectal cancer. Supportive Care in Cancer. 2017. 25:33-40 | Wrong or no Outcome |
| Jensen, Bente, Laustsen, Sussie, Jensen, Jørgen, Borre, Michael, Petersen, Annemette, Jensen, Bente Thoft, Jensen, Jørgen Bjerggaard, Petersen, Annemette Krintel. Exercise-based pre-habilitation is feasible and effective in radical cystectomy pathways-secondary results from a randomized controlled trial. Supportive Care in Cancer. 2016. 24:3325-3331 | Wrong or no Outcome |
| Gómez‐Urquiza, Jose L., Hueso‐Montoro, César, Urquiza‐Olmo, Josefa, Ibarrondo‐Crespo, Rocío, González‐Jiménez, Emilio, Schmidt‐Riovalle, Jacqueline. A randomized controlled trial of the effect of a photographic display with and without music on pre-operative anxiety. Journal of Advanced Nursing (John Wiley & Sons, Inc.). 2016. 72:1666-1676 | Wrong or Unclear Duration |
| Saw, M. M., Kruger-Jakins, T., Edries, N., Parker, R.. Significant improvements in pain after a six-week physiotherapist-led exercise and education intervention, in patients with osteoarthritis awaiting arthroplasty, in South Africa: a randomised controlled trial. BMC Musculoskeletal Disorders. 2016. 17:1-14 | Wrong or no Outcome |
| Webster, Joan, Osborne, Sonya Ranee, Gill, Richard, Chow, Carina Faran Kalan, Wallin, Siobhan, Jones, Lee, Tang, Annie. Does Preoperative Oral Carbohydrate Reduce Hospital Stay? A Randomized Trial. AORN Journal. 2014. 99:233-242 | Wrong or Unclear Duration |
| Kekecs, Zoltán, Jakubovits, Edit, Varga, Katalin, Gombos, Katalin. Effects of patient education and therapeutic suggestions on cataract surgery patients: A randomized controlled clinical trial. Patient Education & Counseling. 2014. 94:116-122 | Wrong or Unclear Duration |
| Mitsuyoshi, Okazaki, Satoshi, Matsukuma, Ryuichiro, Suto, Kensuke, Miyazaki, Masaaki, Hidaka, Mitsutoshi, Matsuo, Shinji, Noshima, Nobuya, Zempo, Takashi, Asahara, Koji, Nomoto. Perioperative synbiotic therapy in elderly patients undergoing gastroenterological surgery: A prospective, randomized control trial. Nutrition. 2013. 29:1224-1230 | Not Prehabilitation |
| Modarres, Maryam, Rahimikian, Fatemeh, Mehran, Abbas. Impact of Pre-Hysterectomy Counseling on Depression among Patients Referred to TUMS Hospitals. HAYAT. 2013. 19:40-50 | Wrong or no Outcome |
| Dettling, Daniela S., Schaaf, Marike, Blom, Rachel L. G. M., Nollet, Frans, Busch, Olivier R. C., Berge Henegouwen, Mark I.. Feasibility and Effectiveness of Pre-operative Inspiratory Muscle Training in Patients Undergoing Oesophagectomy: A Pilot Study. Physiotherapy Research International. 2013. 18:16-26 | Not a Randomized Trial |
| Jie, Bin, Jiang, Zhu-Ming, Nolan, Marie T., Zhu, Shai-Nan, Yu, Kang, Kondrup, Jens. Impact of preoperative nutritional support on clinical outcome in abdominal surgical patients at nutritional risk. Nutrition. 2012. 28:1022-1027 | Not Prehabilitation |
| Lee, Kwo-Chen, Chao, Yuh-Huey, Yiin, Jia-Jean, Chiang, Pei-Yi, Chao, Yann-Fen. Effectiveness of different music-playing devices for reducing preoperative anxiety: A clinical control study. International Journal of Nursing Studies. 2011. 48:1180-1187 | Wrong or Unclear Duration |
| Savci, S., Degirmenci, B., Saglam, M., Arikan, H., Inal-Ince, D., Turan, H. N., Demircin, M.. Short-term effects of inspiratory muscle training in coronary artery bypass graft surgery: A randomized controlled trial. Scandinavian Cardiovascular Journal. 2011. 45:286-293 | Wrong or Unclear Duration |
| Carbajo, M. A., Castro, M. J., Kleinfinger, S., Gómez-Arenas, S., Ortiz-Solórzano, J., Wellman, R., García-Ianza, C., Luque, E.. Effects of a balanced energy and high protein formula diet (Vegestart complet(R)) vs. low-calorie regular diet in morbid obese patients prior to bariatric surgery (laparoscopic single anastomosis gastric bypass): a prospective, double-blind randomized study. Nutricion Hospitalaria. 2010. 25:939-948 | Wrong or no Outcome |
| Nava, S., Culp, W. C., Jr., Beyer, E. A., Takagi, H., Kawai, N., Umemoto, T., Hulzebos, E. H. J., Helders, P. J. M., Favié, N. J., De Bie, R. A., de la Riviere, A. B., Van Meeteren, N. L. U., Culp, William C., Jr., Beyer, Erik A.. Preoperative inspiratory muscle training and postoperative complications...Hulzebos EH, Helders PJ, Favié NJ et al. Preoperative intensive inspiratory muscle training to prevent postoperative pulmonary complications in high-risk patients undergoing CABG surgery: a randomized clinical trial. JAMA. 2006;296:1851-1857. . 2007. 297:697-699 | Duplicate |
| McCarthy, M. A. S.. Perioperative immunonutrition in head and neck cancer: a feasibility study. . 2006. Ph.D.:160 p-160 p | Wrong or Unclear Duration |
| Wang, Y., Wu, H., Pan, Q.. A study on effect of psychological intervention for gynecological preoperative patients. Chinese Nursing Research. 2004. 18:1548-1550 | Wrong or Unclear Duration |
| Li, S.. Applying Chinese classical music to treat preoperative anxiety of patients with gastric cancer. Chinese Nursing Research. 2004. 18:471-472 | Wrong or no Outcome |
| McRee, L. D., Noble, S., Pasvogel, A.. Using massage and music therapy to improve postoperative outcomes. AORN Journal. 2003. 78:433-447 | Wrong or Unclear Duration |
| Brosnahan, J.. Supplementation with key nutrients reduced postoperative infections and length of hospital stay after gastrointestinal surgery. Evidence Based Nursing. 2003. 6:47-47 | Wrong or Unclear Duration |
| Nijveldt, R. J., Houdijk, A. P. J., Boelens, P. G., van Leeuwen, P. A. M., Weyandt, D., Das, U. N., Tepaske, R.. Nutritional supplementation after cardiac surgery...Tepaske R, te Velthuis H, Oudemans-van Straaten HM et al. Effect of preoperative oral immune-enhancing nutritional supplement on patients at high risk of infection after cardiac surgery: a randomised placebo-controlled trial. Lancet 2001; 358:696-701. Lancet. 2002. 359:256-258 | Not a Randomized Trial |
| Beck, C. T.. Commentary on Preadmission self-instruction effects on postadmission and postoperative indicators in CABG patients: partial replication and extension [original article by Rice VH et al appears in RES NURS HEALTH 1992;15(4):253-9]. Nursing Scan in Research. 1993. 6:11-11 | Wrong or Unclear Duration |
| Furrer, Marc A., Huesler, Juerg, Fellmann, Adrian, Burkhard, Fiona C., Thalmann, George N., Wuethrich, Patrick Y.. The Comprehensive Complication Index CCI: A proposed modification to optimize short-term complication reporting after cystectomy and urinary diversion. Urologic Oncology. 2019. 37:291.e9-291.e18 | Not a Randomized Trial |
| . Prehabilitation Feasibility Among Older Adults Undergoing Transplantation. Feasibility of a Prehabilitation Intervention Among Older Adults With Myeloma Receiving Autologous Stem Cell Transplant. 2022. | No Results |
| . Effects of preoperative Respiratory Muscle Training for improvement of postoperative Health Related Quality of Life in Mitral Valve Replacement patients&rdquo. . 2022. | No Results |
| . comparative effect of preoperative resistance and aerobic training in pre frail to moderately frail patients for improving quality of recovery in post coronary artery bypass grafting surgery patients. . 2022. | No Results |
| . Prehabilitation in Prostate Cancer Patients, TelePrehabTrial. Prehabilitation in Prostate Cancer Patients Undergoing Nerve Sparring Robot Assisted Radical Prostatectomy. 2022. | No Results |
| . To see the efficacy of Oral Vitamin D before surgery in controlling low calcium levels after surgery. Impact of preoperative vitamin D supplementation on early post operative hypocalcemia in patients with vitamin D deficiency undergoing hemithyroidectomy. 2022. | No Results |
| . The Effect of Progressive Relaxation Exercises on Bariatric Surgery Period. The Effect of Progressive Relaxation Exercises on Preoperative Anxiety and Postoperative Pain in Bariatric Surgery Patients. 2022. | No Results |
| . Impact of a Multimodal Prehabilitation Program Before Robotic-assisted Radical Prostatectomy. Impact of a Multimodal Prehabilitation Program Before Robotic-assisted Radical Prostatectomy. A Randomized Clinical Trial. 2022. | No Results |
| . Effect of preoperative rehabilitation on clinical outcomes after rotator cuff repair. . 2022. | No Results |
| . Effects of Segmental Breathing Exercise in Patients Awaiting Coronary Artery Bypass Graft Surgery (CABG). Effects of Preoperative Segmental Breathing Exercise on Postoperative Pulmonary Complications in Patients Awaiting Coronary Artery Bypass Graft Surgery (CABG). 2022. | No Results |
| . Effect of preoperative group cognitive behavior counseling on anesthesia and postoperative psychology of patients undergoing painless abortion. . 2022. 38:1650 | Wrong Population |
| . Enhanced Recovery After Surgery (ERAS) Pathway in Patients Undergoing Robot-Assisted Laparoscopic Radical Prostatectomy. Impact of Enhanced Recovery After Surgery (ERAS) Pathway on Outcomes in Patients Undergoing Robot-Assisted Laparoscopic Radical Prostatectomy: a Randomized Controlled Trial. 2022. | No Results |
| . Impact of improving nutrition, increasing endurance and reducing anxiety on post-operative outcomes in patients planned for major abdominal surgeries. IMPACT OF NEW REGIME AS PRE-HABILATATION ON PATIENTS UNDERGOING MAJOR GASTROINTESTINAL SURGERIES: a PROSPECTIVE RANDOMISED STUDY. 2022. | No Results |
| . PrehabPal: a Digital Tool to Help Older Adults Prepare for Cancer Surgery. PrehabPal: a Digital Tool to Help Frail Elders Prepare for Cancer Surgery. 2022. | No Results |
| . Inspiratory Muscle Training and Pulmonary Function in Patients Submitted to Bariatric Surgery. Pre-operative Inspiratory Muscle Training Effectiveness and Pulmonary Function in Patients Submitted to Bariatric Surgery. 2022. | No Results |
| . Effects of prehabilitation before anterior cruciate ligament reconstruction on functional outcomes during rehabilitation - a randomized controlled trial. Effects of prehabilitation before anterior cruciate ligament reconstruction on functional outcomes during rehabilitation - a randomized controlled trial - PRAI (Preoperative Rehabilitation after ACL-Injury). 2022. | No Results |
| . Joint PREP: joint PRehabilitation with Exercise and Protein. A randomised controlled feasibility trial of a prehabilitation intervention in frail older people undergoing total hip or knee replacement. 2022. | No Results |
| . The effects of Phoniatric PREhabilitation in Head and Neck Cancer patients on Aspiration and Preservation of Swallowing. The effects of Phoniatric PREhabilitation in Head and Neck Cancer patients on Aspiration and Preservation of Swallowing - PREHAPS. 2022. | No Results |
| . Individualized Prehabilitation for Enhancing Recovery and Surgical Outcomes in Patients Undergoing Radiotherapy and Surgery for Soft Tissue Sarcoma. Pre-Operative ERAS&reg; (Enhanced Recovery After Surgery): randomized Feasibility Trial of Implementing Individualized Prehabilitation for People Undergoing Neo-Adjuvant Radiotherapy and Lower Limb Soft-Tissue Sarcoma Surgery. 2022. | No Results |
| . The Impact of Cardiac Prehabilitation on Clinical Outcomes: a comparison between Surgical Aortic Valve Replacement (sAVR) and Transcatheter Aortic Valve Implantation (TAVI). The Impact of Cardiac Prehabilitation on Clinical Mental Health Outcomes: a comparison between Surgical Aortic Valve Replacement (sAVR) and Transcatheter Aortic Valve Implantation (TAVI). 2022. | No Results |
| . Assessing the effect of preoperative rehabilitation (prehab) program on physical function, balance and fear of fall of patients candidate for coronary artery bypass graft surgery. . 2022. | No Results |
| . Home-based Preoperative Exercise Training for Lung Cancer Patients Undergoing Surgery. Effect of Home-based Preoperative Exercise Training on Quality of Life After Lung Cancer Surgery: a Randomized Controlled Trial. 2022. | Protocol |
| . Pre-operative Prehabilitation in Cancer Surgery - Objective Recovery Assessment. . 2022. | No Results |
| . Effect of multimodal prehabilitation on surgical outcomes in obese women undergoing open elective gynecological surgeries: a pilot study. Effect of multimodal prehabilitation on surgical outcomes in obese women undergoing open elective gynecological surgeries using midline vertical incision: a pilot study. 2022. | No Results |
| . Exercise, nutrition and mental well being support in reducing complications after surgery for cancer of ovary. Pre-habilitation in ovarian cancer cytoreductive surgery and its effect on postoperative morbidity (PROSPERITY): a randomized controlled trial - PROSPERITY. 2022. | Protocol |
| . A randomised clinical trial comparing preoperative exercise in the home, hospital, and community with standard care in adults awaiting for major abdominal surgery. . 2022. | Protocol |
| . Efficacy of novel preoperative carbohydrate containing standardized ginger extract intake in brain tumors surgery. . 2022. | No Results |
| . Influence of daily preoperative step volume and preoperative incentive spirometry training on pulmonary complications after upper abdominal cancer surgery: a randomized controlled trial. Influence of daily preoperative step volume and preoperative incentive spirometry training on pulmonary complications after upper abdominal cancer surgery: a randomized controlled trial - PREVIST Trial. 2022. | No Results |
| . A Prehab Strengthening Program Prior to ACL Surgery on Lower Limb Structure and Function. The Effects of a Lower Limb Strengthening Training Program During Prehabilitation Prior to ACL Surgery on Lower Limb Structure and Function : a Randomized Clinical Trial. 2022. | No Results |
| . Evaluation of the Impact of Prehabilitation on Recovery Following Open Surgery for Abdominal Aortic Aneurysm. Efficacy of Pre-Operative Prehabilitation in Patients Undergoing Open Surgery for Abdominal Aortic Aneurysm. 2022. | No Results |
| . Additive effect of Roux-en Y Gastric Bypass to preoperative Very low calorie diet on non-alcoholic fatty liver diseases (NAFLD, NASH) - A randomized controlled trial. Additive effect of Roux-en Y Gastric Bypass to preoperative Very low calorie diet on non-alcoholic fatty liver diseases (NAFLD, NASH) - A randomized controlled trial - NASH IN RYGB. 2022. | Protocol |
| . Preoperative exercise training to increase the postoperative, functional capacity of patients with diverticulitis - a randomised controlled study. . 2022. | Protocol |
| . Pre-operative Inspiratory Muscle Strength Training in Total Joint Surgery. Preoperative Inspiratory Muscle Strength Training and Pulmonary Complications After Surgery. 2022. | No Results |
| . High Intensity PreHab Before Major Abdominal Surgery. Effect of a Two Week Prehabilitation Program Before Major Abdominal Surgery. 2022. | Not a Randomized Trial |
| . A randomized controlled study for the usefulness of prehabilitation health care application (PreHA) in patients undergoing gastrointestinal surgery. A randomized controlled study for the usefulness of prehabilitation health care application (PreHA) in patients undergoing gastrointestinal surgery - A randomized controlled study for the usefulness of prehabilitation health care application (PreHA). 2022. | No Results |
| . Randomized control test to evaluate the usefulness of device using for the management of pre-habilitation before surgery for the patients who undergoing neoadjuvant chemotherapy. Randomized control test to evaluate the usefulness of device using for the management of pre-habilitation before surgery for the patients who undergoing neoadjuvant chemotherapy - Randomized control test to evaluate the usefulness of device using for the. 2022. | No Results |
| . Abdominal muscle training prior to surgery, to reduce the occurrence of postoperative pulmonary complications in postoperative cardiac surgery patients compared to standard care : a randomized control trial. Physiotherapy led Preoperative Abdominal Muscle Training to Prevent Postoperative Respiratory Complications following Cardiac Surgery. 2022. | No Results |
| . A Study on the Effect of Protein Supplementation in Perioperative Exercise Therapy. . 2022. | No Results |
| . Pre-habilitation Interventions to Empower Patients With Chronic Pain. . 2022. | No Results |
| . Deep Breathing Exercise With Incentive Spirometer Started in the Preoperative Period. The Effect of Deep Breathing Exercise With Incentive Spirometer Started in the Preoperative Period in Patients Undergoing Open Cardiac Surgery on Pulmonary Function and Complications: a Randomized Controlled Study. 2022. | No Results |
| . Virtual Mind-Body Exercises for People Having Pancreatic Surgery. Pilot Study of Virtual Mind-Body Exercises for Patients Undergoing Pancreatic Surgery. 2022. | No Results |
| . Participating in Tai Chi to Reduce Anxiety and Keep up Physical Function. Participating in Tai Chi to Reduce Anxiety and Keep up Physical Function: implementing a Prehabilitation Intervention for Radical Prostatectomy. 2022. | No Results |
| . Multidimensional Prehabilitation in Pancreatic Surgery for Pancreatic and Periampullary Neoplasms. Randomized Clinical Trial Investigating Multidimensional Prehabilitation in Pancreatic Surgery for Patients With Pancreatic and Periampullary Neoplasms. 2022. | No Results |
| . The Effect of Exercise in Patients Awaiting Bariatric Surgery. The Effect of a Preoperative Exercise Programme on Cardiorespiratory Fitness, Resting Metabolic Rate and Autonomic Control in Patients With Severe Obesity Awaiting Bariatric Surgery. 2022. | No Results |
| . Prehabilitation for Breast Cancer Surgery. Prehabilitation Program Based on Health Education and Nordic Walking to Reduce Musculoskeletal Impairments in Women Undergoing Breast Cancer Surgery. 2022. | No Results |
| . The Effect of Mobile-based Education and Exercise Program Given to Patients With Total Knee Replacement Surgery. The Effect of Mobile-based Education and Exercise Program Given to Patients With Total Knee Replacement Surgery on Anxiety, Kinesiophobia, and Physical Function. 2022. | No Results |
| . Physical Exercise During Preoperative Chemotherapy for Breast Cancer. Physical Exercise During Neoadjuvant Chemotherapy for Breast Cancer as a Means to Increase Pathological Complete Response Rates: the Randomized Neo-ACT Trial. 2022. | No Results |
| . Nutrition and Exercise Prehabilitation to Reduce Morbidity Following Major Liver Surgery in Sarcopenic Patients. Nutrition and EXercise Prehabilitation to Reduce Morbidity (NEXPREM) Following Major Liver Surgery in Sarcopenic Patients. 2022. | Protocol |
| . Exercise Prehabilitation in Patients With Head and Neck Squamous-cell Carcinoma: the FIT4TREAT Trial. . 2022. | No Results |
| . Multidisciplinary Prehabilitation and Postoperative Rehabilitation in Patients Undergoing Resection of Colon Cancer. Multidisciplinary Prehabilitation and Postoperative Rehabilitation for Avoiding Complications in Patients Undergoing Resection of Colon Cancer: ONCOFIT Study. 2022. | No Results |
| . Effect of a Physiotherapy programme in abdominal surgery patients. Effect of a structured Physiotherapy programme in abdominal surgery patients: a randomized controlled trial. 2022. | No Results |
| . Prehab Intervention in Patients Awaiting Total Knee Arthroplasty (TKA). Effect of Preoperative Training and Education on Pre- and Postoperative Functional Performance in Patients Awaiting TKA; A Randomized Controlled Trial - a Pilot Study. 2022. | No Results |
| . A Pilot Study to Test the Feasibility of a Hybrid Preoperative Physical Therapy Intervention for Patients Undergoing Total Joint Arthroplasty. . 2022. | No Results |
| . Perioperative Exercise and Nutritional Optimisation Prehabilitation Before Surgery for Patients With Peritoneal Malignancy. The PANO Trial: perioperative Exercise and Nutrition Optimisation Prehabilitation in Cancer Patients With Peritoneal Malignancy. 2022. | No Results |
| . Multimodal Prehabilitation To Improve The Clinical Outcomes Of Frail Elderly Patients With Gastric Cancer. Multimodal Prehabilitation To Improve The Clinical Outcomes Of Frail Elderly Patients With Gastric Cancer: a Multicenter Randomized Controlled Trial. 2022. | No Results |
| Aily, J. B., Voinier, D., Jakiela, J., Bye, T., Master, H., Thoma, L., White, D.. Does Exercise before Surgery Predict Trajectories of Physical Activity after Knee Replacement?. Arthritis and Rheumatology. 2022. 74:3765-3766 | Not a Randomized Trial |
| Angus Lee, C. H., Murnane, A., Heriot, A. G., Ismail, H., Riedel, B.. Randomized Pilot Study of Enhanced Structured Preoperative Exercise Program for Patients with Rectal Cancer Requiring Neoadjuvant Therapy before Major Resection. Journal of the American College of Surgeons. 2018. 227:S73-S74 | Wrong or Unclear Duration |
| Argunova, Yulia, Belik, Ekaterina, Gruzdeva, Olga, Ivanov, Sergey, Pomeshkina, Svetlana, Barbarash, Olga. Effects of Physical Prehabilitation on the Dynamics of the Markers of Endothelial Function in Patients Undergoing Elective Coronary Bypass Surgery. Journal of personalized medicine. 2022. 12: | Wrong or Unclear Duration |
| Au, Darren, Matthew, Andrew G., Lopez, Paty, Hilton, William J., Awasthi, Rashami, Bousquet-Dion, Guillaume, Ladha, Karim, Carli, Franco, Santa Mina, Daniel. Prehabilitation and acute postoperative physical activity in patients undergoing radical prostatectomy: a secondary analysis from an RCT. Sports Medicine - Open. 2019. 5:N.PAG-N.PAG | Duplicate |
| Ausania, F., Senra, P., Melendez, R., Caballeiro, R., Ouvina, R., Casal-Nunez, E.. Prehabilitation in patients undergoing pancreaticoduodenectomy: A randomized controlled trial. Revista Espanola de Enfermedades Digestivas. 2019. 111:603-608 | Duplicate |
| Aybar, P. E. S., Parpia, S., Simunovic, M., Duceppe, E., Pinto-Sanchez, M. I., Bhandari, M., Levine, M. N.. Perioperative optimization with nutritional supplements in patients undergoing gastrointestinal surgery for cancer: a randomized, placebo controlled feasibility clinical trial. . 2022. 40: | Duplicate |
| Bakhshi, M., Ranjbar, H., Rostami, O., Chamanzari, H., Bahrami-Taghanaki, H.. Effect of Foot Reflexology and Gentle Stretching Exercises on Pain Intensity in Patients after Spine Surgery: a Randomized Controlled Trial. . 2022. 12:32 | Intervention Not Preoperative |
| Banerjee, Srijit, Manley, Kate, Shaw, Barnabas, Lewis, Liane, Cucato, Gabriel, Mills, Robert, Rochester, Mark, Clark, Allan, Saxton, John M.. Vigorous intensity aerobic interval exercise in bladder cancer patients prior to radical cystectomy: a feasibility randomised controlled trial. Supportive Care in Cancer. 2018. 26:1515-1523 | Duplicate |
| Berkel, A. E. M., Bongers, B. C., Kotte, H., Weltevreden, P., De Jongh, F. H. C., Eijsvogel, M. M. M., Wymenga, M., Bigirwamungu-Bargeman, M., Van Der Palen, J., Van Det, M. J., Van Meeteren, N. L. U., Klaase, J. M.. Effects of Community-based Exercise Prehabilitation for Patients Scheduled for Colorectal Surgery With High Risk for Postoperative Complications: Results of a Randomized Clinical Trial. Annals of Surgery. 2022. 275:E299-E306 | Duplicate |
| Berkel, Annefleur E. M., Bongers, Bart C., van Meeteren, Nico L. U., Klaase, Joost M.. Response to the Comments of Onerup et al and Lu and Song on: "Effects of Community-based Exercise Prehabilitation for Patients Scheduled for Colorectal Surgery With High Risk for Postoperative Complications: Results of a Randomized Clinical Trial". Annals of surgery. 2022. 276:e1126-e1128 | Not a Randomized Trial |
| Bernardi, K., Olavarria, O. A., Dhanani, N. H., Lyons, N., Holihan, J. L., Cherla, D. V., Berger, D. H., Ko, T. C., Kao, L. S., Liang, M. K.. Two-year Outcomes of Prehabilitation Among Obese Patients With Ventral Hernias: a Randomized Controlled Trial (NCT02365194). . 2022. 275:288 | Duplicate |
| Bruce, Julie, Mazuquin, Bruno, Mistry, Pankaj, Rees, Sophie, Canaway, Alastair, Hossain, Anower, Williamson, Esther, Padfield, Emma J., Lall, Ranjit, Richmond, Helen, Chowdhury, Loraine, Lait, Clare, Petrou, Stavros, Booth, Katie, Lamb, Sarah E., Vidya, Raghavan, Thompson, Alastair M.. Exercise to prevent shoulder problems after breast cancer surgery: the PROSPER RCT. Health technology assessment (Winchester, England). 2022. 26:1-124 | Intervention Not Preoperative |
| Burrows, B., King, A., Morgan, A., Wilund, K. R.. Prehabilitative Virtual Reality Mindfulness and Personalized Physical Activity for Hemodialysis Patients With Depressive Symptoms: a Feasibility Study. . 2022. 33:282 | Wrong or no Outcome |
| Byun, Hayoung, Jang, Yunjeong, Kim, Ju-Yeon, Kim, Jae-Myung, Lee, Chang Han. Effects of preoperative personal education on shoulder function and lymphedema in patients with breast cancer: A consort. Medicine. 2022. 101:e30810 | Wrong or Unclear Duration |
| Carender, Christopher N., Anthony, Christopher A., Rojas, Edward O., Noiseux, Nicolas O., Bedard, Nicholas A., Brown, Timothy S.. Perioperative Opioid Counseling Reduces Opioid Use Following Primary Total Joint Arthroplasty. The Iowa orthopaedic journal. 2022. 42:169-177 | Wrong or no Outcome |
| Carli, F., Bousquet-Dion, G., Awasthi, R., Elsherbini, N., Liberman, S., Boutros, M., Stein, B., Charlebois, P., Ghitulescu, G., Morin, N., Jagoe, T., Scheede-Bergdahl, C., Minnella, E. M., Fiore, J. F., Resnick, M. J.. Re: Effect of Multimodal Prehabilitation vs Postoperative Rehabilitation on 30-Day Postoperative Complications for Frail Patients Undergoing Resection of Colorectal Cancer: A Randomized Clinical Trial. Journal of Urology. 2020. 204:869-870 | Not a Randomized Trial |
| Chaudhary, Narendra Kumar, Sunuwar, Dev Ram, Sharma, Rachit, Karki, Mandeep, Timilsena, Mukti Nath, Gurung, Anita, Badgami, Sunil, Singh, Devendra Raj, Karki, Prabesh, Bhandari, Kailash Kumar, Pradhan, Pranil Man Singh. The effect of pre-operative carbohydrate loading in femur fracture: a randomized controlled trial. BMC musculoskeletal disorders. 2022. 23:819 | Wrong or Unclear Duration |
| Chen, Jing, Peng, Li-Hua, Min, Su. Implementation of perioperative breathing exercises and its effect on postoperative pulmonary complications and long-term prognosis in elderly patients undergoing laparoscopic colorectal surgery: A randomized controlled trial. Clinical rehabilitation. 2022. 36:1229-1243 | Wrong or Unclear Duration |
| Chiewhatpong, Phasawee, Charoenkwan, Kittipat, Smithiseth, Kannika, Lapisatepun, Warangkana, Lapisatepun, Panuwat, Phimphilai, Mattabhorn, Muangmool, Tanarat, Cheewakriangkrai, Chalong, Suprasert, Prapaporn, Srisomboon, Jatupol. Effectiveness of enhanced recovery after surgery protocol in open gynecologic oncology surgery: A randomized controlled trial. International journal of gynaecology and obstetrics: the official organ of the International Federation of Gynaecology and Obstetrics. 2022. 159:568-576 | Wrong or Unclear Duration |
| Chmelo, Jakub, Phillips, Alexander W., Greystoke, Alastair, Charman, Sarah J., Avery, Leah, Hallsworth, Kate, Welford, Jenny, Cooper, Matthew, Sinclair, Rhona C. F.. A feasibility trial of prehabilitation before oesophagogastric cancer surgery using a multi-component home-based exercise programme: the ChemoFit study. Pilot and feasibility studies. 2022. 8:173 | Not a Randomized Trial |
| Czech, Oliver, Siewierska, Katarzyna, Krzywinska, Aleksandra, Skorniak, Jakub, Maciejczyk, Adam, Matkowski, Rafal, Szczepanska-Gieracha, Joanna, Malicka, Iwona. Virtual Therapy Complementary Prehabilitation of Women Diagnosed with Breast Cancer-A Pilot Study. International journal of environmental research and public health. 2022. 20: | Intervention Not Preoperative |
| Duan, Siyu, Liao, Yan, Tang, Yujie, Zhang, Bin, Peng, Mingchao, Tong, Jianbin, Ouyang, Wen, Le, Yuan. Short-term perioperative cognitive therapy combined with rehabilitation exercise reduces the incidence of neurocognitive disorder in elderly patients: a randomized controlled trial. Minerva anestesiologica. 2022. 88:145-155 | Wrong or Unclear Duration |
| Fatima, Tehreem, Shakoor, Aiman, Ilyas, Mahnoor, Safdar, Maryam, Majeed, Sidra. Effectiveness of preoperative stretchings on postoperative shoulder function in patients undergoing mastectomy. JPMA. The Journal of the Pakistan Medical Association. 2022. 72:625-628 | Not Prehabilitation |
| Fors, Maria, Oberg, Birgitta, Lindback, Yvonne, Enthoven, Paul, Abbott, Allan. What mediates treatment effects in a presurgery physiotherapy treatment in surgical candidates with degenerative lumbar spine disorders? A mediation and conditional process analysis of the PREPARE randomized controlled trial. The Clinical Journal of Pain. 2021. 37:168-176 | Not a Randomized Trial |
| Fung, Pui Lam Polly, Lau, Vivian Nga Man, Ng, Floria Fung, Leung, Wing Wa, Mak, Tony Wing Chung, Lee, Anna. Perioperative changes in haemoglobin and ferritin concentrations from preoperative intravenous iron isomaltoside for iron deficiency anaemia in patients with colorectal cancer: A pilot randomised controlled trial. PloS one. 2022. 17:e0270640 | Single Risk Factor Management |
| Furyk, Claire, Senthuran, Siva, Nye, Dia, Ho, Yik H., Leicht, Anthony S.. Prehabilitation for Frail Patients Undergoing Colorectal Surgery: Lessons Learnt From a Randomised Feasibility Study. Frontiers in rehabilitation sciences. 2021. 2:650835 | Wrong or no Outcome |
| Gloor, S., Misirlic, M., Frei-Lanter, C., Herzog, P., Muller, P., Schafli-Thurnherr, J., Lamdark, T., Schregel, D., Wyss, R., Unger, I., Gisi, D., Greco, N., Mungo, G., Wirz, M., Raptis, D. A., Tschuor, C., Breitenstein, S.. Prehabilitation in patients undergoing colorectal surgery fails to confer reduction in overall morbidity: results of a single-center, blinded, randomized controlled trial. Langenbeck's Archives of Surgery. 2022. 407:897-907 | Duplicate |
| Gravier, F. E., Smondack, P., Boujibar, F., Prieur, G., Medrinal, C., Combret, Y., Muir, J. F., Baste, J. M., Cuvelier, A., Debeaumont, D., Bonnevie, T.. Prehabilitation sessions can be provided more frequently in a shortened regimen with similar or better efficacy in people with non-small cell lung cancer: a randomised trial. . 2022. 68:43 | Duplicate |
| Guo, N., Li, N., Cai, C., Pan, Z., Liu, K.. Bundle of care promotes arteriovenous fistula maturity in patients with end-stage kidney disease. . 2022. | Not a Randomized Trial |
| Halliday, Laura J., Boshier, Piers R., Doganay, Emre, Wynter-Blyth, Venetia, Buckley, John P., Moorthy, Krishna. The effects of prehabilitation on body composition in patients undergoing multimodal therapy for esophageal cancer. Diseases of the esophagus : official journal of the International Society for Diseases of the Esophagus. 2022. #volume#:#pages# | Not a Randomized Trial |
| Halliday, Laura J., Boshier, Piers R., Doganay, Emre, Wynter-Blyth, Venetia, Buckley, John P., Moorthy, Krishna. The effects of prehabilitation on body composition in patients undergoing multimodal therapy for esophageal cancer. Diseases of the esophagus : official journal of the International Society for Diseases of the Esophagus. 2023. 36:#pages# | Not a Randomized Trial |
| Hamad, A., Zhang, H., Huang, H., Ejaz, A., Tsung, A.. Understanding the mechanism behind preoperative exercise therapy in patients with gastrointestinal cancers: A prospective, randomized clinical trial. Journal of Clinical Oncology. 2022. 40: | Wrong or no Outcome |
| Hanley, Adam W., Gililland, Jeremy, Erickson, Jill, Pelt, Christopher, Peters, Christopher, Rojas, Jamie, Garland, Eric L.. Brief preoperative mind-body therapies for total joint arthroplasty patients: A randomized controlled trial. Pain. 2021. 162:1749-1757 | Wrong or Unclear Duration |
| Haque, Adam, Wisely, Nicholas, McCollum, Charles. Editor's Choice - The Abdominal Aortic Aneurysm Get Fit Trial: A Randomised Controlled Trial of Exercise to Improve Fitness in Patients with Abdominal Aortic Aneurysm. European journal of vascular and endovascular surgery : the official journal of the European Society for Vascular Surgery. 2022. 64:309-319 | Intervention Not Preoperative |
| Hardy, Krista, Kwok, Karen, Bouchard, Danielle R., Bharti, Neha, Gamey, Dean, Vergis, Ashley. Impact of a Preoperative Exercise Program on General Fitness in Patients Awaiting Bariatric Surgery: A Pilot Randomized Trial. Cureus. 2022. 14:e22566 | Wrong or no Outcome |
| He, Yue, Tang, Xiumei, Ning, Ning, Chen, Jiali, Li, Peifang, Kang, Pengde. Effects of Preoperative Oral Electrolyte-Carbohydrate Nutrition Supplement on Postoperative Outcomes in Elderly Patients Receiving Total Knee Arthroplasty: A Prospective Randomized Controlled Trial. Orthopaedic surgery. 2022. 14:2535-2544 | Wrong or Unclear Duration |
| Heil, T., Verdaasdonk, E., Maas, H., Van Munster, B., Olde Rikkert, M., De Wilt, H., Melis, R.. Improved postoperative outcomes after prehabilitation for colorectal cancer surgery in older patients: An emulated target trial. Colorectal Disease. 2022. 24:30 | Not a Randomized Trial |
| Heil, Thea C., Verdaasdonk, Emiel G. G., Maas, Huub A. A. M., van Munster, Barbara C., Olde Rikkert, Marcel G. M., de Wilt, Johannes H. W., Melis, Rene J. F.. ASO Visual Abstract: Improved Postoperative Outcomes After Prehabilitation for Colorectal Cancer Surgery in Older Patients: An Emulated Target Trial. Annals of surgical oncology. 2023. 30:257-258 | Wrong or Unclear Duration |
| Heiman, J., Onerup, A., Wessman, C., Olofsson Bagge, R.. Recovery after breast cancer surgery following recommended pre and postoperative physical activity: (PhysSURG-B) randomized clinical trial. British Journal of Surgery. 2021. 108:32-39 | Duplicate |
| Hernon, J., Saxton, J., Jones, M., Howard, G., Swart, A. M., Clark, A., Stirling, S., Turner, D., Murdoch, J., Nortje, J., Bach, S., Fearnhead, N., Din, F., Stephens, A., Lund, J., Tou, S., Kelly, S., Ziprin, P., Dennis, R., Smart, N., Roxburgh, C., Simpson, A., Mishra, A., Knight, K., Dresser, K.. SupPoRtive Exercise Programmes for Accelerating REcovery after major ABdominal Cancer surgery trial (PREPARE-ABC): Pilot phase of a multicentre randomised controlled trial. Colorectal Disease. 2021. 23:3008-3022 | Duplicate |
| Ho, C. J., Chen, Y. T., Wu, H. L., Huang, H. T., Lin, S. Y.. The Effects of a Patient-Specific Integrated Education Program on Pain, Perioperative Anxiety, and Functional Recovery following Total Knee Replacement. . 2022. 12: | Wrong or Unclear Duration |
| Horn, Nicole, Laferton, Johannes A. C., Shedden-Mora, Meike C., Moosdorf, Rainer, Rief, Winfried, Salzmann, Stefan. Baseline depressive symptoms, personal control, and concern moderate the effects of preoperative psychological interventions: the randomized controlled PSY-HEART trial. Journal of behavioral medicine. 2022. 45:350-365 | Wrong or Unclear Duration |
| Humeidan, Michelle L., Otey, Andrew, Zuleta-Alarcon, Alix, Mavarez-Martinez, Ana, Stoicea, Nicoleta, Bergese, Sergio. Perioperative Cognitive Protection—Cognitive Exercise and Cognitive Reserve (The Neurobics Trial): A Single-blind Randomized Trial. Clinical Therapeutics. 2015. 37:2641-2650 | No Results |
| Iannelli, Antonio, Fontas, Eric, Grec, Laurence, Nocca, David, Robert, Maud, Schiavo, Luigi, Schneck, Anne-Sophie. Four-week omega-3 polyunsaturated fatty acids supplementation for liver left lateral section volume reduction in individuals with morbid obesity undergoing bariatric surgery: A double blind, multicenter, randomized placebo-controlled trial. International journal of surgery (London, England). 2022. 101:106614 | Wrong or no Outcome |
| Ibekwe, S. O., Mondal, S., Faloye, A. O.. Pulmonary prehabilitation and smoking cessation. CURRENT OPINION IN ANESTHESIOLOGY. 2023. 36:96-102 | Not a Randomized Trial |
| Jing, G. W., Xie, Q., Tong, J., Liu, L. Z., Jiang, X., Si, L.. Early Intervention of Perioperative Delirium in Older Patients (>60&thinsp;years) with Hip Fracture: a Randomized Controlled Study. . 2022. 14:885 | Wrong or Unclear Duration |
| Jing, Guang-Wu, Xie, Qin, Tong, Jie, Liu, Lian-Zhong, Jiang, Xue, Si, Liang. Early Intervention of Perioperative Delirium in Older Patients (>60 years) with Hip Fracture: A Randomized Controlled Study. Orthopaedic surgery. 2022. 14:885-891 | Wrong or Unclear Duration |
| Kasai, Shunsuke, Yamauchi, Shinichi, Tazawa, Miyako, Takaoka, Ayumi, Hanaoka, Marie, Iwata, Noriko, Masuda, Taiki, Tokunaga, Masanori, Kinugasa, Yusuke. Advantages of preoperative counseling with video for robotic rectal cancer surgery compared with conventional counseling: A randomized controlled trial. Asian journal of endoscopic surgery. 2022. | Wrong or Unclear Duration |
| Kasvis, P., Vigano, A., Bui, T., Kim, H., Hachem, Y., Kilgour, R. D.. IMPACT OF CANCER SYMPTOM BURDEN ON QUALITY OF LIFE IN PATIENTS UNDERGOING PREHABILITATION FOR LIVER RESECTION: RESULTS FROM A 12-WEEK RCT. #journal#. 2022. 30:S171 | Wrong or no Outcome |
| Kaushik, D., Shah, P. K., Mukherjee, N., Ji, N., Dursun, F., Kumar, A. P., Thompson, I. M., Mansour, A. M., Jha, R., Yang, X., Wang, H., Darby, N., Ricardo Rivero, J., Svatek, R. S., Liss, M. A.. Effects of yoga in men with prostate cancer on quality of life and immune response: a pilot randomized controlled trial. . 2022. 25:531 | Duplicate |
| Khalil, Lafi, Jildeh, Toufic, Abbas, Muhammad, Buckley, Patrick, Moutzouros, Vasilios, Okoroha, Kelechi, Tramer, Joseph. Paper 21: Blood Flow Restriction Therapy Improves Early Patient Reported Outcomes Following ACL Reconstruction...American Orthopaedic Society for Sports Medicine (AOSSM) Specialty Day, March 22-26, 2022, Chicago, Illinois. Orthopaedic Journal of Sports Medicine. . 10:1-2 | Duplicate |
| Kharod, Utpala, Panchal, Nirali N., Varma, Jagdish, Sutaria, Krupa. Effect of pre-operative communication using anaesthesia information sheet on pre-operative anxiety of patients undergoing elective surgery-A randomised controlled study. Indian journal of anaesthesia. 2022. 66:559-572 | Wrong or Unclear Duration |
| Kim, Sunghye, Hsu, Fang-Chi, Groban, Leanne, Williamson, Jeff, Messier, Stephen. A pilot study of aquatic prehabilitation in adults with knee osteoarthritis undergoing total knee arthroplasty - short term outcome. BMC Musculoskeletal Disorders. 2021. 22:1-11 | Duplicate |
| Klek, S., Kret, K., Choruz, R., Pisarska-Adamczyk, M., Salowka, J., Cegielny, T., Welanyk, J., Wilczek, M., Pedziwiatr, M.. Immunomodulating versus high-protein oral preoperative supplement in surgical patients &ndash; A two-center, prospective, randomized clinical trial. . 2022. 101: | Duplicate |
| Knoerl, R., Giobbie-Hurder, A., Sannes, T. S., Chagpar, A. B., Dillon, D., Dominici, L. S., Frank, E. S., Golshan, M., McTiernan, A., Rhei, E., Tolaney, S. M., Winer, E. P., Yung, R. L., Irwin, M. L., Ligibel, J. A.. Exploring the impact of exercise and mind-body prehabilitation interventions on physical and psychological outcomes in women undergoing breast cancer surgery. . 2022. 30:2027 | Duplicate |
| Koc, M. A., Akyol, C., Gokmen, D., Aydin, D., Erkek, A. B., Kuzu, M. A.. Effect of Prehabilitation on Stoma Self-Care, Anxiety, Depression, and Quality of Life in Patients with Stomas: A Randomized Controlled Trial. Diseases of the Colon and Rectum. 2023. 66:138-147 | Not Prehabilitation |
| Koc, M. A., Akyol, C., Gokmen, D., Aydin, D., Erkek, B. A., Kuzu, M. A.. Effect of Prehabilitation on Stoma Self-Care, Anxiety, Depression and Quality of Life in Stoma Patients: a Randomized Controlled Trial. . 2022. | Not Prehabilitation |
| Konen, J., Callas, P., Lepuschenko, K., Jones, E., Moore, J., Evans, K., Liu, S., Cataldo, P.. IMPACT OF LOW DOSE CARBOHYDRATE AND CITRULLINE LOADING ON ARGININE/ADMA RATIO IN ELECTIVE COLORECTAL SURGERY. Diseases of the Colon and Rectum. 2022. 65:197-198 | Wrong or Unclear Duration |
| Kwok, K., Bharti, N., Gamey, D., Vergis, A., Hardy, K., Bouchard, D.. The impact of a preoperative exercise program on patients awaiting bariatric surgery. CMAJ. Canadian Medical Association Journal. 2017. 60:S111 | Wrong or no Outcome |
| Lange, Undine Gabriele, Moulla, Yusef, Schutz, Tatjana, Bluher, Matthias, Peter, Veronika, Shang, Edward, Dietrich, Arne. Effectiveness and Tolerability of a Two-Week Hypocaloric Protein-Rich Diet Prior to Obesity Surgery with Two Different Diet Interventions: a Prospective Randomized Trial. Obesity surgery. 2022. 32:2903-2913 | Wrong or no Outcome |
| Li, Dapeng, Tian, Mengran, Zhang, Yan, Yu, Yang, Cheng, Wenyuan, Li, Yigong, Wang, Junyi, Wei, Songfeng, Wang, Xin, Yang, Xiaoyong, Zhao, Jingzhu, Yun, Xinwei, Zhang, Wei, Song, Jiayin, Zhang, Huan, Zheng, Xiangqian, Gao, Ming. Preoperative supplementation of calcitriol and calcium relieves symptom and extent of hypocalcemia in patients undergoing total thyroidectomy and bilateral central compartment neck dissection: A prospective, randomized, open-label, parallel-controlled cli. Frontiers in oncology. 2022. 12:967451 | Wrong or Unclear Duration |
| Lopes, A., Yamada, A. M. T. D., Cardenas, T. C., Carvalho, J. N., Oliveira, E. A., Silva, M. E. R. D., Andrade, J. F. M., de Souza Neto, E., Barros, L. A. D. R., Costa, R. L. R.. PROPER-PRehabilitatiOn Plus Enhanced Recovery after surgery versus enhanced recovery after surgery in gynecologic oncology: a randomized clinical trial. International journal of gynecological cancer : official journal of the International Gynecological Cancer Society. 2022. 32:195-197 | Protocol |
| Lou, J., Cai, Y., Fang, F.. Application Effect of NRS2002 Scale in Perioperative Nutritional Intervention of Patients Undergoing Radical Cystectomy. . 2022. 24: | Wrong or Unclear Duration |
| Lu, Han-Bing, Ma, Rui-Chen, Yin, Ying-Ying, Song, Chun-Yu, Yang, Ting-Ting, Xie, Jiao. Clinical Indicators of Effects of Yoga Breathing Exercises on Patients With Lung Cancer After Surgical Resection: A Randomized Controlled Trial. Cancer nursing. 2023. #volume#:#pages# | Wrong or Unclear Duration |
| Lu, Z., Song, J.. Comment on: "effects of Community-based Exercise Prehabilitation for Patients Scheduled for Colorectal Surgery with High Risk for Postoperative Complications: Results of a Randomized Clinical Trial". Annals of Surgery. 2022. 276:E1120 | Not a Randomized Trial |
| Luo, Chao, Xie, Kai, Zhang, Chi, Cong, Zhuang-Zhuang, Gu, Wen-Feng, Xu, Yang, Qiang, Yong, Li, Xiao-Kun, Zheng, Chao, Hu, Li-Wen, Shen, Yi. Efficacy of immunonutritional supplement after neoadjuvant chemotherapy in patients with esophageal cancer. Journal of Cardiothoracic Surgery. 2022. 17:1-7 | Not a Randomized Trial |
| Ma, Xiaojie, Zhang, Zaozhang, Peng, Mengsi, Yao, Bonuan, Jiang, Hongtao, Ji, Xuanfu, You, Yong. Face-to-Face Mentoring, Remotely Supervised Home Exercise Prehabilitation to Improve Physical Function in Patients Awaiting Kidney Transplantation: A Randomized Clinical Trial. Frontiers in psychology. 2022. 13:831445 | Intervention Not Preoperative |
| Maheshwari, Deepali, Hall, Cynthia D., Jia, Xibei, Tangada, Abhilasha, Wu, Emily K., Leung, Katherine, Flynn, Michael K.. The Effect of Preoperative Fiber on Postoperative Bowel Function After Pelvic Reconstructive Surgery: A Randomized Controlled Trial. Urogynecology (Hagerstown, Md.). 2022. 28:554-560 | Not Prehabilitation |
| Mansell, G., den Hollander, M., Lotzke, H., Smeets, Rjem, Lundberg, M.. A Person-Centred Prehabilitation Program based on Cognitive-Behavioural Physical Therapy for patients scheduled for Lumbar Fusion surgery: a mediation analysis to assess fear of movement (kinesiophobia), self-efficacy, and catastrophizing as mediators of. . 2022. | Not a Randomized Trial |
| Mansell, G., den Hollander, M., Lotzke, H., Smeets, Rjem, Lundberg, M.. A Person-Centred Prehabilitation Program based on Cognitive Behavioural Physical Therapy for patients scheduled for Lumbar Fusion surgery: a mediation analysis to assess fear of movement (kinesiophobia), self-efficacy and catastrophizing as mediators of h. . 2022. 26:1790 | Not a Randomized Trial |
| Manyande, Anne, Berg, Simon, Gettins, Doreen, Stanford, S. Clare, Mazhero, Sarah, Marks, David F., Salmon, Peter, Manyande, A., Berg, S., Gettins, D., Stanford, S. C., Mazhero, S., Marks, D. F., Salmon, P.. Preoperative rehearsal of active coping imagery influences subjective and hormonal responses to abdominal surgery. Psychosomatic Medicine. 1995. 57:177-182 | Wrong or Unclear Duration |
| Marchand, Andree-Anne, Houle, Marieve, O'Shaughnessy, Julie, Chatillon, Claude-Edouard, Descarreaux, Martin. Physical Predictors of Favorable Postoperative Outcomes in Patients Undergoing Laminectomy or Laminotomy for Central Lumbar Spinal Stenosis: Secondary Analysis of a Randomized Controlled Trial. Frontiers in neurology. 2022. 13:848665 | Wrong or no Outcome |
| McLean, L., Charette, M., Varette, K., Brooks, K., Harvey, M. A., Robert, M., Baker, K., Day, A., Della Zazzera, V., Sauerbrei, E., Brison, R.. Pelvic floor muscle training as an adjunct to a midurethral sling: a single-blind randomised controlled trial. . 2022. 33:809 | Duplicate |
| Meyer, Vanessa, Goble, Nicole, Hunter, Michelle M.. Enhancing OT's Role in Preoperative Total Joint Education: A Cognitive-Behavioral Approach to Care...American Occupational Therapy Association, INSPIRE Conference, March 31-April 3, 2022, San Antonio, Texas. American Journal of Occupational Therapy. . 76:1-1 | Wrong or Unclear Duration |
| Milios, Joanne E., Ackland, Timothy R., Green, Daniel J.. Pelvic floor muscle training in radical prostatectomy: a randomized controlled trial of the impacts on pelvic floor muscle function and urinary incontinence. BMC Urology. 2019. 19:1-10 | Duplicate |
| Mils, Kristel, Miro, Monica, Farran, Leandre, Videla, Sebastian, Alba, Esther, Estremiana, Fernando, Bettonica, Carla, Aranda, Humberto. A pilot randomized controlled trial on the utility of gastric conditioning in the prevention of esophagogastric anastomotic leak after Ivor Lewis esophagectomy. The APIL_2013 Trial. International journal of surgery (London, England). 2022. 106:106921 | Not Prehabilitation |
| Mils, K., Miro, M., Farran, L., Videla, S., Alba, E., Estremiana, F., Bettonica, C., Aranda, H.. A pilot randomized controlled trial on the utility of gastric conditioning in the prevention of esophagogastric anastomotic leak after Ivor Lewis esophagectomy. . 2022. | Intervention Not Preoperative |
| Miralpeix, E., Sole-Sedeno, J. M., Rodriguez-Cosmen, C., Taus, A., Muns, M. D., Fabrego, B., Mancebo, G.. Impact of prehabilitation during neoadjuvant chemotherapy and interval cytoreductive surgery on ovarian cancer patients: a pilot study. WORLD JOURNAL OF SURGICAL ONCOLOGY. 2022. 20: | Not a Randomized Trial |
| Moya, Pedro, Soriano-Irigaray, Leticia, Ramirez, Jose Manuel, Garcea, Alessandro, Blasco, Olga, Blanco, Francisco Javier, Brugiotti, Carlo, Miranda, Elena, Arroyo, Antonio. Perioperative Standard Oral Nutrition Supplements Versus Immunonutrition in Patients Undergoing Colorectal Resection in an Enhanced Recovery (ERAS) Protocol A Multicenter Randomized Clinical Trial (SONVI Study). Medicine. 2016. 95:1-11 | Duplicate |
| Norouzi, Mona, Nadjarzadeh, Azadeh, Maleki, Majid, Khayyatzadeh, Sayyed Saeid, Hosseini, Saeid, Yaseri, Mehdi, Fattahi, Hamed. Evaluation of the recovery after heart surgery following preoperative supplementation with a combination of beta-hydroxy-beta-methylbutyrate, L-arginine, and L-glutamine: a double-blind randomized placebo-controlled clinical trial. Trials. 2022. 23:649 | Not Prehabilitation |
| Norouzi, M., Nadjarzadeh, A., Maleki, M., Khayyatzadeh, S. S., Hosseini, S., Yaseri, M., Fattahi, H.. The effects of preoperative supplementation with a combination of beta-hydroxy-beta-methylbutyrate, arginine, and glutamine on inflammatory and hematological markers of patients with heart surgery: a randomized controlled trial. . 2022. 22:51 | Wrong or no Outcome |
| Nyhus, M. Ø, Mathew, S., Salvesen, Ø, Salvesen, K. Å, Stafne, S., Volløyhaug, I.. Effect of preoperative pelvic floor muscle training on pelvic floor muscle contraction and symptomatic and anatomical pelvic organ prolapse after surgery: randomized controlled trial. Ultrasound in Obstetrics & Gynecology. 2020. 56:28-36 | Duplicate |
| Onerup, A., Andersson, J., Angenete, E., Bock, D., Borjesson, M., Ehrencrona, C., Fagevik Olsen, M., Larsson, P. A., De La Croix, H., Wedin, A., Haglind, E.. Effect of Short-term Homebased Pre- and Postoperative Exercise on Recovery after Colorectal Cancer Surgery (PHYSSURG-C): A Randomized Clinical Trial. Annals of Surgery. 2022. 275:448-455 | Duplicate |
| Osterholt, T., Gloistein, C., Todorova, P., Becker, I., Arenskrieger, K., Melka, R., Koehler, F. C., Faust, M., Wahlers, T., Benzing, T., Muller, R. U., Grundmann, F., Burst, V.. Preoperative Short-Term Restriction of Sulfur-Containing Amino Acid Intake for Prevention of Acute Kidney Injury After Cardiac Surgery: A Randomized, Controlled, Double-Blind, Translational Trial. Journal of the American Heart Association. 2022. 11:e025229 | Not Prehabilitation |
| Pacheco-Brousseau, Lissa, Dobransky, Johanna, Jane, Alanna, Beaule, Paul E., Poitras, Stephane. Feasibility of a preoperative strengthening exercise program on postoperative function in patients undergoing hip or knee arthroplasty: a pilot randomized controlled trial. Pilot and feasibility studies. 2022. 8:162 | Wrong or no Outcome |
| Pardo, P. L., Bouzon, C. A., Torregrossa, R. P., Herrera, E. R.. Prehabilitation in older patient with severe aortic stenosis pending intervention. European Geriatric Medicine. 2022. 13:S105 | Not a Randomized Trial |
| Patel, Y., Churchill, I., Sullivan, K., Beauchamp, M., Wald, J., Mbuagbaw, L., Agzarian, J., Shargall, Y., Finley, C., Fahim, C., Hanna, W.. Move For Surgery, a novel preconditioning program to optimize health before thoracic surgery: a randomized controlled trial. CMAJ. Canadian Medical Association Journal. 2021. 64:S107 | Duplicate |
| Pattamatta, Madhuri, Evers, Silvia M. A. A., Smeets, Boudewijn J. J., Peters, Emmeline G., Luyer, Misha D. P., Hiligsmann, Mickael. An economic evaluation of perioperative enteral nutrition in patients undergoing colorectal surgery (SANICS II study). Journal of Medical Economics. 2018. :1-14 | Wrong or Unclear Duration |
| Pattamatta, Madhuri, Evers, Silvia M. A. A., Smeets, Boudewijn J. J., Peters, Emmeline G., Luyer, Misha D. P., Hiligsmann, Mickael. An economic evaluation of perioperative enteral nutrition in patients undergoing colorectal surgery (SANICS II study). Journal of Medical Economics. 2019. 22:238-244 | Wrong or Unclear Duration |
| Pico-Sirvent, I., Manresa-Rocamora, A., Aracil-Marco, A., Moya-Ramon, M.. A Combination of Aerobic Exercise at Fatmax and Low Resistance Training Increases Fat Oxidation and Maintains Muscle Mass, in Women Waiting for Bariatric Surgery. . 2022. | Not a Randomized Trial |
| Powell, A., Allen, S., Casey, P., Hunt, J., Prabhu, P., Jack, S., Rockall, T., Preston, S., Sultan, J.. Cost utility analysis of a prehabilitation programme in patients undergoing treatment for oesophageal cancer. British Journal of Surgery. 2022. 109:ix61 | Wrong or no Outcome |
| Reme, S. E., Munk, A., Holter, M. T. S., Falk, R. S., Jacobsen, H. B.. Pre- and post-operative psychological interventions to prevent pain and fatigue after breast cancer surgery (PREVENT): a randomized controlled trial. medRxiv. 2022. | No Results |
| Risso, Anna Maria, van der Linden, Marietta L., Bailey, Andrea, Gallacher, Peter, Gleeson, Nigel. Exploratory insights into novel prehabilitative neuromuscular exercise-conditioning in total knee arthroplasty. BMC musculoskeletal disorders. 2022. 23:547 | Wrong or no Outcome |
| Salman, Mohamed AbdAlla, Qassem, Mohamed Gamal, Aboul-Enein, Mohamed Saad, A.Ameen, Mahmoud, Abdallah, Ahmed, Omar, Hitham, Hussein, Ahmed Mahmoud, Tourky, Mohamed Sabry, Monazea, Khaled, M.Hassan, Ahmed, Salman, Ahmed, Moustafa, Ahmed, Shaaban, Hossam El-Din, Soliman, Ahmed, Sarhan, Mohamed D.. Effect of preoperative diet regimen on liver size before laparoscopic sleeve gastrectomy in morbidly obese patients. Surgical Endoscopy. 2022. 36:2981-2986 | Not a Randomized Trial |
| Sasi, M. V., Shreyamsa, M., Garg, S., Enny, L., Singh, K. R., Rana, C., Ramakant, P., Mishra, A.. Role of Preoperative Calcium and Vitamin D Supplementation in Preventing Post-total Thyroidectomy Hypocalcemia. World Journal of Endocrine Surgery. 2022. 14:7-14 | Wrong Population |
| Sassani, J. C., Grosse, P. J., Kunkle, L., Baranski, L., Ackenbom, M. F.. Patient Preparedness for Pelvic Organ Prolapse Surgery: A Randomized Equivalence Trial of Preoperative Counseling. Obstetrical and Gynecological Survey. 2022. 77:149-150 | Wrong or Unclear Duration |
| Schiavo, L., Pierro, R., Asteria, C., Calabrese, P., Di Biasio, A., Coluzzi, I., Severino, L., Giovanelli, A., Pilone, V., Silecchia, G.. Low-Calorie Ketogenic Diet with Continuous Positive Airway Pressure to Alleviate Severe Obstructive Sleep Apnea Syndrome in Patients with Obesity Scheduled for Bariatric/Metabolic Surgery: a Pilot, Prospective, Randomized Multicenter Comparative Study. . 2022. 32:634 | Wrong or no Outcome |
| Schulz, G. B., Locke, J. A., Campbell, K. L., Bland, K. A., Van Patten, C. L., Black, P. C., Goldenberg, S. L., Flannigan, R.. Taking Advantage of the Teachable Moment at Initial Diagnosis of Prostate Cancer - Results of a Pilot Randomized Controlled Trial of Supervised Exercise Training. Cancer Nursing. 2022. 45:E680-E688 | Duplicate |
| Schulz, S. V. W., Schumann, U., Otto, S., Kirsten, J., Treff, G., Janni, W., Huober, J., Leinert, E., Steinacker, J. M., Bizjak, D. A.. Two-year follow-up after a six-week high-intensity training intervention study with breast cancer patients: physiological, psychological and immunological differences. . 2022. 44:4813 | Intervention Not Preoperative |
| Shafiei, S. H., Siavashi, B., Ghasemi, M., Golbakhsh, M. R., Baghdadi, S.. Single High-Dose Systemic Methylprednisolone Administered Preoperatively Improves Pain Control and Sleep Quality After Total Hip Arthroplasty: A Double-Blind, Randomized Controlled Trial. Arthroplasty Today. 2022. 16:78-82 | Wrong or Unclear Duration |
| Shahood, H., Pakai, A., Rudolf, K., Bory, E., Szilagyi, N., Sandor, A., Zsofia, V.. The effect of preoperative chest physiotherapy on oxygenation and lung function in cardiac surgery patients: a randomized controlled study. . 2022. 42:8 | Duplicate |
| Shen, Yu, Zhao, Xin, Zhao, Haijian, Chen, Ning, Wang, Jian, Zhuang, Haiwen, Zhang, Xiaoyu. Clinical Application of Enteral Nutrition Combined with Microbial Preparation for Intestinal Preparation in Elderly Patients with Colorectal Cancer. Medical science monitor : international medical journal of experimental and clinical research. 2022. 28:e935366 | Wrong or Unclear Duration |
| Singh, Vikram, Agumbe Pai, Sreekar, Hosmath, Vijaykumar. Clinical outcome of patients undergoing preoperative chest physiotherapy in elective upper abdominal surgeries. Journal of perioperative practice. 2022. :17504589211045225 | Wrong or Unclear Duration |
| Skoffer, Birgit, Dalgas, Ulrik, Maribo, Thomas, Søballe, Kjeld, Mechlenburg, Inger. No Exacerbation of Knee Joint Pain and Effusion Following Preoperative Progressive Resistance Training in Patients Scheduled for Total Knee Arthroplasty: Secondary Analyses From a Randomized Controlled Trial. PM & R: Journal of Injury, Function & Rehabilitation. 2018. 10:687-692 | Wrong or no Outcome |
| St-Pierre, J., Drummond, K., Minella, E., Scheede-Bergdahl, C., Ferri, L., Carli, F.. Feasibility of multimdodal prehabilitation to enhance preoperative functional capacity of esophageal cancer patients during concurrent neoadjuvant chemotherapies - a pilot interventional study. European Journal of Surgical Oncology. 2022. 48:e38 | Wrong or Unclear Duration |
| Sweity, Essa M., Alkaissi, Aidah A., Othman, Wafiq, Salahat, Ahmad. Preoperative incentive spirometry for preventing postoperative pulmonary complications in patients undergoing coronary artery bypass graft surgery: a prospective, randomized controlled trial. Journal of Cardiothoracic Surgery. 2021. 16:1-11 | Wrong or Unclear Duration |
| Sykes, Kevin J., Gibbs, Heather, Farrokhian, Nathan, Arthur, Anna, Flynn, John, Shnayder, Yelizaveta, Kakarala, Kiran, Nallani, Rohit, Smith, Joshua B., Penn, Joseph, Fassas, Scott, Cummings, Emily, Arambula, Zack, Karadaghy, Omar, Bur, Andres M.. Pilot randomized, controlled, preoperative intervention for nutrition trial in head and neck cancer. Head & neck. 2023. 45:156-166 | Duplicate |
| Sykes, K. J., Gibbs, H., Farrokhian, N., Arthur, A., Flynn, J., Shnayder, Y., Kakarala, K., Nallani, R., Smith, J. B., Penn, J., Fassas, S., Cummings, E., Arambula, Z., Karadaghy, O., Bur, A. M.. POINT: Pilot randomized, controlled, preoperative intervention for nutrition trial in head and neck cancer. HEAD AND NECK-JOURNAL FOR THE SCIENCES AND SPECIALTIES OF THE HEAD AND NECK. 2023. 45:156-166 | Duplicate |
| Terradas-Monllor, Marc, Ochandorena-Acha, Mirari, Beltran-Alacreu, Hector, Garcia Oltra, Ester, Collado Saenz, Fernando, Hernandez Hermoso, Jose. A feasibility study of home-based preoperative multimodal physiotherapy for patients scheduled for a total knee arthroplasty who catastrophize about their pain. Physiotherapy theory and practice. 2022. #volume#:1-20 | Wrong or no Outcome |
| Tramer, J. S., Khalil, L. S., Jildeh, T. R., Abbas, M. J., McGee, A., Lau, M. J., Moutzouros, V., Okoroha, K. R.. Blood Flow Restriction Therapy For Two Weeks Prior to Anterior Cruciate Ligament Reconstruction Did Not Impact Quadriceps Strength Compared to Standard Therapy. . 2022. | Not Prehabilitation |
| Tramer, Joseph S., Khalil, Lafi S., Jildeh, Toufic R., Abbas, Muhammad J., McGee, Anna, Lau, Michael J., Moutzouros, Vasilios, Okoroha, Kelechi R.. Blood Flow Restriction Therapy for 2 Weeks Prior to Anterior Cruciate Ligament Reconstruction Did Not Impact Quadriceps Strength Compared to Standard Therapy. Arthroscopy : the journal of arthroscopic & related surgery : official publication of the Arthroscopy Association of North America and the International Arthroscopy Association. 2023. 39:373-381 | Not Prehabilitation |
| Trubnikova, Olga A., Tarasova, Irina V., Moskin, Evgeniy G., Kupriyanova, Darya S., Argunova, Yuliya A., Pomeshkina, Svetlana A., Gruzdeva, Olga V., Barbarash, Olga L.. Beneficial Effects of a Short Course of Physical Prehabilitation on Neurophysiological Functioning and Neurovascular Biomarkers in Patients Undergoing Coronary Artery Bypass Grafting. Frontiers in Aging Neuroscience. 2021. 13:1-11 | Wrong or Unclear Duration |
| Tucker, Katherine, Sullivan, Stephanie, Deal, Allison M., Allman, Kathryn, Cuaboy, Luz, McCabe, Sean D., Gehrig, Paola A.. A prospective randomized trial of standard versus multimedia-supplemented counseling in patients undergoing endometrial cancer staging surgery. Gynecologic oncology. 2022. 166:397-402 | Wrong or Unclear Duration |
| van Rooijen, Stefanus, Carli, Francesco, Dalton, Susanne, Thomas, Gwendolyn, Bojesen, Rasmus, Le Guen, Morgan, Barizien, Nicolas, Awasthi, Rashami, Minnella, Enrico, Beijer, Sandra, Martínez-Palli, Graciela, van Lieshout, Rianne, Gögenur, Ismayil, Feo, Carlo, Johansen, Christoffer, Scheede-Bergdahl, Celena, Roumen, Rudi, Schep, Goof, Slooter, Gerrit. Multimodal prehabilitation in colorectal cancer patients to improve functional capacity and reduce postoperative complications: the first international randomized controlled trial for multimodal prehabilitation. BMC Cancer. 2019. 19:1-11 | Protocol |
| Van Vulpen, J. K., Hiensch, A. E., Van Hillegersberg, R., Ruurda, J. P., Backx, F. J. G., Nieuwenhuijzen, G. A. P., Kouwenhoven, E. A., Groenendijk, R. P. R., Van Der Peet, D. L., Hazebroek, E. J., Rosman, C., Wijnhoven, B. P. L., Van Berge Henegouwen, M. I., Van Laarhoven, H. W. M., Siersema, P. D., May, A. M.. Supervised exercise after oesophageal cancer surgery: the PERFECT multicentre randomized clinical trial. British Journal of Surgery. 2021. 108:786-796 | Intervention Not Preoperative |
| Vasankari, Sini, Hartikainen, Juha, Vasankari, Ville, Anttila, Vesa, Tokola, Kari, Vaha-Ypya, Henri, Husu, Pauliina, Sievanen, Harri, Vasankari, Tommi, Halonen, Jari. Objectively measured preoperative physical activity and sedentary behaviour among Finnish patients scheduled for elective cardiac procedures: baseline results from randomized controlled trial. BMC sports science, medicine & rehabilitation. 2022. 14:130 | Not a Randomized Trial |
| Vecchiato, M., Deana, C., Ziccarelli, A., Raimondi, P., Martino, A., Pontoni, M., Patruno, V., Bassi, F., Petri, R.. EFFECTS OF PREHABILITATION AND POST-OPERATIVE HIGH-FLOW NASAL CANNULA AFTER OPEN ESOPHAGECTOMY ON PULMONARY COMPLICATIONS: A FEASIBILITY STUDY. Diseases of the Esophagus. 2022. 35:38 | Not a Randomized Trial |
| Verdaasdonk, E., Maas, H., Van Munster, B., Rikkert, M. O., De Wilt, H., Melis, R.. Improved postoperative outcomes after prehabilitation for colorectal cancer surgery in older patients: An emulated target trial. European Geriatric Medicine. 2022. 13:S7 | Not a Randomized Trial |
| Vincent, S., Paskey, T., Critchlow, E., Mann, E., Chapman, T., Abboudi, J., Jones, C., Kirkpatrick, W., Namdari, S., Hammoud, S., Ilyas, A. M.. Prospective Randomized Study Examining Preoperative Opioid Counseling on Postoperative Opioid Consumption after Upper Extremity Surgery. Hand (New York, N.Y.). 2022. 17:200-205 | Wrong or Unclear Duration |
| Wall, C., Glyn, T., Bissett, I., Rowbotham, D., Haines, M., Gearry, R., Eglinton, T.. Preoperative nutrition optimisation in nourished Crohn's disease patients: A feasibility randomised controlled trial. Colorectal Disease. 2022. 24:144 | Wrong Comparator Group |
| Wang, Shuang, Yu, Hai-Long, Zheng, Liang, Ma, Jun-Xiong, Wang, Hong, Xiang, Liang-Bi, Chen, Yu. Randomized controlled trial of overall functional exercise process in perioperative of percutaneous transforaminal endoscopic discectomy. Medicine. 2022. 101:e32544 | Wrong or Unclear Duration |
| Wang, Zinian, Tu, Chengjian, Pratt, Rachel, Khoury, Thaer, Qu, Jun, Fahey, Jed W., McCann, Susan E., Zhang, Yuesheng, Wu, Yue, Hutson, Alan D., Ambrosone, Christine B., Edge, Stephen B., Cappuccino, Helen H., Takabe, Kazuaki, Young, Jessica S., Tang, Li. A Presurgical-Window Intervention Trial of Isothiocyanate-Rich Broccoli Sprout Extract in Patients with Breast Cancer. Molecular nutrition & food research. 2022. 66:e2101094 | Wrong or no Outcome |
| Wong, Ting Xuan, Wong, Wei Xiang, Chen, Seong Ting, Ong, Shu Hwa, Shyam, Sangeetha, Ahmed, Nurzarina, Hamdan, Khairul Hazim, Awang, Raflis Ruzairee, Ibrahim, Mohd Razali, Palayan, Kandasami, Chee, Winnie Siew Swee. Effects of Perioperative Oral Nutrition Supplementation in Malaysian Patients Undergoing Elective Surgery for Breast and Colorectal Cancers-A Randomised Controlled Trial. Nutrients. 2022. 14:#pages# | Wrong or Unclear Duration |
| Wong, T. X., Wong, W. X., Chen, S. T., Ong, S. H., Shyam, S., Ahmed, N., Hamdan, K. H., Awang, R. R., Ibrahim, M. R., Palayan, K., Chee, W. S. S.. Effects of Perioperative Oral Nutrition Supplementation in Malaysian Patients Undergoing Elective Surgery for Breast and Colorectal Cancers&mdash;A Randomised Controlled Trial. . 2022. 14: | Duplicate |
| Woodfield, J. C., Clifford, K., Wilson, G. A., Munro, F., Baldi, J.. Short&dash;term high&dash;intensity interval training improves fitness before surgery: a randomized clinical trial. . 2022. 32:856 | Duplicate |
| Woodfield, John C., Clifford, Kari, Wilson, Genevieve A., Munro, Fran, Baldi, James C. Short‐term high‐intensity interval training improves fitness before surgery: A randomized clinical trial. Scandinavian Journal of Medicine & Science in Sports. 2022. 32:856-865 | Duplicate |
| Xiayun, Wang, Yifan, G. U., Hong, Chen, Lin, Zhang, Xiujuan, D. A. I., Aijie, Tang, Xinmiao, H. U., Qian, W. U.. 术前三联预康复对老年结直肠肿瘤病人 康复的影响. Chinese Nursing Research. 2022. 36:3233-3238 | Wrong or Unclear Duration |
| Yagi, Taro, Sawada, Kenjiro, Miyamoto, Mayuko, Kinose, Yasuto, Nakagawa, Satoshi, Takiuchi, Tsuyoshi, Kodama, Michiko, Kobayashi, Eiji, Hashimoto, Kae, Mabuchi, Seiji, Tomimatsu, Takuji, Yoshino, Kiyoshi, Kimura, Tadashi. Safety and efficacy of Ninjin'yoeito along with iron supplementation therapy for preoperative anemia, fatigue, and anxiety in patients with gynecological disease: an open-label, single-center, randomized phase-II trial. BMC women's health. 2022. 22:229 | Not Prehabilitation |
| Yang, F. N., Li, L. J., Mi, Y. Z., Zou, L. M., Chu, X. F., Sun, A. Y., Sun, H. B., Liu, X. B., Xu, X. X.. Effectiveness of the Tailored, Early Comprehensive Rehabilitation Program (t-ECRP) based on ERAS in improving the physical function recovery for patients following minimally invasive esophagectomy: a prospective randomized controlled trial. SUPPORTIVE CARE IN CANCER. 2022. 30:5027-5036 | Intervention Not Preoperative |
| Yang, Yang, Zhang, Haibin, Li, Yuling, Liu, Zhifen, Liu, Sha, Li, Xinrong, Fan, Gaiping, Xu, Yong, Wang, Bin-quan. The effectiveness of computer-assisted cognitive behavioral therapy (cCBT) for psychological outcomes in patients with laryngectomy: Randomized controlled trial. Journal of Affective Disorders. 2022. 300:59-65 | Wrong or Unclear Duration |
| Zheng, Y., Huang, Z., Dai, L., Liu, Y., Chen, Y., Zhang, W., Lin, R.. The Effect of Preoperative Rehabilitation Training on the Early Recovery of Joint Function after Artificial Total Knee Arthroplasty and Its Effect Evaluation. . 2022. 2022: | Wrong or Unclear Duration |
| Zhou Xiaomei, Zong Li, Chen Xiaoyan, Xiao Ting, Miao Yuanshu. Application of delirium prevention nursing program in elderly patients with esophageal cancer surgery. Nursing of Integrated Traditional Chinese & Western Medicine. 2018. 4:57-61 | Not a Randomized Trial |
| Cai Daoling. Application of health education based on Green's model in perioperative functional exercise of lung cancer patients. Chinese Journal of Integrative Nursing. 2022. 8:50-55 | Wrong or Unclear Duration |
| Roxburgh, Brendon H, Campbell, Holly A, Cotter, James D, Reymann, Ulla, Williams, Michael J A, Gwynne-Jones, David, Thomas, Kate N. Upper-limb high-intensity interval training or passive heat therapy to optimise cardiorespiratory fitness prior to total hip or knee arthroplasty: a randomised controlled trial.. #journal#. 2023. #volume#:#pages# | Wrong or no Outcome |
| Duarte-Rojo, Andres, Bloomer, Pamela M, Grubbs, Rachel K, Stine, Jonathan G, Ladner, Daniela, Hughes, Christopher B, Dunn, Michael A, Jakicic, John M. Use of a mobile-assisted telehealth regimen to increase exercise (MATRIX) in transplant candidates - A home-based prehabilitation pilot and feasibility trial.. #journal#. 2023. #volume#:#pages# | Intervention Not Preoperative |
| Du, Di, Li, Hong, Xu, Yongqing, Zheng, Tian'E, Xu, Xiaoyan, Wang, Jun, Tao, Rou, Wang, Junmei, Yang, Yuan, Xu, Jiang, Li, Jiexiu, Jiang, Min. Study on the Effect of Pain Programmed Care Based on the Concept of Prehabilitation on the Recovery of Joint Function and WHOQOL-BREF Score in Elderly Patients after Total Hip Arthroplasty.. #journal#. 2023. #volume#:#pages# | Wrong or no Outcome |
| Tonnesen, Hanne, Raffing, Rie, Lauridsen, Susanne Vahr, Lauritzen, Jes Bruun, Elholm, Anne Marie Halmo, Jensen, Helle Saederup, Espinosa, Peter, Jansson, Karl Ake, Berman, Anne H, Fernandez-Valencia, Jenaro, Munoz-Mahamud, Ernesto, Santina, Manuel, Combalia, Andres. Two novel prehabilitation apps to help patients stop smoking and risky drinking prior to hip and knee arthroplasty.. #journal#. 2023. #volume#:#pages# | Single Risk Factor Management |
| Serper, Marina, Jones, Lauren S, Clement, Thomas, Reddy, Rajender K, Reese, Peter P. A randomized, controlled, prehabilitation intervention to maximize early recovery (PRIMER) in liver transplantation.. #journal#. 2023. #volume#:#pages# | Wrong or no Outcome |
| Li, Yu-Qian, Qu, Xiao-Peng, Peng, Li-Wei, An, Jie-Yuan, Liu, Xin-Wei, Zhang, Yue, Wang, Chao, Jiang, Xue, Gao, Li, Li, Gang, Wang, Da-Li, Zhao, De-Chang, Qu, Yan, Liu, Bei. Targeted nutritional intervention with enhanced recovery after surgery for carotid endarterectomy: A prospective clinical trial.. #journal#. 2023. 10:951174 | Not a Randomized Trial |
| Croce, Laura, Pallavicini, Cristina, Busca, Noemi, Cali, Benedetto, Bellastella, Giuseppe, Coperchini, Francesca, Magri, Flavia, Chiovato, Luca, Cena, Hellas, Rotondi, Mario. Pre-surgery dietician counseling can prevent post-thyroidectomy body weight gain: results of an intervention trial.. #journal#. 2023. 81:246 | Wrong or Unclear Duration |
| Bulut, Gamze, Karabulut, Neziha. The Effects of Breathing Exercises on Patients Having Laparoscopic Cholecystectomy Surgery.. #journal#. 2023. 32:805 | Intervention Not Preoperative |
| Chen, Sai, Li, Xin, Wu, Yunshan, Li, Yana, Cao, Peili, Yin, Yuchun, Chen, Zhenguang. Preoperative respiratory muscle training combined with aerobic exercise improves respiratory vital capacity and daily life activity following surgical treatment for myasthenia gravis.. #journal#. 2023. 18:160 | Wrong or Unclear Duration |
| Castaldo, Giuseppe, Schiavo, Luigi, Pagano, Imma, Molettieri, Paola, Conte, Aurelio, Sarno, Gerardo, Pilone, Vincenzo, Rastrelli, Luca. Clinical Impact of Enteral Protein Nutritional Therapy on Patients with Obesity Scheduled for Bariatric Surgery: A Focus on Safety, Efficacy, and Pathophysiological Changes.. #journal#. 2023. 15:#pages# | Wrong or no Outcome |
| Sumin, Alexey N, Oleinik, Pavel A, Bezdenezhnykh, Andrey V, Bezdenezhnykh, Natalia A. Prehabilitation in Cardiovascular Surgery: The Effect of Neuromuscular Electrical Stimulation (Randomized Clinical Trial).. #journal#. 2023. 20:#pages# | Wrong or Unclear Duration |
| Cabral S.M., Oliveira C., Castro C., Goncalves D.M., Irving S., Santos L.. Nutritional domain during prehabilitation in the age of ERAS: opportunities during neoadjuvancy for locally advanced gastric cancer. #journal#. 2023. 57:805 | Not a Randomized Trial |
| Tay T., Haque A.. A four-year follow-up of the Abdominal Aortic Aneurysm Get Fit randomised control trial. #journal#. 2023. 131:e94 | Wrong Population |
| Dave P., Mir J., Das A., Lorentz N., Galetta M., Lebovic J., Tretiakov P., Schoenfeld A., Onafowokan O., Passias P.. 145. Optimizing mental health conditions prior to adult spinal deformity surgery: does preoperative optimization improve surgical outcomes?. #journal#. 2023. 23:S74 | Wrong or Unclear Duration |
| Guven B., Ibrahimoglu O., Calikoglu I., Yuksel S., Demir A., Bektas H.. THE EFFECT OF PROGRESSIVE RELAXATION EXERCISES ON BARIATRIC SURGERY PERIOD (PREBARI). PRELIMINARY RESULTS OF A PROSPECTIVE RANDOMIZED CONTROLLED CLINICAL TRIAL. #journal#. 2023. 33:322 | Wrong or no Outcome |
| Estalella L., Espina B., Guasch A., Moline A., Renzulli M., Pavel M.-C., Llacer-Millan E., Pueyo E., Ramirez E., Memba R., Jorba R.. Multimodal Prehabilitation during Neoadjuvant Chemotherapy in Patients with Colorectal Liver Metastases: Preliminary Results. #journal#. 2023. 25:S389 | Wrong or no Outcome |
| Serper M., Jones L., Clement T., Dwinnells K., Zaleski D., Reese P.. Prehabilitation intervention to maximize early recovery (PRIMER) in liver transplantation: a randomized, controlled trial. #journal#. 2023. 78:S48 | Wrong or no Outcome |
| Bean D., Collier J., Rice D., Kluger M., McNair P., Young S., Walker M., Tuck N.. Cognitive behavioural pain management prior to total knee joint replacement: a feasibility trial. #journal#. 2023. 136:105 | Wrong or no Outcome |
| Andring L., Corrigan K., Bailard N., Rooney M., Domingo M., Varkey J., Foster-Mills T., Fellman B., Kazantsev T., Lin L., Jhingran A., Colbert L., Eifel P., Klopp A., Joyner M.. The Role for an Enhanced Recovery Pathway for Gynecologic Cancer Patients Receiving Brachytherapy: A Prospective Clinical Trial. #journal#. 2023. 46:S84 | Wrong Population |
| Hanley A.W., Gililland J., Erickson J., Pelt C., Peters C., Rojas J., Garland E.L.. Brief preoperative mind-body therapies for total joint arthroplasty patients: A randomized controlled trial. #journal#. 2021. 162:1749 | Wrong or Unclear Duration |
| Pymer S., Gurung R., Wallace T., Chetter I., Smith G., Ibeggazene S., Carradice D., Huang C., Rhavindhran B.. A Preoperative Supervised Exercise Program Provides a 5-year Mortality Benefit for Patients Undergoing Abdominal Aortic Aneurysm Repair. #journal#. 2023. 77:e252 | Wrong or Unclear Duration |
| Meyer V.M., Beydoun H.A., Gyenai L., Goble N.M., Hunter M.M., McGill R.J.. The Effect of Preoperative Behavioral Intervention on Pain, Anxiety, Opioid Use, and Function in Patients Undergoing Total Knee Arthroplasty: A Randomized Controlled Study. #journal#. 2023. 188:e1010 | Wrong or Unclear Duration |
| Granger C., Denehy L., Edbrooke L., Abo S., Whish-Wilson G., Parry S.. Patients before lung cancer surgery have poor exercise capacity. #journal#. 2023. 28:180 | Not a Randomized Trial |
| Khorrami M.-H., Mohseni A., Gholipour F., Alizadeh F., Zargham M., Izadpanahi M.-H., Sichani M., Khorrami F.. Single session pre-operative pelvic floor muscle training with biofeedback on urinary incontinence and quality of life after radical prostatectomy: A randomized controlled trial. #journal#. 2023. 34:23 | Wrong or Unclear Duration |
| Harvey J., Tolerico P.H., Bell T., Mason L., McKinney H., Shaeffer C., Kashyap R.. PREHABILITATION FOR PATIENTS UNDERGOING TRANSCATHETER AORTIC VALVE REPLACEMENT: A PILOT RANDOMIZED CLINICAL TRIAL. #journal#. 2023. 81:868 | Not Prehabilitation |
| Lu H.-B., Ma R.-C., Yin Y.-Y., Song C.-Y., Yang T.-T., Xie J.. Clinical Indicators of Effects of Yoga Breathing Exercises on Patients With Lung Cancer After Surgical Resection: A Randomized Controlled Trial. #journal#. 2023. #volume#:#pages# | Wrong or Unclear Duration |
| Construction and application of perioperative progressive exercise rehabilitation management model in patients with abdominal malignant tumor. #journal#. 2023. #volume#:#pages# | Protocol |
| Effects of movement with breathing exercise regimen on cardiopulmonary function in patients undergoing coronary artery bypass graft surgery. #journal#. 2023. #volume#:#pages# | Not a Randomized Trial |
| Effects of preoperative exercise on vascular caliber for arteriovenous fistula: a randomized controlled trial. #journal#. 2023. #volume#:#pages# | Protocol |
| Effect of Music Prehabilitation on Preoperative Anxiety Before Surgery. #journal#. 2023. #volume#:#pages# | Wrong or no Outcome |
| The effect of preoperative inspiratory muscle training on postoperative complications of thoracic tumors: a randomized controlled study. #journal#. 2023. #volume#:#pages# | Protocol |
| Based on best evidence perioperative respiratory muscle exercise program in patients undergoing cardiac surgery. #journal#. 2023. #volume#:#pages# | Protocol |
| Pre-Habilitation With Mindfulness and Exercise for Patients Undergoing Radical Cystectomy (PRIMER Trial). #journal#. 2023. #volume#:#pages# | Wrong or no Outcome |
| Terradas-Monllor M, Ochandorena-Acha M, Beltran-Alacreu H, Garcia Oltra E, Collado Saenz F, Hernandez Hermoso J. A feasibility study of home-based preoperative multimodal physiotherapy for patients scheduled for a total knee arthroplasty who catastrophize about their pain. #journal#. 2023. 39:1606 | Wrong or no Outcome |
| Multimodal Prehabilitation for Lung Cancer Surgery. #journal#. 2023. #volume#:#pages# | Protocol |
| Evaluation and study on the effect of preoperative and postoperative quantitative muscle strength training on pain, function, gait and balance after total hip arthroplasty. #journal#. 2023. #volume#:#pages# | Protocol |
| Pragmatic randomized controlled trial of prehabilitation strategy in the elderly patients in the department of hepatobiliary pancreatic surgery. #journal#. 2023. #volume#:#pages# | Protocol |
| Singh V, Agumbe Pai S, Hosmath V. Clinical outcome of patients undergoing preoperative chest physiotherapy in elective upper abdominal surgeries. #journal#. 2023. 33:182 | Wrong or Unclear Duration |
| Prehabilitation Effect on Function and Patient Satisfaction Following Total Knee Arthroplasty. #journal#. 2023. #volume#:#pages# | Protocol |
| Effect of preoperative inspiratory muscle training combined with rehabilitation education on postoperative complications in high-risk patients with lung cancer. #journal#. 2023. #volume#:#pages# | Protocol |
| Effect of pre-rehabilitation training on postoperative daily living ability of lung cancer patients. #journal#. 2023. #volume#:#pages# | Protocol |
| Prehabilitation "Karl-Heinz" with a focus on cardiac and cognitive functions prior to interventions on the heart: an analysis of the state of health. #journal#. 2023. #volume#:#pages# | Protocol |
| Online Prehabilitation for Patients Awaiting Liver Transplantation. #journal#. 2023. #volume#:#pages# | Protocol |
| Fit 4 Surgery 2: using an app to get fit for lung cancer surgery. #journal#. 2023. #volume#:#pages# | Protocol |
| Djurhuus SS, Simonsen C, Toft BG, Thomsen SN, Wielsoe S, Roder MA, Hasselager T, Ostergren PB, Jakobsen H, Pedersen BK, Hojman P, Brasso K, Christensen JF. Exercise training to increase tumour natural killer-cell infiltration in men with localised prostate cancer: a randomised controlled trial. #journal#. 2023. 131:116 | Wrong Population |
| The PREHAAAB Trial: multimodal Prehabilitation for Patients Awaiting Open Abdominal Aortic Aneurysm Repair. #journal#. 2023. #volume#:#pages# | Wrong or no Outcome |
| Furon Y, Dang Van S, Blanchard S, Saulnier P, Baufreton C. Effects of high-intensity inspiratory muscle training on systemic inflammatory response in cardiac surgery - A randomized clinical trial. #journal#. 2023. #volume#:1 | Duplicate |
| Evaluating Pre-Treatment Vestibular Physical Therapy Rehab for Patients With Vestibular Schwannomas. #journal#. 2023. #volume#:#pages# | No Results |
| Multimodal Prehabilitation for Resectable Gastric Cancer. #journal#. 2023. #volume#:#pages# | Wrong or no Outcome |
| BaRiatric Surgery AnD FRUctose Handeling In Obese subjecTs. #journal#. 2023. #volume#:#pages# | Protocol |
| Effect of pre-operative walking exercise on post-operative bowel function in patient with major gynecological surgery, randomized clinical trial. #journal#. 2023. #volume#:#pages# | Wrong or Unclear Duration |
| Tramer JS, Khalil LS, Jildeh TR, Abbas MJ, McGee A, Lau MJ, Moutzouros V, Okoroha KR. Blood Flow Restriction Therapy for 2 Weeks Prior to Anterior Cruciate Ligament Reconstruction Did Not Impact Quadriceps Strength Compared to Standard Therapy. #journal#. 2023. 39:373 | Wrong Population |
| Sykes KJ, Gibbs H, Farrokhian N, Arthur A, Flynn J, Shnayder Y, Kakarala K, Nallani R, Smith JB, Penn J, Fassas S, Cummings E, Arambula Z, Karadaghy O, Bur AM. Pilot randomized, controlled, preoperative intervention for nutrition trial in head and neck cancer. #journal#. 2023. 45:156 | Duplicate |
| Koc MA, Akyol C, Gokmen D, Aydin D, Erkek AB, Kuzu MA. Effect of Prehabilitation on Stoma Self-Care, Anxiety, Depression, and Quality of Life in Patients with Stomas: a Randomized Controlled Trial. #journal#. 2023. 66:138 | Single Risk Factor Management |
| Rojewski, Alana M, Fucito, Lisa M, Baker, Nathaniel L, Krishnan-Sarin, Suchitra, Carpenter, Matthew J, Bernstein, Steven L, Toll, Benjamin A. A preoperative contingency management intervention for smoking abstinence in cancer patients: A preliminary randomized controlled trial.. #journal#. 2021. 23:1064 | Single Risk Factor Management |
| Kasvis, Popi, Vigano, Antonio, Bui, Tram, Carli, Franco, Kilgour, Robert D.. Impact of Dietary Counseling on Health-Related Quality of Life in Patients with Cancer Awaiting Hepato-Pancreato-Biliary Surgery.. #journal#. 2023. 75:1151 | Not a Randomized Trial |
| Strijker, Dieuwke, Meijerink, Wilhelmus J. H. J., van Heusden-Schotalbers, Linda A. G., van den Berg, Manon G. A., van Asseldonk, Monique J. M. D., Drager, Luuk D., de Wilt, Johannes H. W., van Laarhoven, Kees J. H. M., van den Heuvel, Baukje. Multimodal Prehabilitation in Patients Undergoing Complex Colorectal Surgery, Liver Resection, and Hyperthermic Intraperitoneal Chemotherapy (HIPEC): A Pilot Study on Feasibility and Potential Efficacy.. #journal#. 2023. 15:1870 | Not a Randomized Trial |
| Kim, Sunghye, Hsu, Fang-Chi, Groban, Leanne, Williamson, Jeff, Messier, Stephen. A pilot study of aquatic prehabilitation in adults with knee osteoarthritis undergoing total knee arthroplasty - short term outcome.. #journal#. 2021. 22:1 | Duplicate |
| Khushnood, Kiran, Sultan, Nasir, Awan, Malik Muhammad Ali, Altaf, Shafaq, Mehmood, Riafat, Qureshi, Sidra. Effects of Pre-operative Physical Therapy on Functional Capacity, Kinesiophobia, and Post-operative ICU Stay in Coronary Artery Bypass Grafting Candidates.. #journal#. 2023. 21:81 | Wrong or Unclear Duration |
| Ferreira, Vanessa, Lawson, Claire, Carli, Francesco, Scheede-Bergdahl, Celena, Chevalier, Stéphanie. Feasibility of a novel mixed-nutrient supplement in a multimodal prehabilitation intervention for lung cancer patients awaiting surgery: A randomized controlled pilot trial.. #journal#. 2021. 93:N.PAG | Wrong or no Outcome |
| McIsaac, Daniel I., Hladkowicz, Emily, Bryson, Gregory L., Forster, Alan J., Gagne, Sylvain, Huang, Allen, Lalu, Manoj, Lavallée, Luke T., Moloo, Husein, Nantel, Julie, Power, Barbara, Scheede-Bergdahl, Celena, van Walraven, Carl, McCartney, Colin J.L., Taljaard, Monica. Home-based prehabilitation with exercise to improve postoperative recovery for older adults with frailty having cancer surgery: the PREHAB randomised clinical trial.. #journal#. 2022. 129:41 | Duplicate |
| Hassan, Anwar, Boyle, Shelley, Lai, William, Barve, Kirti, Scanlon, Katherine, Shakeshaft, Anthony J., Cox, Michael R.. Prehabilitation and education in major abdominal and thoracic surgery reduces length of stay and ventilation days.. #journal#. 2022. 43:149 | Not a Randomized Trial |
| Waller, Ellen, Sutton, Paul, Rahman, Seema, Allen, Jonathan, Saxton, John, Aziz, Omer. Prehabilitation with wearables versus standard of care before major abdominal cancer surgery: a randomised controlled pilot study (trial registration: NCT04047524).. #journal#. 2022. 36:1008 | Duplicate |
| Labuschagne, Rozelle, Roos, Ronel. Pre-operative physiotherapy for elderly patients undergoing abdominal surgery.. #journal#. 2022. 78:1 | Duplicate |
| Wang, Shuang, Yu, Hai-Long, Zheng, Liang, Ma, Jun-Xiong, Wang, Hong, Xiang, Liang-Bi, Chen, Yu. Randomized controlled trial of overall functional exercise process in perioperative of percutaneous transforaminal endoscopic discectomy.. #journal#. 2022. 101:e32544 | Wrong or Unclear Duration |
| Rengel, Kimberly F., Mehdiratta, Nitin, Vanston, Susan W., Archer, Kristin R., Jackson, James C., Thompson, Jennifer L., Pandharipande, Pratik P., Hughes, Christopher G.. A randomised pilot trial of combined cognitive and physical exercise prehabilitation to improve outcomes in surgical patients.. #journal#. 2021. 126:e55 | Duplicate |
| Pattamatta, Madhuri, Evers, Silvia M. A. A., Smeets, Boudewijn J. J., Peters, Emmeline G., Luyer, Misha D. P., Hiligsmann, Mickael. An economic evaluation of perioperative enteral nutrition in patients undergoing colorectal surgery (SANICS II study).. #journal#. 2019. 22:238 | Wrong or Unclear Duration |
| IJmker‐Hemink, Vera E., Wanten, Geert J. A., Nes, Lindsey C. F., den Berg, Manon G. A.. Effect of a Preoperative Home‐Delivered, Protein‐Rich Meal Service to Improve Protein Intake in Surgical Patients: A Randomized Controlled Trial.. #journal#. 2021. 45:479 | Wrong or no Outcome |
| Huber, Erika O, Roos, Ewa M, Meichtry, André, de Bie, Rob A, Bischoff-Ferrari, Heike A. Effect of preoperative neuromuscular training (NEMEX-TJR) on functional outcome after total knee replacement: an assessor-blinded randomized controlled trial.. #journal#. 2015. 16:101 | Duplicate |
| Salzmann, Stefan, Euteneuer, Frank, Laferton, Johannes A. C., Auer, Charlotte J., Shedden-Mora, Meike C., Schedlowski, Manfred, Moosdorf, Rainer, Rief, Winfried. Effects of preoperative psychological interventions on catecholamineand cortisol levels after surgery in coronary artery bypass graft patients: the randomized controlled PSY-HEART trial.. #journal#. 2017. 79:806 | Wrong or Unclear Duration |
| Swaminathan, Nagalakshmi, Kundra, Pankaj, Ravi, Ramya, Kate, Vikram. ERAS protocol with respiratory prehabilitation versus conventional perioperative protocol in elective gastrectomy- a randomized controlled trial.. #journal#. 2020. 81:149 | Wrong or Unclear Duration |
| Humeidan, Michelle L., Otey, Andrew, Zuleta-Alarcon, Alix, Mavarez-Martinez, Ana, Stoicea, Nicoleta, Bergese, Sergio. Perioperative Cognitive Protection—Cognitive Exercise and Cognitive Reserve (The Neurobics Trial): A Single-blind Randomized Trial.. #journal#. 2015. 37:2641 | No Results |
| Moya, Pedro, Soriano-Irigaray, Leticia, Ramirez, Jose Manuel, Garcea, Alessandro, Blasco, Olga, Blanco, Francisco Javier, Brugiotti, Carlo, Miranda, Elena, Arroyo, Antonio. Perioperative Standard Oral Nutrition Supplements Versus Immunonutrition in Patients Undergoing Colorectal Resection in an Enhanced Recovery (ERAS) Protocol A Multicenter Randomized Clinical Trial (SONVI Study).. #journal#. 2016. 95:1 | Duplicate |
| Au, Darren, Matthew, Andrew G., Lopez, Paty, Hilton, William J., Awasthi, Rashami, Bousquet-Dion, Guillaume, Ladha, Karim, Carli, Franco, Santa Mina, Daniel. Prehabilitation and acute postoperative physical activity in patients undergoing radical prostatectomy: a secondary analysis from an RCT.. #journal#. 2019. 5:N.PAG | Duplicate |
| Angus Lee, Chun Hin, Murnane, Andrew, Heriot, Alexander G., Ismail, Hilmy, Riedel, Bernhard. Randomized Pilot Study of Enhanced Structured Preoperative Exercise Program for Patients with Rectal Cancer Requiring Neoadjuvant Therapy Before Major Resection.. #journal#. #year#. 227:S73 | Wrong or Unclear Duration |
| Jongbloed, Franny, de Bruin, Ron W. F., Klaassen, René A., Beekhof, Piet, van Steeg, Harry, Dor, Frank J. M. F., van der Harst, Erwin, Dollé, Martijn E. T., IJzermans, Jan N. M.. Short-Term Preoperative Calorie and Protein Restriction Is Feasible in Healthy Kidney Donors and Morbidly Obese Patients Scheduled for Surgery.. #journal#. 2016. 8:1 | Wrong or Unclear Duration |
| Fulop, A., Lakatos, L., Susztak, N., Szijarto, A., Banky, B.. The effect of trimodal prehabilitation on the physical and psychological health of patients undergoing colorectal surgery: a randomised clinical trial.. #journal#. 2021. 76:82 | Duplicate |
| 周晓梅, 宗莉, 陈晓燕, 肖婷, 缪愿戍. 谵妄预防护理方案在老年食管癌手术患者中的应用.. #journal#. 2018. 4:57 | Not a Randomized Trial |
| Brahmbhatt, P, Hong, NL, Srikandarajah, A, Lemon-Wong, S, Alavi, N, Gibson, L, Berger-Richardson, D, Rapier, T, Mascarenhas, J, Isenberg-Grzeda, E, Selvadurai, S, Mina, DS, Wright, F. A Randomized Control Pilot of Prehabilitation During Neoadjuvant Chemotherapy for Women with Breast Cancer: A Mixed Methods Study. #journal#. 2023. 30:S280 | Wrong or no Outcome |
| Molenaar, CJL, Minnella, EM, Coca-Martinez, M. Effect of Multimodal Prehabilitation on Reducing Postoperative Complications and Enhancing Functional Capacity Following Colorectal Cancer Surgery: the PREHAB Randomized Clinical Trial (vol 158, pg 572, 2023). #journal#. 2023. #volume#:#pages# | Duplicate |
| Wooldrik, S.. Prehabilitation and rehabilitation in breast cancer surgery patients - a pilot study. European Journal of Cancer. 2024. 200:113648 | No Results |
| Woods, Carri. FASTING VERSUS A HEARTHEALTHY DIET BEFORE CARDIAC CATHETERIZATION: A RANDOMIZED CONTROLLED TRIAL. American Journal of Critical Care. 2024. 33:29-33 | Wrong or Unclear Duration |
| Wilschut, Esther D., de Winter, Eduard P., Bos, Ernst J., Putter, Hein, Rotmans, Joris I., van der Bogt, Koen E. A.. Supervised Pre-Operative Forearm Exercise to Increase Blood Vessel Diameter in Haemodialysis Patients: The PINCH Trial. European journal of vascular and endovascular surgery : the official journal of the European Society for Vascular Surgery. 2024. #volume#:#pages# | No Results |
| Usupharach, W., Yasud, M., Songsaengrit, B., Donsom, M., Yasud, P., Chantawong, S., Ekphaphan, N., Kittipanya-Ngam, P.. EFFECT OF PRE-OPERATIVE MULTIMEDIA PHYSICAL THERAPY PROGRAM IN OPEN HEART SURGERY: A RANDOMIZED CONTROLLED STUDY. Cardiopulmonary Physical Therapy Journal. 2024. 35:a29-a30 | Wrong or no Outcome |
| Takahashi, Mamoru, Okada, Harutaro, Kogaki, Mako, Shirokihara, Rio, Kawate, Yuka, Tokumasu, Hironobu, Aoyama, Akihiro. Short-term effects of preoperative nutritional intervention in lung surgery for malignant tumors: a single-center prospective study. General thoracic and cardiovascular surgery. 2024. #volume#:#pages# | Not a Randomized Trial |
| Skladany, Lubomir, Liska, David, Gurin, Daniel, Molcan, Pavol, Bednar, Roman, Vnencakova, Janka, Koller, Tomas. The influence of prehabilitation in patients with liver cirrhosis before liver transplantation: a randomized clinical trial. European journal of physical and rehabilitation medicine. 2024. 60:122-129 | Intervention Not Preoperative |
| Simpson, A. Hamish R. W., Clement, Nicholas D., Simpson, Sharon A., Pandit, Hemandt, Smillie, Susie, Leeds, Anthony R., Conaghan, Philip G., Kingsbury, Sarah R., Hamilton, David, Craig, Peter, Ray, David, Keerie, Catriona, Kinsella, Elaine, Bell-Higgs, Anna, McGarty, Arlene, Beadle, Christine, Howie, Colin R., Norrie, John. A preoperative package of care for osteoarthritis, consisting of weight loss, orthotics, rehabilitation, and topical and oral analgesia (OPPORTUNITY): a two-centre, open-label, randomised controlled feasibility trial. The Lancet. Rheumatology. 2024. 6:e237-e246 | Wrong Population |
| Sethi, S., Ravindhran, B., Long, J., Gurung, R., Huang, C., Smith, G. E., Carradice, D., Wallace, T., Ibeggazene, S., Chetter, I. C., Pymer, S.. A preoperative supervised exercise program potentially improves long-term survival after elective abdominal aortic aneurysm repair. Journal of vascular surgery. 2024. 79:15 | Duplicate |
| Serper, M., Jones, L. S., Clement, T., Reddy, R. K., Reese, P. P.. A randomized, controlled, prehabilitation intervention to maximize early recovery (PRIMER) in liver transplantation. Liver Transplantation. 2024. 30:10-19 | Wrong or no Outcome |
| Sahar, W., Waseem, M., Riaz, M., Nazeer, N., Ahmad, M., Haider, Z.. Effects of prehabilitation resistance training in mild to moderate clinically frail patients awaiting coronary artery bypass graft surgery. Journal of Investigative Medicine. 2024. 72:151-158 | Duplicate |
| Roxburgh, B. H., Campbell, H. A., Cotter, J. D., Reymann, U., Williams, M. J. A., Gwynne-Jones, D., Thomas, K. N.. Upper-Limb High-Intensity Interval Training or Passive Heat Therapy to Optimize Cardiorespiratory Fitness Prior to Total Hip or Knee Arthroplasty: A Randomized Controlled Trial. Arthritis Care and Research. 2024. 76:393-402 | Wrong or no Outcome |
| Rayner, C., Allen, S. A., Seymour, T. S., Preston, S. P., Frampton, A. E., Bartlett, D., Annels, N., Abbassi-Ghadi, N.. Increasing tumour infiltrating lymphocytes through exercise alone in oesophageal adenocarcinoma. European journal of surgical oncology. 2024. 50:#pages# | Wrong or no Outcome |
| Moreira-Gonçalves, D., Castro, C., Jorge, N., Cabral, S., Vaz, J. A., Fonseca, A. S., Romano, O., Neves, P., Martins, P., Santos, L. L.. Effects of structured vs non-structured home-based prehabilitation during neoadjuvant chemotherapy with FLOT in surgical gastric cancer patients: preliminary results of a randomized trail (PROTECT). Annals of Surgical Oncology. 2024. 31:S288-S289 | Wrong or Unclear Duration |
| Ma, Jiaojie, Li, Zhuo, Chen, Yang, Zhang, Yachao, Wang, Qian, Yan, Guangxuan, Dong, Weijie, Li, Shanshan. Perioperative nutrition management in patients with spinal tuberculosis taking ERAS measures. Asia Pacific journal of clinical nutrition. 2024. 33:39-46 | Wrong or Unclear Duration |
| Kasvis, P., Vigano, A., Bui, T., Carli, F., Kilgour, R.. Cancer symptom burden negatively affects health-related quality of life in patients undergoing prehabilitation prior to liver resection: results from a 12-week randomized controlled trial. Applied physiology, nutrition, and metabolism = Physiologie appliquee, nutrition et metabolisme. 2024. 49:64-76 | Duplicate |
| Hirst, Nicholas, McBride, Kate, Steffens, Daniel. Psychological Interventions in Prehabilitation Randomized Controlled Trials for Patients Undergoing Cancer Surgery: Sufficient or Suboptimal?. Annals of surgical oncology. 2024. 31:2183-2186 | Not a Randomized Trial |
| Guclu, Duygu, Isiksacan, Nilgun, Seyit, Hakan, Gedikbasi, Asuman, Karabulut, Mehmet, Erdil, Irem, Tasci, Tamay Seda, Yaman, Mustafa. EFFECT OF DIET BEFORE BARIATRIC SURGERY ON GHRELIN LEVEL THROUGH DNA METHYLATION. Annals of nutrition & metabolism. 2024. #volume#:#pages# | Wrong or no Outcome |
| Goncalves, D., Castro, C., Jorge, N., Mota, C. D., Araujo, L., Antunes, J., Pinhal, C., Ferreira, C., Fernandes, F., Cabral, S., Oliveira, C., Almeida, S., Fonseca, A. S., Vaz, J. A., Romano, O., Neves, P., Ferreira, P., Santos, F., Martins, P., Santos, L. L.. Effects of structured vs non-structured home-based prehabilitation during neoadjuvant chemotherapy with FLOT in surgical gastric cancer patients: preliminary results of a randomized trial (PROTECT). European Journal of Surgical Oncology. 2024. 50:107358 | Wrong or Unclear Duration |
| Furon, Y., Dang Van, S., Blanchard, S., Saulnier, P., Baufreton, C.. Effects of high-intensity inspiratory muscle training on systemic inflammatory response in cardiac surgery - A randomized clinical trial. Physiotherapy theory and practice. 2024. 40:778-788 | Duplicate |
| Brahmbhatt, Priya, Look Hong, Nicole J., Sriskandarajah, Apishanthi, Alavi, Nasrin, Selvadurai, Sarah, Berger-Richardson, David, Lemon-Wong, Sharon, Mascarenhas, Joanna, Gibson, Leslie, Rapier, Tracey, Isenberg-Grzeda, Elie, Bernstein, Lori J., Santa Mina, Daniel, Wright, Frances C.. A Feasibility Randomized Controlled Trial of Prehabilitation During Neoadjuvant Chemotherapy for Women with Breast Cancer: A Mixed Methods Study. Annals of surgical oncology. 2024. 31:2261-2271 | Duplicate |
| Atoui, S., Carli, F., Bernard, P., Lee, L., Stein, B., Charlebois, P., Liberman, A. S.. Does a multimodal prehabilitation program improve sleep quality and duration in patients undergoing colorectal resection for cancer? Pilot randomized control trial. Journal of behavioral medicine. 2024. 47:43-61 | Duplicate |
| Abdelaziz, Heba Ahmed, Dean, Yomna E., Elshafie, Ahmed Mohamed Ahmed. Effect of three modalities on emergence agitation among post-traumatic stress disorder patients undergoing laparoscopy: a randomized controlled study. BMC psychiatry. 2024. 24:78 | Wrong or Unclear Duration |
| Wilschut, E. D., De Winter, E. P., Bos, E. J., Van Zoest, D., De Vries, M. R., Putter, H., Siddiqi, L., Hamming, J. F., Rotmans, J. I., Van Der Bogt, K. E. A.. Supervised Preoperative Forearm Exercise To Increase Blood Vessel Diameter In Patients Requiring An Arteriovenous Access For Hemodialysis. Journal of Vascular Access. 2023. 24:NP42-NP43 | Wrong or no Outcome |
| Triguero-Canovas, Daniel, Lopez-Rodriguez-Arias, Francisco, Gomez-Martinez, Manuel, Sanchez-Guillen, Luis, Peris-Castello, Franc, Alcaide-Quiros, Maria Jose, Morillas-Blasco, Pedro, Arroyo, Antonio, Ramirez, Jose Manuel. Home-based prehabilitation improves physical conditions measured by ergospirometry and 6MWT in colorectal cancer patients: a randomized controlled pilot study. Supportive care in cancer : official journal of the Multinational Association of Supportive Care in Cancer. 2023. 31:673 | Duplicate |
| Thoft Jensen, Bente, Bjerggaard Jensen, Jorgen. One-Year Follow-Up after Multimodal Prehabilitation Interventions in Radical Cystectomy. Cancers. 2023. 15:#pages# | Duplicate |
| Tay, T., Haque, A.. A four-year follow-up of the Abdominal Aortic Aneurysm Get Fit randomised control trial. British journal of anaesthesia. 2023. 131:e94 | Duplicate |
| Sun, J., Shan, Y., Wu, L., Li, N., Xu, F., Kong, X., Zhang, B.. Preoperative high-intensity strength training combined with balance training can improve early outcomes after total knee arthroplasty. Journal of orthopaedic surgery and research. 2023. 18:1 | Duplicate |
| Srivastava, M., Sharma, S. K., Kumar, A., Kibria, T.. Impact of Pre-Operative Nutritional Support on Surgical Outcome in Gastrointestinal Surgery. Research Journal of Medical Sciences. 2023. 17:82-87 | Not Prehabilitation |
| Singh, F., Newton, R. U., Taaffe, D. R., Lopez, P., Thavaseelan, J., Brown, M., Ooi, E., Nosaka, K., Hayne, D., Galvao, D. A.. Prehabilitative versus rehabilitative exercise in prostate cancer patients undergoing prostatectomy. Journal of cancer research and clinical oncology. 2023. 149:16563 | Duplicate |
| Serper, M., Jones, L., Clement, T., Dwinnells, K., Zaleski, D., Reese, P.. Prehabilitation intervention to maximize early recovery (PRIMER) in liver transplantation: a randomized, controlled trial. Journal of hepatology. 2023. 78:S48 | Duplicate |
| Saleh, Hesham, Williamson, Tyler K., Passias, Peter G.. Perioperative Nutritional Supplementation Decreases Wound Healing Complications Following Elective Lumbar Spine Surgery: A Randomized Controlled Trial. Spine (03622436). 2023. 48:376-383 | Duplicate |
| Rodriguez, J. G. Z., Cos, H., Srivastava, R., Bewley, A., Raper, L., Li, D. W., Dai, R. X., Williams, G. A., Fields, R. C., Hawkins, W. G., Lu, C. Y., Sanford, D. E., Hammill, C. W.. Preoperative levels of physical activity can be increased in pancreatectomy patients via a remotely monitored, telephone-based intervention: A randomized trial. Surgery in Practice and Science. 2023. 15:6 | Duplicate |
| Peng, L., Song, Y., Lv, B., Jing, C.. The effect of implementation of pain neuroscience education and rehabilitation exercise on post-operative pain and recovery after laparoscopic colorectal surgery: a prospective randomized controlled trial. Journal of anesthesia. 2023. 37:775 | Wrong or Unclear Duration |
| Okoroha, Kelechi R., Tramer, Joseph S., Khalil, Lafi S., Jildeh, Toufic R., Abbas, Muhammad J., Buckley, Patrick J., Lindell, Craig, Moutzouros, Vasilios. Effects of a Perioperative Blood Flow Restriction Therapy Program on Early Quadriceps Strength and Patient-Reported Outcomes After Anterior Cruciate Ligament Reconstruction. Orthopaedic journal of sports medicine. 2023. 11:23259671231209694 | Wrong Population |
| Ninomiya, K., Takahira, N., Ikeda, T., Suzuki, K., Sato, R., Mihara, M.. Effects of perioperative exercise therapy combined with nutritional supplementation on functional recovery after fast-track total hip arthroplasty. Journal of orthopaedic science. 2023. 28:1291 | Duplicate |
| Ngo-Huang, A. T., Parker, N. H., Xiao, L., Schadler, K. L., Petzel, M. Q. B., Prakash, L. R., Kim, M. P., Tzeng, C. D., Lee, J. E., Ikoma, N., Wolff, R. A., Javle, M. M., Koay, E. J., Pant, S. D., Folloder, J. P., Wang, X., Cotto, A. M., Ju, Y. R., Garg, N., Wang, H., Bruera, E. D., Basen-Engquist, K. M., Katz, M. H. G.. Effects of a Pragmatic Home-based Exercise Program Concurrent with Neoadjuvant Therapy on Physical Function of Patients with Pancreatic Cancer: the Pancfit Randomized Clinical Trial. Annals of surgery. 2023. 278:22 | Duplicate |
| Molenaar, C. J. L., Minnella, E. M., Coca-Martinez, M.. Effect of multimodal prehabilitation on reducing postoperative complications and enhancing functional capacity following colorectal cancer surgery: the PREHAB randomized clinical trial (vol 158, pg 572, 2023). Jama Surgery. 2023. 158:675-675 | Duplicate |
| Meyer, Vanessa M., Beydoun, Hind A., Gyenai, Leonora, Goble, Nicole M., Hunter, Michelle M., McGill, Robert J.. The effect of preoperative behavioral intervention on pain, anxiety, opioid use, and function in patients undergoing total knee arthroplasty: A randomized controlled study. Military Medicine. 2023. 188:e1010-e1017 | Wrong or Unclear Duration |
| Martinez-Huenchullan, S., Enriquez-Schmidt, J., Mautner-Molina, C., Kalazich-Rosales, M., Munoz, M., Fuentes-Leal, F., Monrroy-Uarac, M., San Martin-Correa, M., Carcamo-Ibaceta, C., Ehrenfeld, P.. Moderate and high-intensity aerobic exercise exert differing metabolic benefits in candidates to undergo bariatric surgery: a randomised controlled trial. Diabetologia. 2023. 66:S250-S251 | Wrong or no Outcome |
| Machado, P., Pimenta, S., Garcia, A. L., Nogueira, T., Silva, S., Oliveiros, B., Martins, R. A., Cruz, J.. Home-Based Preoperative Exercise Training for Lung Cancer Patients Undergoing Surgery: a Feasibility Trial. Journal of clinical medicine. 2023. 12:#pages# | Not a Randomized Trial |
| Littlewood, Chris, Moffatt, Maria, Beckhelling, Jacqueline, Davis, Daniel, Burden, Adrian, Pitt, Lisa, Lalande, Stacey, Maddocks, Catrin, Stephens, Gareth, Tunnicliffe, Helen, Pawson, Jessica, Lloyd, James, Manca, Andrea, Wade, Julia, Foster, Nadine E.. Physiotherapist-led exercise versus usual care (waiting-list) control for patients awaiting rotator cuff repair surgery: A pilot randomised controlled trial (POWER). Musculoskeletal science & practice. 2023. 68:102874 | Intervention Not Preoperative |
| Kuppuswamy, P., Chaganti, Y. S. R., Hari, P., Mothilal. Aerobic exercise and preoperative respiratory muscle training improve respiratory vital capacity and everyday activity after surgical treatment for myasthenia gravis. Journal of cardiovascular disease research. 2023. 14:1599 | Wrong or Unclear Duration |
| Kotfis, K., Wojciechowska, A., Zimny, M., Jamiol-Milc, D., Szylinska, A., Kwiatkowski, S., Kaim, K., Dolegowska, B., Stachowska, E., Zukowski, M., Pankowiak, M., Torbe, A., Wischmeyer, P.. Preoperative Oral Carbohydrate (CHO) Supplementation Is Beneficial for Clinical and Biochemical Outcomes in Patients Undergoing Elective Cesarean Delivery under Spinal Anaesthesia-A Randomized Controlled Trial. Journal of Clinical Medicine. 2023. 12:4978 | Wrong or Unclear Duration |
| Koc, M. A., Akyol, C., Gokmen, D., Aydin, D., Erkek, A. B., Kuzu, M. A.. Effect of Prehabilitation on Stoma Self-Care, Anxiety, Depression, and Quality of Life in Patients With Stomas: a Randomized Controlled Trial. Diseases of the colon and rectum. 2023. 66:138 | Not Prehabilitation |
| Knoerl, Robert, Sannes, Timothy S., Giobbie-Hurder, Anita, Frank, Elizabeth S., McTiernan, Anne, Winer, Eric P., Irwin, Melinda L., Ligibel, Jennifer A.. Exploring anxiety as an influencing factor of the impact of exercise and mind-body prehabilitation on cognitive functioning among women undergoing breast cancer surgery. Journal of psychosocial oncology. 2023. #volume#:1-9 | Duplicate |
| Knight, W., Moore, J., Whyte, G., Zylstra, J., Lane, A., Pate, J., Gervais-Andre, L., Maisey, N., Hill, M., Tham, G., Lagergrens, J., Kelly, M., Baker, C., Van Hemelrijck, M., Goh, V., Gossgae, J., Browning, M., Davies, A.. Long term follow-up of patients undergoing prehabilitation prior to oesophagectomy: what happens to patients declining or withdrawing from prehabilitation exercise programmes?. British journal of surgery. 2023. 110:viii78 | Not a Randomized Trial |
| Khushnood, K., Sultan, N., Awan, M. M. A., Altaf, S., Mehmood, R., Qureshi, S.. Effects of Pre-operative Physical Therapy on Functional Capacity, Kinesiophobia, and Post-operative ICU Stay in Coronary Artery Bypass Grafting Candidates. Iranian rehabilitation journal. 2023. 21:81 | Wrong or Unclear Duration |
| Jafari, H., Bagheri-Nesami, M., Khosravi, S., Habibi, M. R., Tohamtan, R. A. M.. The Effect of Breathing Exercises on Respiratory Condition After Coronary Artery Bypass Surgery. Journal of Nursing and Midwifery Sciences. 2023. 10:8 | Intervention Not Preoperative |
| Hidayat, M., Sagheer, Z., Khifs, S. A., Habani, E. K. I.. EVALUATING THE EFFECTIVENESS OF PERI-OPERATIVE NUTRITIONAL INTERVENTIONS IN ENHANCING SURGICAL RECOVERY IN GENERAL SURGERY PATIENTS. Journal of Population Therapeutics and Clinical Pharmacology. 2023. 30:1818-1824 | Wrong or Unclear Duration |
| Hartman, Y. A. W., Konijnenberg, L. S. F., Dinnissen, D. J. M., Rodwell, L., Li, W. W. L., Nijveldt, R., Van Royen, N., Thijssen, D. H. J.. Handgrip exercise in patients scheduled for cardiac surgery to attenuate troponin release: a feasibility study. American journal of physiology. Heart and circulatory physiology. 2023. 325:H1144 | Wrong or Unclear Duration |
| Guven, B., Ibrahimoglu, O., Calikoglu, I., Yuksel, S., Demir, A., Bektas, H.. THE EFFECT OF PROGRESSIVE RELAXATION EXERCISES ON BARIATRIC SURGERY PERIOD (PREBARI). PRELIMINARY RESULTS OF A PROSPECTIVE RANDOMIZED CONTROLLED CLINICAL TRIAL. Obesity surgery. 2023. 33:322 | Protocol |
| Estrada, J., Aregui, A., Fossey-Diaz, V., Aparicio, T., Cattan, P., Raynaud-Simon, A.. Feasability of a trimodal prehabilitation program in geriatric oncology. Clinical Nutrition ESPEN. 2023. 58:475 | Not a Randomized Trial |
| Du, D., Li, H., Xu, Y., Zheng, T., Xu, X., Wang, J., Tao, R., Wang, J., Yang, Y., Xu, J., Li, J., Jiang, M.. Study on the Effect of Pain Programmed Care Based on the Concept of Prehabilitation on the Recovery of Joint Function and WHOQOL-BREF Score in Elderly Patients after Total Hip Arthroplasty. Alternative therapies in health and medicine. 2023. #volume#:#pages# | Wrong or Unclear Duration |
| Dave, P., Mir, J., Das, A., Lorentz, N., Galetta, M., Lebovic, J., Tretiakov, P., Schoenfeld, A., Onafowokan, O., Passias, P.. 145. Optimizing mental health conditions prior to adult spinal deformity surgery: does preoperative optimization improve surgical outcomes?. Spine journal. 2023. 23:S74 | Wrong or Unclear Duration |
| Coca-Martinez, Miquel, Girsowicz, Elie, Doonan, Robert J., Obrand, Daniel I., Bayne, Jason P., Steinmetz, Oren K., Mackenzie, Kent S., Carli, Francesco, Martinez-Palli, Graciela, Gill, Heather L.. Multimodal Prehabilitation for Peripheral Arterial Disease Patients with Intermittent Claudication-A Pilot Randomized Controlled Trial. Annals of vascular surgery. 2023. #volume#:#pages# | Wrong or no Outcome |
| Chen, B., Yan, X., Wang, X., Mao, Y.. Effectiveness of precise and quantitative rapid pulmonary rehabilitation nursing program for elderly patients with lung cancer during the perioperative period: a randomized controlled trial. Pakistan journal of medical sciences. 2023. 39:572 | Wrong or Unclear Duration |
| Chang, G. J., Gunn, H., Barber, A., Lowenstein, L. M., Dohan, D. P., Broering, J. M., Dockter, T., Tan, A. D., Dueck, A. C., Bailey, L., Chow, S. L. M., Cleary, R. K., Mesleh, M., Woodall, C., Solfelt, M. L., Finlayson, E.. Surgical care and outcomes in older patients with cancer through implementation of a presurgical toolkit (OPTI-Surg): Interim results of a phase III cluster randomized trial (Alliance A231601CD). Journal of Clinical Oncology. 2023. 41:1541 | Wrong or Unclear Duration |
| Cabral, S. M., Oliveira, C., Castro, C., Goncalves, D. M., Irving, S., Santos, L.. Nutritional domain during prehabilitation in the age of ERAS: opportunities during neoadjuvancy for locally advanced gastric cancer. Clinical nutrition ESPEN. 2023. 57:805 | Wrong or no Outcome |
| Bulut, G., Karabulut, N.. The Effects of Breathing Exercises on Patients Having Laparoscopic Cholecystectomy Surgery. Clinical nursing research. 2023. 32:805 | Wrong or Unclear Duration |
| Brown, L. A., Griffiths, J. A., Santer, P., Jakeman, P. M., Smith, T. G.. Potential for using simulated altitude as a means of prehabilitation: a physiology study. Anaesthesia. 2023. 78:1472-1480 | Intervention Not Preoperative |
| Blackwell, James E. M., Gharahdaghi, Nima, Deane, Colleen S., Brook, Matthew S., Williams, John P., Lund, Jonathan N., Atherton, Philip J., Smith, Ken, Wilkinson, Daniel J., Phillips, Bethan E.. Molecular mechanisms underpinning favourable physiological adaptations to exercise prehabilitation for urological cancer surgery. Prostate cancer and prostatic diseases. 2023. #volume#:#pages# | Wrong or no Outcome |
| Bausys, A., Luksta, M., Anglickiene, G., Maneikiene, V. V., Kryzauskas, M., Rybakovas, A., Dulskas, A., Kuliavas, J., Stratilatovas, E., Macijauskiene, L., Simbelyte, T., Celutkiene, J., Jamontaite, I. E., Cirtautas, A., Lenickiene, S., Petrauskiene, D., Cikanaviciute, E., Gaveliene, E., Klimaviciute, G., Rauduvyte, K., Bausys, R., Strupas, K.. Effect of home-based prehabilitation on postoperative complications after surgery for gastric cancer: randomized clinical trial. British Journal of Surgery. 2023. 110:1800-1807 | Duplicate |
| Akowuah, E. F., Wagnild, J. M., Bardgett, M., Prichard, J. G., Mathias, A., Harrison, S. L., Ogundimu, E. O., Hancock, H. C., Maier, R. H., Wilkinson, Chris, Kasim, Adetayo, Witharana, Pasan, Trevis, Jason, Neave, Carmen, Sarginson, Louise, Honeyman, Becky, Walker, Yvonne, Lewis, Sue, Holden, Adrian, Ainsworth, Karen. A randomised controlled trial of prehabilitation in patients undergoing elective cardiac surgery. Anaesthesia. 2023. 78:1120-1128 | Duplicate |
| . A Yoga Program for Patients Undergoing Prostate Cancer Surgery. Developing and Pilot-testing a Yoga Program to Address Post-prostatectomy Side-effects Among Veterans With Prostate Cancer. 2023. #volume#:#pages# | Unregistered Abstract |
| Pragmatic randomized controlled trial of prehabilitation strategy in the elderly patients in the department of hepatobiliary pancreatic surgery. #journal#. 2023. #volume#:#pages# | Unregistered Abstract |
| Preoperative Ketogenic Diet for Reduction of Hepatic Steatosis. Implementation of a Preoperative Ketogenic Diet for Reduction of Hepatic Steatosis Prior to Hepatectomy: a Randomized Control Trial. 2023. #volume#:#pages# | Protocol |
| . Multimodal Prehabilitation for Lung Cancer Surgery. A Novel Multimodal Intervention for Surgical Prehabilitation of Patients With Lung Cancer: the MMP-LUNG Trial. 2023. #volume#:#pages# | Protocol |
| . The Impact of Preoperative Bowel Exercise on Postoperative Bowel Functions in Gynecologic Malignancies. #journal#. 2023. #volume#:#pages# | Protocol |
| . Readiness Brain Operation Optimization Training (ReBOOT) for Epilepsy Surgery. #journal#. 2023. #volume#:#pages# | Protocol |
| . The Effect of a 2-week Preoperative Vegan Diet Versus Omnivorous Diet on the Protein Turnover in the Osteoarthritic Knee. #journal#. 2023. #volume#:#pages# | Protocol |
| . Effects of a Prehabilitation Program in Patients Undergoing Lumbar Radiculopathy Surgery. Effects of a Prehabilitation Program Based on Therapeutic Exercise, Back Care Education and Neuroscience Pain Education in Patients Undergoing Lumbar Radiculopathy Surgery. 2023. #volume#:#pages# | Protocol |
| . Dietary Fiber Before Colorectal Cancer Surgery. FiberUP in Clinical Practice: increasing Preoperative Dietary Fiber Intake in Colorectal Cancer Patients. 2023. #volume#:#pages# | Protocol |
| . Digital Home-Based Prehabilitation Before Surgery. #journal#. 2023. #volume#:#pages# | Protocol |
| . PRIORITY-CONNECT 2 Pilot Trial. Virtual Multimodal Hub for Patients Undergoing Major Gastrointestinal Cancer Surgery - PRIORITY-CONNECT 2 Pilot Randomised Type I Hybrid Effectiveness-Implementation Trial. 2023. #volume#:#pages# | Protocol |
| . Evaluating Pre-Treatment Vestibular Physical Therapy Rehab for Patients With Vestibular Schwannomas. #journal#. 2023. #volume#:#pages# | Protocol |
| Waller, Ellen, Sutton, Paul, Rahman, Seema, Allen, Jonathan, Saxton, John, Aziz, Omer. Prehabilitation with wearables versus standard of care before major abdominal cancer surgery: a randomised controlled pilot study (trial registration: NCT04047524). Surgical Endoscopy & Other Interventional Techniques. 2022. 36:1008-1017 | Duplicate |
| Sassani, Jessica C., Grosse, Philip J., Kunkle, Lauren, Baranski, Lindsey, Ackenbom, Mary F.. Patient Preparedness for Pelvic Organ Prolapse Surgery: A Randomized Equivalence Trial of Preoperative Counseling. Obstetrical & Gynecological Survey. 2022. 77:149-150 | Not Prehabilitation |
| McIsaac, Daniel I., Hladkowicz, Emily, Bryson, Gregory L., Forster, Alan J., Gagne, Sylvain, Huang, Allen, Lalu, Manoj, Lavall&eacute;e, Luke T., Moloo, Husein, Nantel, Julie, Power, Barbara, Scheede-Bergdahl, Celena, van Walraven, Carl, McCartney, Colin J. L., Taljaard, Monica. Home-based prehabilitation with exercise to improve postoperative recovery for older adults with frailty having cancer surgery: the PREHAB randomised clinical trial. BJA: The British Journal of Anaesthesia. 2022. 129:41-48 | Duplicate |
| Rengel, Kimberly F., Mehdiratta, Nitin, Vanston, Susan W., Archer, Kristin R., Jackson, James C., Thompson, Jennifer L., Pandharipande, Pratik P., Hughes, Christopher G.. A randomised pilot trial of combined cognitive and physical exercise prehabilitation to improve outcomes in surgical patients. BJA: The British Journal of Anaesthesia. 2021. 126:e55-e57 | Duplicate |
| Kim, Sunghye, Hsu, Fang-Chi, Groban, Leanne, Williamson, Jeff, Messier, Stephen. A pilot study of aquatic prehabilitation in adults with knee osteoarthritis undergoing total knee arthroplasty - short term outcome. BMC Musculoskeletal Disorders. 2021. 22:1-11 | Duplicate |
| Ijmker‐Hemink, Vera E., Wanten, Geert J. A., Nes, Lindsey C. F., den Berg, Manon G. A.. Effect of a Preoperative Home‐Delivered, Protein‐Rich Meal Service to Improve Protein Intake in Surgical Patients: A Randomized Controlled Trial. JPEN Journal of Parenteral & Enteral Nutrition. 2021. 45:479-489 | Duplicate |
| Fulop, A., Lakatos, L., Susztak, N., Szijarto, A., Banky, B.. The effect of trimodal prehabilitation on the physical and psychological health of patients undergoing colorectal surgery: a randomised clinical trial. Anaesthesia. 2021. 76:82-90 | Duplicate |
| Ferreira, Vanessa, Lawson, Claire, Carli, Francesco, Scheede-Bergdahl, Celena, Chevalier, St&eacute;phanie. Feasibility of a novel mixed-nutrient supplement in a multimodal prehabilitation intervention for lung cancer patients awaiting surgery: A randomized controlled pilot trial. International Journal of Surgery. 2021. 93:N.PAG-N.PAG | Duplicate |
| Bhattacharyya, Ananya, Ramamoorthy, Lakshmi, Pottakkat, Biju. Effect of Pre-operative Nutritional Protocol Implementation on Postoperative Outcomes Following Gastrointestinal Surgeries: A Randomized Clinical Trial. Journal of Caring Science. 2021. 10:177-183 | Duplicate |
| Swaminathan, Nagalakshmi, Kundra, Pankaj, Ravi, Ramya, Kate, Vikram. ERAS protocol with respiratory prehabilitation versus conventional perioperative protocol in elective gastrectomy- a randomized controlled trial. International Journal of Surgery. 2020. 81:149-157 | Duplicate |
| Serper, M., Jones, L. S., Clement, T., Reddy, K., Duarte-Rojo, A., Reese, P. P.. Using Nudges for Liver Transplantation Prehabilitation: Results from PRIMER (Prehabilitation Intervention to Maximize Early Recovery), a Home-Based Behavioral Pilot and Feasibility Trial. American Journal of Transplantation. 2020. 20:1110-1111 | Wrong or no Outcome |
| Kaye, Deborah R., Schafer, Christine, Thelen-Perry, Steven, Parker, Christine, Iglay-Reger, Heidi, Daignault-Newton, Stephanie, Qin, Yongmei, Morgan, Todd M., Weizer, Alon Z., Kaffenberger, Samuel D., Herrel, Lindsey A., Hafez, Khaled S., Lee, Cheryl T., Skolarus, Ted A., Englesbe, Michael J., Montgomery, Jeffrey S.. The Feasibility and Impact of a Presurgical Exercise Intervention Program (Prehabilitation) for Patients Undergoing Cystectomy for Bladder Cancer. Urology. 2020. 145:106-112 | Not a Randomized Trial |
| Au, Darren, Matthew, Andrew G., Lopez, Paty, Hilton, William J., Awasthi, Rashami, Bousquet-Dion, Guillaume, Ladha, Karim, Carli, Franco, Santa Mina, Daniel. Prehabilitation and acute postoperative physical activity in patients undergoing radical prostatectomy: a secondary analysis from an RCT. Sports Medicine - Open. 2019. 5:N.PAG-N.PAG | Duplicate |
| 周晓梅, 宗莉, 陈晓燕, 肖婷, 缪愿戍. 谵妄预防护理方案在老年食管癌手术患者中的应用. Nursing of Integrated Traditional Chinese & Western Medicine. 2018. 4:57-61 | Intervention Not Preoperative |
| Lemanu, D. P., Singh, P. P., Shao, R. Y., Pollock, T. T., MacCormick, A. D., Arroll, B., Hill, A. G.. Text messaging improves preoperative exercise in patients undergoing bariatric surgery. ANZ journal of surgery. 2018. #volume#:#pages# | Wrong Comparator Group |
| Angus Lee, Chun Hin, Murnane, Andrew, Heriot, Alexander G., Ismail, Hilmy, Riedel, Bernhard. Randomized Pilot Study of Enhanced Structured Preoperative Exercise Program for Patients with Rectal Cancer Requiring Neoadjuvant Therapy Before Major Resection. Journal of the American College of Surgeons. 2018. 227:S73-S74 | Wrong or Unclear Duration |
| Salzmann, Stefan, Euteneuer, Frank, Laferton, Johannes A. C., Auer, Charlotte J., Shedden-Mora, Meike C., Schedlowski, Manfred, Moosdorf, Rainer, Rief, Winfried. Effects of preoperative psychological interventions on catecholamineand cortisol levels after surgery in coronary artery bypass graft patients: the randomized controlled PSY-HEART trial. Psychosomatic Medicine. 2017. 79:806-814 | Wrong or Unclear Duration |
| Demark-Wahnefried, Wendy, Nix, Jeffery W., Hunter, Gary R., Rais-Bahrami, Soroush, Desmond, Renee A., Chacko, Balu, Morrow, Casey D., Azrad, Maria, Frug&eacute;, Andrew D., Tsuruta, Yuko, Ptacek, Travis, Tully, Scott A., Segal, Roanne, Grizzle, William E.. Erratum to: Feasibility outcomes of a presurgical randomized controlled trial exploring the impact of caloric restriction and increased physical activity versus a wait-list control on tumor characteristics and circulating biomarkers in men electing prostatectomy for prostate cancer. BMC Cancer. 2017. 17:1-2 | Duplicate |
| Humeidan, Michelle L., Otey, Andrew, Zuleta-Alarcon, Alix, Mavarez-Martinez, Ana, Stoicea, Nicoleta, Bergese, Sergio. Perioperative Cognitive Protection&mdash;Cognitive Exercise and Cognitive Reserve (The Neurobics Trial): A Single-blind Randomized Trial. Clinical Therapeutics. 2015. 37:2641-2650 | No Results |
| Huber, Erika O., Roos, Ewa M., Meichtry, Andr&eacute;, de Bie, Rob A., Bischoff-Ferrari, Heike A.. Effect of preoperative neuromuscular training (NEMEX-TJR) on functional outcome after total knee replacement: an assessor-blinded randomized controlled trial. BMC Musculoskeletal Disorders. 2015. 16:101-101 | Duplicate |
| Tarnoff, M., Rodriguez, L., Escalona, A., Ramos, A., Neto, M., Alamo, M., Reyes, E., Pimentel, F., Ibanez, L.. Open label, prospective, randomized controlled trial of an endoscopic duodenal-jejunal bypass sleeve versus low calorie diet for pre-operative weight loss in bariatric surgery. Surgical Endoscopy & Other Interventional Techniques. 2009. 23:650-656 | Wrong Comparator Group |
| Paquette, Philippe, Higgins, Johanne, Danino, Michel Alain, Harris, Patrick, Lamontagne, Martin, Gagnon, Dany H.. Effects of a preoperative neuromobilization program offered to individuals with carpal tunnel syndrome awaiting carpal tunnel decompression surgery: A pilot randomized controlled study. Journal of hand therapy : official journal of the American Society of Hand Therapists. 2021. 34:37-46 | Wrong Population |
| Llorens, J., Rovira, L., Ballester, M., Moreno, J., Hernandez-Laforet, J., Santonja, F. J., Cassinello, N., Ortega, J.. Preoperative Inspiratory Muscular Training to Prevent Postoperative Hypoxemia in Morbidly Obese Patients Undergoing Laparoscopic Bariatric Surgery. A Randomized Clinical Trial. Obesity Surgery. 2014. #volume#:#pages# | No adherence data |
| Elrefai, M.. Value of preoperative diet before sleeve gastrectomy: A prospective randomized study. Obesity Surgery. 2019. 29:860 | No adherence data |
| Bui, T., Kasvis, P., Vigano, A., Metrakos, P., Chaudhury, P., Barkun, J., Lamoussenery, D., Carli, F., Kilgour, R.. Impact of a trimodal prehabilitation program on functional recovery after hepatobiliary and pancreatic cancer surgery: preliminary findings from a randomized controlled pilot trial. Supportive Care in Cancer. 2019. 27:S240 | No adherence data |
| Tafuri, A., Bassi, S., Sebben, M., Pirozzi, M., Balzarro, M., Porcaro, A. B., Artibani, W., Cerruto, M. A.. A pilot randomized trial of preoperative pelvic floor muscle exercise vs usual care to improve sexual function and health related quality of live after RARP: Preliminary disappointed results. Neurourology and Urodynamics. 2018. 37:S7-S8 | No adherence data |
| Kikuchi, Y., Hiroshima, Y., Matsuo, K., Kawaguchi, D., Murakami, T., Yabushita, Y., Endo, I., Taguri, M., Koda, K., Tanaka, K.. A Randomized Clinical Trial of Preoperative Administration of Branched-Chain Amino Acids to Prevent Postoperative Ascites in Patients with Liver Resection for Hepatocellular Carcinoma. Annals of Surgical Oncology. 2016. 23:3727-3735 | No adherence data |
| Barakat, H. M., Shahin, Y., Khan, J. A., McCollum, P. T., Chetter, I. C.. Preoperative supervised exercise improves outcomes after elective abdominal aortic aneurysm repair. Annals of Surgery. 2016. 264:47-53 | No adherence data |
| Ashida, R., Okamura, Y., Nakao, K., Mizuno, T., Aoki, S., Kiuchi, R., Sugiura, T., Ito, T., Yamamoto, Y., Mochizuki, T., Uesaka, K.. The impact of preoperative enteral nutrition enriched administration with eicosapentaenoic acid (EPA) on postoperative hypercytokinemia after pancreatoduodenectomy: Results of a double-blinded randomized controlled trial. Clinical Nutrition. 2016. 35:S241-S242 | No adherence data |
| Schuetz, T., Peter, V., Garnov, N., Schaudinn, A., Linder, N., Busse, H., Shang, E., Petroff, D., Dietrich, A.. Effect of two preoperative low-energy diets on liver fat content in bariatric patients: A randomized trial. Obesity Facts. 2015. 8:227 | No adherence data |
| Ng, S. L.. A randomised controlled trial study of the efficacy of intensive pre-operative pelvic floor muscle training to decrease post-prostatectomy urinary incontinence. International Journal of Urology. 2014. 21:A169 | No adherence data |
| Villadsen, A., Overgaard, S., Holsgaard-Larsen, A., Christensen, R., Roos, E.. Postoperative effects of neuromuscular exercise prior to hip orknee arthroplasty - A randomised controlled trial. Osteoarthritis and Cartilage. 2013. 21:S34-S35 | No adherence data |
| Schouten, R., Van Der Kaaden, I., Van 't Hof, G., Feskens, P.. Preoperative diets before bariatric surgery: A randomized, single-blinded, non-inferiority trial. Obesity Surgery. 2013. 23:1071 | No adherence data |
| Li, C., Zavorsky, G. S., Kim, D. J., Christou, N. V., Feldman, L. S., Carli, F.. Effects of a bariatric preoperative exercise program: A pilot randomized study. Surgical Endoscopy and Other Interventional Techniques. 2013. 27:S431 | No adherence data |
| Gilbertson, Nicole M., Gaitan, Julian M., Osinski, Victoria, Rexrode, Elizabeth A., Garmey, James C., Mehaffey, J. Hunter, Hassinger, Taryn E., Kranz, Sibylle, McNamara, Coleen A., Weltman, Arthur, Hallowell, Peter T., Malin, Steven K.. Pre-operative aerobic exercise on metabolic health and surgical outcomes in patients receiving bariatric surgery: A pilot trial. PloS one. 2020. 15:e0239130 | No adherence data |
| Swaminathan, Nagalakshmi, Kundra, Pankaj, Ravi, Ramya, Kate, Vikram. ERAS protocol with respiratory prehabilitation versus conventional perioperative protocol in elective gastrectomy- a randomized controlled trial. International journal of surgery (London, England). 2020. 81:149-157 | No adherence data |
| Steinmetz, Carolin, Bjarnason-Wehrens, Birna, Baumgarten, Heike, Walther, Thomas, Mengden, Thomas, Walther, Claudia. Prehabilitation in patients awaiting elective coronary artery bypass graft surgery - effects on functional capacity and quality of life: a randomized controlled trial. Clinical rehabilitation. 2020. 34:1256-1267 | No adherence data |
| Skoffer, Birgit, Maribo, Thomas, Mechlenburg, Inger, Korsgaard, Christian Gaarden, Soballe, Kjeld, Dalgas, Ulrik. Efficacy of preoperative progressive resistance training in patients undergoing total knee arthroplasty: 12-month follow-up data from a randomized controlled trial. Clinical rehabilitation. 2020. 34:82-90 | No adherence data |
| Jantharapattana, Kitti, Orapipatpong, Orachat. Efficacy of EPA-enriched supplement compared with standard formula on body weight changes in malnourished patients with head and neck cancer undergone surgery: a randomized study. Head & neck. 2020. 42:188-197 | No adherence data |
| Ausania, Fabio, Senra, Paula, Melendez, Reyes, Caballeiro, Regina, Ouvina, Ruben, Casal-Nunez, Enrique. Prehabilitation in patients undergoing pancreaticoduodenectomy: a randomized controlled trial. Revista espanola de enfermedades digestivas : organo oficial de la Sociedad Espanola de Patologia Digestiva. 2019. 111:603-608 | No adherence data |
| Lotzke, Hanna, Brisby, Helena, Gutke, Annelie, Hagg, Olle, Jakobsson, Max, Smeets, Rob, Lundberg, Mari. A Person-Centered Prehabilitation Program Based on Cognitive-Behavioral Physical Therapy for Patients Scheduled for Lumbar Fusion Surgery: A Randomized Controlled Trial. Physical therapy. 2019. 99:1069-1088 | No adherence data |
| Vlisides, Phillip E., Das, Abhijit R., Thompson, Allie M., Kunkler, Bryan, Zierau, Mackenzie, Cantley, Michael J., McKinney, Amy M., Giordani, Bruno. Home-based Cognitive Prehabilitation in Older Surgical Patients: A Feasibility Study. Journal of neurosurgical anesthesiology. 2019. 31:212-217 | No adherence data |
| Chakravartty, Saurav, Vivian, Gillian, Mullholland, Nicola, Shaikh, Hizbullah, McGrath, John, Sidhu, Paul S., Jaffer, Ounali, Patel, Ameet G.. Preoperative liver shrinking diet for bariatric surgery may impact wound healing: a randomized controlled trial. Surgery for obesity and related diseases : official journal of the American Society for Bariatric Surgery. 2019. 15:117-125 | No adherence data |
| Kanekiyo, Shinsuke, Takeda, Shigeru, Iida, Michihisa, Nishiyama, Mitsuo, Kitahara, Masahiro, Shindo, Yoshitaro, Tokumitsu, Yukio, Tomochika, Shinobu, Tsunedomi, Ryoichi, Suzuki, Nobuaki, Abe, Toshihiro, Yoshino, Shigefumi, Hazama, Shoichi, Ueno, Tomio, Nagano, Hiroaki. Efficacy of perioperative immunonutrition in esophageal cancer patients undergoing esophagectomy. Nutrition (Burbank, Los Angeles County, Calif.). 2019. 59:96-102 | No adherence data |
| Casana, Jose, Calatayud, Joaquin, Ezzatvar, Yasmin, Vinstrup, Jonas, Benitez, Josep, Andersen, Lars L.. Preoperative high-intensity strength training improves postural control after TKA: randomized-controlled trial. Knee surgery, sports traumatology, arthroscopy : official journal of the ESSKA. 2019. 27:1057-1066 | No adherence data |
| Jahic, Dzenan, Omerovic, Djemil, Tanovic, Adnana Talic, Dzankovic, Fuad, Campara, Merita Tiric. The Effect of Prehabilitation on Postoperative Outcome in Patients Following Primary Total Knee Arthroplasty. Medical archives (Sarajevo, Bosnia and Herzegovina). 2018. 72:439-443 | No adherence data |
| Zhao, Qun, Li, Yong, Yu, Bin, Yang, Peigang, Fan, Liqiao, Tan, Bibo, Tian, Yuan. Effects of Preoperative Enteral Nutrition on Postoperative Recent Nutritional Status in Patients with Siewert II and III Adenocarcinoma of Esophagogastric Junction after Neoadjuvant Chemoradiotherapy. Nutrition and cancer. 2018. 70:895-903 | No adherence data |
| Kong, Seong-Ho, Lee, Hyuk-Joon, Na, Ju-Ri, Kim, Won Gyoung, Han, Dong-Seok, Park, Shin-Hoo, Hong, Hyunsook, Choi, Yunhee, Ahn, Hye Seong, Suh, Yun-Suhk, Yang, Han-Kwang. Effect of perioperative oral nutritional supplementation in malnourished patients who undergo gastrectomy: A prospective randomized trial. Surgery. 2018. 164:1263-1270 | No adherence data |
| Grundmann, Franziska, Muller, Roman-Ulrich, Reppenhorst, Annika, Hulswitt, Lennart, Spath, Martin R., Kubacki, Torsten, Scherner, Maximilian, Faust, Michael, Becker, Ingrid, Wahlers, Thorsten, Schermer, Bernhard, Benzing, Thomas, Burst, Volker. Preoperative Short-Term Calorie Restriction for Prevention of Acute Kidney Injury After Cardiac Surgery: A Randomized, Controlled, Open-Label, Pilot Trial. Journal of the American Heart Association. 2018. 7:#pages# | No adherence data |
| Banerjee, Srijit, Manley, Kate, Shaw, Barnabas, Lewis, Liane, Cucato, Gabriel, Mills, Robert, Rochester, Mark, Clark, Allan, Saxton, John M.. Vigorous intensity aerobic interval exercise in bladder cancer patients prior to radical cystectomy: a feasibility randomised controlled trial. Supportive care in cancer : official journal of the Multinational Association of Supportive Care in Cancer. 2018. 26:1515-1523 | No adherence data |
| Barberan-Garcia, Anael, Ubre, Marta, Roca, Josep, Lacy, Antonio M., Burgos, Felip, Risco, Raquel, Momblan, Dulce, Balust, Jaume, Blanco, Isabel, Martinez-Palli, Graciela. Personalised Prehabilitation in High-risk Patients Undergoing Elective Major Abdominal Surgery: A Randomized Blinded Controlled Trial. Annals of surgery. 2018. 267:50-56 | No adherence data |
| Lai, Yutian, Su, Jianhua, Qiu, Peiyuan, Wang, Mingming, Zhou, Kun, Tang, Yuxin, Che, Guowei. Systematic short-term pulmonary rehabilitation before lung cancer lobectomy: a randomized trial. Interactive cardiovascular and thoracic surgery. 2017. 25:476-483 | No adherence data |
| Au, Darren, Matthew, Andrew G., Lopez, Paty, Hilton, William J., Awasthi, Rashami, Bousquet-Dion, Guillaume, Ladha, Karim, Carli, Franco, Santa Mina, Daniel. Prehabilitation and acute postoperative physical activity in patients undergoing radical prostatectomy: a secondary analysis from an RCT. Sports medicine - open. 2019. 5:18 | No adherence data |
| Vagvolgyi, Attila, Rozgonyi, Zsolt, Kerti, Maria, Agathou, George, Vadasz, Paul, Varga, Janos. Effectiveness of pulmonary rehabilitation and correlations in between functional parameters, extent of thoracic surgery and severity of post-operative complications: randomized clinical trial. Journal of thoracic disease. 2018. 10:3519-3531 | No adherence data |
| Grant, Louise F., Cooper, Derek J., Conroy, Jon L.. The HAPI 'Hip Arthroscopy Pre-habilitation Intervention' study: does pre-habilitation affect outcomes in patients undergoing hip arthroscopy for femoro-acetabular impingement?. Journal of hip preservation surgery. 2017. 4:85-92 | No adherence data |
| Burnand, Katherine M., Lahiri, Rajiv P., Burr, Nicholas, Jansen van Rensburg, Lize, Lewis, Michael P. N.. A randomised, single blinded trial, assessing the effect of a two week preoperative very low calorie diet on laparoscopic cholecystectomy in obese patients. HPB : the official journal of the International Hepato Pancreato Biliary Association. 2016. 18:456-61 | No adherence data |
| Cavill, Stuart, McKenzie, Kylie, Munro, Adrienne, McKeever, Janice, Whelan, Lucy, Biggs, Luke, Skinner, Elizabeth H., Haines, Terry P.. The effect of prehabilitation on the range of motion and functional outcomes in patients following the total knee or hip arthroplasty: A pilot randomized trial. Physiotherapy theory and practice. 2016. 32:262-70 | No adherence data |
| Kaya, Seyda Ors, Akcam, Tevfik Ilker, Ceylan, Kenan Can, Samancilar, Ozgur, Ozturk, Ozgur, Usluer, Ozan. Is preoperative protein-rich nutrition effective on postoperative outcome in non-small cell lung cancer surgery? A prospective randomized study. Journal of cardiothoracic surgery. 2016. 11:14 | No adherence data |
| Yamana, Ippei, Takeno, Shinsuke, Hashimoto, Tatsuya, Maki, Kenji, Shibata, Ryosuke, Shiwaku, Hironari, Shimaoka, Hideki, Shiota, Etsuji, Yamashita, Yuichi. Randomized Controlled Study to Evaluate the Efficacy of a Preoperative Respiratory Rehabilitation Program to Prevent Postoperative Pulmonary Complications after Esophagectomy. Digestive surgery. 2015. 32:331-7 | No adherence data |
| Gade, Hege, Friborg, Oddgeir, Rosenvinge, Jan H., Smastuen, Milada Cvancarova, Hjelmesaeth, Joran. The Impact of a Preoperative Cognitive Behavioural Therapy (CBT) on Dysfunctional Eating Behaviours, Affective Symptoms and Body Weight 1 Year after Bariatric Surgery: A Randomised Controlled Trial. Obesity surgery. 2015. 25:2112-9 | No adherence data |
| Ding, Dayong, Feng, Ye, Song, Bin, Gao, Shuohui, Zhao, Jisheng. Effects of preoperative and postoperative enteral nutrition on postoperative nutritional status and immune function of gastric cancer patients. The Turkish journal of gastroenterology : the official journal of Turkish Society of Gastroenterology. 2015. 26:181-5 | No adherence data |
| Kabata, Pawel, Jastrzebski, Tomasz, Kakol, Michal, Krol, Karolina, Bobowicz, Maciej, Kosowska, Anna, Jaskiewicz, Janusz. Preoperative nutritional support in cancer patients with no clinical signs of malnutrition--prospective randomized controlled trial. Supportive care in cancer : official journal of the Multinational Association of Supportive Care in Cancer. 2015. 23:365-70 | No adherence data |
| Dijkstra-Eshuis, Joke, Van den Bos, Tine W. L., Splinter, Rosa, Bevers, Rob F. M., Zonneveld, Willemijn C. G., Putter, Hein, Pelger, Rob C. M., Voorham-van der Zalm, Petra J.. Effect of preoperative pelvic floor muscle therapy with biofeedback versus standard care on stress urinary incontinence and quality of life in men undergoing laparoscopic radical prostatectomy: a randomised control trial. Neurourology and urodynamics. 2015. 34:144-50 | No adherence data |
| Bergin, Carole, Speroni, Karen Gabel, Travis, Tom, Bergin, John, Sheridan, Michael J., Kelly, Karen, Daniel, Marlon G.. Effect of preoperative incentive spirometry patient education on patient outcomes in the knee and hip joint replacement population. Journal of perianesthesia nursing : official journal of the American Society of PeriAnesthesia Nurses. 2014. 29:20-7 | No adherence data |
| Ocampo-Trujillo, A., Carbonell-Gonzalez, J., Martinez-Blanco, A., Diaz-Hung, A., Munoz, C. A., Ramirez-Velez, R.. Pre-operative training induces changes in the histomorphometry and muscle function of the pelvic floor in patients with indication of radical prostatectomy. Actas urologicas espanolas. 2014. 38:378-84 | No adherence data |
| Kaibori, Masaki, Ishizaki, Morihiko, Matsui, Kosuke, Nakatake, Richi, Yoshiuchi, Sawako, Kimura, Yutaka, Kwon, A. Hon. Perioperative exercise for chronic liver injury patients with hepatocellular carcinoma undergoing hepatectomy. American journal of surgery. 2013. 206:202-9 | No adherence data |
| Geraerts, Inge, Van Poppel, Hendrik, Devoogdt, Nele, Joniau, Steven, Van Cleynenbreugel, Ben, De Groef, An, Van Kampen, Marijke. Influence of preoperative and postoperative pelvic floor muscle training (PFMT) compared with postoperative PFMT on urinary incontinence after radical prostatectomy: a randomized controlled trial. European urology. 2013. 64:766-72 | No adherence data |
| Kim, Do Kyung, Hwang, Ji Hye, Park, Won Hah. Effects of 4 weeks preoperative exercise on knee extensor strength after anterior cruciate ligament reconstruction. Journal of physical therapy science. 2015. 27:2693-6 | No adherence data |
| Tungtrongjit, Yodpiti, Weingkum, Pattira, Saunkool, Piyarat. The effect of preoperative quadriceps exercise on functional outcome after total knee arthroplasty. Journal of the Medical Association of Thailand = Chotmaihet thangphaet. 2012. 95 Suppl 10:S58-66 | No adherence data |
| Soni, Anushka, Joshi, Abhay, Mudge, Nicola, Wyatt, Matthew, Williamson, Lyn. Supervised exercise plus acupuncture for moderate to severe knee osteoarthritis: a small randomised controlled trial. Acupuncture in medicine : journal of the British Medical Acupuncture Society. 2012. 30:176-81 | No adherence data |
| Huang, S. W., Chen, P. H., Chou, Y. H.. Effects of a preoperative simplified home rehabilitation education program on length of stay of total knee arthroplasty patients. Orthopaedics & traumatology, surgery & research : OTSR. 2012. 98:259-64 | No adherence data |
| Barbalho-Moulim, Marcela Cangussu, Miguel, Gustavo Peixoto Soares, Forti, Eli Maria Pazzianotto, Campos, Flavio do Amaral, Costa, Dirceu. Effects of preoperative inspiratory muscle training in obese women undergoing open bariatric surgery: respiratory muscle strength, lung volumes, and diaphragmatic excursion. Clinics (Sao Paulo, Brazil). 2011. 66:1721-7 | No adherence data |
| Pehlivan, Esra, Turna, Akif, Gurses, Atilla, Gurses, Hulya Nilgun. The effects of preoperative short-term intense physical therapy in lung cancer patients: a randomized controlled trial. Annals of thoracic and cardiovascular surgery : official journal of the Association of Thoracic and Cardiovascular Surgeons of Asia. 2011. 17:461-8 | No adherence data |
| Rosenfeldt, Franklin, Braun, Lesley, Spitzer, Ondine, Bradley, Scott, Shepherd, Judy, Bailey, Michael, van der Merwe, Juliana, Leong, Jee-Yoong, Esmore, Donald. Physical conditioning and mental stress reduction--a randomised trial in patients undergoing cardiac surgery. BMC complementary and alternative medicine. 2011. 11:20 | No adherence data |
| Gstoettner, Michaela, Raschner, Christian, Dirnberger, Eva, Leimser, Hannes, Krismer, Martin. Preoperative proprioceptive training in patients with total knee arthroplasty. The Knee. 2011. 18:265-70 | No adherence data |
| Carbajo, M. A., Castro, Maria J., Kleinfinger, S., Gomez-Arenas, S., Ortiz-Solorzano, J., Wellman, R., Garcia-Ianza, C., Luque, E.. Effects of a balanced energy and high protein formula diet (Vegestart complet R) vs. low-calorie regular diet in morbid obese patients prior to bariatric surgery (laparoscopic single anastomosis gastric bypass): a prospective, double-blind randomized study. Nutricion hospitalaria. 2010. 25:939-48 | No adherence data |
| Ishikawa, Yoshinori, Yoshida, Hiroshi, Mamada, Yasuhiro, Taniai, Nobuhiko, Matsumoto, Satoshi, Bando, Koichi, Mizuguchi, Yoshiaki, Kakinuma, Daisuke, Kanda, Tomohiro, Tajiri, Takashi. Prospective randomized controlled study of short-term perioperative oral nutrition with branched chain amino acids in patients undergoing liver surgery. Hepato-gastroenterology. 2010. 57:583-90 | No adherence data |
| Kulkarni, S. R., Fletcher, E., McConnell, A. K., Poskitt, K. R., Whyman, M. R.. Pre-operative inspiratory muscle training preserves postoperative inspiratory muscle strength following major abdominal surgery - a randomised pilot study. Annals of the Royal College of Surgeons of England. 2010. 92:700-7 | No adherence data |
| Stein, Traci R., Olivo, Erin L., Grand, Sandy Hermele, Namerow, Pearila B., Costa, Joseph, Oz, Mehmet C.. A pilot study to assess the effects of a guided imagery audiotape intervention on psychological outcomes in patients undergoing coronary artery bypass graft surgery. Holistic nursing practice. 2010. 24:213-22 | No adherence data |
| Centemero, Antonia, Rigatti, Lorenzo, Giraudo, Donatella, Lazzeri, Massimo, Lughezzani, Giovanni, Zugna, Daniela, Montorsi, Francesco, Rigatti, Patrizio, Guazzoni, Giorgio. Preoperative pelvic floor muscle exercise for early continence after radical prostatectomy: a randomised controlled study. European urology. 2010. 57:1039-43 | No adherence data |
| Okabayashi, Takehiro, Nishimori, Isao, Yamashita, Koichi, Sugimoto, Takeki, Namikawa, Tsutomu, Maeda, Hiromichi, Yatabe, Tomoaki, Hanazaki, Kazuhiro. Preoperative oral supplementation with carbohydrate and branched-chain amino acid-enriched nutrient improves insulin resistance in patients undergoing a hepatectomy: a randomized clinical trial using an artificial pancreas. Amino acids. 2010. 38:901-7 | No adherence data |
| Topp, Robert, Swank, Ann M., Quesada, Peter M., Nyland, John, Malkani, Arthur. The effect of prehabilitation exercise on strength and functioning after total knee arthroplasty. PM & R : the journal of injury, function, and rehabilitation. 2009. 1:729-35 | No adherence data |
| Okamoto, Yoshiki, Okano, Keiichi, Izuishi, Kunihiko, Usuki, Hisashi, Wakabayashi, Hisao, Suzuki, Yasuyuki. Attenuation of the systemic inflammatory response and infectious complications after gastrectomy with preoperative oral arginine and omega-3 fatty acids supplemented immunonutrition. World journal of surgery. 2009. 33:1815-21 | No adherence data |
| Ferreira, Paulo Eduardo Gomes, Rodrigues, Alfredo Jose, Evora, Paulo Roberto Barboza. Effects of an inspiratory muscle rehabilitation program in the postoperative period of cardiac surgery. Arquivos brasileiros de cardiologia. 2009. 92:275-82 | No adherence data |
| Parker, Patricia A., Pettaway, Curtis A., Babaian, Richard J., Pisters, Louis L., Miles, Brian, Fortier, Adoneca, Wei, Qi, Carr, Danielle D., Cohen, Lorenzo. The effects of a presurgical stress management intervention for men with prostate cancer undergoing radical prostatectomy. Journal of clinical oncology : official journal of the American Society of Clinical Oncology. 2009. 27:3169-76 | No adherence data |
| Furze, Gill, Dumville, Jo C., Miles, Jeremy N. V., Irvine, Karen, Thompson, David R., Lewin, Robert J. P.. "Prehabilitation" prior to CABG surgery improves physical functioning and depression. International journal of cardiology. 2009. 132:51-8 | No adherence data |
| Goodman, Helen, Parsons, Amanda, Davison, June, Preedy, Michael, Peters, Emma, Shuldham, Caroline, Pepper, John, Cowie, Martin R.. A randomised controlled trial to evaluate a nurse-led programme of support and lifestyle management for patients awaiting cardiac surgery 'Fit for surgery: Fit for life' study. European journal of cardiovascular nursing : journal of the Working Group on Cardiovascular Nursing of the European Society of Cardiology. 2008. 7:189-95 | No adherence data |
| Dronkers, Jaap, Veldman, Andre, Hoberg, Ellen, van der Waal, Cees, van Meeteren, Nico. Prevention of pulmonary complications after upper abdominal surgery by preoperative intensive inspiratory muscle training: a randomized controlled pilot study. Clinical rehabilitation. 2008. 22:134-42 | No adherence data |
| Hulzebos, Erik H. J., Helders, Paul J. M., Favie, Nine J., De Bie, Rob A., Brutel de la Riviere, Aart, Van Meeteren, Nico L. U.. Preoperative intensive inspiratory muscle training to prevent postoperative pulmonary complications in high-risk patients undergoing CABG surgery: a randomized clinical trial. JAMA. 2006. 296:1851-7 | No adherence data |
| Xu, Jianmin, Zhong, Yunshi, Jing, Dayong, Wu, Zhaohan. Preoperative enteral immunonutrition improves postoperative outcome in patients with gastrointestinal cancer. World journal of surgery. 2006. 30:1284-9 | No adherence data |
| Burgio, Kathryn L., Goode, Patricia S., Urban, Donald A., Umlauf, Mary G., Locher, Julie L., Bueschen, Anton, Redden, David T.. Preoperative biofeedback assisted behavioral training to decrease post-prostatectomy incontinence: a randomized, controlled trial. The Journal of urology. 2006. 175:196-201 | No adherence data |
| Jarvis, Sherin K., Hallam, Taryn K., Lujic, Sanja, Abbott, Jason A., Vancaillie, Thierry G.. Peri-operative physiotherapy improves outcomes for women undergoing incontinence and or prolapse surgery: results of a randomised controlled trial. The Australian & New Zealand journal of obstetrics & gynaecology. 2005. 45:300-3 | No adherence data |
| Mitchell, Caroline, Walker, Jane, Walters, Stephen, Morgan, Anne B., Binns, Teena, Mathers, Nigel. Costs and effectiveness of pre- and post-operative home physiotherapy for total knee replacement: randomized controlled trial. Journal of evaluation in clinical practice. 2005. 11:283-92 | No adherence data |
| Smedley, F., Bowling, T., James, M., Stokes, E., Goodger, C., O'Connor, O., Oldale, C., Jones, P., Silk, D.. Randomized clinical trial of the effects of preoperative and postoperative oral nutritional supplements on clinical course and cost of care. The British journal of surgery. 2004. 91:983-90 | No adherence data |
| Gocen, Zeliha, Sen, Ayse, Unver, Bayram, Karatosun, Vasfi, Gunal, Izge. The effect of preoperative physiotherapy and education on the outcome of total hip replacement: a prospective randomized controlled trial. Clinical rehabilitation. 2004. 18:353-8 | No adherence data |
| Futter, C. M., Weiler-Mithoff, E., Hagen, S., Van de Sijpe, K., Coorevits, P. L., Litherland, J. C., Webster, M. H. C., Hamdi, M., Blondeel, P. N.. Do pre-operative abdominal exercises prevent post-operative donor site complications for women undergoing DIEP flap breast reconstruction? A two-centre, prospective randomised controlled trial. British journal of plastic surgery. 2003. 56:674-83 | No adherence data |
| van Bokhorst-De Van Der Schueren, M. A., Quak, J. J., von Blomberg-van der Flier, B. M., Kuik, D. J., Langendoen, S. I., Snow, G. B., Green, C. J., van Leeuwen, P. A.. Effect of perioperative nutrition, with and without arginine supplementation, on nutritional status, immune function, postoperative morbidity, and survival in severely malnourished head and neck cancer patients. The American journal of clinical nutrition. 2001. 73:323-32 | No adherence data |
| Bales, G. T., Gerber, G. S., Minor, T. X., Mhoon, D. A., McFarland, J. M., Kim, H. L., Brendler, C. B.. Effect of preoperative biofeedback/pelvic floor training on continence in men undergoing radical prostatectomy. Urology. 2000. 56:627-30 | No adherence data |
| MacFie, J., Woodcock, N. P., Palmer, M. D., Walker, A., Townsend, S., Mitchell, C. J.. Oral dietary supplements in pre- and postoperative surgical patients: a prospective and randomized clinical trial. Nutrition (Burbank, Los Angeles County, Calif.). 2000. 16:723-8 | No adherence data |
| Arthur, H. M., Daniels, C., McKelvie, R., Hirsh, J., Rush, B.. Effect of a preoperative intervention on preoperative and postoperative outcomes in low-risk patients awaiting elective coronary artery bypass graft surgery. A randomized, controlled trial. Annals of internal medicine. 2000. 133:253-62 | No adherence data |
| Braga, M., Gianotti, L., Radaelli, G., Vignali, A., Mari, G., Gentilini, O., Di Carlo, V.. Perioperative immunonutrition in patients undergoing cancer surgery: results of a randomized double-blind phase 3 trial. Archives of surgery (Chicago, Ill. : 1960). 1999. 134:428-33 | No adherence data |
| Braga, M., Gianotti, L., Vignali, A., Di Carlo, V.. Immunonutrition in gastric cancer surgical patients. Nutrition (Burbank, Los Angeles County, Calif.). 1998. 14:831-5 | No adherence data |
| Weiner, P., Zeidan, F., Zamir, D., Pelled, B., Waizman, J., Beckerman, M., Weiner, M.. Prophylactic inspiratory muscle training in patients undergoing coronary artery bypass graft. World journal of surgery. 1998. 22:427-31 | No adherence data |
| Weidenhielm, L., Mattsson, E., Brostrom, L. A., Wersall-Robertsson, E.. Effect of preoperative physiotherapy in unicompartmental prosthetic knee replacement. Scandinavian journal of rehabilitation medicine. 1993. 25:33-9 | No adherence data |
| Roukema, J. A., Carol, E. J., Prins, J. G.. The prevention of pulmonary complications after upper abdominal surgery in patients with noncompromised pulmonary status. Archives of surgery (Chicago, Ill. : 1960). 1988. 123:30-4 | No adherence data |
| Yildiz Sy, Yazicioglu M. B. Tiryaki C. Ciftci A. Boyacioglu Z.. The effect of enteral immunonutrition in upper gastrointestinalsurgery for cancer: a prospective study. Turkish journal of medical sciences. 2016. 46:393 | No adherence data |
| Aytekin E, Sukur E. Oz N. Telatar A. Eroglu Demir S. Sayiner Caglar N. Ozturkmen Y. Ozgonenel L.. The effect of a 12 week prehabilitation program on pain and function for patients undergoing total knee arthroplasty: a prospective controlled study. The effect of a 12 week prehabilitation program on pain and function for patients undergoing total knee arthroplasty: a prospective controlled study. 2018. #volume#:#pages# | No adherence data |
| Argunova Ya, Korotkevich A. A. Pomeshkina S. A. Kokov A. N. Inozemtseva A. A. Barbarash O. L.. Efficacy of physical trainings as cardioprotection method for coronary bypass surgery. Efficacy of physical trainings as cardioprotection method for coronary bypass surgery. 2018. 23:159 | No adherence data |
| Fulop A, Lakatos L. Susztak N. Szijarto A. Banky B.. The effect of trimodal prehabilitation on the physical and psychological health of patients undergoing colorectal surgery: a randomised clinical trial. Anaesthesia. 2020. #volume#:#pages# | No adherence data |
| Gonzalez L, Soto J. Leyton B. Cancino J. Olivares M. Pino J. Cancino J.. Isocaloric high intensity interval training and continuous training on body composition and fitness in bariatric surgery candidates with morbid obesity. Obesity surgery. 2019. 29:607 | No adherence data |
| Da Cunha Fmr, Ruas G. Fanan J. M. V. Crema E. Volpe M. S.. Effects of preoperative respiratory muscle training on early and late postoperative outcome of patients undergoing esophageal surgery. Intensive care medicine. 2013. 39:S369 | No adherence data |
| Zdunski S, Rongies W. Ziolkowski M. Koziel T. Kazimierski P. Halaj R. Sierdzinski J.. Assessment of knee joint range of motion and the level of pain in patients after arthroscopic ACL reconstruction in the selected physiotherapy model. Ocena zakresu ruchu stawu kolanowego (ROM) oraz poziomu dolegliwosci bolowych (VAA) u pacjentow po artroskopowej rekonstrukcji wiȩzadla krzyzowego przedniego w wybranym modelu usprawniania. 2017. 31:41 | No adherence data |
| Manasek V, Bezdek K.. Perioperative oral nutritional support in colorectal cancer patients may improve clinical and health economics outcomes. Perioperative oral nutritional support in colorectal cancer patients may improve clinical and health economics outcomes.. 2015. 26:iv88 | No adherence data |
| Brosky T, Topp R. Finley M. Killian C. Pariser D. Brown K. Bloemer G. Stearns Z.. Effects of prehabilitation on early rehabilitation outcomes following total knee arthroplasty in patients with knee osteoarthritis. Physiotherapy (United Kingdom). 2011. 97:eS160 | No adherence data |
| Dambrauskas Z, Maleckas A. Van Nieuwenhove Y. Thorell A.. The effects of short-term preoperative very low calorie diet (VLCD) on long-term outcomes after laparoscopic roux-en-y gastric bypass for morbid obesity. Obesity surgery. 2013. 23:1184 | No adherence data |
| Gloor, Severin, Misirlic, Merima, Frei-Lanter, Cornelia, Herzog, Pascal, Muller, Phaedra, Schafli-Thurnherr, Judit, Lamdark, Tenzin, Schregel, Dorothee, Wyss, Roland, Unger, Ines, Gisi, David, Greco, Nicola, Mungo, Giuseppe, Wirz, Markus, Raptis, Dimitri Aristotle, Tschuor, Christoph, Breitenstein, Stefan. Prehabilitation in patients undergoing colorectal surgery fails to confer reduction in overall morbidity: results of a single-center, blinded, randomized controlled trial. Langenbeck's archives of surgery. 2022. #volume#:#pages# | No adherence data |
| Kaushik, Dharam, Shah, Pankil K., Mukherjee, Neelam, Ji, Niannian, Dursun, Furkan, Kumar, Addanki P., Thompson, Ian M., Jr., Mansour, Ahmed M., Jha, Richapriya, Yang, Xiaoyu, Wang, Hanzhang, Darby, Nydia, Ricardo Rivero, J., Svatek, Robert S., Liss, Michael A.. Effects of yoga in men with prostate cancer on quality of life and immune response: a pilot randomized controlled trial. Prostate cancer and prostatic diseases. 2021. #volume#:#pages# | No adherence data |
| Lee, Soo Young, Lee, Jaram, Park, Hyeong-Min, Kim, Chang Hyun, Kim, Hyeong Rok. Impact of Preoperative Immunonutrition on the Outcomes of Colon Cancer Surgery: Results from a Randomized Controlled Trial. Annals of surgery. 2021. #volume#:#pages# | No adherence data |
| Veshnavei, Hossein Abdollahi. Urinary incontinency after radical prostatectomy and effects of 1 month pre-operative biofeedback training. American journal of clinical and experimental urology. 2021. 9:489-496 | No adherence data |
| Berkel, Annefleur E. M., Bongers, Bart C., Kotte, Hayke, Weltevreden, Paul, de Jongh, Frans H. C., Eijsvogel, Michiel M. M., Wymenga, Machteld, Bigirwamungu-Bargeman, Marloes, van der Palen, Job, van Det, Marc J., van Meeteren, Nico L. U., Klaase, Joost M.. Effects of Community-based Exercise Prehabilitation for Patients Scheduled for Colorectal Surgery With High Risk for Postoperative Complications: Results of a Randomized Clinical Trial. Annals of surgery. 2022. 275:e299-e306 | No adherence data |
| Shahood, Hadel, Pakai, Annamaria, Rudolf, Kiss, Bory, Eva, Szilagyi, Noemi, Sandor, Adrienn, Zsofia, Verzar. The effect of preoperative chest physiotherapy on oxygenation and lung function in cardiac surgery patients: a randomized controlled study. Annals of Saudi medicine. 2022. 42:8-16 | No adherence data |
| Bhattacharyya, Ananya, Ramamoorthy, Lakshmi, Pottakkat, Biju. Effect of Pre-operative Nutritional Protocol Implementation on Postoperative Outcomes Following Gastrointestinal Surgeries: A Randomized Clinical Trial. Journal of caring sciences. 2021. 10:177-183 | No adherence data |
| Lopez-Rodriguez-Arias, Francisco, Sanchez-Guillen, Luis, Aranaz-Ostariz, Veronica, Triguero-Canovas, Daniel, Lario-Perez, Sandra, Barber-Valles, Xavier, Lacueva, Francisco J., Ramirez, Jose M., Arroyo, Antonio. Effect of home-based prehabilitation in an enhanced recovery after surgery program for patients undergoing colorectal cancer surgery during the COVID-19 pandemic. Supportive care in cancer : official journal of the Multinational Association of Supportive Care in Cancer. 2021. 29:7785-7791 | No adherence data |
| Sittitrai, Pichit, Ruenmarkkaew, Donyarat, Booyaprapa, Somkamol, Kasempitakpong, Boosita. Effect of a perioperative immune-enhancing diet in clean-contaminated head and neck cancer surgery: A randomized controlled trial. International journal of surgery (London, England). 2021. 93:106051 | No adherence data |
| Garcia-Delgado, Yaiza, Lopez-Madrazo-Hernandez, Maria Jose, Alvarado-Martel, Dacil, Miranda-Calderin, Guillermo, Ugarte-Lopetegui, Arantza, Gonzalez-Medina, Raul Alberto, Hernandez-Lazaro, Alba, Zamora, Garlene, Perez-Martin, Nuria, Sanchez-Hernandez, Rosa Maria, Ibarra-Gonzalez, Adriana, Bengoa-Dolon, Monica, Mendoza-Vega, Carmen Teresa, Appelvik-Gonzalez, Svein Mikael, Caballero-Diaz, Yurena, Hernandez-Hernandez, Juan Ramon, Wagner, Ana Maria. Prehabilitation for Bariatric Surgery: A Randomized, Controlled Trial Protocol and Pilot Study. Nutrients. 2021. 13:#pages# | No adherence data |
| Rengel, Kimberly F., Mehdiratta, Nitin, Vanston, Susan W., Archer, Kristin R., Jackson, James C., Thompson, Jennifer L., Pandharipande, Pratik P., Hughes, Christopher G.. A randomised pilot trial of combined cognitive and physical exercise prehabilitation to improve outcomes in surgical patients. British journal of anaesthesia. 2021. 126:e55-e57 | No adherence data |
| Ijmker-Hemink, Vera E., Wanten, Geert J. A., de Nes, Lindsey C. F., van den Berg, Manon G. A.. Effect of a Preoperative Home-Delivered, Protein-Rich Meal Service to Improve Protein Intake in Surgical Patients: A Randomized Controlled Trial. JPEN. Journal of parenteral and enteral nutrition. 2021. 45:479-489 | No adherence data |
| Paquette, Philippe, Higgins, Johanne, Danino, Michel Alain, Harris, Patrick, Lamontagne, Martin, Gagnon, Dany H.. Effects of a preoperative neuromobilization program offered to individuals with carpal tunnel syndrome awaiting carpal tunnel decompression surgery: A pilot randomized controlled study. Journal of hand therapy : official journal of the American Society of Hand Therapists. 2021. 34:37-46 | No adherence data |
| Liljensoe, A., Laursen, J. O., Bliddal, H., Soballe, K., Mechlenburg, I.. Weight Loss Intervention Before Total Knee Replacement: A 12-Month Randomized Controlled Trial. Scandinavian journal of surgery : SJS : official organ for the Finnish Surgical Society and the Scandinavian Surgical Society. 2021. 110:3-12 | No adherence data |
| Patel Ys, Churchill I. F. Sullivan K. A. Beauchamp M. Wald J. Mbuagbaw L. Agzarian J. Shargall Y. Finley C. J. Fahim C. Hanna W. C.. OA04.01 Move For Surgery ? A Novel Preconditioning Program to Optimize Health Before Thoracic Surgery: a Randomized Controlled Trial. Journal of thoracic oncology. 2021. 16:S852 | No adherence data |
| Chen, Z. H., Lin, S. Y., Dai, Q. B., Hua, J., Chen, S. Q.. The Effects of Pre-Operative Enteral Nutrition from Nasal Feeding Tubes on Gastric Outlet Obstruction. Nutrients. 2017. 9:9 | No adherence data |
| Sifuentes, A. D. M., Flores, D. S., Villegas, L. H.. Effect of pre-habilitation on quality of life and post-operation fatigue syndrome in Medico Nacional-Leon IMSS de Leon Guanajuato. Revista Hispanoamericana De Hernia. 2018. 6:11-16 | No adherence data |
| Sawatzky, J. A. V., Kehler, D. S., Ready, A. E., Lerner, N., Boreskie, S., Lamont, D., Luchik, D., Arora, R. C., Duhamel, T. A.. Prehabilitation program for elective coronary artery bypass graft surgery patients: a pilot randomized controlled study. Clinical Rehabilitation. 2014. 28:648-657 | No adherence data |
| Garcia, R. S., Yanez-Brage, M. I., Moolhuyzen, E. G., Riobo, M. S., Paz, A. L., Mate, J. M. B.. Preoperative exercise training prevents functional decline after lung resection surgery: a randomized, single-blind controlled trial. Clinical Rehabilitation. 2017. 31:1057-1067 | No adherence data |
| Faria, S. L., Faria, O. P., Cardeal, M. D., Ito, M. K.. Effects of a very low calorie diet in the preoperative stage of bariatric surgery: a randomized trial. Surgery for Obesity and Related Diseases. 2015. 11:230-237 | No adherence data |
| Knoerl, R., Giobbie-Hurder, A., Sannes, T. S., Chagpar, A. B., Dillon, D., Dominici, L. S., Frank, E. S., Golshan, M., McTiernan, A., Rhei, E., Tolaney, S. M., Winer, E. P., Yung, R. C. L., Irwin, M. L., Ligibel, J. A.. Exploring the impact of exercise and mind-body prehabilitation interventions on physical and psychological outcomes in women undergoing breast cancer surgery. Supportive Care in Cancer. 2022. 30:2027-2036 | No adherence data |
| Liang, M. K., Bernardi, K., Holihan, J. L., Cherla, D. V., Escamilla, R., Lew, D. F., Berger, D. H., Ko, T. C., Kao, L. S.. Modifying Risks in Ventral Hernia Patients With Prehabilitation A Randomized Controlled Trial. Annals of Surgery. 2018. 268:674-680 | No adherence data |
| Ma, R. C., Zhao, Y., Liu, X., Cao, H. P., Wang, Y. O., Yin, Y. Y., Xie, J.. Multimodal Exercise Program A pilot randomized trial for patients with lung cancer receiving surgical treatment. Clinical Journal of Oncology Nursing. 2021. 25:E26-E34 | No adherence data |
| de Luis, D. A., Izaola, O., Alonso, M. G., Aller, R., Cabezas, G., de la Fuente, B.. Effect of a commercial hypocaloric diet in weight loss and post surgical morbidities in obese patients with chronic arthropathy, a randomized clinical trial. European Review for Medical and Pharmacological Sciences. 2012. 16:1814-1820 | No adherence data |
| Aunger, J. A., Greaves, C. J., Davis, E. T., Asama, E. A., Whittaker, A. C., Greig, C. A.. A novel behavioural INTErvention to REduce Sitting Time in older adults undergoing orthopaedic surgery (INTEREST): results of a randomised-controlled feasibility study. Aging Clinical and Experimental Research. 2020. 32:2565-2585 | No adherence data |
| Bond, D., Thomas, J., Vithiananthan, S., Unick, J., Webster, J., Roye, G., Ryder, B., Sax, H.. Intervention-related increases in preoperative physical activity are maintained 6-months after Bariatric surgery: results from the bari-active trial. International Journal of Obesity. 2017. 41:467-470 | No adherence data |
| Ding, D. Y., Wang, D., Shu, Z. B.. Effects of Preoperative Enteral Nutrition Support on Postoperative Nutritional Status and Immune Function of Colorectal Cancer Patients. 4th International Conference on Management Science, Education Technology, Arts, Social Science and Economics (MSETASSE). 2016. 85:1282-1287 | No adherence data |
| Ismail, Msme, Sharifudin, M. A., Shokri, A. A., Ab Rahman, S.. Preoperative physiotherapy and short-term functional outcomes of primary total knee arthroplasty. Singapore Medical Journal. 2016. 57:138-143 | No adherence data |
| An, J., Ryu, H. K., Lyu, S. J., Yi, H. J., Lee, B. H.. Effects of Preoperative Telerehabilitation on Muscle Strength, Range of Motion, and Functional Outcomes in Candidates for Total Knee Arthroplasty: A Single-Blind Randomized Controlled Trial. International Journal of Environmental Research and Public Health. 2021. 18:15 | No adherence data |
| Abdelaal, G. A., Eldahdouh, S. S., Abdelsamie, M., Labeeb, A.. Effect of preoperative physical and respiratory therapy on postoperative pulmonary functions and complications after laparoscopic upper abdominal surgery in obese patients. Egyptian Journal of Chest Diseases and Tuberculosis. 2017. 66:735-738 | No adherence data |
| Kim, S., Hsu, F. C., Groban, L., Williamson, J., Messier, S.. A pilot study of aquatic prehabilitation in adults with knee osteoarthritis undergoing total knee arthroplasty - short term outcome. Bmc Musculoskeletal Disorders. 2021. 22:11 | No adherence data |
| Dlima, D. D., Colwell, C. W., Morris, B. A., Hardwick, M. E., Kozin, F.. The effect of preoperative exercise on total knee replacement outcomes. Clinical Orthopaedics and Related Research. 1996. #volume#:174-182 | No adherence data |
| Allameh, Farzad, Rayegani, Seyed Mansoor, Razzaghi, Mohammadreza, Abedi, Amir Reza, Rahavian, Amirhossein, Javadi, Atefeh, Montazeri, Saeed. Comparison of the effect of the pelvic floor muscle biofeedback prior or postradical prostatectomy on urinary incontinence: A randomized controlled trial. Turkish Journal of Urology. 2021. 47:736-441 | No adherence data |
| Yeğen, Serkan Fatih, Kafadar, Mehmet Tolga, Gök, Mehmet Ali. Comparison of Perioperative Standard and Immunomodulating Enteral Nutrition in Patients Received Major Abdominal Cancer Surgery: a Prospective, Randomized, Controlled Clinical Trial. Indian Journal of Surgery. 2020. 82:828-834 | No adherence data |
| van Noort, Harm H. J., Witteman, Ben J. M., Vermeulen, Hester, Huisman-de Waal, Getty. An outpatient nursing nutritional intervention to prehabilitate undernourished patients planned for surgery: A multicentre, cluster-randomised pilot study. Clinical Nutrition. 2020. 39:2420-2427 | No adherence data |
| Reddy, D. V. S., Kamath, S. U., Annappa, R., Krishnamurthy, S. L., Kamath, K., Mallya, S.. Does Preoperative Rehabilitation give Better Short Term Results in Anterior Cruciate Ligament Reconstruction?. Ambulatory Surgery. 2020. 26:40-43 | No adherence data |
| Pellegrini, Christine A., Chang, Rowland W., Dunlop, Dorothy D., Conroy, David E., Lee, Jungwha, Van Horn, Linda, Spring, Bonnie, Cameron, Kenzie A.. Comparison of a Patient-Centered Weight Loss Program starting before versus after knee replacement: A pilot study. Obesity Research & Clinical Practice. 2018. 12:472-478 | No adherence data |
| Calatayud, Joaquin, Casaña, Jose, Ezzatvar, Yasmin, Jakobsen, Markus, Sundstrup, Emil, Andersen, Lars, Casaña, Jose, Jakobsen, Markus D., Andersen, Lars L.. High-intensity preoperative training improves physical and functional recovery in the early post-operative periods after total knee arthroplasty: a randomized controlled trial. Knee Surgery, Sports Traumatology, Arthroscopy. 2017. 25:2864-2872 | No adherence data |
| 赖玉田, 苏建华, 杨梅, 周坤, 车国卫. 术前短期综合肺康复训练对肺癌合并轻中度 慢性阻塞性肺病患者的影响： 一项前瞻性随机对照试验. Chinese Journal of Lung Cancer. 2016. 19:746-753 | No adherence data |
| Elmarakby, A.. Effect of Threshold Inspiratory Muscle Training on Maximal Inspiratory Pressure and Pulmonary Gas Exchange in Patients Undergoing Coronary Artery Bypass Graft Surgery. Critical Reviews in Physical & Rehabilitation Medicine. 2016. 28:249-261 | No adherence data |
| van Leeuwen, D. M., de Ruiter, C. J., Nolte, P. A., de Haan, A.. Preoperative Strength Training for Elderly Patients Awaiting Total Knee Arthroplasty. Rehabilitation Research & Practice. 2014. #volume#:1-9 | No adherence data |
| Shaarani, Shahril R., O’Hare, Christopher, Quinn, Alison, Moyna, Niall, Moran, Raymond, O’Byrne, John M.. Effect of Prehabilitation on the Outcome of Anterior Cruciate Ligament Reconstruction. American Journal of Sports Medicine. 2013. 41:2117-2127 | No adherence data |
| Lier, Hø, Biringer, E., Stubhaug, B., Tangen, T.. The impact of preoperative counseling on postoperative treatment adherence in bariatric surgery patients: A randomized controlled trial. Patient Education & Counseling. 2012. 87:336-342 | No adherence data |
| McHugh, G.. The role of perhabilitation on the outcome of total knee arthroplasty: A randomized control trial. #journal#. 2011. M.D.:N.PAG p-N.PAG p | No adherence data |
| Buijs, N., van Bokhorst-de van der Schueren, M. A., Langius, J. A., Leemans, C. R., Kuik, D. J., Vermeulen, M. A., van Leeuwen, P. A.. Perioperative arginine-supplemented nutrition in malnourished patients with head and neck cancer improves long-term survival. American Journal of Clinical Nutrition. 2010. 92:1151-1156 | No adherence data |
| Funderburk, J. A., Callis, S.. Aquatic intervention effect on quality of life prior to obesity surgery: a pilot study. Annual in Therapeutic Recreation. 2010. 18:66-78 | No adherence data |
| Ferrara, P. E., Rabini, A., Aprile, I., Maggi, L., Piazzini, D. B., Logroscino, G., Lombi, G. M., Amabile, E., Tancredi, G., Aulisa, A. G., Padua, L., Bertolini, C.. Effect of pre-operative physiotherapy in patients with end-stage osteoarthritis undergoing hip arthroplasty [corrected] [published erratum appears in CLIN REHABIL 2008 Dec;22(12):1137]. Clinical Rehabilitation. 2008. 22:977-986 | No adherence data |
| Evgeniadis, G., Beneka, A., Malliou, P., Mavromoustakos, S., Godolias, G.. Effects of pre- or postoperative therapeutic exercise on the quality of life, before and after total knee arthroplasty for osteoarthritis. Journal of Back & Musculoskeletal Rehabilitation. 2008. 21:161-169 | No adherence data |
| McCarthy, M.. Perioperative immunonutrition in head and neck cancer. #journal#. 2008. #volume#:N.PAG p-N.PAG p | No adherence data |
| Anan, Go, Kaiho, Yasuhiro, Iwamura, Hiromichi, Ito, Jun, Kohada, Yuki, Mikami, Jotaro, Sato, Makoto. Preoperative pelvic floor muscle exercise for early continence after holmium laser enucleation of the prostate: a randomized controlled study. BMC Urology. 2020. 20:1-7 | No adherence data |
| Bauer, Claus Juergen, Findlay, Michael, Koliamitra, Christina, Zimmer, Philipp, Schick, Volker, Ludwig, Sebastian, Gurtner, Geoffrey C., Riedel, Bernhard, Schier, Robert. Preoperative exercise induces endothelial progenitor cell mobilisation in patients undergoing major surgery - A prospective randomised controlled clinical proof-of-concept trial. Heliyon. 2022. 8:e10705 | No adherence data |
| de Almeida, Luane Landim, Mendes Junior, Adriano Fernando, Neto, Jose da Mota, Simoni, Leandro Furtado De, Lopes, Karine Helena Souza, Guimaraes, Paloma Carvalho, Valerio, Brenda Iasmin de Oliveira, Sciascia, Aaron. Pre-Operative Scapular Rehabilitation for Arthroscopic Repair of Traumatic Rotator Cuff Tear: Results of a Randomized Clinical Trial. International journal of sports physical therapy. 2021. 16:216-226 | No adherence data |
| Fernandez-Blanco, Raquel, Rincon-Garcia, David, Valero-Alcaide, Raquel, Atin-Arratibel, Maria Angeles, De Miguel-Diez, Javier, Corrochano-Cardona, Ricardo, Torres-Castro, Rodrigo, Moro-Tejedor, Maria Nieves. Preoperative respiratory therapy in patients undergoing surgery for lung cancer: A randomized controlled trial. Physiotherapy research international : the journal for researchers and clinicians in physical therapy. 2023. 28:e1973 | No adherence data |
| Fors, Maria, Enthoven, Paul, Abbott, Allan, Öberg, Birgitta. Effects of pre-surgery physiotherapy on walking ability and lower extremity strength in patients with degenerative lumbar spine disorder: Secondary outcomes of the PREPARE randomised controlled trial. BMC Musculoskeletal Disorders. 2019. 20:1-11 | No adherence data |
| Franz, Alexander, Ji, Sanghyeon, Bittersohl, Bernd, Zilkens, Christoph, Behringer, Michael. Impact of a Six-Week Prehabilitation With Blood-Flow Restriction Training on Pre- and Postoperative Skeletal Muscle Mass and Strength in Patients Receiving Primary Total Knee Arthroplasty. Frontiers in physiology. 2022. 13:881484 | No adherence data |
| Furon, Yoakim, Dang Van, Simon, Blanchard, Simon, Saulnier, Patrick, Baufreton, Christophe. Effects of high-intensity inspiratory muscle training on systemic inflammatory response in cardiac surgery - A randomized clinical trial. Physiotherapy theory and practice. 2023. #volume#:1-11 | No adherence data |
| Heiman, Jenny, Onerup, Aron, Bock, David, Haglind, Eva, Olofsson Bagge, Roger. The effect of nonsupervised physical activity before and after breast cancer surgery on quality of life: Results from a randomized controlled trial (PhysSURG-B). Scandinavian journal of surgery : SJS : official organ for the Finnish Surgical Society and the Scandinavian Surgical Society. 2022. 111:75-82 | No adherence data |
| Ibrar, I., Shabbir, S., Ahmad, H., Zafar, M., Hassan, T., Waheed, M.. Effects of Pre-Surgical Education and Physical Therapy Training for Dyspnea Prevention in Patients Undergoing Valvular Cardiac Surgery. #journal#. 2022. 16:518 | No adherence data |
| Khalil, L., Jildeh, T., Abbas, M., Buckley, P., Moutzouros, V., Okoroha, K., Tramer, J.. Blood Flow Restriction Therapy Improves Early Patient Reported Outcomes Following ACL Reconstruction. #journal#. 2022. 10:#pages# | No adherence data |
| Kotecha, Harsh, Surme, Shahish, Vieira, Alfven, Velankar, Ameya, Agrawal, Laksh, Shah, Dhruv, Chaudhari, Jaimini. Is prehabilitation a void in management of anterior cruciate ligament injuries? A Prospective study. Journal of Research & Practice on the Musculoskeletal System (JRPMS). 2022. 6:65-71 | No adherence data |
| Labuschagne, Rozelle, Roos, Ronel. Pre-operative physiotherapy for elderly patients undergoing abdominal surgery. The South African journal of physiotherapy. 2022. 78:1782 | No adherence data |
| Nowosielski, K., Zbalski, M., Szostek, P., Szanecki, W.. PREHABILITATION PROGRAM IN OVARIAN CANCER PATIENTS - TOWARDS MORE OBJECTIVE MEASUREMENT OF COMPLIANCE - PRELIMINARY RESULTS. #journal#. 2022. 32:A455 | No adherence data |
| Sadiq, H., Rampam, S., Patel, J., Crawford, S., Walz, M., Kapoor, A.. Preoperative walking intervention did not appear to improve patient-reported postoperative recovery in older adults with frailty traits: Randomized trial. Medicine (United States). 2022. 101:E30689 | No adherence data |
| Sykes, K. J., Gibbs, H., Farrokhian, N., Arthur, A., Flynn, J., Shnayder, Y., Kakarala, K., Nallani, R., Smith, J. B., Penn, J., Fassas, S., Cummings, E., Arambula, Z., Karadaghy, O., Bur, A. M.. POINT: pilot randomized, controlled, preoperative intervention for nutrition trial in head and neck cancer. #journal#. 2022. #volume#:#pages# | No adherence data |
| Tesar, Milan, Kozusnikova, Veronika, Martinek, Lubomir, Durdik, Stefan, Ihnat, Peter. Preoperative nutritional support for patients undergoing elective colorectal cancer surgery - does it really work?. Biomedical papers of the Medical Faculty of the University Palacky, Olomouc, Czechoslovakia. 2022. #volume#:#pages# | No adherence data |
| Tokgoz, Gulfidan, Arman, Nilay, Seyit, Hakan, Karabulut, Mehmet. Effects of pre-surgical aerobic dance-based exercise on lower extremity in people with morbid obesity awaiting bariatric surgery: Randomized controlled study. Clinical obesity. 2022. 12:e12529 | No adherence data |
| Tunçkale, Tamer, Sarıfakıoğlu, Banu, Kavasoğlu, Deniz, Ustaömer, Kübra. Another perspective on lumber spinal stenosis treatment: Should exercise be added to pre-surgical treatment?. Journal of Surgery & Medicine (JOSAM). 2022. 6:912-921 | No adherence data |
| Xia, Xiaoli, Ding, Guirong, Shi, Lingyun, Wang, Meixiang, Tian, Jing. Effects of preoperative walking on bowel function recovery for patients undergoing gynecological malignancy laparoscopy. Precision Medical Sciences. 2022. 11:122-129 | No adherence data |
| Sahar, Wajeeha, Waseem, Mehwish, Riaz, Muhammad, Nazeer, Nouman, Ahmad, Muhammad, Haider, Zulfiqar. EXPRESS: Effects of Prehabilitation Resistance Training in Mild to Moderate Clinically Frail Patients Awaiting Coronary Artery Bypass Graft Surgery.. #journal#. 2023. #volume#:10815589231207795 | No adherence data |
| Bausys, Augustinas, Luksta, Martynas, Anglickiene, Giedre, Maneikiene, Vyte V, Kryzauskas, Marius, Rybakovas, Andrius, Dulskas, Audrius, Kuliavas, Justas, Stratilatovas, Eugenijus, Macijauskiene, Lina, Simbelyte, Toma, Celutkiene, Jelena, Jamontaite, Ieva E, Cirtautas, Alma, Lenickiene, Svetlana, Petrauskiene, Dalia, Cikanaviciute, Evelina, Gaveliene, Edita, Klimaviciute, Gertruda, Rauduvyte, Kornelija, Bausys, Rimantas, Strupas, Kestutis. Effect of home-based prehabilitation on postoperative complications after surgery for gastric cancer: randomized clinical trial.. #journal#. 2023. #volume#:#pages# | No adherence data |
| Chen, Bei, Yan, Xiaoxia, Wang, Xiaojun, Mao, Yanjun. Effectiveness of precise and quantitative rapid pulmonary rehabilitation nursing program for elderly patients with lung cancer during the perioperative period: A randomized controlled trial.. #journal#. 2023. 39:572 | No adherence data |
| Ngo-Huang, An T, Parker, Nathan H, Xiao, Lianchun, Schadler, Keri L, Petzel, Maria Q B, Prakash, Laura R, Kim, Michael P, Tzeng, Ching-Wei D, Lee, Jeffrey E, Ikoma, Naruhiko, Wolff, Robert A, Javle, Milind M, Koay, Eugene J, Pant, Shubham D, Folloder, Justin P, Wang, Xuemei, Cotto, Alicia M, Ju, Ye Rang, Garg, Naveen, Wang, Huamin, Bruera, Eduardo D, Basen-Engquist, Karen M, Katz, Matthew H G. Effects of a Pragmatic Home-based Exercise Program Concurrent With Neoadjuvant Therapy on Physical Function of Patients With Pancreatic Cancer: The PancFit Randomized Clinical Trial.. #journal#. 2023. 278:22 | No adherence data |
| Gunsel-Yildirim, Gokce, Ceylan, Kenan Can, Dikmen, Derya. The effect of perioperative immunonutritional support on nutritional and inflammatory status in patients undergoing lung cancer surgery: a prospective, randomized controlled study.. #journal#. 2023. 31:365 | No adherence data |
| Chen, Juan, Luo, Ai-Lin, Yang, Lin, Wang, Wei, Zhou, Xian, Yang, Mei. Nutrition management by a multidisciplinary team for prevention of nutritional deficits and morbidity following esophagectomy.. #journal#. 2023. 56:e12421 | No adherence data |
| Zhang, Juan, Sun, Wei, Wang, Tingting, Li, Jie, Yu, Min. Effect of preoperative functional exercise on cephalic vein diameter, anastomotic blood flow, and postoperative complications in patients with arteriovenous internal fistuloplasty.. #journal#. 2023. 73:653 | No adherence data |
| Zarate Rodriguez J.G., Cos H., Srivastava R., Bewley A., Raper L., Li D., Dai R., Williams G.A., Fields R.C., Hawkins W.G., Lu C., Sanford D.E., Hammill C.W.. Preoperative levels of physical activity can be increased in pancreatectomy patients via a remotely monitored, telephone-based intervention: A randomized trial. #journal#. 2023. 15:100212 | No adherence data |
| Gillis C., Hasil L., Fenton T.. Pragmatic Prehabilitation for Colorectal Surgery: A Randomized Controlled Trial. #journal#. 2023. 47:S49 | No adherence data |
| Shannon A.H., Hamad A., Gombita R., Sarna A., Patel R., Cloyd J.M., Kim A., Huang H., Pawlik T., Tsung A., Ejaz A.. Implementation and Early Outcomes of a Randomized Controlled Trial of Preoperative Exercise Therapy Before Gastrointestinal Cancer Surgery. #journal#. 2023. 30:S219 | No adherence data |
| Wall C., Glyn T., Rowbotham D., Haines M., Bissett I., Eglinton T., Gearry R.. Randomised feasibility study of preoperative medical nutrition therapy in adults undergoing surgery for Crohn's disease. #journal#. 2023. 17:i899 | No adherence data |
| Sun, Jian-ning, Shan, Yu-zhou, Wu, Li-xia, Li, Ning, Xu, Fei-hu, Kong, Xiang-ru, Zhang, Bei. Preoperative high-intensity strength training combined with balance training can improve early outcomes after total knee arthroplasty.. #journal#. 2023. 18:1 | No adherence data |
| Niazi, Sepideh, Gisour, Bita Bagheri, Ahmadi Tafti, Seyed Hossein, Aliannejad, Rasoul, Khah, Amir Sobhrakhshan, Pourgharib Shahi, Mohammad Hossein. Effectiveness of preoperative respiratory rehabilitation on ICU stay and social pain in patients undergoing heart surgery.. #journal#. 2022. 15:207 | No adherence data |
| Abou-Ashour, HS, El Kased, A, Elsisi, A, Abdelrazek, E, Youssef, FI, Shahin, M. Impact of preoperative nutritional support on patients with gastric cancer surgery. #journal#. 2022. 41:215 | No adherence data |
| Zdziechowski, Adam, Zdziechowska, Magdalena, Rysz, Jacek, Woldanska-Okonska, Marta. The Effectiveness of Preoperative Outpatient and Home Rehabilitation and the Impact on the Results of Hip Arthroplasty: Introductory Report. Healthcare (Basel, Switzerland). 2024. 12:#pages# | No adherence data |
| Ros-Nebot, Bibiana, Rodiera-Olive, Josep, Verdera-Roig, Merce, Tril-Queralt, Cristina, Pradas-Abadia, Andrea, Julian-Gonzalez, Sara, Falco-Pegueroles, Anna. Cognitive Training to Reduce Memory Disturbance Associated With Postoperative Cognitive Impairment After Elective Noncardiac Surgery: An Experimental Study. Journal of perianesthesia nursing : official journal of the American Society of PeriAnesthesia Nurses. 2024. #volume#:#pages# | No adherence data |
| Machado, Pedro, Pimenta, Sara, Garcia, Ana Luis, Nogueira, Tiago, Silva, Sonia, Dos Santos, Claudia Lares, Martins, Maria Vitoria, Canha, Andre, Oliveiros, Barbara, Martins, Raul A., Cruz, Joana. Effect of Preoperative Home-Based Exercise Training on Quality of Life After Lung Cancer Surgery: A Multicenter Randomized Controlled Trial. Annals of surgical oncology. 2024. 31:847-859 | No adherence data |
| Griffin, Sally B., Palmer, Michelle A., Strodl, Esben, Lai, Rainbow, Chuah, Teong L., Burstow, Matthew J., Ross, Lynda J.. Preoperative dietitian-led Very Low Calorie Diet (VLCD) Clinic for adults living with obesity undergoing gynaecology, laparoscopic cholecystectomy and hernia repair procedures: a pilot parallel randomised controlled trial. The British journal of nutrition. 2024. 131:1436-1446 | No adherence data |
| Enriquez-Schmidt, Javier, Mautner Molina, Camila, Kalazich Rosales, Mariana, Munoz, Maximiliano, Ruiz-Uribe, Matias, Fuentes Leal, Francisca, Monrroy Uarac, Manuel, Carcamo Ibaceta, Carlos, Fazakerley, Daniel J., Larance, Mark, Ehrenfeld, Pamela, Martinez-Huenchullan, Sergio. Moderate-intensity constant or high-intensity interval training? Metabolic effects on candidates to undergo bariatric surgery. Nutrition, metabolism, and cardiovascular diseases : NMCD. 2024. #volume#:#pages# | No adherence data |
| Brahmbhatt, P., Hong, N. J. L., Sriskandarajah, A., Alavi, N., Selvadurai, S., Berger-Richardson, D., Lemon-Wong, S., Mascarenhas, J., Gibson, L., Rapier, T., Isenberg-Grzeda, E., Bernstein, L. J., Mina, D. S., Wright, F. C.. A Feasibility Randomized Controlled Trial of Prehabilitation During Neoadjuvant Chemotherapy for Women with Breast Cancer: A Mixed Methods Study. Annals of Surgical Oncology. 2024. #volume#:11 | No adherence data |
| Triguero, C., aacute, novas, D., oacute, pez, Rodr, iacute, guez-Arias, F., oacute, mez, Mart, iacute, nez, M., aacute, nchez, Guill, eacute, n, L., Peris, Castell, oacute, Alcaide, Quir, oacute, s, M. J., Morillas-Blasco, P., Arroyo, A., Ram, iacute, rez, J. M.. Home-based prehabilitation improves physical conditions measured by ergospirometry and 6MWT in colorectal cancer patients: a randomized controlled pilot study. Supportive care in cancer. 2023. 31:673 | No adherence data |
| St-Pierre, J., Coca-Martinez, M., Drummond, K., Minnella, E., Ferri, L., Scheede-Bergdahl, C., Carli, F.. PREHABILITATION DURING NACT IN ESOPHAGEAL CANCER CARE-A FEASIBILITY STUDY. Diseases of the Esophagus. 2023. 36:66 | No adherence data |
| Obukhova, O., Volf, L., Egofarov, N. M., Kolesnichenko, M., Kirillov, Y., Povaga, S., Perminov, Y., Skorokhod, A.. Impact of perioperative high-protein nutritional support on postoperative outcomes in the treatment of primary lung cancer: a multicenter comparative low-intervention study. Clinical nutrition ESPEN. 2023. 58:461 | No adherence data |
| Kumar, Sushil, Parshad, Sanjeev, Bijyal, Sajan, Mittal, Gourav, Sikka, Gitanjali. A Comparison of Two Methods of Pre-operative Inspiratory Muscle Training on Post-operative Outcome Following Esophagectomy. Indian journal of surgical oncology. 2023. 14:956-962 | No adherence data |
| Khorrami, M. H., Mohseni, A., Gholipour, F., Alizadeh, F., Zargham, M., Izadpanahi, M. H., Sichani, M., Khorrami, F.. Single session pre-operative pelvic floor muscle training with biofeedback on urinary incontinence and quality of life after radical prostatectomy: a randomized controlled trial. Urological science. 2023. 34:23 | No adherence data |
| Harvey, James, Tolerico, Paul H., Bell, Theodore, Mason, Lara, McKinney, Heather, Shaeffer, Christine, Kashyap, Rahul. PREHABILITATION FOR PATIENTS UNDERGOING TRANSCATHETER AORTIC VALVE REPLACEMENT: A PILOT RANDOMIZED CLINICAL TRIAL. Journal of the American College of Cardiology (JACC). 2023. 81:868-868 | No adherence data |
| Estalella, L., Espina, B., Guasch, A., Moline, A., Renzulli, M., Pavel, M. C., Llacer-Millan, E., Pueyo, E., Ramirez, E., Memba, R., Jorba, R.. Multimodal Prehabilitation during Neoadjuvant Chemotherapy in Patients with Colorectal Liver Metastases: preliminary Results. HPB. 2023. 25:S389 | No adherence data |
| Ortega J, Cassinello N. Rovira L. Mayte B. Julio L.. Preoperative respiratory physiotherapy can improve oxygenation during bariatric surgery. Obesity surgery. 2013. 23:1077 | No adherence data |
| Moreno J, Rovira L. Hernandez J. Ballester M. Belda J. Llorens J.. Effect of preoperatory chest physiotherapy program on the oxigenation, after laparoscopic bariatric surgery. European journal of anaesthesiology. 2012. 29:88 | No adherence data |
| Kasvis P, Bui T. Kilgour R. Carli F. Vigano A.. A multimodal prehabilitation program in hepato-pancreato-biliary cancer patients awaiting surgery: preliminary results. A multimodal prehabilitation program in hepato-pancreato-biliary cancer patients awaiting surgery: preliminary results. 2018. Conference: 2018 Joint Meeting of the Multinational Association of Supportive Care in Cancer, MASCC and the International Society of Oral Oncology, ISOO. Austria. 26:S392 | No adherence data |
| Bassi, S., Tafuri, A., Sebben, M., Pirozzi, M., Balzarro, M., Porcaro, A. B., Artibani, W., Cerruto, M. A.. Preliminary disappointed results from a pilot randomized trial of preoperative pelvic floor muscle training versus usual care to improve continence and HR-QOL after RARP. Neurourology and Urodynamics. 2018. 37:S6-S7 | No adherence data |
| Sethi, Sifut, Ravindhran, Bharadhwaj, Long, Judith, Gurung, Roji, Huang, Chao, Smith, George E, Carradice, Daniel, Wallace, Tom, Ibeggazene, Said, Chetter, Ian C, Pymer, Sean. A preoperative supervised exercise program potentially improves long-term survival after elective abdominal aortic aneurysm repair.. #journal#. 2023. #volume#:#pages# | No adherence data |
| Fernandes, L., Roos, E. M., Overgaard, S., Villadsen, A., Soegaard, R.. Supervised neuromuscular exercise prior to hip or knee replacement: Cost-utility analysis alongside a randomised controlled trial. Osteoarthritis and Cartilage. 2015. 23:A35-A36 | No adherence data |
| Baumgarten, H., Steinmetz, C., Borst, C., Walther, T., Walther, C.. Preoperative exercise training before elective coronary artery bypass graft surgery: A prospective randomized evaluation on feasibility and effects on operative outcomes. Thoracic and Cardiovascular Surgeon. 2017. 65:#pages# | No adherence data |
| Skoffer, Birgit, Maribo, Thomas, Mechlenburg, Inger, Hansen, Per M., Soballe, Kjeld, Dalgas, Ulrik. Efficacy of Preoperative Progressive Resistance Training on Postoperative Outcomes in Patients Undergoing Total Knee Arthroplasty. Arthritis care & research. 2016. 68:1239-51 | No adherence data |
| Chakravartty, S., Sidhu, P., Vivian, G., Patel, A. G.. Randomised controlled trial on pre-operative liver shrinking diet on peri-operative outcomes in patients undergoing gastric bypass: Is it time to change our practice?. Obesity Surgery. 2014. 24:1141 | No adherence data |
| Lee, H. J., Na, J. R., Suh, Y. S., Kong, S. H., Yang, H. K.. Effect of perioperative oral nutritional supplementation in malnourished patients who will receive gastrectomy: A prospective randomized trial. Clinical Nutrition. 2014. 33:S249 | No adherence data |
| Banerjee, S., Manley, K., Shaw, B., Thomas, L., Rochester, M., Saxton, J., Mills, R.. Is preoperative exercise to improve fitness before cystectomy feasible? Results from a randomised controlled study. British Journal of Surgery. 2014. 101:59-59 | No adherence data |
| Barberan-Garcia, A., Ubre, M., Pascual-Argente, N., Risco, R., Faner, J., Balust, J., Lacy, A. M., Puig-Junoy, J., Roca, J., Martinez-Palli, G.. Post-discharge impact and cost-consequence analysis of prehabilitation in high-risk patients undergoing major abdominal surgery: secondary results from a randomised controlled trial. British journal of anaesthesia. 2019. 123:450-456 | No adherence data |
| Lai, Yutian, Huang, Jian, Yang, Mei, Su, Jianhua, Liu, Jing, Che, Guowei. Seven-day intensive preoperative rehabilitation for elderly patients with lung cancer: a randomized controlled trial. The Journal of surgical research. 2017. 209:30-36 | No adherence data |
| Kaya, S. O., Ilker Akcam, T., Ceylan, K. C., Samancilar, O., Ozturk, O., Usluer, O.. Is preoperative protein-rich nutrition effective on postoperative outcome in non-small cell lung cancer surgery? A prospective randomized study. Interactive Cardiovascular and Thoracic Surgery. 2015. 21:#pages# | No adherence data |
| Gade, H., Friborg, O., Sandbu, R., Rosenvinge, J., Hjelmesaeth, J.. Long-term follow-up (4 years) of patients receiving cognitive behavioural therapy (CBT) or usual care before bariatric surgery: A randomized controlled trial. Obesity Facts. 2017. 10:38 | No adherence data |
| Hulzebos, E. H. J., van Meeteren, N. L. U., van den Buijs, B. J. W., de Bie, R. A., de la Rivière, A. B., Helders, P. J. M.. Feasibility of preoperative inspiratory muscle training in patients undergoing coronary artery bypass surgery with a high risk of postoperative pulmonary complications: a randomized controlled pilot study. Clinical Rehabilitation. 2006. 20:949-959 | No adherence data |
| Valkenet K, Trappenburg J. C. A. Hulzebos E. H. van Meeteren N. L. U. Backx F. J. G.. Effects of a pre-operative home-based inspiratory muscle training programme on perceived health-related quality of life in patients undergoing coronary artery bypass graft surgery. Physiotherapy (united kingdom). 2016. (no pagination):#pages# | No adherence data |
| Van Bokhorst-de Van der Schuer, M. A., Langendoen, S. I., Vondeling, H., Kuik, D. J., Quak, J. J., Van Leeuwen, P. A.. Perioperative enteral nutrition and quality of life of severely malnourished head and neck cancer patients: a randomized clinical trial. Clinical nutrition (Edinburgh, Scotland). 2000. 19:437-44 | No adherence data |
| Yulia Argunova Y, Pomeshkina S. A. Moskin E. G. Sogoyan N. Barbarash O. L.. Effects of prehabilitation program on quality of life and adherence to therapy in patients undergoing coronary artery bypass grafting. European journal of preventive cardiology. 2019. 26:S35 | No adherence data |
| Cunha LFCD, Baixinho CL, Henriques MA, Sousa LMM, Dixe MDA. Evaluation of the effectiveness of an intervention in a health team to prevent falls in hospitalized elderly people. Rev Esc Enferm USP. 2021;55:e03695. Published 2021 May 31. doi:10.1590/S1980-220X2019031403695 | No adherence data |
| Kaushik, D., Shah, P., Mukherjee, N., Ji, N., Kumar, P. A., Thompson, I. M., Mansour, A., Jha, R., Yang, X., Wang, H., Darby, N., Rivero, J. R., Svatek, R. S., Liss, M. A.. A phase ii randomized clinical trial of yoga in men with prostate cancer. Journal of Urology. 2021. 206:e1175-e1176 | No adherence data |
| Shahood, H., Pakai, A., Kiss, R., Bory, E., Szilagyi, N., Sandor, A., Boncz, I., Verzar, Z.. The Effect of Preoperative Chest Physiotherapy on Oxygenation and Lung Functions Among Cardiac Surgery Patients: A Randomized Controlled Study. Value in Health. 2022. 25:S306 | No adherence data |
| Soler-Silva, A., Triguero, D., Sanchis-Lopez, A., Lario-Perez, S., Lillo, C., Lopez-Rodriguez Arias, F., Sanchez-Guillen, L., Oller, I., Aranaz, V., Alcaide, M. J., Arroyo, A.. Does the performance of a multimodal pre-habilitation protocol gives patients with colorectal cancer better quality life after surgery?. Surgical Endoscopy. 2022. 36:S358-S359 | No adherence data |
| Patel, YS, Sullivan, KA, Churchill, IF, Beauchamp, MK, Wald, J, Mbuagbaw, L, Fahim, C, Hanna, WC. Preconditioning program reduces the incidence of prolonged hospital stay after lung cancer surgery: Results from the Move For Surgery randomized clinical trial. #journal#. 2023. #volume#:#pages# | No adherence data |
| Garcia Rs, Paz A. L. Brage M. I. Y. Moolhuyzen E. G. Rioboo M. S. Mate J. M. B.. Does preoperative exercise training prevent functional decline after video-assisted thoracic surgery?. European respiratory journal. 2016. 48:#pages# | No adherence data |
| Ligibel Ja, Irwin M. Dillon D. Barry W. Giobbie-Hurder A. Frank E. Winer E. P. McTiernan A. Cornwell M. Pun M. Brown M. Jeselsohn R.. Impact of pre-operative exercise on breast cancer gene expression. Cancer research. 2017. 77:#pages# | No adherence data |
| Bernardi, K., Olavarria, O. A., Dhanani, N. H., Lyons, N., Holihan, J. L., Cherla, D. V., Berger, D. H., Ko, T. C., Kao, L. S., Liang, M. K.. Two-year Outcomes of Prehabilitation Among Obese Patients With Ventral Hernias A Randomized Controlled Trial (NCT02365194). Annals of Surgery. 2022. 275:288-294 | No adherence data |
| Anan, G., Iwamura, H., Ito, J., Kaiho, Y., Sato, M.. Preoperative pelvic floor muscle exercise for early continence after holmium laser enucleation of the prostate: A randomized controlled study. Journal of Urology. 2019. 201:e463-e464 | No adherence data |
| bw, R. B. R.. Treatment before and after shoulder muscle injury surgery. Rehabilitation pre-and post-operative of Rotator Cuff injury. 2019. #volume#:#pages# | No adherence data |
| Rampam, Sanjeev, Sadiq, Hammad, Patel, Jay, Meyer, David, Uy, Karl, Yates, Jennifer, Schanzer, Andres, Movahedi, Babak, Lindberg, James, Crawford, Sybil, Gurwitz, Jerry, Mazor, Kathleen, Stefan, Mihaela, White, Daniel, Walz, Matthias, Kapoor, Alok. Supervised preoperative walking on increasing early postoperative stamina and mobility in older adults with frailty traits: A pilot and feasibility study. Health science reports. 2022. 5:e738 | No adherence data |
| Tesar, M., Ostruzska, P., Kozusnikova, V., Martinek, L., Zadrapova, M., Grasslova, L., Ihnat, P.. Preoperative non-selective administration of nutritional supplements to patients undergoing elective colorectal resection - standard of perioperative care?. Rozhledy v chirurgii : mesicnik Ceskoslovenske chirurgicke spolecnosti. 2022. 101:232-238 | No adherence data |
| Machado, P. F. A., Pimenta, S., Garcia, A. L., Nogueira, T., Silva, S., Dos Santos, C. L., Martins, M. V., Canha, A., Oliveiros, B., Martins, R. A., Cruz, J.. ASO Visual Abstract: Effect of Preoperative Home-Based Exercise Training on Quality of Life After Lung Cancer Surgery: A Multicenter Randomized Controlled Trial. Annals of surgical oncology. 2024. 31:902-903 | No adherence data |

**Appendix 7. Trial Risk of Bias**

| **Author, year** | **Sequence Generation** | **Allocation Concealment** | **Blinding of participants and personnel** | **Blinding of outcome assessors** | **Blinding of standard care participants** | **Blinding for Harms** | **Incomplete Outcome data** | **Selective Outcome reporting** | **Other sources of bias** | **Overall RoB** |
| --- | --- | --- | --- | --- | --- | --- | --- | --- | --- | --- |
| Birch, 2020 |  |  |  |  |  |  |  |  |  |  |
| Blackwell, 2020 |  |  |  |  |  |  |  |  |  |  |
| Moug, 2018 |  |  |  |  |  |  |  |  |  |  |
| Granicher, 2020 |  |  |  |  |  |  |  |  |  |  |
| Holsgaard-Larsen, 2020 |  |  |  |  |  |  |  |  |  |  |
| Hollis, 2019 |  |  |  |  |  |  |  |  |  |  |
| Northgraves, 2016 |  |  |  |  |  |  |  |  |  |  |
| Blasco, 2020 |  |  |  |  |  |  |  |  |  |  |
| O'Gara, 2020 |  |  |  |  |  |  |  |  |  |  |
| Laurent, 2020 |  |  |  |  |  |  |  |  |  |  |
| Liu, 2020 |  |  |  |  |  |  |  |  |  |  |
| Liu, 2020 |  |  |  |  |  |  |  |  |  |  |
| Milios, 2019 |  |  |  |  |  |  |  |  |  |  |
| Karlsson, 2019 |  |  |  |  |  |  |  |  |  |  |
| Valkenet, 2018 |  |  |  |  |  |  |  |  |  |  |
| Hjelmesaeth, 2019 |  |  |  |  |  |  |  |  |  |  |
| Ruiz-Tovar, 2019 |  |  |  |  |  |  |  |  |  |  |
| Mudge, 2018 |  |  |  |  |  |  |  |  |  |  |
| Palma-Milla, 2016 |  |  |  |  |  |  |  |  |  |  |
| Lindback, 2017 |  |  |  |  |  |  |  |  |  |  |
| Tew, 2017 |  |  |  |  |  |  |  |  |  |  |
| Ida, 2017 |  |  |  |  |  |  |  |  |  |  |
| Licker, 2016 |  |  |  |  |  |  |  |  |  |  |
| Lai, 2019 |  |  |  |  |  |  |  |  |  |  |
| Huang, 2017 |  |  |  |  |  |  |  |  |  |  |
| Rolving, 2016 |  |  |  |  |  |  |  |  |  |  |
| Dunne, 2016 |  |  |  |  |  |  |  |  |  |  |
| Kalarchian, 2016 |  |  |  |  |  |  |  |  |  |  |
| Ruiz-Tovar, 2015 |  |  |  |  |  |  |  |  |  |  |
| Huber, 2015 |  |  |  |  |  |  |  |  |  |  |
| Zeng, 2014 |  |  |  |  |  |  |  |  |  |  |
| Jensen, 2014 |  |  |  |  |  |  |  |  |  |  |
| van Adrichem, 2014 |  |  |  |  |  |  |  |  |  |  |
| Falewee, 2014 |  |  |  |  |  |  |  |  |  |  |
| Matassi, 2014 |  |  |  |  |  |  |  |  |  |  |
| Morano, 2013 |  |  |  |  |  |  |  |  |  |  |
| Brown, 2012 |  |  |  |  |  |  |  |  |  |  |
| McKay, 2012 |  |  |  |  |  |  |  |  |  |  |
| Oosting, 2012 |  |  |  |  |  |  |  |  |  |  |
| Parikh, 2012 |  |  |  |  |  |  |  |  |  |  |
| Burden, 2011 |  |  |  |  |  |  |  |  |  |  |
| Benzo, 2011 |  |  |  |  |  |  |  |  |  |  |
| Bitterli, 2011 |  |  |  |  |  |  |  |  |  |  |
| Carli, 2010 |  |  |  |  |  |  |  |  |  |  |
| Hoogeboom, 2010 |  |  |  |  |  |  |  |  |  |  |
| Nielsen, 2008 |  |  |  |  |  |  |  |  |  |  |
| Tibaek, 2009 |  |  |  |  |  |  |  |  |  |  |
| Rooks, 2006 |  |  |  |  |  |  |  |  |  |  |
| Beaupre, 2003 |  |  |  |  |  |  |  |  |  |  |
| Wang, 2002 |  |  |  |  |  |  |  |  |  |  |
| Moya, 2016 |  |  |  |  |  |  |  |  |  |  |
| Baillot, 2017 |  |  |  |  |  |  |  |  |  |  |
| Woodfield, 2022 |  |  |  |  |  |  |  |  |  |  |
| Schulz, 2021 |  |  |  |  |  |  |  |  |  |  |
| McLean, 2022 |  |  |  |  |  |  |  |  |  |  |
| Onerup, 2022 |  |  |  |  |  |  |  |  |  |  |
| Hernon, 2021 |  |  |  |  |  |  |  |  |  |  |
| Tenconi, 2021 |  |  |  |  |  |  |  |  |  |  |
| Przkora, 2021 |  |  |  |  |  |  |  |  |  |  |
| Steffens, 2021 |  |  |  |  |  |  |  |  |  |  |
| Mathew, 2021 |  |  |  |  |  |  |  |  |  |  |
| Heiman, 2021 |  |  |  |  |  |  |  |  |  |  |
| Aragoncillo Sauco, 2021 |  |  |  |  |  |  |  |  |  |  |
| Ferreira, 2020 |  |  |  |  |  |  |  |  |  |  |
| Domínguez‑Navarro, 2020 |  |  |  |  |  |  |  |  |  |  |
| Minnella, 2021 |  |  |  |  |  |  |  |  |  |  |
| Minnella, 2020 |  |  |  |  |  |  |  |  |  |  |
| Contreras, 2018 |  |  |  |  |  |  |  |  |  |  |
| Soares, 2013 |  |  |  |  |  |  |  |  |  |  |
| Gillis, 2016 |  |  |  |  |  |  |  |  |  |  |
| Carli, 2020 |  |  |  |  |  |  |  |  |  |  |
| van Nieuwenhove, 2011 |  |  |  |  |  |  |  |  |  |  |
| Minella, 2018 |  |  |  |  |  |  |  |  |  |  |
| Marchand, 2021 |  |  |  |  |  |  |  |  |  |  |
| Barth, 2019 |  |  |  |  |  |  |  |  |  |  |
| Gillis, 2014 |  |  |  |  |  |  |  |  |  |  |
| Humeidan, 2020 |  |  |  |  |  |  |  |  |  |  |
| Gravier, 2021 |  |  |  |  |  |  |  |  |  |  |
| Bousquet-Dion, 2018 |  |  |  |  |  |  |  |  |  |  |
| Santa Mina, 2018 |  |  |  |  |  |  |  |  |  |  |
| Gade, 2016 |  |  |  |  |  |  |  |  |  |  |
| Dronkers, 2010 |  |  |  |  |  |  |  |  |  |  |
| Allen, 2021 |  |  |  |  |  |  |  |  |  |  |
| Diaz-Feijoo, 2022 |  |  |  |  |  |  |  |  |  |  |
| Klek, 2022 |  |  |  |  |  |  |  |  |  |  |
| McIsaac, 2022 |  |  |  |  |  |  |  |  |  |  |
| Nguyen, 2022 |  |  |  |  |  |  |  |  |  |  |
| Paul, 2022 |  |  |  |  |  |  |  |  |  |  |
| Serrano, 2022 |  |  |  |  |  |  |  |  |  |  |
| Tan, 2023 |  |  |  |  |  |  |  |  |  |  |
| Kasvis, 2023 |  |  |  |  |  |  |  |  |  |  |
| Singh, 2023 |  |  |  |  |  |  |  |  |  |  |
| Atoui, 2023 |  |  |  |  |  |  |  |  |  |  |
| Akowuah, 2023 |  |  |  |  |  |  |  |  |  |  |
| Molenaar, 2023 |  |  |  |  |  |  |  |  |  |  |
| Reynolds, 2015 |  |  |  |  |  |  |  |  |  |  |
| Yau, 2021 |  |  |  |  |  |  |  |  |  |  |
| Gill, 2009 |  |  |  |  |  |  |  |  |  |  |
| Lluch, 2017 |  |  |  |  |  |  |  |  |  |  |
| Nguyen, 2022 |  |  |  |  |  |  |  |  |  |  |
| Waller, 2022 |  |  |  |  |  |  |  |  |  |  |
| Li, 2024 |  |  |  |  |  |  |  |  |  |  |
| Granicher, 2024 |  |  |  |  |  |  |  |  |  |  |
| Wall, 2000 |  |  |  |  |  |  |  |  |  |  |
| Bojesen, 2023 |  |  |  |  |  |  |  |  |  |  |

**
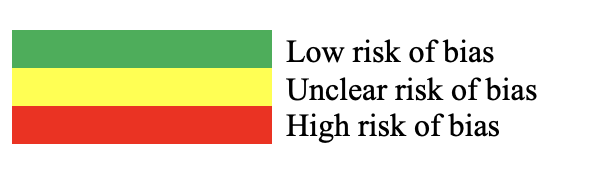
**

**Appendix 8. Credibility of Effect Modification Analyses (ICEMAN) in a meta-analysis of randomized controlled trials**

**Predictor: Age (Binary adherence)**

| **1: Is the analysis of effect modification based on comparison within rather than between trials?** | | | |
| --- | --- | --- | --- |
| [ **x** ] Completely between | [ ] Mostly between or unclear | [ ] Mostly within | [ ] Completely within |
| *Subgroup analysis or meta-regression comparing overall effects of each individual trial. This is typical for aggregate data meta-analysis.* | *Subgroup analysis or meta-regression with most information coming from overall effects, but some trials providing within-trial subgroup information* | *Most trials providing within-trial subgroup information; or individual participant data analysis that combines within and between trial information* | *All trials providing within-trial subgroup information or individual participant data; and the analysis separates within from between trial information, e.g., meta-analysis of interactions* |
|  | | | |
| **2: For within-trial comparisons, is the effect modification similar from trial to trial?** [ **x** ] Not applicable: no or one within-RCT comparison | | | |
| [ ] Definitely not similar | [ ] Probably not similar or unclear | [ ] Mostly similar | [ ] Definitely similar |
| *Effect modification reported for two or more trials and clearly different directions* | *Effect modification not reported for individual trials or too imprecise to tell* | *Effect modification reported for two or more trials, mostly similar in direction, but considerable differences in magnitude* | *Effect modification reported for two or more trials, similar in direction, only some differences in magnitude* |
|  | | | |
| **3: For between-trial comparisons, is the number of trials large?** [ ] Not applicable: no between RCT comparison | | | |
| [ ] Very small | [ ] Rather small or unclear | [ ] Rather large | [ **x** ] Large |
| *1 or 2 or in smallest subgroup; 5 or less in continuous meta-regression* | *3-4 in smallest subgroup; 6-10 in continuous meta-regression* | *5-9 in smallest subgroup; 11 to 15 in continuous meta-regression* | *10 or more in smallest subgroup; more than 15 in continuous meta-regression* |
|  | | | |
| **4: Was the direction of effect modification correctly hypothesized a priori?** | | | |
| [ ] Definitely no | [ **x** ] Probably no or unclear | [ ] Probably yes | [ ] Definitely yes |
| *Clearly post-hoc or results inconsistent with hypothesized direction or biologically very implausible* | *Vague hypothesis or hypothesized direction unclear* | *No prior protocol available but unequivocal statement of a priori hypothesis with correct direction of effect modification* | *Prior protocol available and includes correct specification of direction of effect modification, e.g., based on a biologic rationale* |
|  | | | |
| **5: Does a test for interaction suggest that chance is an unlikely explanation of the apparent effect modification?** (consider irrespective of number of effect modifiers) | | | |
| [ ] Chance a very likely explanation | [ **x** ] Chance a likely explanation or unclear | [ ] Chance may not explain | [ ] Chance an unlikely explanation |
| *Interaction or meta-regression p-value >0.05* | *Interaction or meta-regression p-value ≤0.05 and >0.01, or no test of interaction reported and not computable* | *Interaction or meta-regression p-value ≤0.01 and >0.005* | *Interaction or meta-regression p-value ≤0.005* |
|  | | | |
| **6: Did the authors test only a small number of effect modifiers or consider the number in their statistical analysis?** | | | |
| [ **x** ] Definitely no | [ ] Probably no or unclear | [ ] Probably yes | [ ] Definitely yes |
| *Explicitly exploratory analysis or large number of effect modifiers tested (e.g., greater than 10) and multiplicity not considered in analysis* | *No mention of number or 4-10 effect modifiers tested and number not considered in analysis* | *No protocol available but unequivocal statement of 3 or fewer effect modifiers tested* | *Protocol available and 3 or fewer effect modifiers tested or number considered in analysis* |
|  | | | |
| **7: Did the authors use a random effects model?** | | | |
| [ ] Definitely no | [ ] Probably no or unclear | [ ] Probably yes | [ **x** ] Definitely yes |
| *Fixed (or common) effect or fixed effects model explicitly stated* | *Probably fixed effect(s) model* | *Probably random (or mixed) effects* | *Random (or mixed) effects explicitly stated* |
|  | | | |
| **8: If the effect modifier is a continuous variable, were arbitrary cut points avoided?** [ ] not applicable: not continuous | | | |
| [ ] Definitely no | [ ] Probably no or unclear | [ ] Probably yes | [ **x** ] Definitely yes |
| *Analysis based on exploratory cut point(s), e.g., picking cut point associated with highest interaction p-value* | *Analysis based on cut point(s) of unclear origin* | *Analysis based on pre-specified cut point(s), e.g., suggested by prior RCT* | *Analysis based on the full continuum, e.g., assuming a linear or logarithmic relationship* |
|  | | | |
| **9 Optional: Are there any additional considerations that may increase or decrease credibility?** (manual section 3.9) [ ] not applicable | | | |
|  | [ **x** ] Yes, probably decrease | [ ] Yes, probably increase | |
| \|  \| \| \| --- \| --- \| \|  \| **x** \| \| \| \| \|  \| \|  \|  \| \|  \|  \| \| \| \| \|  \| \|  \|  \| \| \| \| \|  \| \|  \| **Very low credibility** \| \| **Low credibility** \| **Moderate credibility** \| **High credibility** \|  \| \|  \|  \| \|  \|  \|  \|  \| \|  \| Minimal to no support for effect modification.  Use overall effect for each subgroup \| \| Some but insufficient support for effect modification.  Use overall effect for each subgroup but note remaining uncertainty \| Likely effect modification.  Use separate effects for each subgroup but note remaining uncertainty \| Very likely effect modification.  Use separate effects for each subgroup \|  \| \| Comment: \| \| \| \| \| \| \| | | | |

Advanced age was a statistically predictor associated with lower prehabilitation adherence (lower among older participants); however, low in credibility. Per year older: OR 0.95 (95%CI 0.91 to 0.99); R^2^ 13.30%, *P*-value = 0.025

**Appendix 9. Frequency of themes and overarching quotes for barriers and facilitators**

**Section 1: Frequency of themes and overarching quotes for barriers to prehabilitation.**

| **Theme** | **Frequency counts (%)** | **Quotes** |
| --- | --- | --- |
| **Health condition** | 28 (26.6) | *“I didn’t have enough energy.”*  *"My hip problems prevented me from*  *performing the exercises."* |
| **Personal factors** | 11 (10.4) | *“I had difficulty focusing.”*  *“Too overwhelmed by surgery and/or recovery.”*  *The frequency of games was too often.”*  *“I was too busy.”* |
| **Logistical issues** | 7 (6.6) |  |
| **Social influences** | 9 (8.5) |  |

**Section 2: Frequency of themes and overarching quotes for facilitators to prehabilitation**

| **Theme** | **Frequency counts (%)** | **Quotes** |
| --- | --- | --- |
| **Social influences/support** | 3 (2.8) |  |
| **Supervision by specialists** | 23 (21.9) | *“It was interesting to get exercise help from a specialist. It made me feel as I was being taken care of, and thus I have exercised more to show that I can.”*  *“I live alone and have no relatives, so the social contact with the physiotherapist was invaluable in addition to the meaningful exercise.”* |
| **Training locations including home-based programmes** | 10 (9.5) |  |
| **Personalisation** | 22 (20.9) |  |
| **Adequate access to materials, supplements and equipment** | 11 (10.4) |  |
| **Resources to support engagement and motivation** | 15 (14.2) | *“It was positive to challenge myself and get a little breathless. It was interesting to be able to participate, and that I got help to improve my fitness. A very good coach/instructor.”*  *“I enjoyed playing the training games.”* |
